# Copper-catalyzed multicomponent reactions for the efficient synthesis of diverse spirotetrahydrocarbazoles

**DOI:** 10.3762/bjoc.18.80

**Published:** 2022-07-07

**Authors:** Shao-Cong Zhan, Ren-Jie Fang, Jing Sun, Chao-Guo Yan

**Affiliations:** 1 College of Chemistry & Chemical Engineering, Yangzhou University, Yangzhou 225002, China

**Keywords:** Diels–Alder reaction, indole, indolo-2,3-quinodimethane, Levy reaction, tetrahydrocarbazole, spirooxindole

## Abstract

In the presence of copper sulfate, three- or four-component reactions of 2-methylindole, aromatic aldehydes and various cyclic dienophiles in refluxing toluene afforded diverse spirotetrahydrocarbazoles. This reaction is an important development of the Levy reaction by using 2-methylindole to replace ethyl indole-2-acetate and successfully provides facile access to important polysubstituted spiro[carbazole-3,3'-indolines], spiro[carbazole-2,3'-indolines], spiro[carbazole-3,5'-pyrimidines] and spiro[carbazole-3,1'-cycloalkanes] in satisfactory yields and with high diastereoselectivity.

## Introduction

**Scheme 1:**
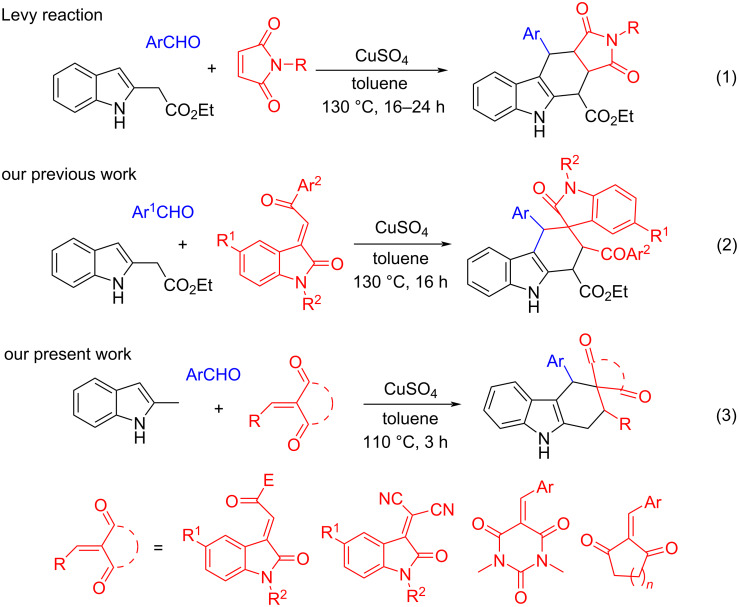
Construction of diverse tetrahydrocarbazoles via Levy-type reaction.

Tetrahydrocarbazole is one of the most privileged heterocyclic core structures. It widely exists in various naturally occurring alkaloids and pharmacologically active compounds exhibiting important bioactivities such as antitumor activity and antiprotein kinase C activity [1–3]. Additionally, the corresponding carbazole derivatives also show important applications in various functional materials [4–7]. Owing to their remarkable significance, developing convenient synthetic protocols for functionalized tetrahydrocarbazoles has attracted continual attention in synthetic and pharmaceutical chemistry [8–15]. Among many well-designed strategies for the synthesis of tetrahydrocarbazoles, the direct assembly of the tetrahydrocyclohexenyl ring with readily available functionalized indoles as precursors has proven to be one of the most efficient synthetic protocols [16–21]. Therefore, many [4 + 2] reactions of 3-vinylindolines or 2-vinylindolines with diverse dienophiles have been successfully developed for the synthesis of many tetrahydrocarbazole and carbazole derivatives [22–50]. On the other hand, the Diels–Alder reaction of the in situ generated indole-2,3-quinodimethanes with various dienophiles is also a powerful method for rapid construction of functionalized tetrahydrocarbazoles [51–68]. In this respect, Lévy reported a copper-catalyzed three-component reaction of aromatic aldehydes, ethyl indole-2-acetate and *N*-alkylmaleimides for the efficient construction of polycyclic tetrahydrocarbazoles, in which indolo-2,3-quinodimethane intermediate was initially generated and sequentially underwent a [4 + 2] cycloaddition reaction (reaction 1 in Scheme 1) [69–74]. This metal-catalyzed one-pot reaction not only combined the advantages of a traditional Diels–Alder reaction and the recently developed multicomponent reactions, but also meets the goal of green and sustainable chemistry. Recently, we have reported the efficient construction of a series of heterocyclic spiro compounds including tetrahydrospiro[carbazole-3,3’-indolines] by using the Levy three-component reaction of indole-2-acetate, aromatic aldehydes and various cyclic dienophiles such as 3-phenacylideneoxindoles (reaction 2 in Scheme 1) [75–77]. It was noticed that only ethyl indole-2-acetate was successfully employed as the precursor to generate the active indolo-2,3-quinodimethane intermediate in the Levy reaction, which greatly limited its practical synthetic value. We envisioned that other substituted indoles without electron-withdrawing activating groups could be employed in the Levy reaction, which will greatly develop the potential synthetic applications of the Levy-type reaction. Herein, we wish to report a Levy-type reaction by using readily available 2-methylindole to replace ethyl indole-2-acetate for the efficient synthesis of diverse spiro tetrahydrocarbazoles. In the presence of CuSO_4_, the multicomponent reactions of aromatic aldehydes, 2-methylindole and various cyclic dieneophiles such as 3-phenacylideneoxindoles, isatylidene malononitriles and the in situ generated 5-arylidene-1,3-dimethylbarbituric acids or 2-arylidene-1,3-cycloketones efficiently afforded diverse cyclic spirotetrahydrocarbazoles in good yields and with high diastereoselectivity (reaction 3 in Scheme 1).

## Results and Discussion

Initially, (*E*)-1-benzyl-3-(2-oxo-2-phenylethylidene)indolin-2-one, benzaldehyde, and 2-methylindole served as model substrates for the optimization of reaction conditions. Inspired by our previous work [75–77], we first screened different catalysts (Table 1, entries 1−5) with toluene as the solvent and found that CuSO_4_ was the best choice, providing **1a** in 58% isolated yield. Notably, the yield of the byproduct **1a’** was also obtained in less than 5% yield, which indicated the high diastereoselectivity of this reaction. Several other solvents such as EtOH and MeCN were explored, the product **1a** was formed in lower yields (Table 1, entries 6 and 7). Importantly, the reaction also proceeded smoothly when the temperature was reduced to 90 °C and with better yield at 110 °C (Table 1, entries 8 and 9). Moreover, different reaction times and the catalyst loadings were also examined (Table 1, entries 10–12), but no better results were observed. Therefore, a mixture of 2-methylindole (0.5 mmol), benzaldehyde (0.6 equiv), and 3-methyleneoxindole (1.0 equiv) with CuSO_4_ (0.1 mmol) as the catalyst in toluene (10.0 mL) reacting at 110 °C for 3 h were selected as the optimal reaction conditions.

**Table 1:** Optimization of reaction conditions^a^.

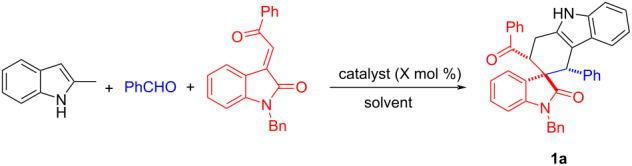					

Entry	Catalyst	Solvent	Time (h)	Temp. (°C)	Yield (%)^b^

1	FeCl_3_	toluene	3	130	<10
2	CuI	toluene	3	130	<10
3	Cu(OTf)_2_	toluene	3	130	30
4	Cu(OAc)_2_	toluene	3	130	25
5	CuSO_4_	toluene	3	130	58
6	CuSO_4_	EtOH	3	130	30
7	CuSO_4_	MeCN	3	130	40
**8**	**CuSO** **_4_**	**toluene**	**3**	**110**	**61**
9	CuSO_4_	toluene	3	90	50
10	CuSO_4_	toluene	6	110	61
11^c^	CuSO_4_	toluene	3	110	58
12^d^	CuSO_4_	toluene	3	110	61

^a^Reaction conditions: 2-methylindole (0.5 mmol), aldehyde (0.6 mmol), 3-phenacylideneoxindole (0.5 mmol), catalyst (0.1 mmol), solvent (10.0 mL). ^b^Isolated yields. ^c^CuSO_4_ (0.05 mmol). ^d^CuSO_4_ (0.2 mmol).

**Scheme 2:**
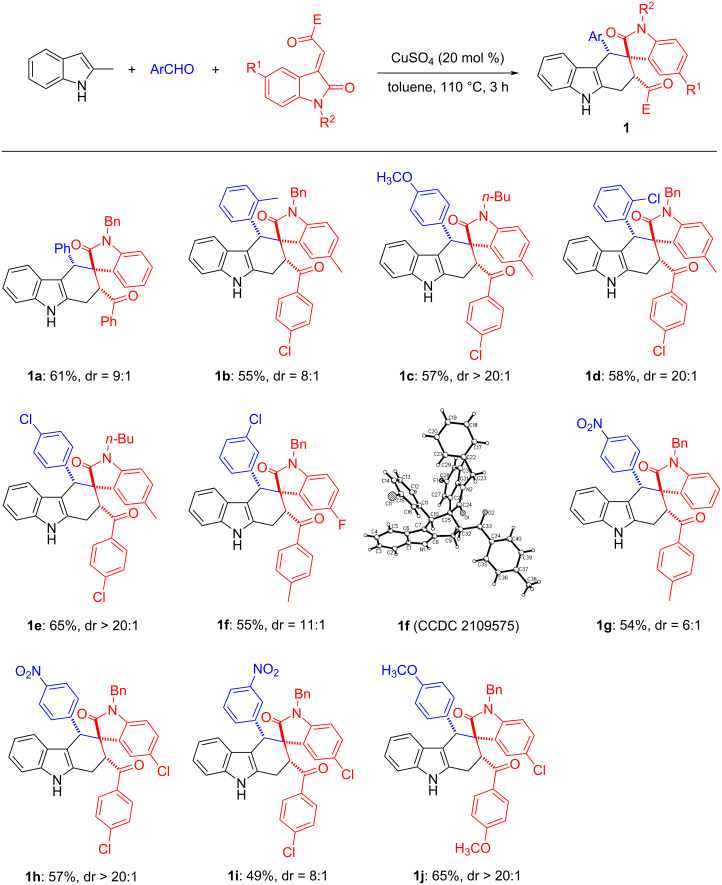
Synthesis of spiro[carbazole-3,3'-inolines]. Reaction conditions: 2-methylindole (0.5 mmol), aromatic aldehydes (0.6 mmol), 3-phenacylideneoxindole (0.5 mmol), CuSO_4_ (0.10 mmol), toluene (6.0 mL), 110 °C, 3 h. Isolated yields are shown. The dr values were determined by ^1^H NMR.

Having established the optimal reaction conditions, we first sought to determine the generality of the aromatic aldehydes. As illustrated in Scheme 2, the reaction usually resulted in a mixture of major isomers **1a**–**j** and the minor isomers **1a’**–**j’** with the molecular ratios of 6:1 to 20:1. Aromatic aldehydes with both electron-donating and electron-withdrawing groups were compatible. This reaction tolerated many kinds of functional groups, including Me (**1b**), OMe (**1c** and **1j**), Cl (**1d**, **1e** and **1f**), and NO_2_ (**1g**, **1h** and **1i**) groups. Besides different aromatic aldehydes, we further tested the generality of various 3-phenacylideneoxindoles. Unsurprisingly, a wide range of 3-methyleneoxindoles containing different substituents at various positions (**1e**–**j**) reacted smoothly to give the corresponding products with good to excellent diastereoselectivity. A protecting *n*-butyl group (**1e**) also could promote such a transformation with good yield and high diastereoselectivity. All major isomers **1a**–**j** and some minor isomers **1a’, 1b’, 1f’, 1g’, 1j’** were successfully isolated and fully characterized. Notably, the relative configuration of major isomer **1f** (CCDC 2109575) was determined by X-ray crystallographic analysis, in which the *m-*chlorophenyl, benzoyl and the phenyl group in oxindole are in *cis-*configuration. It has been known that the benzoyl group and the phenyl group in oxindole stand in *cis*-position in the starting 3-phenacylideneoxindoles [75]. Therefore, a concerted Diels–Alder reaction should be involved in this three-component reaction.

**Scheme 3:**
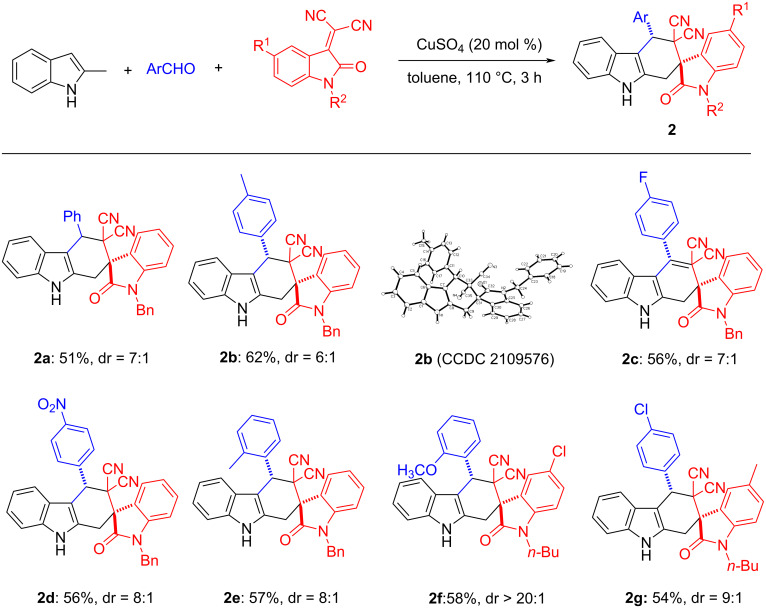
Synthesis of spiro[carbazole-2,3'-indolines]. Reaction conditions: 2-methylindole (0.5 mmol), aromatic aldehyde (0.6 mmol), isatylidene malononitrile (0.5 mmol), CuSO_4_ (0.10 mmol), toluene (6.0 mL), 110 °C, 3 h. Isolated yields are shown. The dr values were determined by ^1^H NMR.

On the basis of this success, we further considered whether other dienophiles could be applied in such catalytic system. Under the same reaction conditions, the three-component reaction of 2-methylindole, benzaldehyde and 2-(1-benzyl-2-oxoindolin-3-ylidene)malononitrile reacted smoothly to give the expected spiro[carbazole-2,3'indolines] in 51% yield with a diastereometric ratio (dr) value of 7:1. As shown in Scheme 3, a wide range of aromatic aldehydes with different substituents on the aromatic ring reacted smoothly to give the desired products **2a**–**e** in good yields and with good to excellent diastereoselectivity. On the other hand, we also tested various isatylidene malononitriles, substrates with a differently substituted indole ring or with different protecting groups on the indole N-1 position (**2f**, **2g**). All substrates reacted smoothly to give the corresponding products in moderate to good yields and with high stereoselectivity. It should be pointed out that the minor products **2a’**–**e’** and **2g’** were also isolated and fully characterized.

Moreover, the relative configuration of major isomer **2b** (CCDC 2109576) and minor isomer **2g’** (CCDC 2109577) were determined by X-ray crystallographic analysis. In the major isomer **2b**, the phenyl group at C4-position is in *cis*-position to the aryl group in the oxindole scaffold, while these two groups exist in *trans*-configuration in the minor isomer **2g’**.

**Scheme 4:**
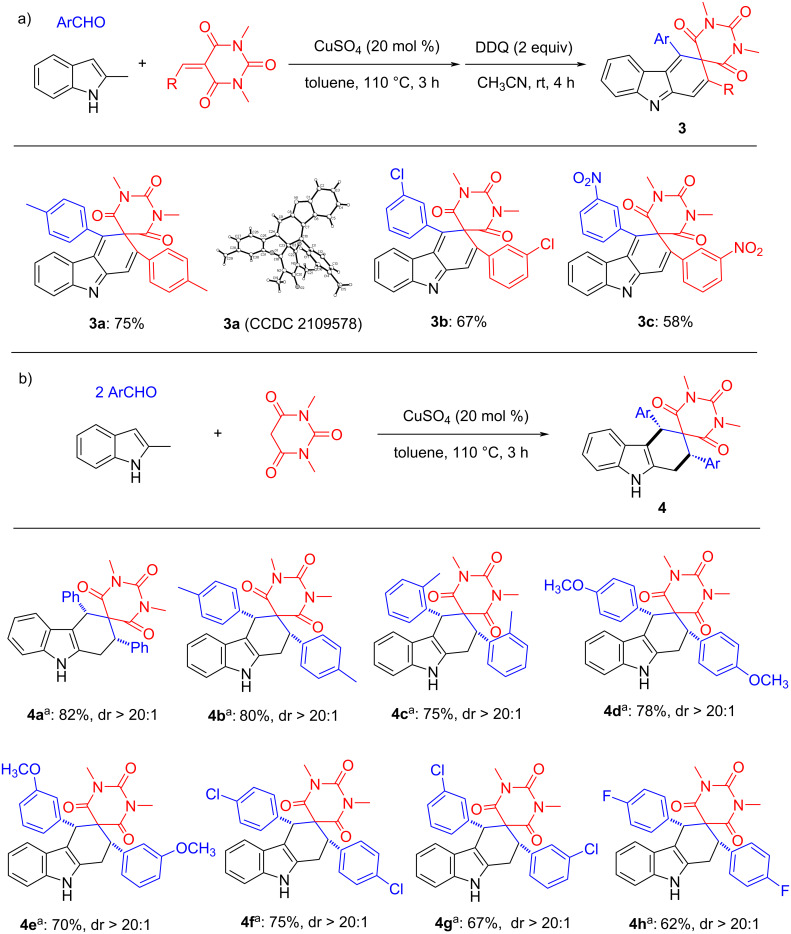
Synthesis of tetrahydrospiro[carbazole-3,5'-pyrimidines]. Reaction conditions: 2-methylindole (0.5 mmol), aromatic aldehyde (0.6 mmol), 5-arylidene-1,3-dimethylbarbituric acid (0.5 mmol), CuSO_4_ (0.1 mmol), toluene (6.0 mL), 110 °C, 3 h, and then DDQ (1.0 mmol), CH_3_CN (10. 0 mL), rt, 2 h. Isolated yields are shown. The dr values were determined by ^1^H NMR. ^a^Reaction conditions: 2-methylindole (0.5 mmol), aromatic aldehyde (1.2 mmol), 1,3-dimethylbarbituric acid (0.5 mmol), CuSO_4_ (0.1 mmol), toluene (6.0 mL), 110 °C, 3 h.

5-Arylidene-1,3-dimethylbarbituric acids, as good dienophiles, could also react smoothly in such a catalytic system (Scheme 4a). Firstly, the three-component reaction of 2-methylindole, 5-arylidene-1,3-dimethylbarbituric acid and 4-methylbenzaldehyde under the standard reaction conditions and further oxidation with DDQ generated the final aromatized product **3a** in 75% yield. Under the same reaction conditions, similar aromatized products **3b** and **3c** were also produced in good yields with different kinds of aromatic aldehydes and 5-arylidene-1,3-dimethylbarbituric acids. 5-Arylidene-1,3-dimethylbarbituric acids could be easily generated through Knoevenagel condensation of aromatic aldehydes and 1,3-dimethylbarbituric acid under the catalysis of Lewis acid. We envisioned whether the desired dienophiles, 5-arylidene-1,3-dimethylbarbituric acids, could be in situ generated by aromatic aldehydes and 1,3-dimethylbarbituric acid under the standard conditions. Therefore, the four-component reaction was carried out by using two molecular aromatic aldehydes, 1,3- dimethylbarbituric acid and 2-methylindole utilizing CuSO_4_ as the catalyst in toluene at 110 °C. The desired product **4a** was obtained in 82% with excellent diastereoselectivity (dr > 20:1). In this reaction, both dienes (*o*-QDMs) and dienophiles were in situ formed from the starting material. As shown in Scheme 4b, a wide range of aromatic aldehydes that bore different substituents at the *ortho-* (**4c**), *meta-* (**4e**, **4g**) and *para-*position (**4b**, **4d**, **4f** and **4h**) of the phenyl ring reacted smoothly to give the corresponding products **4a**–**h** in moderate to good yields with excellent diastereoselectivity (dr > 20:1). The single crystal structures of the compound **3a** (CCDC 2109578) and **4e** (CCDC 2109579) were successfully determined by X-ray diffraction. It is pleased to find that the obtained spiro[carbazole-3,5'-pyrimidines] **4a**–**h** have same *cis*-configuration to that of the above prepared spiro[carbazole-3,3'-indolines] **1a**–**j** and spiro[carbazole-2,3'-indolines] **2a**–**h**, in which the two phenyl groups exist in *cis*-position. This result clearly indicated that this reaction has a similar outcome of stereochemistry.

**Scheme 5:**
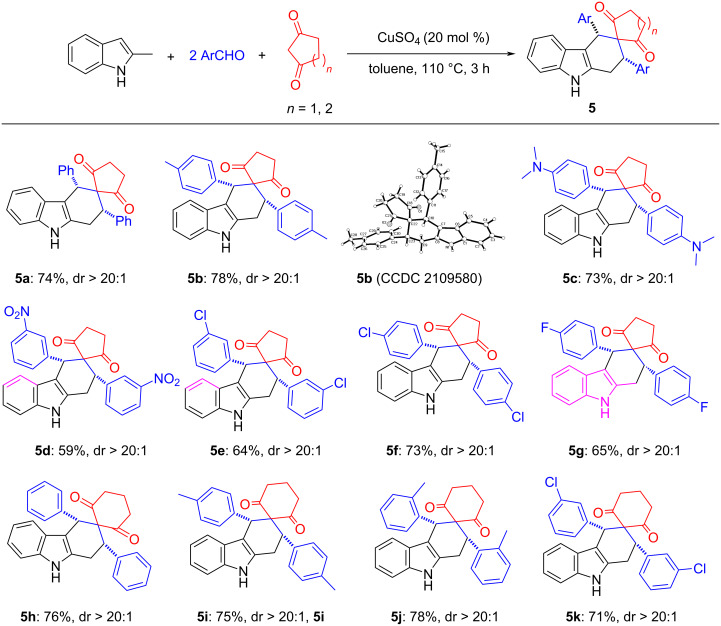
Synthesis of tetrahydrospiro[carbazole-3,1'-cycloalkane]-diones. Reaction conditions: 2-methylindole (0.5 mmol), aromatic aldehyde (1.0 mmol), cyclic 1,3-dione (0.5 mmol), CuSO_4_ (0.1 mmol), toluene (6.0 mL), 110 °C, 3 h. Isolated yields are shown. The dr values were determined by ^1^H NMR.

Based on the success of the four-component reaction through in situ generation of dienes and dienophiles, we further considered whether more common cyclic 1,3-diones could be applied in this catalytic system. To our delight, when cyclopentane-1,3-dione was used to replace 1,3-dimethylbarbituiric acid under the same conditions, the desired product **5a** was obtained in 74% yield with excellent diastereoselectivity (dr > 20:1). Furthermore, we examined the scope of aromatic aldehydes (Scheme 5), different substituents on the aromatic ring showed marginal effect on the yields. The spiro compounds **5a**–**g** were successfully produced in moderate to excellent yields with excellent diastereoselectivity (dr > 20:1). Cyclohexane-1,3-dione also reacted smoothly to produce the corresponding products **5h**–**k** in high yields with high diastereoselectivity. The single crystal structure of compound **5b** (CCDC 2109580) clearly indicated that the two aryl groups are in *cis*-position.

**Scheme 6:**
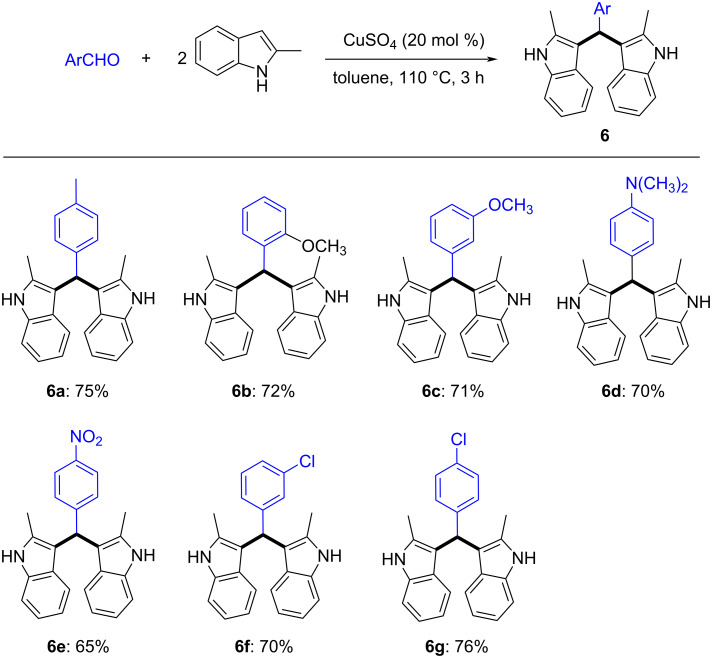
Synthesis of 3,3'-(arylmethylene)bis(2-methyl-1*H*-indole). Reaction conditions: 2-methylindole (1.0 mmol), aromatic aldehyde (0.5 mmol), CuSO_4_ (0.1 mmol), toluene (6.0 mL), 110 °C, 3 h. Isolated yields are shown.

When we did not add the dienophiles to this system, 4-methylbenzaldehyde could react with two molecules of 2-methylindole under the same reaction conditions to generate the well-known 3,3'-(*p*-tolylmethylene)bis(2-methylindole) (**6a**) in 75% yield. Subsequently, we sought to determine the generality of aromatic aldehydes. As illustrated in Scheme 6, a diversity of functional groups, which include Me (**6a**), OMe (**6b**, **6c**), NMe_2_ (**6d**), NO_2_ (**6e**), and Cl (**6f**, **6g**), could be well tolerated in this reaction. Importantly, the reaction is not sensitive to the electronic properties of the arenes, as the substrates bearing either electron-donating (**6a**–**d**), or electron-withdrawing (**6e**–**g**) groups on the aryl ring were all transformed smoothly into the corresponding desired products **6a–g** with good to excellent yields. Though 3,3'-(*p*-tolylmethylene)bisindoles have been previously prepared by acid-catalyzed reaction of aromatic aldehydes and various indoles. Here we also provided an alternative synthetic protocol by Lewis acid CuSO_4_ catalyzed reaction.

**Scheme 7:**
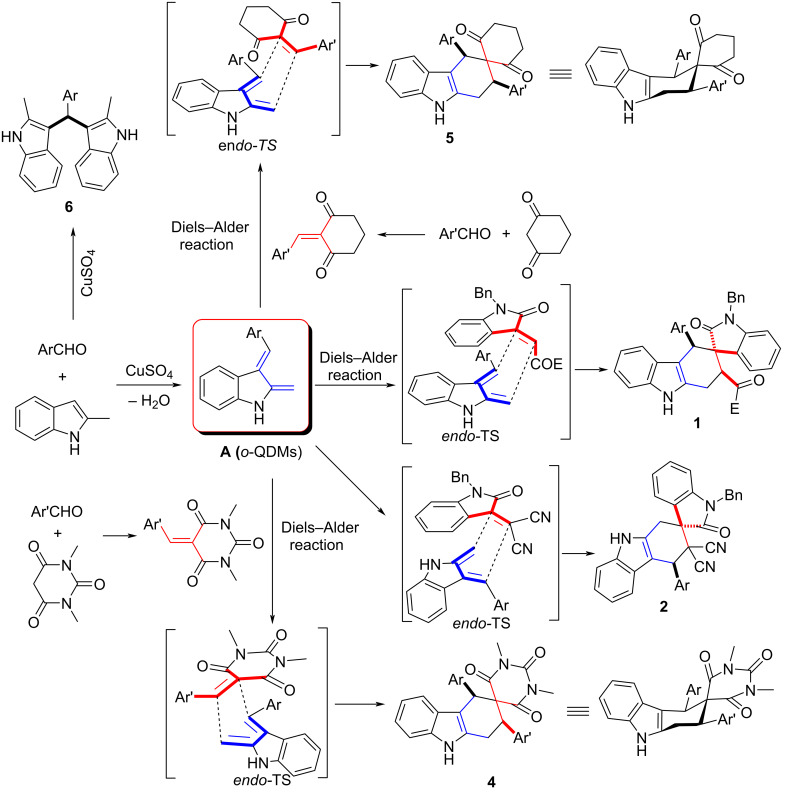
Proposed reaction mechanism for the multicomponent reaction.

Based on the above experimental results and the previously works [75–77], a plausible reaction pathway is illustrated in Scheme 7. At first, 2-methylindole reacts with aromatic aldehydes in the presence of the catalyst CuSO_4_ to generate the intermediate 3-substituted indole, which undergoes dehydration to form the key intermediate indole-based *ortho*-quinodimethanes (*o*-QDMs, **A**). In the meantime, the cyclic 1,3-diones and aromatic aldehyde undergo Knoevenagel condensation to afford the different kinds of dienophiles. Subsequently, the Diels–Alder cycloaddition between the indole-based *ortho*-quinodimethanes (*o*-QDMs, **A**) and dienophiles affords the final spiro compounds **1**, **2**, **4** and **5** as major isomers through an *endo*-transition state. Due to the different polarity, 3-phenacylideneoxindole and isatylidene malononitrile resulted in regioisomeric spiro[carbazole-3,3'-indoline] **1** and spiro[carbazole-2,3'-indoline] **2** as the final products. It can be seen that the two aryl groups at 1,3-positions actually exist on the *e*-bonds in the newly formed cyclohexyl ring in the spiro compounds **4** and **5**. This also means that the major isomers **4** and **5** are the thermodynamically stable isomers. In the major isomer **1**, the aryl group and the benzoyl group at 1,3-position also stand on the *e*-bonds, which indicated that the major isomer **1** is also belonging to the thermodynamically stable isomer. This result showed that this reaction is a thermodynamically controlled reaction. On the other hand, the tetrahydrospiro[carbazole-3,5'-pyrimidine] **4** can be converted to aromatized spiro[carbazole-3,5'-pyrimidine] **3** through the oxidation of DDQ. In the absence of the effective dienophile, the normal Friedel–Crafts alkylation of 2-methylindole with aromatic aldehyde gives the well-known 3,3'-(arylmethylene)bis(2-methylindoles) **6**.

## Conclusion

In summary, we have developed a copper-catalyzed multicomponent Diels–Alder reaction of 2-methylindole, aromatic aldehydes and cyclic 1,3-diones through in situ generated dienes and dienophiles under the same conditions. These strategies are sustainable, general and practical, which providing facile access to important polysubstituted spiro[carbazole-3,3'-indolines], spiro[carbazole-2,3'-indolines], spiro[carbazole-3,5'-pyrimidines] and spiro[carbazole-3,1'-cycloalkanes] with good yields and high diastereoselectivity. This reaction actually developed the practical synthetic values of the well-known Levy reaction by using inactivated indole derivatives. The outcome of diastereoselectivity of the reaction was clearly elucidated by determination of the several single crystal structures and the analysis of the reaction mechanism. The experimental results indicate the in situ generation of both indole-based *ortho-*quinodimethanes (*o*-QDMs) as active dienes and cyclic dienophiles are the key intermediates in these reactions. Moreover, this protocol exhibited good functional group tolerance, broad substrate scope and facile scalability. It would provide great potential for applications in organic synthesis, pharmaceutical chemistry and materials science.

## Experimental

**1. General procedure for the preparation of the spiro[carbazole-3,3'-inolines] 1a–j** and **1a’**–**j’:** A mixture of 2-methyl-1*H*-indole (0.5 mmol, 1.0 equiv), aldehyde (0.6 mmol, 1.2 equiv), 3-methyleneoxindole (0.5 mmol, 1.0 equiv) and CuSO_4_ (0.1 mmol, 0.2 equiv) in dry toluene (6.0 mL) was stirred at 110 °C for about three hours. After removing the solvent by evaporating at reduced pressure, the residue was subjected to column chromatography with ethyl acetate and light petroleum (v/v = 1:5–1:8) as eluent to give pure **1a**–**j** and **1a’**–**j’**.

**2-Benzoyl-1'-benzyl-4-phenyl-1,2,4,9-tetrahydrospiro[carbazole-3,3'-indolin]-2'-one (1a):** Purple solid, 61%, mp 182–185 °C; ^1^H NMR (400 MHz, CDCl_3_) δ 8.23 (s, 1H, NH), 7.89 (d, *J* = 7.2 Hz, 1H, ArH), 7.54 (t, *J* = 7.2 Hz, 1H, ArH), 7.43–7.39 (m, 3H, ArH), 7.30 (d, *J* = 8.0 Hz, 1H, ArH), 7.23–7.15 (m, 2H, ArH), 7.14–7.08 (m, 5H, ArH), 6.99 (t, *J* = 7.6 Hz, 1H, ArH), 6.83–6.73 (m, 3H, ArH), 6.63–6.62 (m, 2H, ArH), 6.36 (d, *J* = 7.6 Hz, 1H, ArH), 6.28 (t, *J* = 7.2 Hz, 2H, ArH), 5.00 (s, 1H, CH), 4.83 (dd, *J*_1_ = 12.4 Hz, *J*_2_ = 5.2 Hz, 1H, CH), 4.60 (d, *J* = 16.0 Hz, 1H, CH), 4.46 (d, *J* = 16.0 Hz, 1H, CH), 3.49 (t, *J* = 12.4 Hz, 1H, CH), 3.26 (dd, *J*_1_ = 16.8 Hz, *J*_2_ = 5.2 Hz, 1H, CH); ^13^C NMR (100 MHz, CDCl_3_) δ 199.3, 178.0, 143.6, 136.6, 136.5, 136.2, 135.2, 133.3, 132.3, 130.9, 130.0, 128.7, 128.6, 128.4, 128.2, 127.9, 127.7, 127.0, 126.9, 126.7, 126.5, 125.6, 121.8, 121.5, 120.2, 119.1, 110.7, 110.7, 109.0, 56.1, 50.0, 48.8, 43.6, 25.4; IR(KBr) υ: 3367, 3210, 3155, 3017, 2980, 2831, 2864, 1877, 1623, 1611, 1507, 1456, 1355, 1241, 1178, 1143, 955, 931, 849, 789 cm^−1^; HRMS–ESI (*m*/*z*): [M + Na]^+^ calcd for C_39_H_30_N_2_O_2_, 581.2199; found, 581.2191.

**2. General procedure for the preparation of the spiro[carbazole-2,3'-indolines] 2a**–**g** and **2a’**–**g’:** A mixture of 2-methyl-1*H*-indole (0.5 mmol, 1.0 equiv), aldehyde (0.6 mmol, 1.2 equiv), 2-(1-benzyl-2-oxoindolin-3-ylidene)malononitrile (0.5 mmol, 1.0 equiv) and CuSO_4_ (0.1 mmol, 0.2 equiv) in dry toluene (6.0 mL) was stirred at 110 °C for about three hours. After removing the solvent by evaporating at reduced pressure, the residue was subjected to column chromatography with ethyl acetate and light petroleum (v/v =1:5–1:8) as eluent to give pure **2a**–**g** and **2a’**–**g’**.

**1'-Benzyl-2'-oxo-4-phenyl-4,9-dihydrospiro[carbazole-2,3'-indoline]-3,3(1*H*)-dicarbonitrile (2a):** White solid, 51%, mp 201–204 °C; ^1^H NMR (400 MHz, CDCl_3_) δ 8.27 (s, 1H, NH), 7.52–7.46 (m, 3H, ArH), 7.43–7.38 (m, 4H, ArH), 7.35–7.28 (m, 5H, ArH), 7.20 (t, *J* = 7.6 Hz, 1H, CH), 6.95–6.90 (m, 4H, ArH), 6.54 (d, *J* = 8.0 Hz, 1H, CH), 5.14 (d, *J* = 15.6 Hz, 1H, CH), 4.96 (d, *J* = 15.6 Hz, 1H, CH), 4.89 (s, 1H, CH), 4.10 (dd, *J*_1_ = 16.4 Hz, *J*_2_ = 2.4 Hz, 1H, CH), 2.96 (d, *J* = 16.4 Hz, 1H, CH); ^13^C NMR (100 MHz, CDCl_3_) δ 173.2, 136.6, 134.7, 133.6, 130.9, 129.4, 128.8, 128.0, 127.6, 125.0, 123.6, 122.7, 120.2, 120.1, 112.7, 111.0, 110.4, 106.9, 51.8, 47.6, 46.2, 44.7, 29.1; IR(KBr) υ: 3355, 3207, 3117, 3048, 2963, 2831, 2167, 1871, 1641, 1633, 1554, 1431, 1370, 1240, 1131, 1100, 972, 961, 881, 764 cm^−1^; HRMS–ESI (*m*/*z*): [M + Na]^+^ calcd for C_34_H_24_N_4_O, 527.1842; found, 527.1849.

**3. General procedure for the preparation of the tetrahydrospiro[carbazole-3,5'-pyrimidines] 3a–c:** A mixture of 2-methyl-1*H*-indole (0.5 mmol, 1.0 equiv), aldehyde (0.6 mmol, 1.2 equiv), 5-arylidene-1,3-dimethylbaribituric acid (0.5 mmol, 1.0 equiv) and CuSO_4_ (0.1 mmol, 0.2 equiv) in dry toluene (6.0 mL) was stirred at 110 °C for about three hours. After removing the solvent by evaporating at reduced pressure, the mixture of the above obtained product and DDQ (1.0 mmol, 0.227 g, 2.0 equiv) in dry acetonitrile (10.0 mL) was stirred at room temperature for about four hours. After removing the solvent by evaporating at reduced pressure, the residue was subjected to column chromatography with ethyl acetate and light petroleum (v/v = 1:3–1:6) as eluent to give pure products **3a**–**c**.

**1',3'-Dimethyl-2,4-di-*p*-tolyl-2'*H*-spiro[carbazole-3,5'-pyrimidine]-2',4',6'(1'*H*,3'*H*)-trione (3a):** Yellow solid, 75%, mp 190–192 °C; ^1^H NMR (400 MHz, CDCl_3_) δ 7.59 (d, *J* = 7.6 Hz, 1H, ArH), 7.35–7.30 (m, 4H, ArH), 7.25–7.17 (m, 2H, ArH), 7.04 (s, 1H, ArH), 6.96–6.91 (m, 4H, ArH), 6.35 (d, *J* = 7.6 Hz, 1H, CH), 3.10 (s, 3H, CH_3_), 3.07 (s, 3H, CH_3_), 2.37 (s, 3H, CH_3_), 2.34 (s, 3H, CH_3_); ^13^C NMR (100 MHz, CDCl_3_) δ 165.3, 162.7, 157.9, 149.7, 149.0, 147.3, 142.6, 139.1, 138.7, 137.1, 136.0, 133.7, 130.6, 130.3, 130.0, 129.1, 128.7, 128.3, 128.0, 126.3, 126.1, 125.5, 124.7, 124.2, 123.1, 120.8, 29.6, 28.8, 21.4, 21.3; IR (KBr) υ: 3219, 3158, 3043, 2966, 2900, 1843, 1755, 1648, 1617, 1537, 1466, 1358, 1318, 1266, 1150, 987, 899, 765 cm^−1^; HRMS–ESI (*m*/*z*): [M + Na]^+^ calcd for C_31_H_25_N_3_O_3_, 510.1788; found, 510.1788.

**4. General procedure for the preparation of the spiro[carbazole-3,3'-inolines] 4a**–**h:** A mixture of 2-methyl-1*H*-indole (0.5 mmol, 1.0 equiv), aldehyde (1.2 mmol, 1.2 equiv), 1,3-dimethylbaribituric acid (0.5 mmol, 1.0 equiv) and CuSO_4_ (0.1 mmol, 0.2 equiv) in dry toluene (6.0 mL) was stirred at 110 °C for about three hours. After removing the solvent by evaporating at reduced pressure, the residue was subjected to column chromatography with ethyl acetate and light petroleum (v/v = 1:5–1:8) as eluent to give pure **4a**–**h**.

**1',3'-Dimethyl-2,4-diphenyl-1,2,4,9-tetrahydro-2'*H*-spiro[carbazole-3,5'-pyrimidine]-2',4',6'(1'*H*,3'*H*)-trione (4a):** Purple solid, 82%, mp 205–208 °C; ^1^H NMR (400 MHz, CDCl_3_) δ 8.02 (s, 1H, NH), 7.30–7.27 (m, 2H, ArH), 7.25–7.24 (m, 2H, ArH), 7.24–7.22 (m, 2H, ArH), 7.19–7.15 (m, 3H, ArH), 7.15–7.10 (m, 1H, ArH), 7.06 (t, *J* = 7.2 Hz, 1H, ArH), 6.94 (d, *J* = 7.2 Hz, 1H, ArH), 6.80 (t, *J* = 8.0 Hz, 1H, ArH), 6.41 (d, *J* = 8.0 Hz, 1H, ArH), 5.24 (s, 1H, CH), 4.13 (dd, *J*_1_ = 12.0 Hz, *J*_2_ = 5.6 Hz, 1H, CH), 3.90–3.82 (m, 1H, CH), 3.05 (dd, *J*_1_ = 16.4 Hz, *J*_2_ = 5.6 Hz, 1H, CH), 2.97 (s, 3H, CH_3_), 2.81 (s, 3H, CH_3_); ^13^C NMR (100 MHz, CDCl_3_) δ 171.7, 167.8, 150.0, 138.9, 137.2, 136.0, 134.6, 129.3, 128.9, 128.7, 128.2, 128.2, 128.1, 128.1, 128.1, 126.3, 121.0, 119.7, 118.9, 110.6, 107.5, 62.5, 51.1, 48.2, 28.3, 27.8, 27.7; IR (KBr) υ: 3407, 3078, 2981, 1873, 1744, 1658, 1667, 1582, 1466, 1356, 1321, 1221, 1180, 912, 833 cm^−1^; HRMS–ESI (*m*/*z*): [M + Na]^+^ calcd for C_29_H_25_N_3_O_3_, 486.1788; found, 486.1794.

**5. General procedure for the preparation of the tetrahydrospiro[carbazole-3,1'-cycloalkane]-diones 5a–k:** A mixture of 2-methyl-1*H*-indole (0.5 mmol, 1.0 equiv), aldehyde (1.2 mmol, 2.4 equiv), 1,3-diones (0.5 mmol, 1.0 equiv) and CuSO_4_ (0.1 mmol, 0.2 equiv) in dry toluene (6.0 mL) was stirred at 110 °C for about three hours. After removing the solvent by evaporating at reduced pressure, the residue was subjected to column chromatography with ethyl acetate and light petroleum (v/v = 1:5–1:8) as eluent to give pure **5a–k**.

**2,4-Diphenyl-1,2,4,9-tetrahydrospiro[carbazole-3,1'-cyclopentane]-2',5'-dione (5a):** Purple solid, 74%, mp 178–180 °C; ^1^H NMR (400 MHz, CDCl_3_) δ 8.02 (s, 1H, NH), 7.30 (d, *J* = 8.4 Hz, 1H, ArH), 7.28–7.27 (m, 2H, ArH), 7.26–7.25 (m, 1H, ArH), 7.22 (d, *J* = 8.0 Hz, 1H, ArH), 7.19–7.17 (m, 2H, ArH), 7.14 (t, *J* = 7.6 Hz, 1H, ArH), 7.06 (t, *J* = 7.2 Hz, 1H, ArH), 6.92 (d, *J* = 8.0 Hz, 1H, ArH), 6.79 (t, *J* = 7.2 Hz, 1H, ArH), 6.39 (d, *J* = 8.0 Hz, 1H, ArH), 4.87 (s, 1H, CH), 3.97–3.90 (m, 1H, CH), 3.66 (dd, *J*_1_ = 12.4 Hz, *J*_2_ = 4.8 Hz, 1H, CH), 2.95 (dd, *J*_1_ = 16.0 Hz, *J*_2_ = 4.8 Hz, 1H, CH), 2.01–1.90 (m, 2H, CH_2_), 1.39–1.31 (m, 1H, CH), 1.26–1.22 (m, 1H, CH); ^13^C NMR (100 MHz, CDCl_3_) δ 218.2, 216.7, 139.1, 137.8, 136.1, 134.7, 129.9, 129.5, 128.8, 128.7, 128.5, 128.2, 127.8, 127.5, 126.3, 121.0, 119.7, 118.9, 110.7, 107.8, 65.9, 48.8, 47.4, 37.3, 36.5, 26.5; IR (KBr) υ: 3200, 3173, 3064, 2978, 1861, 1745, 1677, 1631, 1567, 1467, 1382, 1311, 1254, 1132, 960, 841, 781 cm^−1^; HRMS–ESI (*m*/*z*): [M + Na]^+^ calcd for C_28_H_23_NO_2_, 428.1621; found, 428.1626.

## Supporting Information

The crystallographic data of the compounds **1f** (CCDC 2109575), **2b** (CCDC 2109576), **2g’** (CCDC 2109577), **3a** (CCDC 2109578), **4e** (CCDC 2109579) and **5b** (CCDC 2109580) have been deposited at the Cambridge Crystallographic Database Center (http://www.ccdc.cam.ac.uk).

File 1 Characterization data and ^1^H NMR, ^13^C NMR, HRMS spectra of the compounds.
