# Supplementary material for: Copper-catalyzed multicomponent reactions for the efficient synthesis of diverse spirotetrahydrocarbazoles
Source: Beilstein J Org Chem. 2022 Jul 7;18:796–808. doi: 10.3762/bjoc.18.80 (PMC9273986; doi:10.3762/bjoc.18.80)
Supplement: File 1 — Characterization data and 1H NMR, 13C NMR, HRMS spectra of the compounds. [file Beilstein_J_Org_Chem-18-796-s001.pdf]

BEILSTEIN JOURNAL OF ORGANIC CHEMISTRY

Supporting Information

for

Copper-catalyzed multicomponent reactions for the efficient synthesis of diverse spirotetrahydrocarbazoles

Shao-Cong Zhan, Ren-Jie Fang, Jing Sun and Chao-Guo Yan

Beilstein J. Org. Chem. **2022,** *18,* 796–808. doi:10.3762/bjoc.18.80

Characterization data and ^{1}H NMR, ^{13}C NMR, HRMS spectra of the compounds

License and Terms: This is a supporting information file under the terms of the Creative Commons Attribution License (https://creativecommons.org/licenses/by/4.0). Please note that the reuse, redistribution and reproduction in particular requires that the author(s) and source are credited and that individual graphics may be subject to special legal provisions.
The license is subject to the *Beilstein Journal of Organic Chemistry* terms and conditions: (https://www.beilstein-journals.org/bjoc/terms)

Table of contents

**Figures of the single crystal structures**	**S2–S4**
**Single crystal data**	**S5–S7**
**Experimental section**	**S8–S9**
**^{1}H, ^{13}C{1H} NMR and HRMS spectra of all compounds**	**S10–S123**

Single crystals were grown by slow evaporation of concentrated solution in $CHCl_3$ / DCM / EtOH (compounds **1f, 2b, 2g', 3a, 4e, 5b**) in glass vials, which were then sealed by plugs with needles on them.

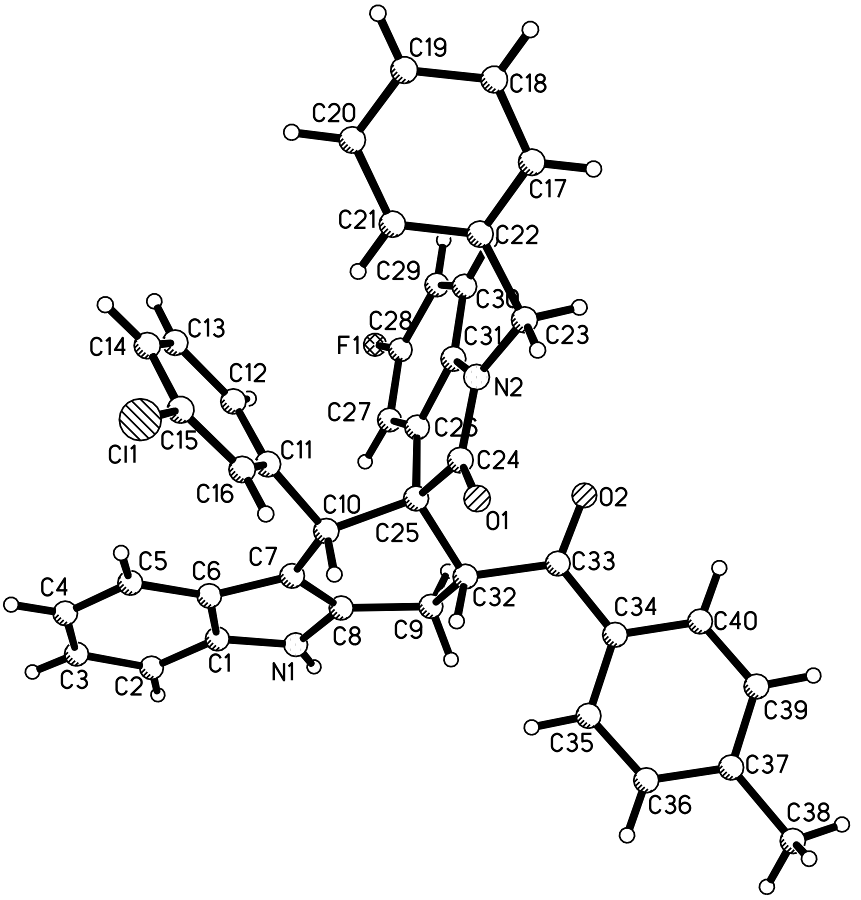

Fig. S1 ORTEP drawing (30%) of the crystal structure of **1f**

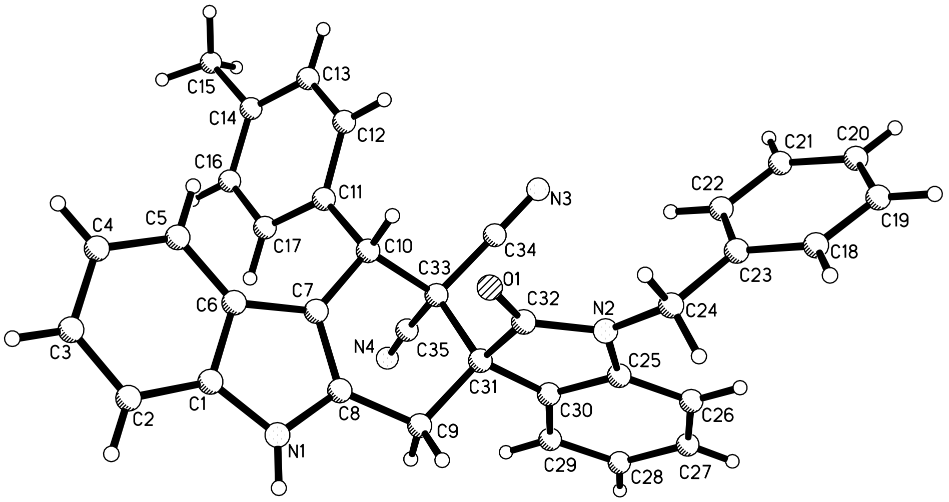

Fig. S2 ORTEP drawing (30%) of the crystal structure of **2b**

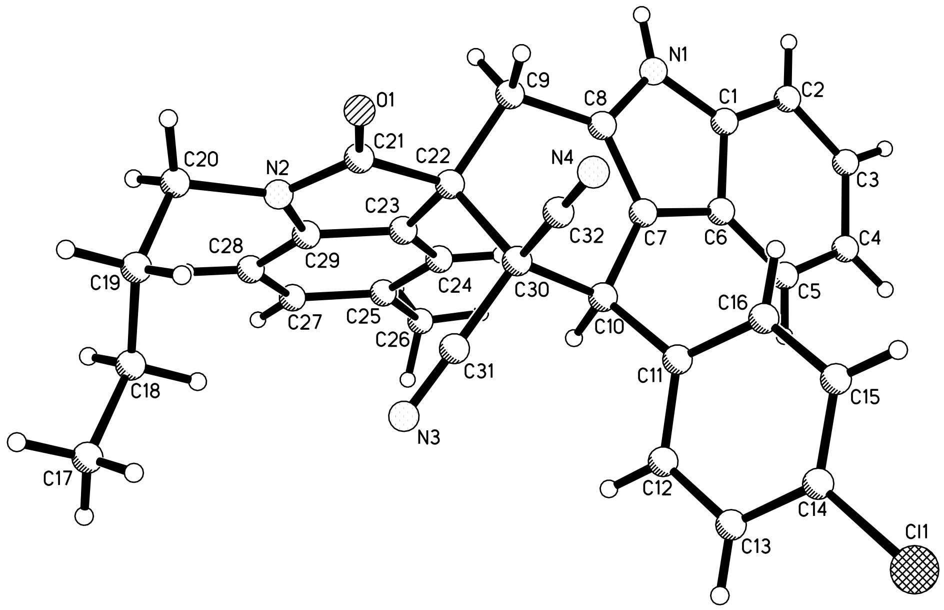

Fig. S3 ORTEP drawing (30%) of the crystal structure of **2g'**

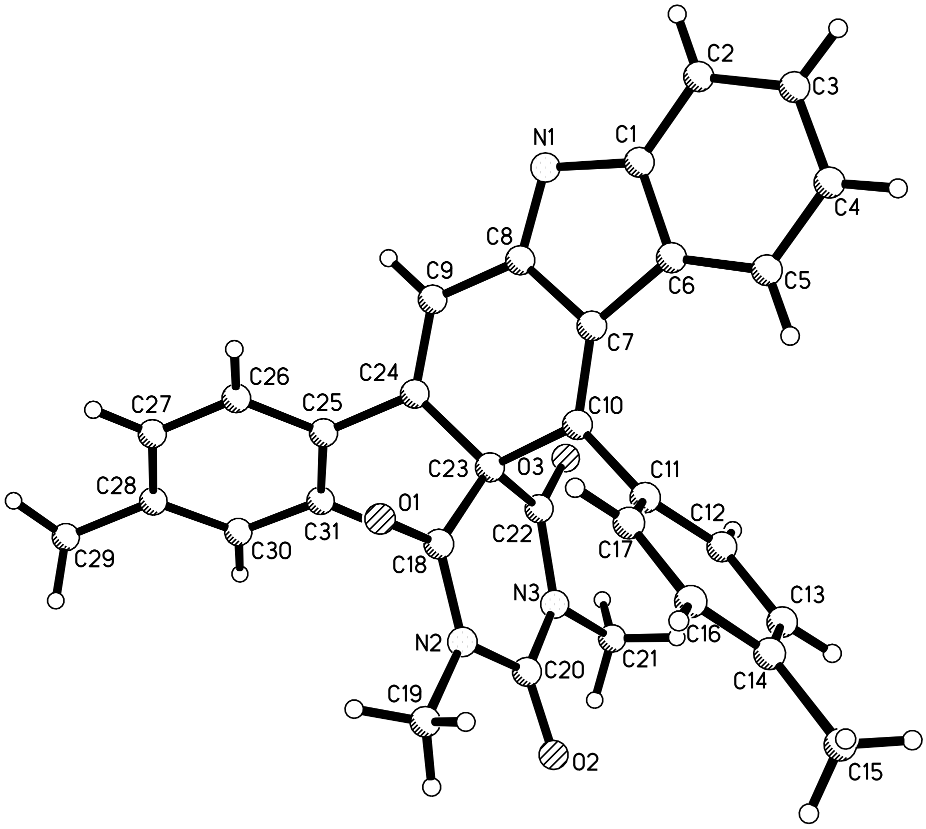

Fig. S4 ORTEP drawing (30%) of the crystal structure of **3a**

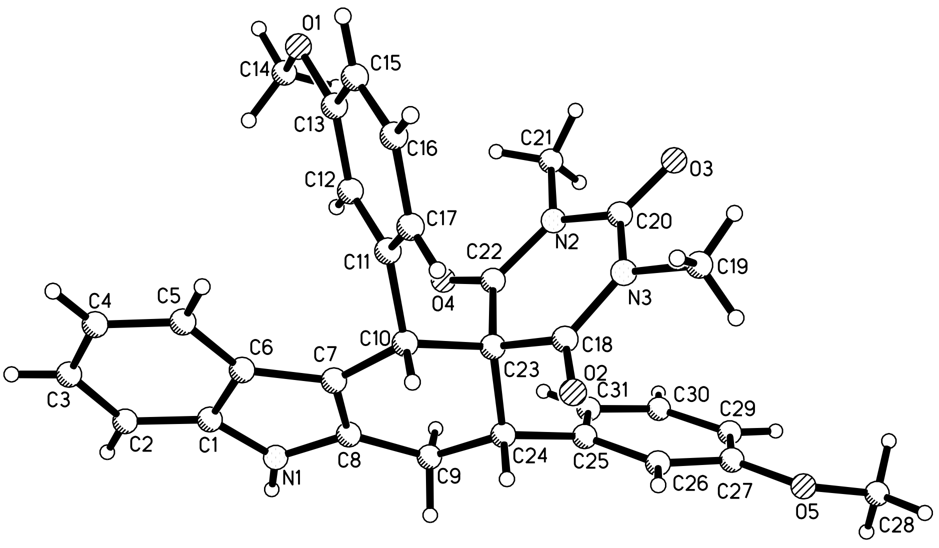

Fig. S5 ORTEP drawing (30%) of the crystal structure of **4e**

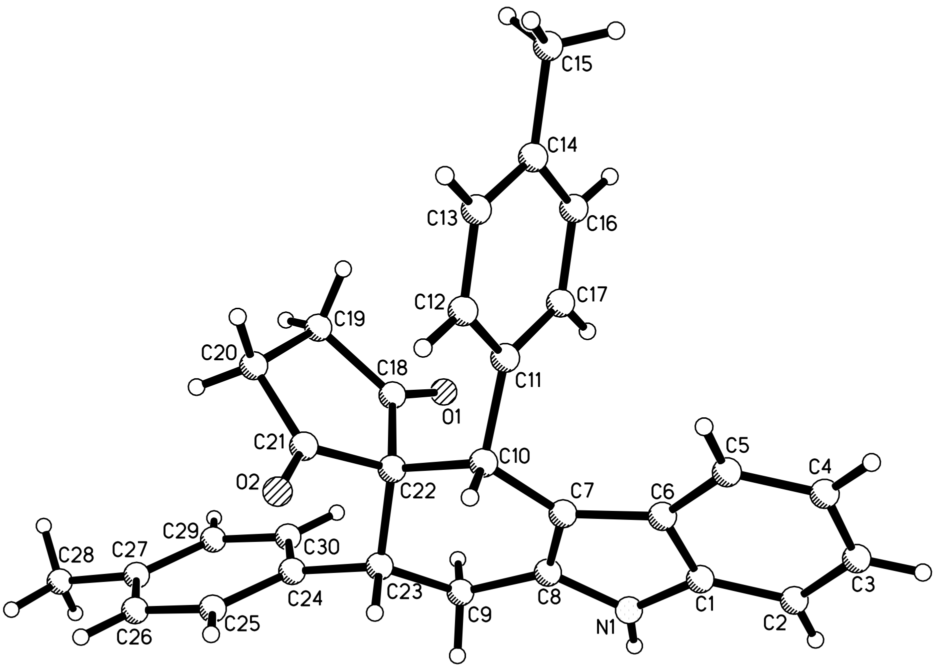

Fig. S6 ORTEP drawing (30%) of the crystal structure of **5b**

Table S1 The single crystal date of compounds **1f, 2b'**

Phase	**1f**	**2b**
Empirical formula	$C_{40}H_{30}ClFN_2O_2$	$C_{35}H_{26}N_4O$
Formula weight	625.11	518.60
Temperature/K	296(2) K	296(2) K
Wavelength/ Å	0.71073	0.71073
Crystal system	Orthorhombic	Monoclinic
Space group	Pbca	P2(1)/n
a /Å	10.6504(5)	17.974(8)
b / Å	20.2099(12)	8.236(4)
c / Å	29.3774(14)	21.725(9)
α (°)	90	90
β (°)	90	109.319(11)
γ (°)	90	90
V (Å ^{3})	6323.3(6)	3035(2)
Z	8	4
Calculated density (g·cm^{-3})	1.313	1.135
Absorption coefficient(mm^{-1})	0.166	0.070
F(000)	2608	1088
θ range /(°)	2.015 to 24.999 deg.	2.401 to 24.995
Limiting indices	- 12<=h<=10, - 21<=k<=24, -34<=l<=34	-21<=h<=21, -9<=k<=9, -25<=l<=23
Reflections collected/unique	49453 / 5558 [R(int) = 0.0996]	20790 / 5259 [R(int) = 0.1344]
Completeness to theta	99.9 %	98.4 %
Max. and min. transmission	0.7456 and 0.6685	0.7456 and 0.6320
Refinement method	Full-matrix least-squares on F^2	Full-matrix least-squares on F^2
Data/restraints/parameters	5558 / 0 / 417	5259 / 0 / 362
Goodness-of-fit on F^2	1.054	1.016
Final *R* indices[I>2sigma(I)]	R1 = 0.0796, wR2 = 0.2216	R1 = 0.0801, wR2 = 0.2005
R indices (all data)	R1 = 0.1537, wR2 = 0.2491	R1 = 0.1978, wR2 = 0.2542
Largest diff. peak and hole /(e ·Å^{-3})	1.473 and -0.443	0.246 and -0.212

Table S2 The single crystal date of compounds **2g’, 3a**

Phase	**2g’**	**3a**
Empirical formula	$C_{33}H_{28}Cl_4N_4O$	$C_{31}H_{25}N_3O_3$
Formula weight	638.39	487.54
Temperature/K	296(2) K	296(2) K
Wavelength/ Å	0.71073	0.71073
Crystal system	Monoclinic	Monoclinic
Space group	P2(1)/c	P2(1)/n
a /Å	10.4591(4)	12.6929(8)
b / Å	12.0856(5)	14.6126(8)
c / Å	24.9411(10)	14.7027(7)
α (°)	90	90
β (°)	93.7861(13)	113.5420(18)
γ (°)	90	90
V ($Å^3$)	3145.8(2)	2500.0(2)
Z	8	4
Calculated density ($g·cm^{-3}$)	1.348	1.295
Absorption coefficient(mm^{-1})	0.409	0.085
F(000)	1320	1024
θ range /(°)	2.349 to 26.000 deg.	2.275 to 25.995
Limiting indices	-12<=h<=12, -12<=k<=14, -30<=l<=30	-15<=h<=13, -18<=k<=17, -18<=l<=17
Reflections collected/unique	29030 / 6165 [R(int) = 0.0353]	23220 / 4903 [R(int) = 0.0554]
Completeness to theta	99.8 %	99.7 %
Max. and min. transmission	0.7456 and 0.6750	0.7456 and 0.6972
Refinement method	Full-matrix least-squares on F^2	Full-matrix least-squares on F^2
Data/restraints/parameters	6165 / 0 / 381	4903 / 0 / 339
Goodness-of-fit on F^2	1.020	1.029
Final *R* indices[I>2sigma(I)]	R1 = 0.0572, wR2 = 0.1346	R1 = 0.0495, wR2 = 0.1057
R indices (all data)	R1 = 0.0882, wR2 = 0.1538	R1 = 0.1077, wR2 = 0.1285
Largest diff. peak and hole /($e·Å^{-3}$)	0.598 and -0.516	0.194 and -0.149

Table S3 The single crystal date of compounds **4e, 5b**

Phase	**4e**	**5b**
Empirical formula	$C_{31}H_{29}N_3O_5$	$C_{31}H_{28}Cl_3NO_2$
Formula weight	523.57	552.89
Temperature/K	296(2) K	296(2) K
Wavelength/ Å	0.71073	0.71073
Crystal system	Monoclinic	Monoclinic
Space group	P2(1)/c	P2(1)/c
a /Å	9.1112(5)	9.0519(14)
b / Å	32.6212(16)	12.354(2)
c / Å	17.7777(10)	25.164(5)
α (°)	90	90
β (°)	91.0593(17)	99.596(5)
γ (°)	90	90
V (Å ^{3})	5283.0(5)	2774.6(8)
Z	8	4
Calculated density (g·cm^{-3})	1.317	1.324
Absorption coefficient(mm^{-1})	0.090	0.359
F(000)	2208	1152
θ range /(°)	2.196 to 25.999	2.327 to 25.998
Limiting indices	-11<=h<=11, -40<=k<=40, -21<=l<=21	-11<=h<=10, -14<=k<=15, -31<=l<=27
Reflections collected/unique	46537 / 10364 [R(int) = 0.0880]	23988 / 5440 [R(int) = 0.1160]
Completeness to theta	99.8 %	99.9 %
Max. and min. transmission	0.7455 and 0.6933	0.7456 and 0.4919
Refinement method	Full-matrix least-squares on F^2	Full-matrix least-squares on F^2
Data/restraints/parameters	10364 / 65 / 711	5440 / 0 / 336
Goodness-of-fit on F^2	1.022	1.020
Final *R* indices[I>2sigma(I)]	R1 = 0.0745, wR2 = 0.1512	R1 = 0.0803, wR2 = 0.1909
R indices (all data)	R1 = 0.1792, wR2 = 0.1920	R1 = 0.1849, wR2 = 0.2480
Largest diff. peak and hole /(e ·Å^{-3})	0.600 and -0.238	0.392 and -0.495

Experimental section

Unless noted, the commercial reagents and solvents were used without further purification. Melting points were recorded with a micromelting point apparatus and are uncorrected. IR spectra were recorded using a Bruker Tensor 27 spectrometer (KBr disc). The ^{1}H and 13C{^{1}H} NMR spectra were recorded with a Varian 400 spectrometer at 400 or 100 MHz. High-resolution mass spectra (HRMS) were recorded in ESI mode using a MicroTOF mass spectrometer. Single-crystal X-ray data were collected with a Bruker Smart APEX-2 CCD diffractometer. All reactions were monitored by thin-layer chromatography (TLC) using silica gel plates (silica gel 60 F254 0.25 mm), and components were monitored by observation under UV light (254 and 365 nm).

1. **General procedure for the synthesis of functionalized spiro[carbazole-3,3'-inolines]:** A mixture of 2-methyl-1*H*-indole (0.5 mmol, 1.0 equiv), aldehyde (0.6 mmol, 1.2 equiv), 3-methyleneoxindole (0.5 mmol, 1.0 equiv) and $CuSO_4$ (0.1 mmol, 0.2 equiv) in dry toluene (6.0 mL) was stirred at 110 ^{o}C for about three hours. After removing the solvent by evaporating at reduced pressure, the residue was subjected to column chromatography with ethyl acetate and light petroleum (v/v = 1:5–1:8) as eluent to give pure **1a–j** and **1a'–j'**.

2. **General procedure for the synthesis of functionalized spiro[carbazole-2,3'-indolines]:** A mixture of 2-methyl-1*H*-indole (0.5 mmol, 1.0 equiv), aldehyde (0.6 mmol, 1.2 equiv), 2-(1-benzyl-2-oxoindolin-3-ylidene)malononitrile (0.5 mmol, 1.0 equiv) and $CuSO_4$ (0.1 mmol, 0.2 equiv) in dry toluene (6.0 mL) was stirred at 110 ^{o}C for about three hours. After removing the solvent by evaporating at reduced pressure, the residue was subjected to column chromatography with ethyl acetate and light petroleum (v/v = 1:5-1:8) as eluent to give pure **2a–g** and **2a'–g'**.

3. **General procedure for the synthesis of functionalized spiro[carbazole-3,5'-pyrimidine]**

reaction: A mixture of 2-methyl-1*H*-indole (0.5 mmol, 1.0 equiv), aldehyde (0.6 mmol, 1.2 equiv), 5-arylidene-1,3-dimethylbaribituric acid (0.5 mmol, 1.0 equiv) and $CuSO_4$ (0.1 mmol, 0.2 equiv) in dry toluene (6.0 mL) was stirred at 110°C for about three hours. After removing the solvent by evaporating at reduced pressure, the mixture of the above obtained product and DDQ (1.0 mmol, 0.227 g, 2.0 equiv) in dry acetonitrile (10.0 mL) was stirred at room temperature for about four hours. After removing the solvent by evaporating at reduced pressure, the residue was subjected to column chromatography with ethyl acetate and light petroleum (v/v = 1:3–1:6) as eluent to give pure products **3a–c**.

4. **General procedures for the synthesis of functionalized spiro[carbazole-3,3'-inolines]**: A mixture of 2-methyl-1*H*-indole (0.5 mmol, 1.0 equiv), aldehyde (1.2 mmol, 1.2 equiv) and 1,3-dimethylbaribituric acid (0.5 mmol, 1.0 equiv)) and $CuSO_4$ (0.1 mmol, 0.2 equiv) in dry toluene (6.0 mL) was stirred at 110°C for about three hours. After removing the solvent by evaporating at reduced pressure, the residue was subjected to column chromatography with ethyl acetate and light petroleum (v/v = 1:5–1:8) as eluent to give pure **4a–h**.

5. **General procedures for the synthesis of functionalized tetrahydrospiro[carbazole-3,1'-cyclopentane]-2',5'-diones and tetrahydrospiro[carbazole-3,1'-cyclohexane]-2',6'-diones:** A mixture of 2-methyl-1*H*-indole (0.5 mmol, 1.0 equiv), aldehyde (1.2 mmol, 2.4 equiv), 1,3-diones (0.5 mmol, 1.0 equiv) and $CuSO_4$ (0.1 mmol, 0.2 equiv) in dry toluene (6.0 mL) was stirred at 110 °C for about three hours. After removing the solvent by evaporating at reduced pressure, the residue was subjected to column chromatography with ethyl acetate and light petroleum (v/v = 1:5–1:8) as eluent to give pure **5a–k**.

2-Benzoyl-1'-benzyl-4-phenyl-1,2,4,9-tetrahydrospiro[carbazole-3,3'-indolin]-2'-one (1a): purple solid, 61%, m.p. 182-185 ºC; ^{1}H NMR (400 MHz, $CDCl_3$) δ: 8.23 (s, 1H, NH), 7.89 (d, J = 7.2 Hz, 1H, ArH), 7.54 (t, J = 7.2 Hz, 1H, ArH), 7.43-7.39 (m, 3H, ArH), 7.30 (d, J = 8.0 Hz, 1H, ArH), 7.23-7.15 (m, 2H, ArH), 7.14-7.08 (m, 5H, ArH), 6.99 (t, J = 7.6 Hz, 1H, ArH), 6.83-6.73 (m, 3H, ArH), 6.63-6.62 (m, 2H, ArH), 6.36 (d, J = 7.6 Hz, 1H, ArH), 6.28 (t, J = 7.2 Hz, 2H, ArH), 5.00 (s, 1H, CH), 4.83 (dd, J_1 = 12.4 Hz, J_2 = 5.2 Hz, 1H, CH), 4.60 (d, J = 16.0 Hz, 1H, CH), 4.46 (d, J = 16.0 Hz, 1H, CH), 3.49 (t, J = 12.4 Hz, 1H, CH), 3.26 (dd, J_1 = 16.8 Hz, J_2 = 5.2 Hz, 1H, CH); ^{13}C NMR (400 MHz, $CDCl_3$) δ: 199.3, 178.0, 143.6, 136.6, 136.5, 136.2, 135.2, 133.3, 132.3, 130.9, 130.0, 128.7, 128.6, 128.4, 128.2, 127.9, 127.7, 127.0, 126.9, 126.7, 126.5, 125.6, 121.8, 121.5, 120.2, 119.1, 110.7, 110.7, 109.0, 56.1, 50.0, 48.8, 43.6, 25.4; IR (KBr) υ: 3367, 3210, 3155, 3017, 2980, 2831, 2864, 1877, 1623, 1611, 1507, 1456, 1355, 1241, 1178, 1143, 955, 931, 849, 789 cm^{-1}; MS (m/z): HRMS (ESI) Calcd. for $C_{39}H_{30}N_2O_2$($[M+Na]^+$): 581.2199, found: 581.2191.

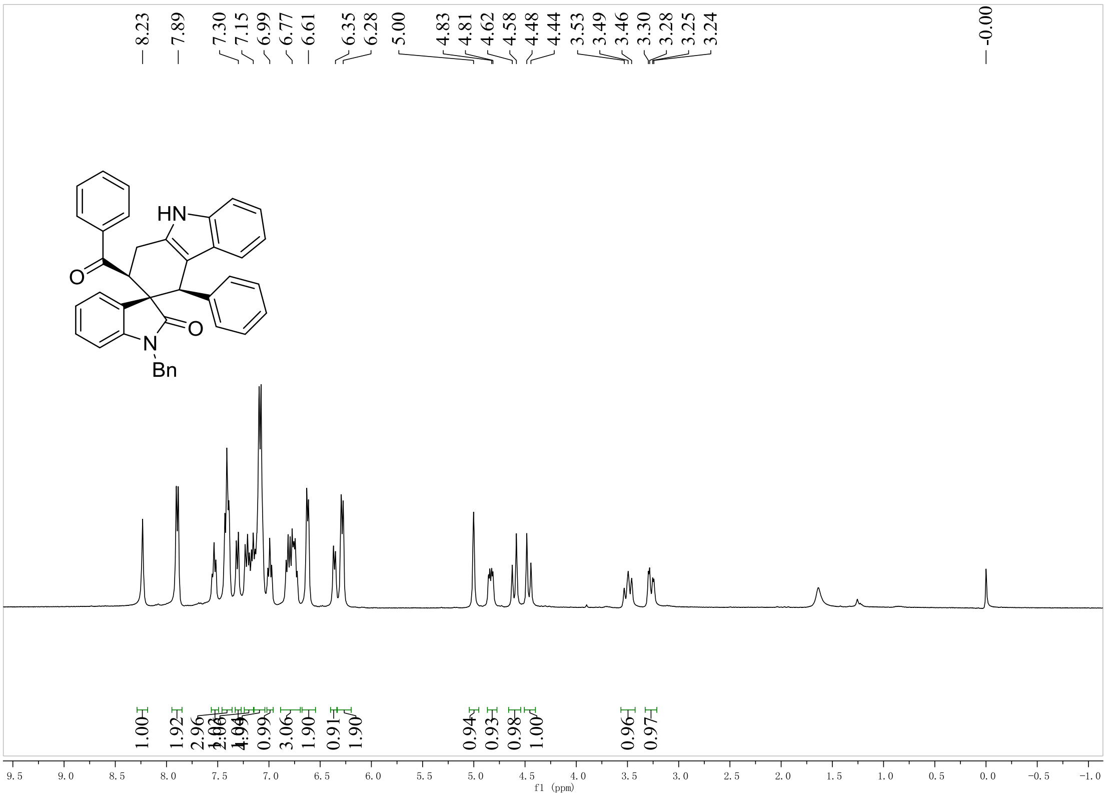

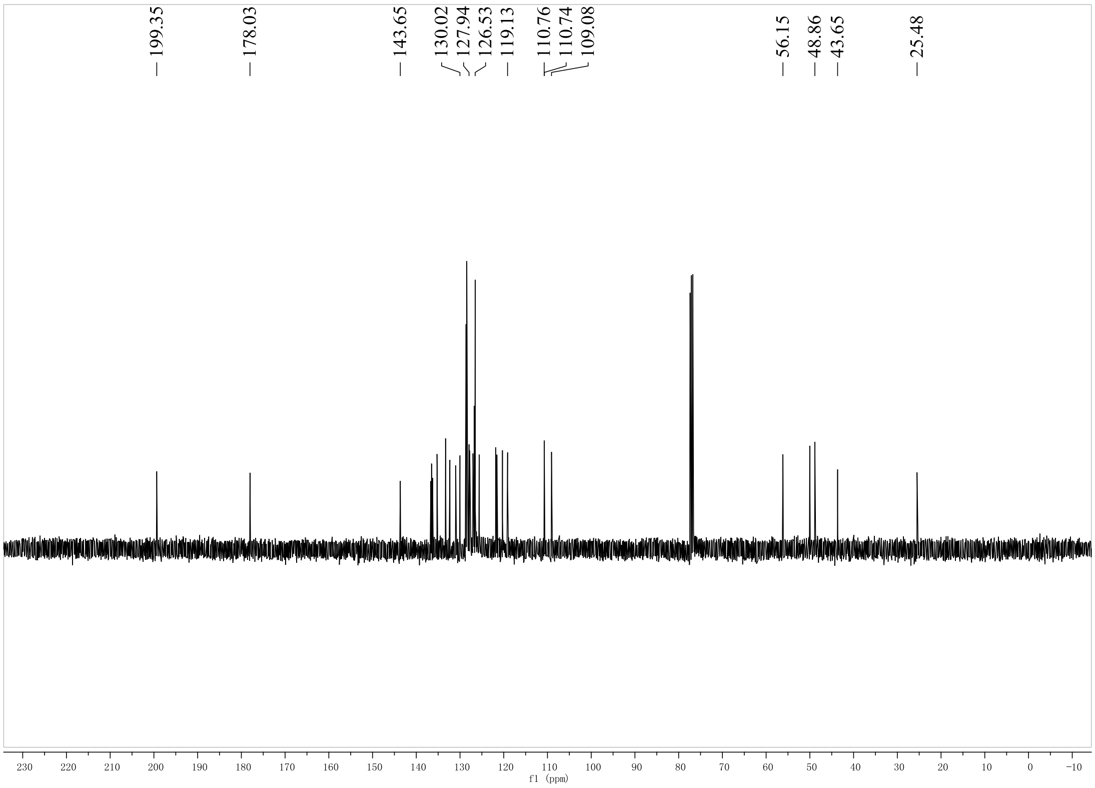
199.35
178.03
143.65
130.02
127.94
126.53
119.13
110.76
110.74
109.08
56.15
48.86
43.65
25.48
230 220 210 200 190 180 170 160 150 140 130 120 110 100 90 80 70 60 50 40 30 20 10 0 −10
f1 (ppm)

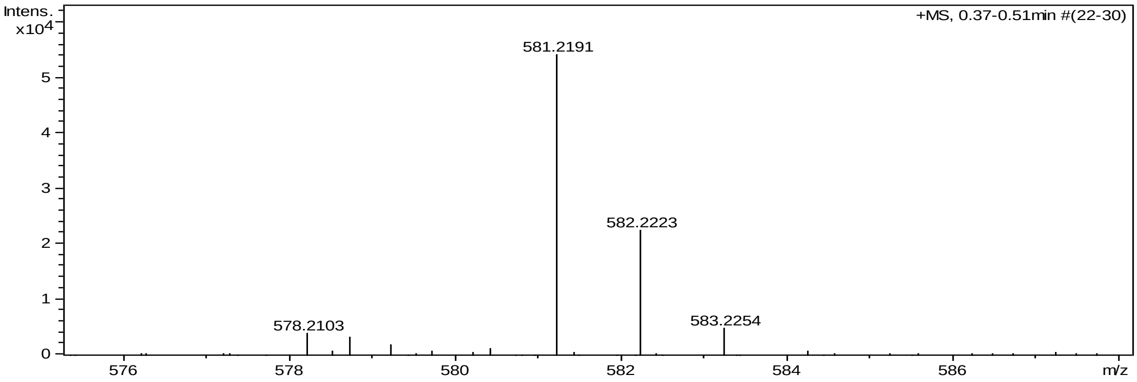
Intens.
x10^4
+MS, 0.37-0.51min #(22-30)
581.2191
582.2223
583.2254
578.2103
576 578 580 582 584 586
m/z

2-Benzoyl-1'-benzyl-4-phenyl-1,2,4,9-tetrahydrospiro[carbazole-3,3'-indolin]-2'-one (1a'): purple solid, 6%, m.p. 195-198 °C; ^{1}H NMR (400 MHz, $CDCl_3$) δ: 8.04 (s, 1H, NH), 7.79 (d, J = 7.6 Hz, 2H, ArH), 7.51 (t, J = 7.2 Hz, 1H, ArH), 7.39 (t, J = 7.2 Hz, 2H, ArH), 7.25-7.19 (m, 5H, ArH), 7.16-7.06 (m, 5H, ArH), 7.04-6.97 (m, 4H, ArH), 6.83 (d, J = 7.2 Hz, 2H, ArH), 6.75-6.73 (m, 1H, ArH), 6.62 (d, J = 7.6 Hz, 1H, ArH), 4.94 (d, J = 15.6 Hz, 1H, CH), 4.84 (s, 1H, CH), 4.56 (d, J = 15.6 Hz, 1H, CH), 4.55-4.53 (m, 1H, CH), 3.81-3.75 (m, 1H, CH), 3.07 (dd, J_1 = 16.8 Hz, J_2 = 7.6 Hz, 1H, CH); ^{13}C NMR (400 MHz, $CDCl_3$) δ: 199.7, 176.5, 142.7, 139.2, 136.6, 136.5, 135.8, 133.1, 132.2, 131.8, 130.1, 128.7, 128.6, 128.5, 128.5, 127.8, 127.7, 127.2, 127.1, 127.0, 126.9, 124.6, 122.2, 121.5, 119.3, 119.1, 110.8, 110.5, 108.9, 54.0, 47.1, 44.2, 43.7, 24.5; IR (KBr) υ: 3354, 3263, 3211, 3130, 3078, 2935, 2864, 2817, 2077, 1867, 1644, 1607, 1537, 1437, 1355, 1256, 1155, 1109, 997, 988, 845, 768 cm^{-1}; MS (m/z): HRMS (ESI) Calcd. for $C_{39}H_{30}N_2O_2$ ($[M+Na]^+$): 581.2199, found: 589.2187.

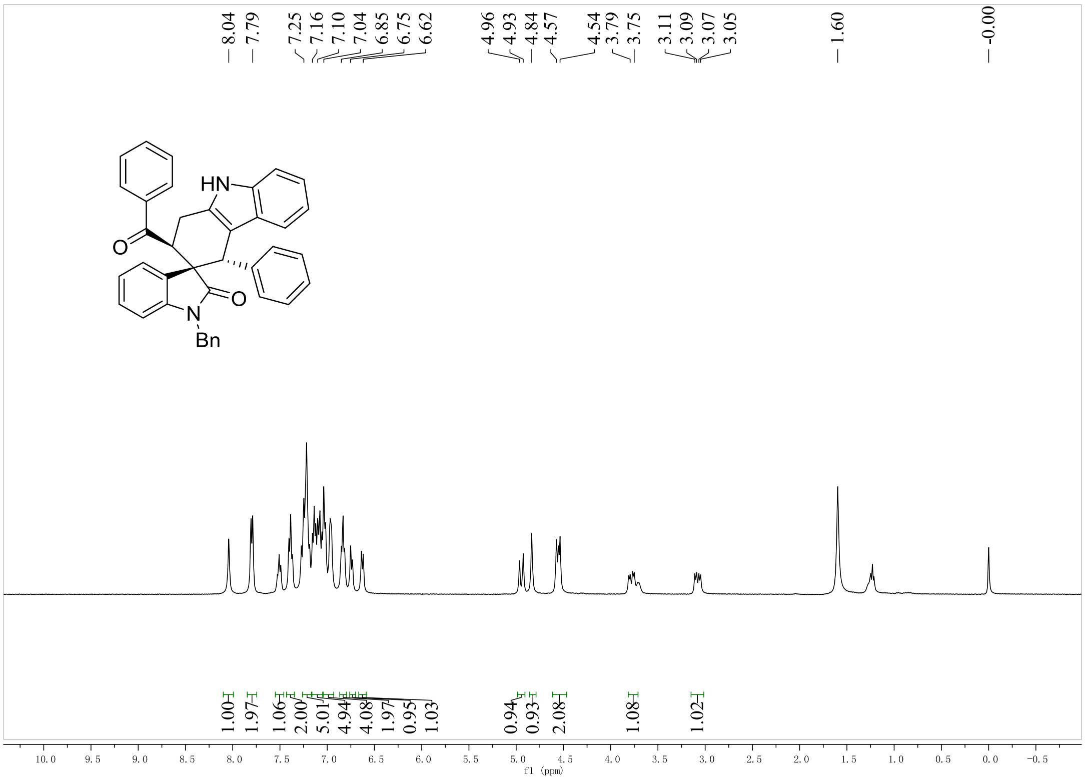

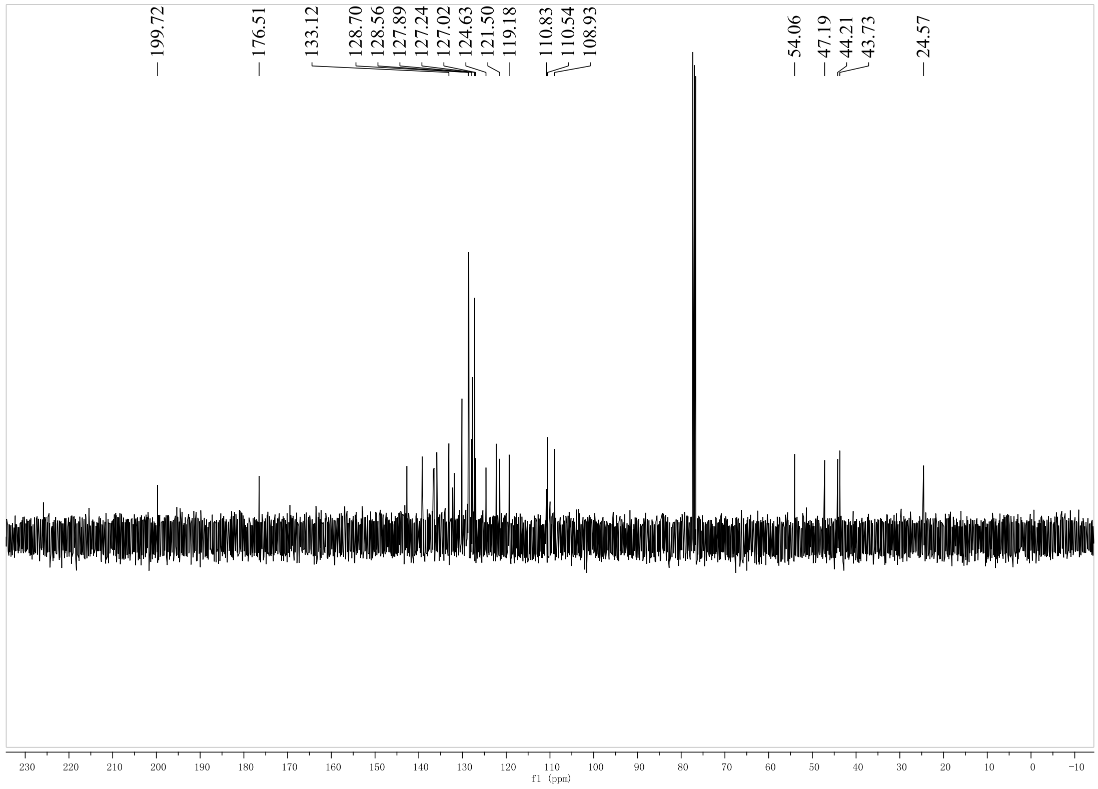

199.72
176.51
133.12
128.70
128.56
127.89
127.24
127.02
124.63
121.50
119.18
110.83
110.54
108.93
54.06
47.19
44.21
43.73
24.57
f1 (ppm)

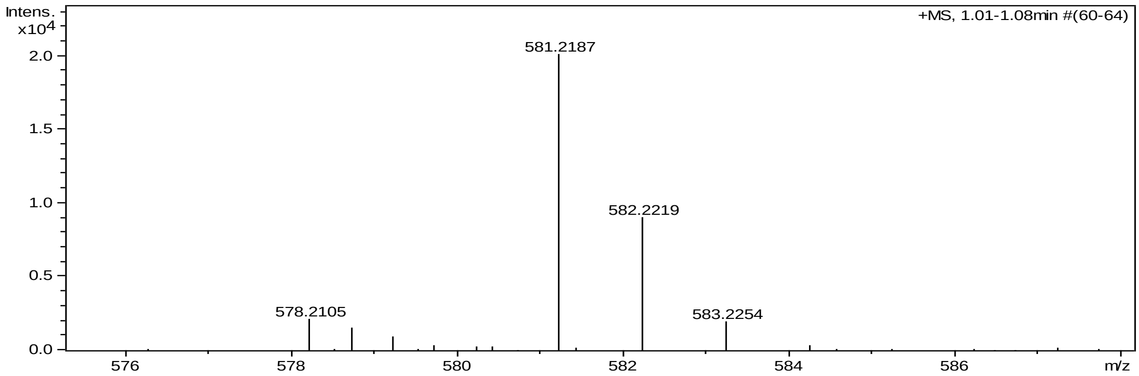

Intens.
x10^4
+MS, 1.01-1.08min #(60-64)
581.2187
582.2219
578.2105
583.2254
m/z

1'-Benzyl-2-(4-chlorobenzoyl)-5'-methyl-4-(*o*-tolyl)-1,2,4,9-tetrahydrospiro[carbazole-3,3'-indolin]-2'-one (1b):

purple solid, 55%, m.p. 190-193 ºC; ^{1}H NMR (400 MHz, $CDCl_3$) δ: 8.08 (s, 1H, NH), 7.87 (d, *J* = 8.4 Hz, 2H, ArH), 7.42 (d, *J* = 8.4 Hz, 2H, ArH), 7.34 (d, *J* = 8.0 Hz, 1H, ArH), 7.17 (d, *J* = 7.2 Hz, 1H, ArH), 7.10-7.04 (m, 5H, ArH), 6.99-6.97 (m, 1H, ArH), 6.87 (d, *J* = 8.0 Hz, 1H, ArH), 6.75 (t, *J* = 7.8 Hz, 1H, ArH), 6.56 (t, *J* = 7.2 Hz, 1H, ArH), 6.44 (d, *J* = 7.2 Hz, 2H, ArH), 6.35 (d, *J* = 8.0 Hz, 1H, ArH), 6.23 (d, *J* = 8.0 Hz, 1H, ArH), 6.05 (d, *J* = 8.0 Hz, 1H, ArH), 5.44 (s, 1H, CH), 4.90-4.84 (m, 2H, CH_2), 4.24 (d, *J* = 16.4 Hz, 1H, CH), 3.67-3.59 (m, 1H, CH), 3.32 (dd, J_1 = 16.8 Hz, J_2 = 5.2 Hz, 1H, CH), 2.54 (s, 3H, CH_3), 2.19 (s, 3H, CH_3); ^{13}C NMR (400 MHz, $CDCl_3$) δ: 198.3, 178.3, 141.5, 139.6, 137.7, 136.6, 135.2, 135.1, 134.5, 131.4, 131.1, 130.7, 130.2, 130.0, 128.8, 128.5, 128.4, 126.8, 126.1, 124.6, 121.6, 119.7, 119.2, 112.2, 110.6, 109.0, 55.4, 49.6, 43.9, 43.5, 25.5, 21.5, 20.4; IR (KBr) υ: 3344, 3271, 3160, 3018, 2955, 2864, 2821, 1874, 1631, 1600, 1567, 1488, 1363, 1231, 1131, 1107, 955, 908, 841, 766 cm^{-1}; MS (*m*/*z*): HRMS (ESI) Calcd. for $C_{41}H_{33}Cl^{35}N_2O_2$ ($[M+Na]^+$): 643.2123, found: 643.2105. $C_{41}H_{33}Cl^{37}N_2O_2$ ($[M+Na]^+$): 645.2123, found: 645.2101.

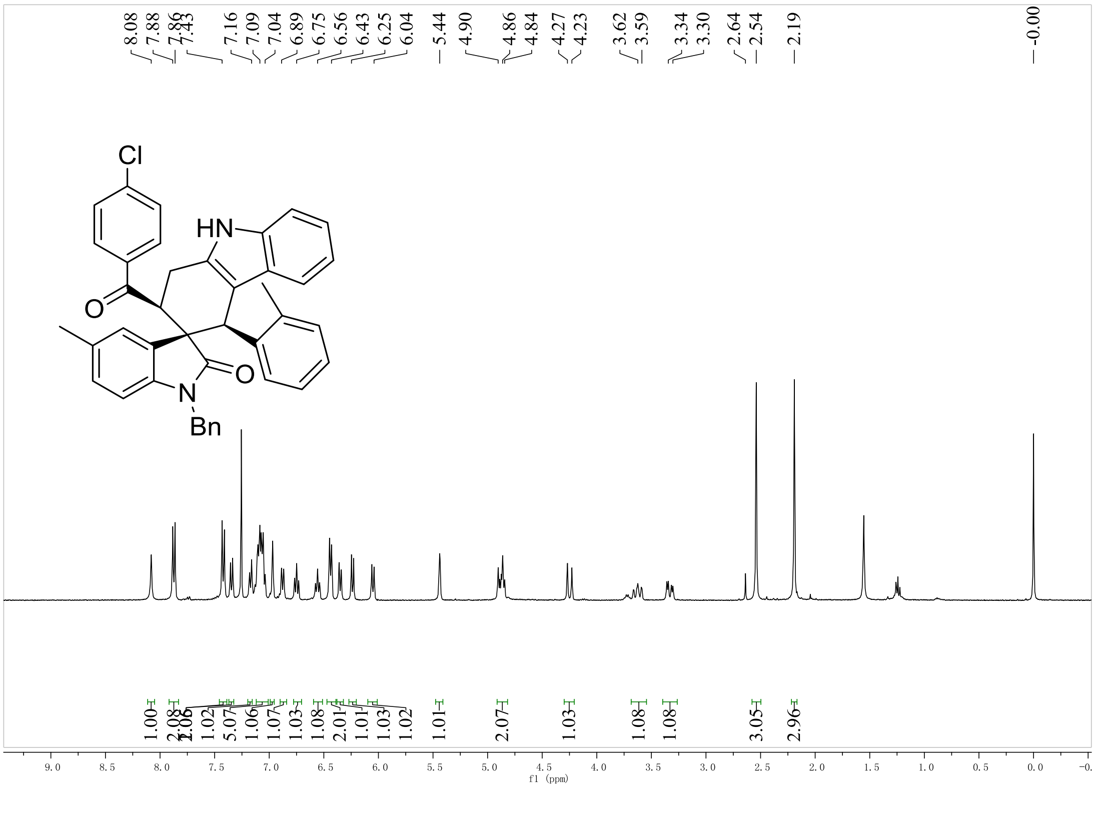

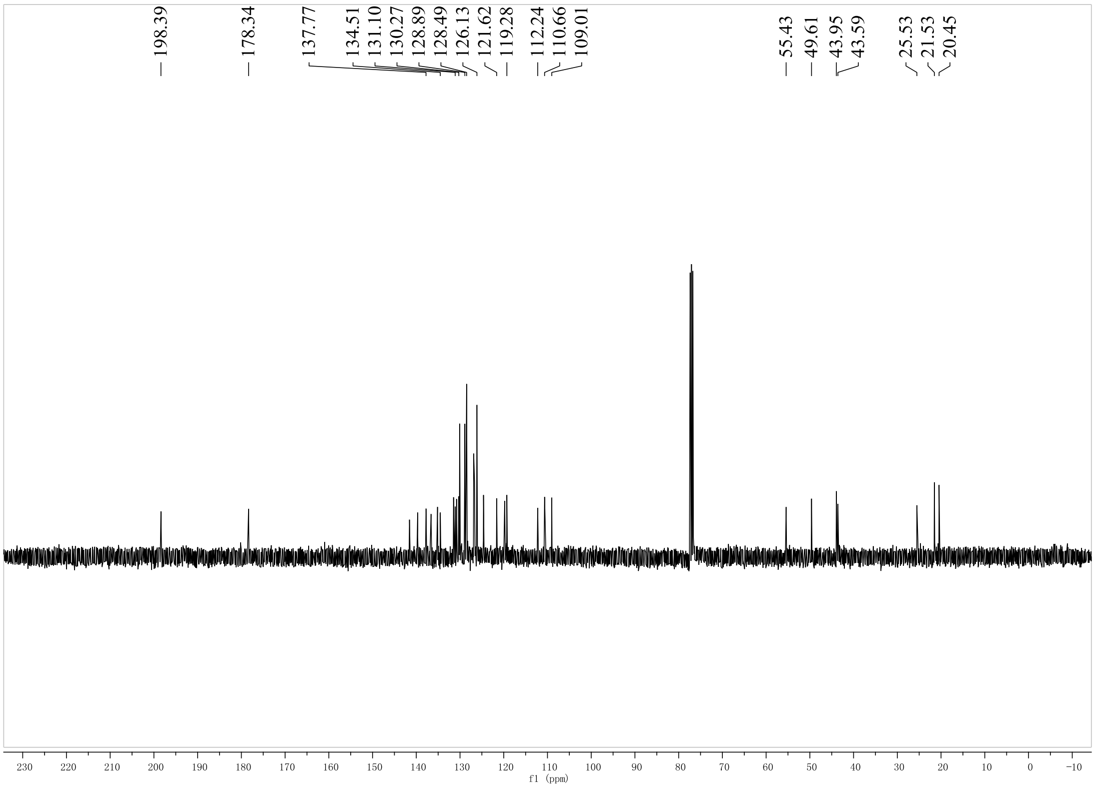
198.39
178.34
137.77
134.51
131.10
130.27
128.89
128.49
126.13
121.62
119.28
112.24
110.66
109.01
55.43
49.61
43.95
43.59
25.53
21.53
20.45
f1 (ppm)

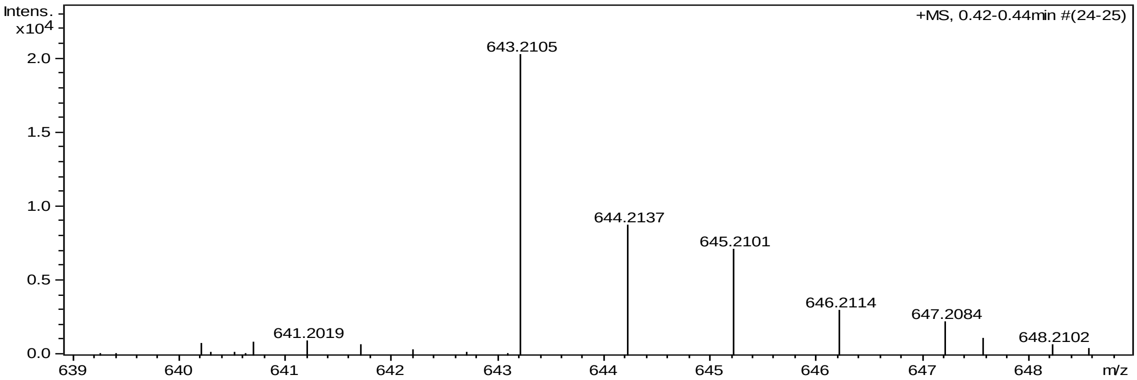
Intens.
x10^4
+MS, 0.42-0.44min #(24-25)
643.2105
644.2137
645.2101
646.2114
647.2084
641.2019
648.2102
m/z

1'-Benzyl-2-(4-chlorobenzoyl)-5'-methyl-4-(*o*-tolyl)-1,2,4,9-tetrahydrospiro[carbazole-3,3'-indolin]-2'-one (1b'):

purple solid, 7%, m.p. 200-203 °C; ^{1}H NMR (400 MHz, $CDCl_3$) δ: 8.01 (s, 1H, NH), 7.75 (d, *J* = 8.8 Hz, 2H, ArH), 7.36-7.32 (m, 3H, ArH), 7.20-7.19 (m, 3H, ArH), 7.14-7.11 (m, 5H, ArH), 7.08-7.05 (m, 2H, ArH), 6.97 (d, *J* = 8.0 Hz, 1H, ArH), 6.88 (t, *J* = 7.2 Hz, 1H, ArH), 6.81 (d, *J* = 8.0 Hz, 1H, ArH), 6.68 (d, *J* = 8.4 Hz, 2H, ArH), 4.84 (d, *J* = 15.6 Hz, 1H, CH), 4.79 (t, *J* = 5.6 Hz, 1H, CH), 4.70 (s, 1H, CH), 4.57 (d, *J* = 15.6 Hz, 1H, CH), 3.41 (dd, J_1 = 16.8 Hz, J_2 = 5.6 Hz, 1H, CH), 3.28 (dd, J_1 = 16.8 Hz, J_2 = 11.6 Hz, 1H, CH), 2.16 (s, 3H, CH_3), 2.12 (s, 3H, CH_3); ^{13}C NMR (400 MHz, $CDCl_3$) δ: 216.4, 215.2, 148.4, 148.0, 140.8, 139.9, 136.3, 136.1, 135.3, 135.0, 134.6, 134.1, 130.0, 129.7, 129.5, 125.4, 124.8, 123.9, 123.1, 123.0, 122.9, 121.8, 121.7, 119.6, 119.5, 119.2, 111.0, 110.9, 109.9, 106.7, 65.1, 48.4, 46.8, 37.2, 37.1, 36.3, 26.3; IR (KBr) υ: 3301, 3267, 3154, 3037, 2955, 2864, 2831, 2051, 1878, 1647, 1633, 1524, 1466, 1371, 1234, 1178, 1168, 944, 907, 862, 755 cm^{-1}; MS (*m*/*z*): HRMS (ESI) Calcd. for $C_{41}H_{33}Cl^{35}N_2O_2$([M+Na]$^+$): 643.2123, found: 643.2106. $C_{41}H_{33}Cl^{37}N_2O_2$([M+Na]$^+$): 644.2156, found: 644.2139.

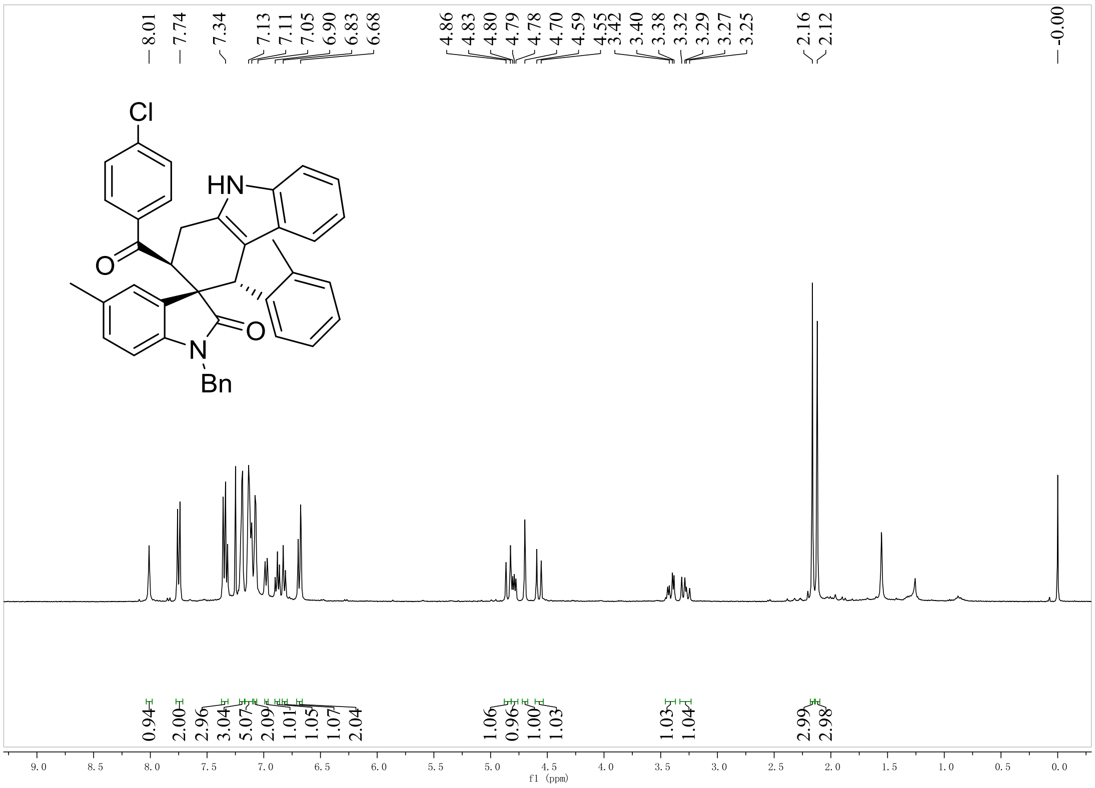

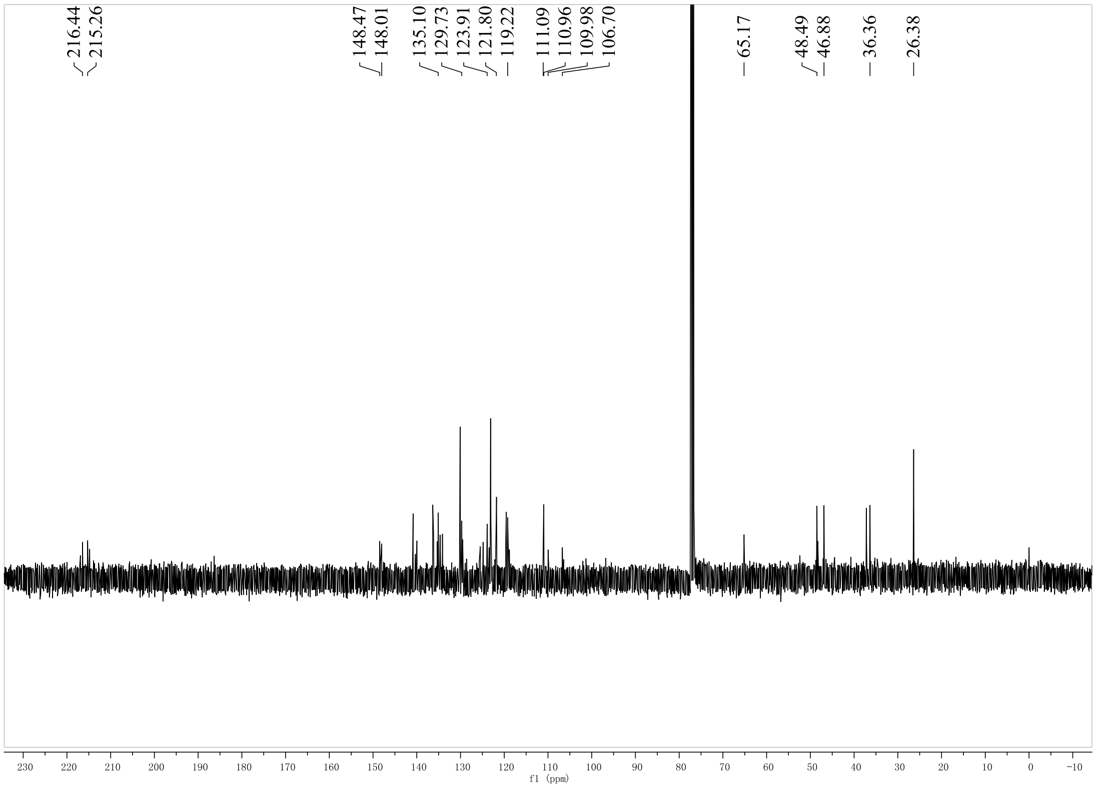
216.44
215.26
148.47
148.01
135.10
129.73
123.91
121.80
119.22
111.09
110.96
109.98
106.70
65.17
48.49
46.88
36.36
26.38
f1 (ppm)

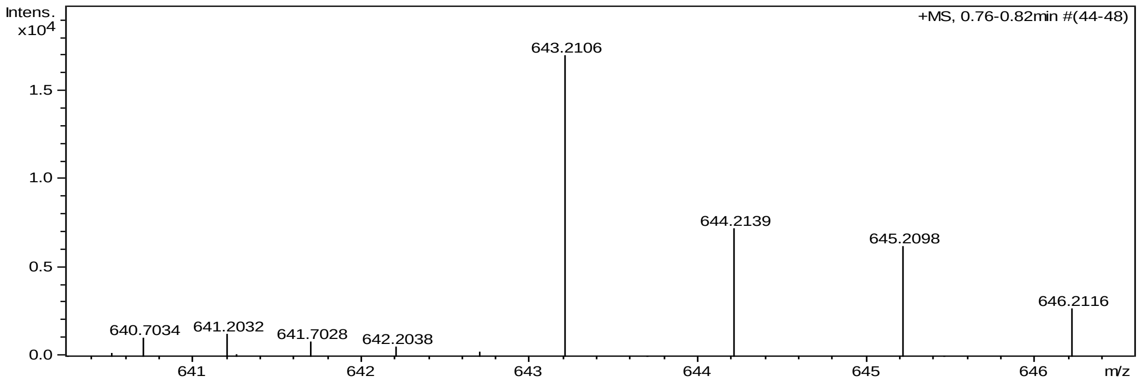
Intens.
x10^4
+MS, 0.76-0.82min #(44-48)
643.2106
644.2139
645.2098
646.2116
640.7034
641.2032
641.7028
642.2038
m/z

1'-Butyl-2-(4-chlorobenzoyl)-4-(4-methoxyphenyl)-5'-methyl-1,2,4,9-tetrahydrospiro[carbazole-3,3'-indolin]-2'-one (1c):

purple solid, 57%, m.p. 193-195 ºC; ^{1}H NMR (400 MHz, $CDCl_3$) δ: 8.07 (s, 1H, NH), 7.86 (d, *J* = 8.4 Hz, 2H, ArH), 7.41-7.36 (m, 3H, ArH), 7.26-7.23 (m, 1H, ArH), 7.11 (t, *J* = 7.2 Hz, 1H, ArH), 6.94-6.90 (m, 2H, ArH), 6.81 (t, *J* = 7.2 Hz, 1H, ArH), 6.69 (d, *J* = 8.4 Hz, 1H, ArH), 6.40 (d, *J* = 7.6 Hz, 2H, ArH), 6.28-6.27 (m, 2H, ArH), 4.90 (s, 1H, CH), 4.72 (dd, J_1 = 12.4 Hz, J_2 = 5.2 Hz, 1H, CH), 3.69 (s, 3H, OCH_3), 3.65-3.57 (m, 1H, CH), 3.26-3.23 (m, 2H, CH_2), 3.18 (d, *J* = 5.2 Hz, 1H, CH), 2.19 (s, 3H, CH_3), 1.18-1.11 (m, 2H, CH_2), 1.01-0.96 (m, 2H, CH_2), 0.76 (t, *J* = 7.2 Hz, 3H, CH_3); ^{13}C NMR (400 MHz, $CDCl_3$) δ: 197.8, 177.1, 158.3, 141.3, 139.6, 136.5, 134.6, 132.0, 131.6, 130.8, 130.6, 130.1, 128.7, 128.5, 128.2, 128.1, 126.8, 126.4, 121.5, 120.4, 119.1, 112.6, 111.9, 111.1, 110.5, 107.7, 56.1, 54.9, 48.8, 47.9, 39.6, 29.2, 25.1, 21.4, 20.0, 13.7; IR(KBr) υ: 3362, 3301, 3256, 3144, 3067, 2963, 2811, 2750, 2143, 1831, 1654, 1611, 1542, 1466, 1355, 1241, 1100, 1054, 966, 931, 849, 772 cm^{-1}; MS (*m/z*): HRMS (ESI) Calcd. for $C_{38}H_{35}Cl^{35}N_2O_3$ ($[M+Na]^+$): 625.2228, found: 625.2228. $C_{38}H_{35}Cl^{37}N_2O_3$ ($[M+Na]^+$): 626.2262, found: 626.2254.

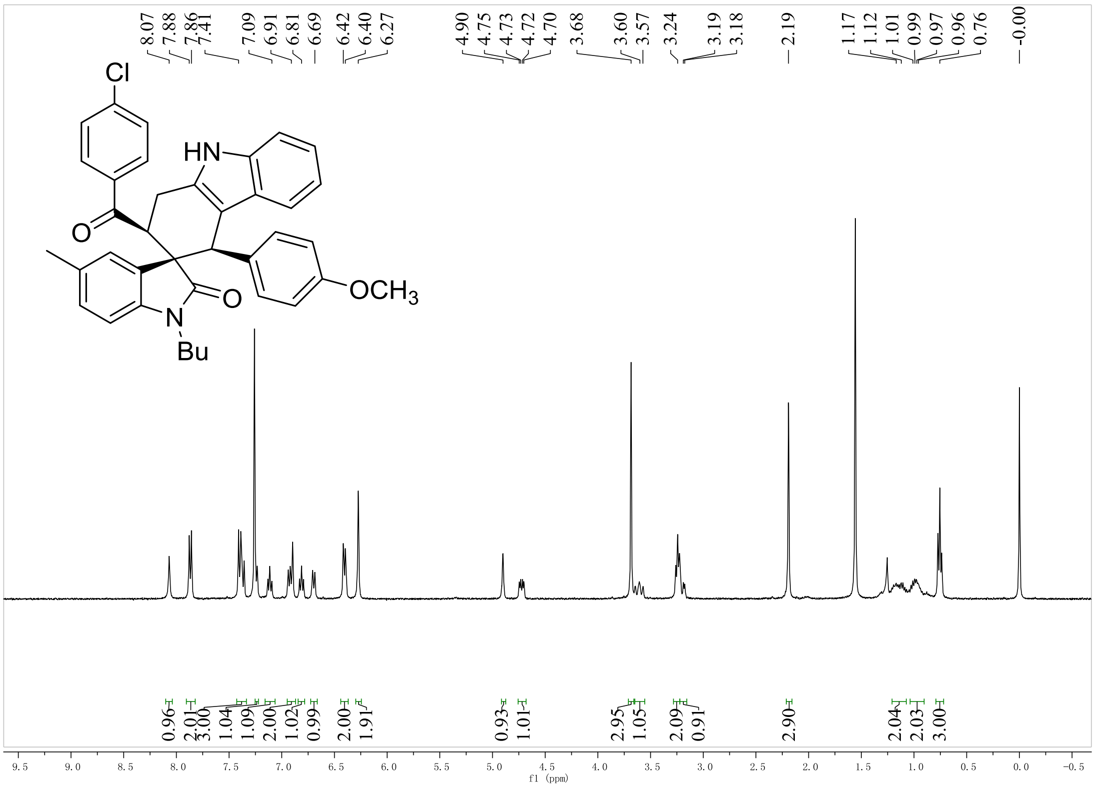

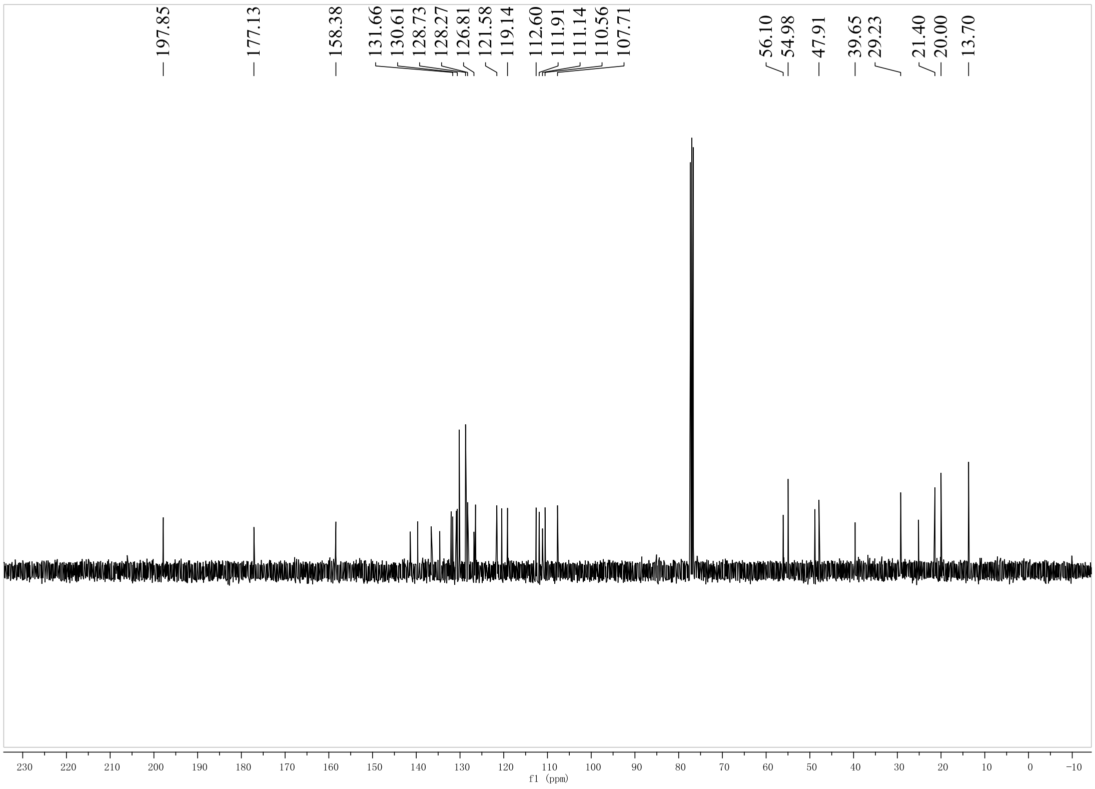

197.85
177.13
158.38
131.66
130.61
128.73
128.27
126.81
121.58
119.14
112.60
111.91
111.14
110.56
107.71
56.10
54.98
47.91
39.65
29.23
21.40
20.00
13.70
230 220 210 200 190 180 170 160 150 140 130 120 110 100 90 80 70 60 50 40 30 20 10 0 −10
f1 (ppm)

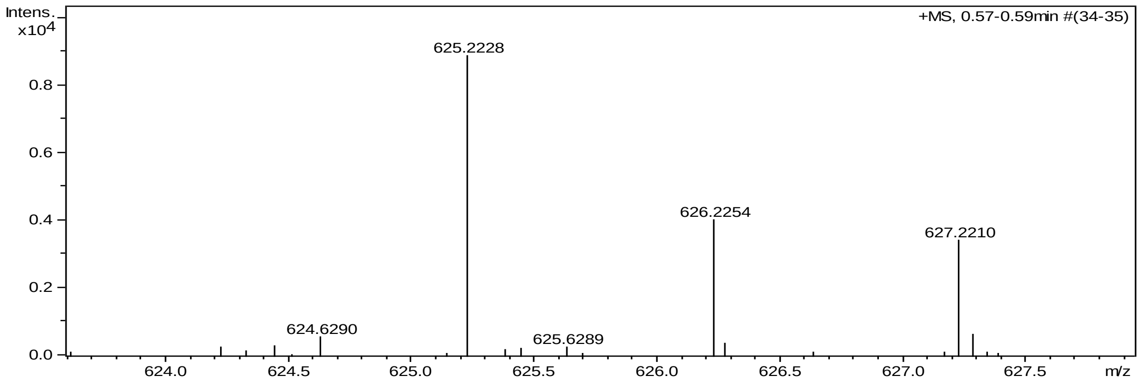

Intens.
x10^4
+MS, 0.57-0.59min #(34-35)
625.2228
626.2254
627.2210
624.6290
625.6289
0.0
0.2
0.4
0.6
0.8
624.0 624.5 625.0 625.5 626.0 626.5 627.0 627.5 m/z

1'-Benzyl-2-(4-chlorobenzoyl)-4-(2-chlorophenyl)-5'-methyl-1,2,4,9-tetrahydrospiro[carbazole-3,3'-indolin]-2'-one (1d):

purple solid, 58%, m.p. 184-187 ºC; ^{1}H NMR (400 MHz, $CDCl_3$) δ: 8.08 (s, 1H, NH), 7.73 (d, *J* = 8.4 Hz, 2H, ArH), 7.37 (d, *J* = 7.2 Hz, 2H, ArH), 7.35-7.32 (m, 2H, ArH), 7.18-7.16 (m, 4H, ArH), 7.14-7.11 (m, 5H, ArH), 6.96 (d, *J* = 8.0 Hz, 1H, ArH), 6.92 (d, *J* = 4.0 Hz, 2H, ArH), 6.64 (d, *J* = 8.0 Hz, 2H, ArH), 4.96 (s, 1H, CH), 4.94 (d, *J* = 15.6 Hz, 1H, CH), 4.71 (t, *J* = 8.4 Hz, 1H, CH), 4.58 (d, *J* = 16.0 Hz, 1H, CH), 3.34 (d, *J* = 8.4 Hz, 2H, CH_2), 2.11 (s, 3H, CH_3); ^{13}C NMR (400 MHz, $CDCl_3$) δ: 198.3, 175.5, 140.3, 139.7, 137.3, 136.8, 135.8, 135.2, 134.7, 132.4, 131.8, 131.5, 130.9, 130.1, 128.9, 128.8, 128.4, 128.3, 128.3, 127.4, 127.1, 126.7, 126.5, 124.6, 122.1, 119.7, 118.7, 110.6, 109.9, 108.8, 53.0, 44.1, 43.3, 42.9, 21.4, 0.1 cm^{-1}; IR (KBr) υ: 3341, 3305, 3248, 3103, 3054, 2954, 2866, 2760, 2149, 1860, 1653, 1667, 1553, 1470, 1344, 1249, 1150, 1140, 967, 933, 849, 761 cm^{-1}; MS (*m/z*): HRMS (ESI) Calcd. for $C_{40}H_{30}Cl_2{}^{35}N_2O_2$ ([M+Na]$^+$): 663.1577, found: 663.1564. $C_{40}H_{30}Cl_2{}^{37}N_2O_2$ ([M+Na]$^+$): 665.1547, found: 665.1545.

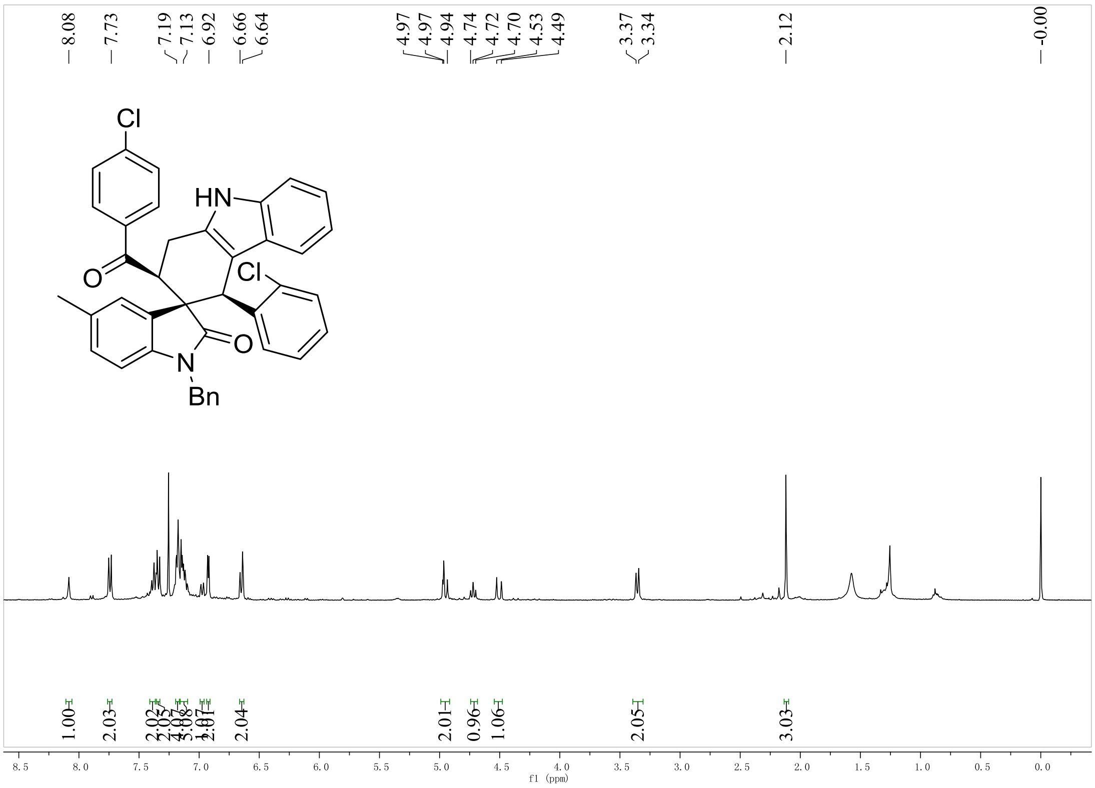

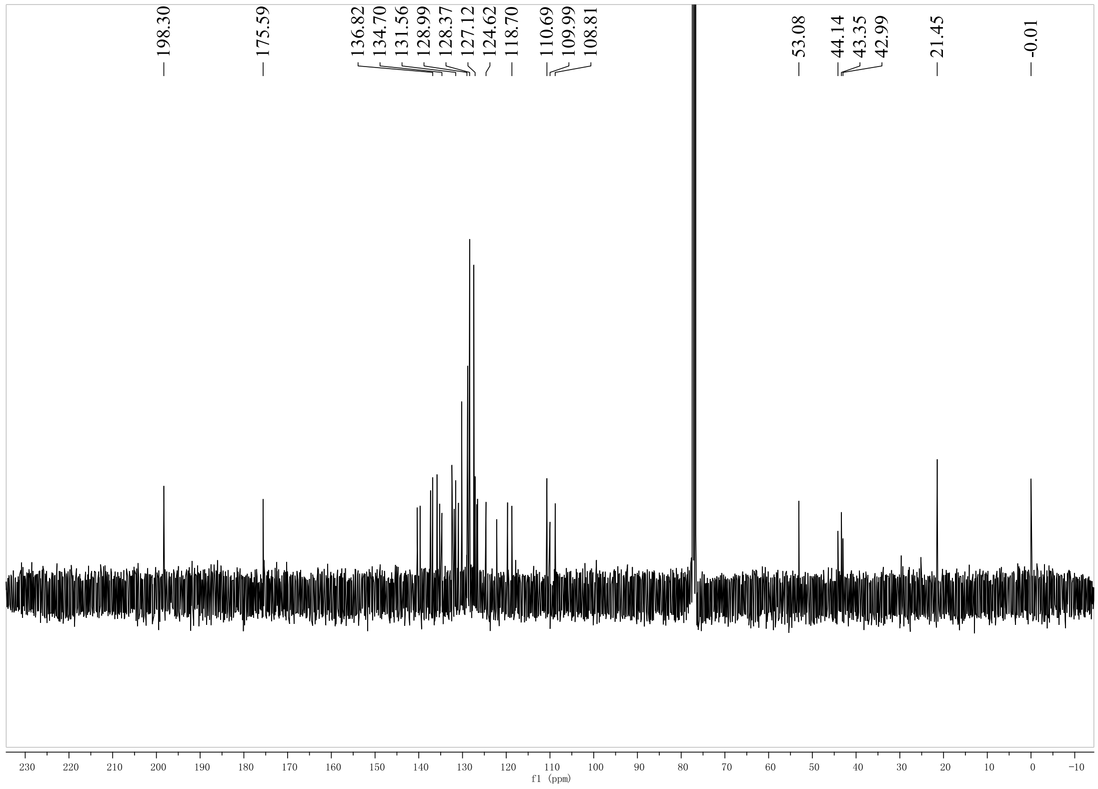
198.30
175.59
136.82
134.70
131.56
128.99
128.37
127.12
124.62
118.70
110.69
109.99
108.81
53.08
44.14
43.35
42.99
21.45
-0.01
f1 (ppm)

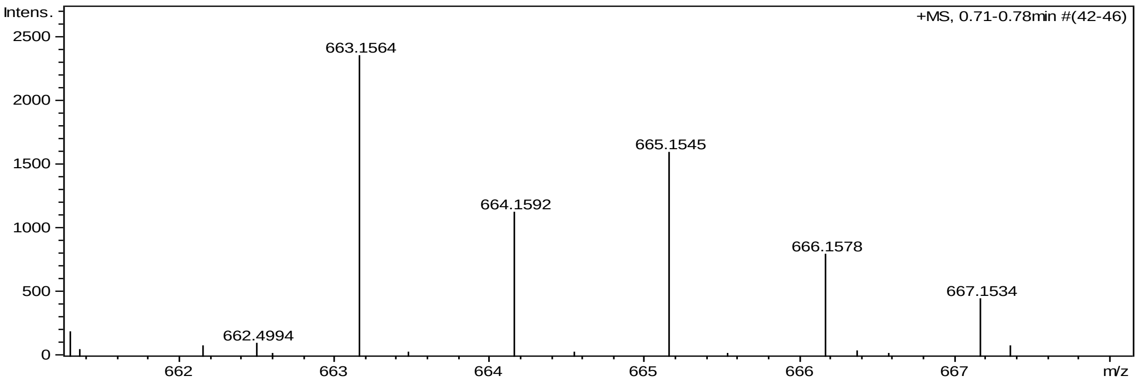
Intens.
+MS, 0.71-0.78min #(42-46)
663.1564
665.1545
664.1592
666.1578
667.1534
662.4994
m/z

1'-Butyl-2-(4-chlorobenzoyl)-4-(4-chlorophenyl)-5'-methyl-1,2,4,9-tetrahydrospiro[carbazole-3,3'-indolin]-2'-one (1e):

purple solid, 65%, m.p. 185-188 ºC; ^{1}H NMR (400 MHz, $CDCl_3$) δ: 8.12 (s, 1H, NH), 7.85 (d, *J* = 8.4 Hz, 2H, ArH), 7.39 (t, *J* = 8.4 Hz, 3H, ArH), 7.29-7.28 (m, 1H, ArH), 7.17-7.12 (m, 2H, ArH), 6.94 (d, *J* = 7.6 Hz, 1H, ArH), 6.88-6.87 (m, 1H, ArH), 6.84 (t, *J* = 7.6 Hz, 1H, ArH), 6.70 (dd, J_1 = 8.4 Hz, J_2 = 2.0 Hz, 1H, ArH), 6.42 (d, *J* = 8.0 Hz, 1H, ArH), 6.37 (d, *J* = 8.0 Hz, 1H, ArH), 6.32 (dd, J_1 = 8.4 Hz, J_2 = 2.0 Hz, 1H, ArH), 4.93 (s, 1H, CH), 4.71 (dd, J_1 = 12.4 Hz, J_2 = 5.2 Hz, 1H, ArH), 3.64-3.56 (m, 1H, CH), 3.26 (q, *J* = 7.2 Hz, 2H, CH_2), 3.23-3.19 (m, 1H, CH), 2.19 (s, 3H, CH_3), 1.16-1.10 (m, 2H, CH_2), 1.02-0.89 (m, 2H, CH_2), 0.78 (t, *J* = 7.2 Hz, 3H, CH_3); ^{13}C NMR (400 MHz, $CDCl_3$) δ: 197.6, 176.8, 141.2, 139.8, 136.5, 135.0, 134.5, 132.6, 132.3, 131.9, 131.1, 130.8, 130.1, 128.7, 128.5, 127.6, 127.4, 126.7, 126.4, 121.7, 120.1, 119.3, 110.6, 107.8, 55.9, 48.8, 47.8, 39.7, 29.2, 25.0, 21.3, 20.0, 13.6; IR (KBr) υ: 3307, 3278, 3148, 3033, 2971, 2863, 2801, 2133, 1862, 1655, 1638, 1564, 1467, 1358, 1231, 1146, 1100, 948, 903, 843, 788 cm^{-1}; MS (*m/z*): HRMS (ESI) Calcd. for $C_{37}H_{32}Cl_2{}^{35}N_2O_2$ ($[M+Na]^+$): 629.1733, found: 629.1733. $C_{37}H_{32}Cl_2{}^{37}N_2O_2$ ($[M+Na]^+$): 631.1704, found: 631.1701.

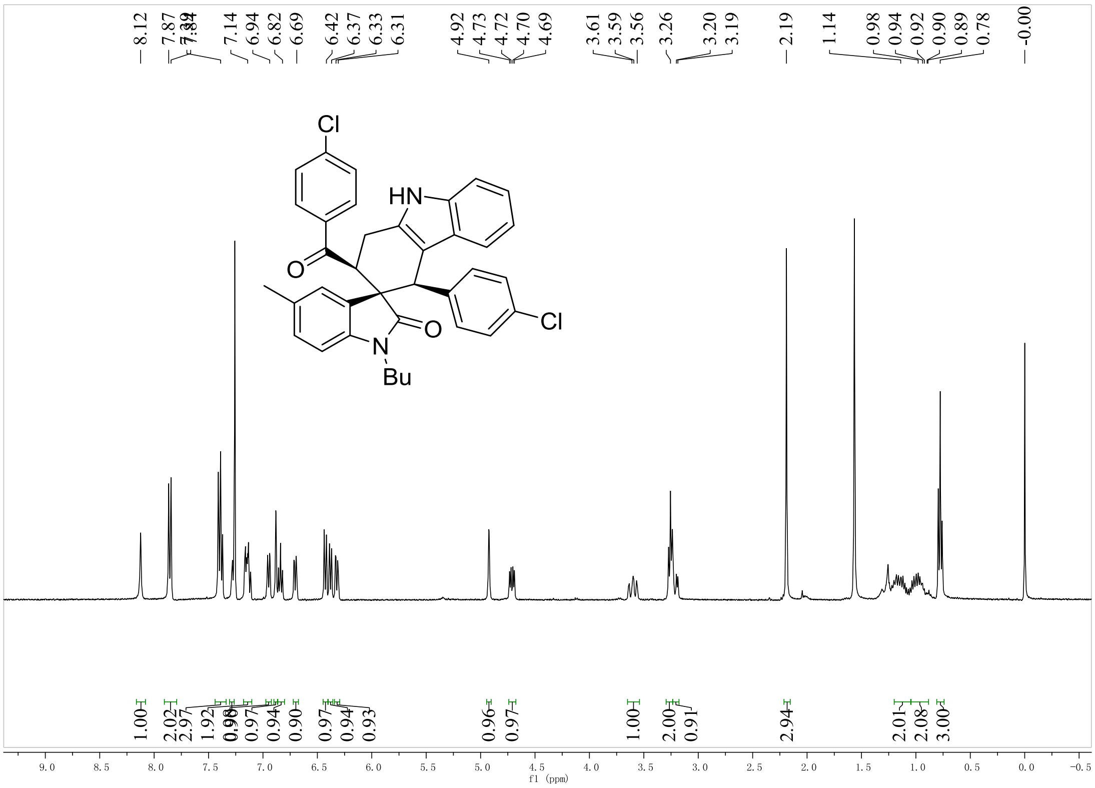

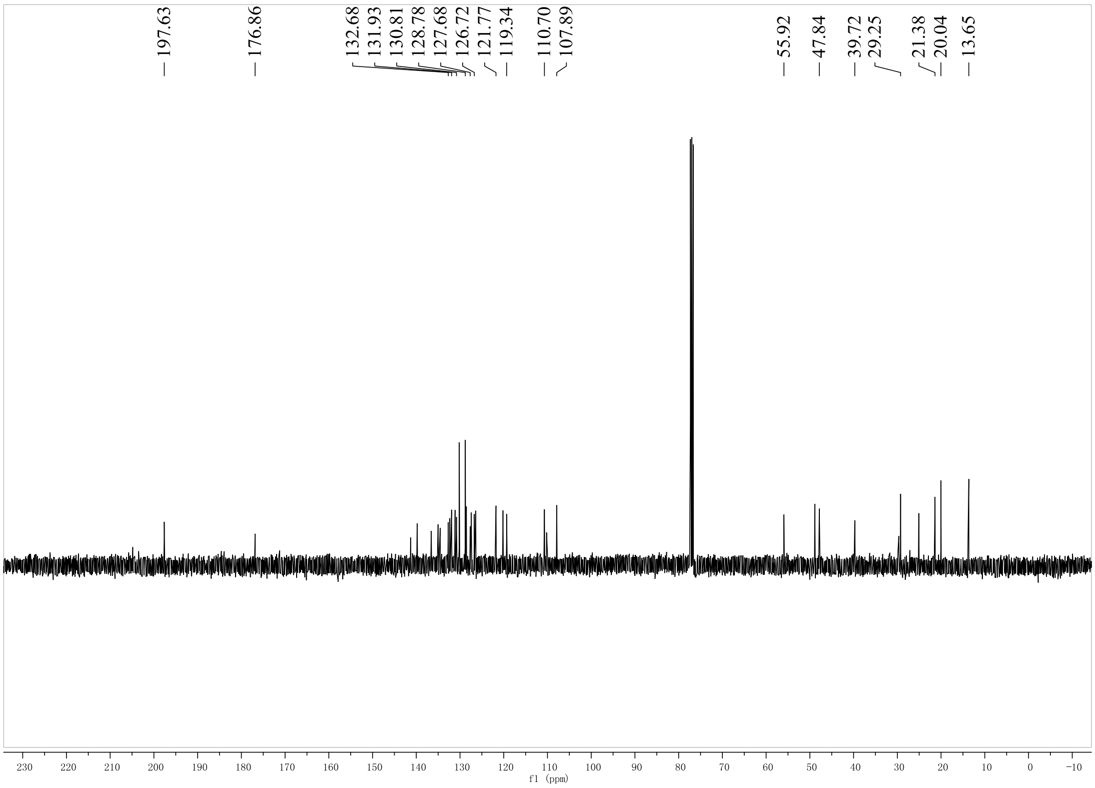

197.63
176.86
132.68
131.93
130.81
128.78
127.68
126.72
121.77
119.34
110.70
107.89
55.92
47.84
39.72
29.25
21.38
20.04
13.65
230 220 210 200 190 180 170 160 150 140 130 120 110 100 90 80 70 60 50 40 30 20 10 0 −10
f1 (ppm)

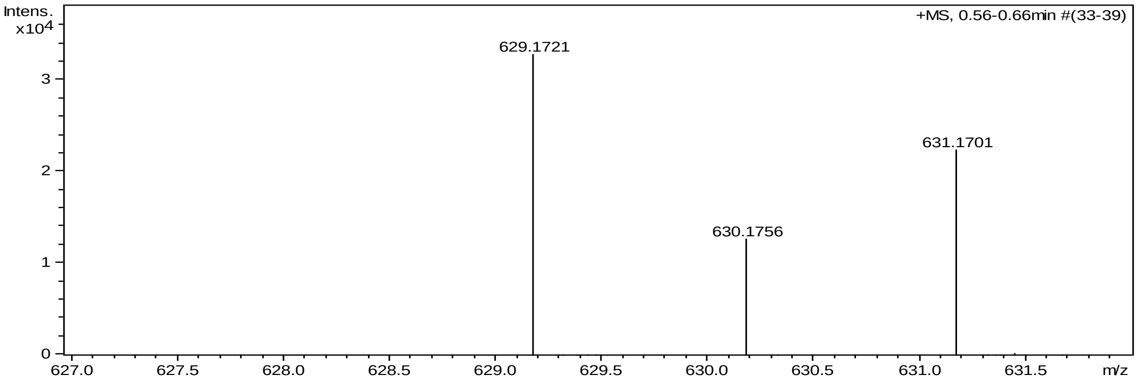

Intens.
x10^4
+MS, 0.56-0.66min #(33-39)
629.1721
631.1701
630.1756
3
2
1
0
627.0 627.5 628.0 628.5 629.0 629.5 630.0 630.5 631.0 631.5 m/z

1'-Benzyl-4-(3-chlorophenyl)-5'-fluoro-2-(4-methylbenzoyl)-1,2,4,9-tetrahydrospiro[carbazole-3,3'-indolin]-2'-one (1f):

purple solid, 55%, m.p. 195-198 ºC; ^{1}H NMR (400 MHz, $CDCl_3$) δ: 8.17 (s, 1H, NH), 7.83 (d, J = 8.0 Hz, 2H, ArH), 7.45-7.35 (m, 2H, ArH), 7.26-7.24 (m, 3H, ArH), 7.17-7.11 (m, 5H, ArH), 6.86-6.81 (m, 2H, ArH), 6.77-6.73 (m, 2H, ArH), 6.71-6.67 (m, 1H, ArH), 6.38 (d, J = 7.2 Hz, 1H, ArH), 6.34 (d, J = 7.6 Hz, 1H, ArH), 6.28-6.20 (m, 1H, ArH), 4.98 (d, J = 7.2 Hz, 1H, CH), 4.82 (dd, J_1 = 12.0 Hz, J_2 = 5.6 Hz, 1H, CH), 4.61 (t, J = 16.0 Hz, 1H, CH), 4.51 (t, J = 16.0 Hz, 1H, CH), 3.47 (t, J = 16.0 Hz, 1H, CH), 3.37-3.30 (m, 1H, CH), 2.41 (s, 3H, CH_3); ^{13}C NMR (400 MHz, $CDCl_3$) δ: 198.5, 177.6, 159.7 (d, J = 11.0 Hz), 157.3 (d, J = 10.9 Hz), 144.5 (d, J = 2.6 Hz), 139.7 (d, J = 11.7 Hz), 138.4 (d, J = 16.2 Hz), 136.6, 134.9, 133.3, 133.2, 133.1, 132.2 (d, J = 11.8 Hz), 130.6, 129.7, 129.4, 128.9, 128.8 (d, J = 10.2 Hz), 128.5, 127.4 (d, J = 9.8 Hz), 127.2, 126.6, 126.4 (d, J = 9.2 Hz), 121.9 (d, J = 22.0 Hz), 119.9 (d, J = 21.8 Hz), 119.3 (d, J = 21.8 Hz), 114.3 (d, J = 11.5 Hz), 114.1 (d, J = 11.2 Hz), 113.7 (d, J = 8.3 Hz), 113.4 (d, J = 7.8 Hz), 110.9, 109.6 (d, J = 5.9 Hz), 109.5 (d, J = 10.6 Hz), 56.3, 49.4, 48.6, 43.9, 25.3, 21.7; IR (KBr) υ: 3299, 3217, 3166, 3031, 2973, 2855, 2814, 2168, 1859, 1643, 1600, 1578, 1463, 1354, 1280, 1167, 1139, 983, 955, 883, 755 cm^{-1}; MS (m/z): HRMS (ESI) Calcd. for $C_{40}H_{30}Cl^{35}FN_2O_2$ ($[M+Na]^+$): 647.1872, found: 647.1860. $C_{40}H_{30}Cl^{37}FN_2O_2$ ($[M+Na]^+$): 648.1906, found: 648.1891.

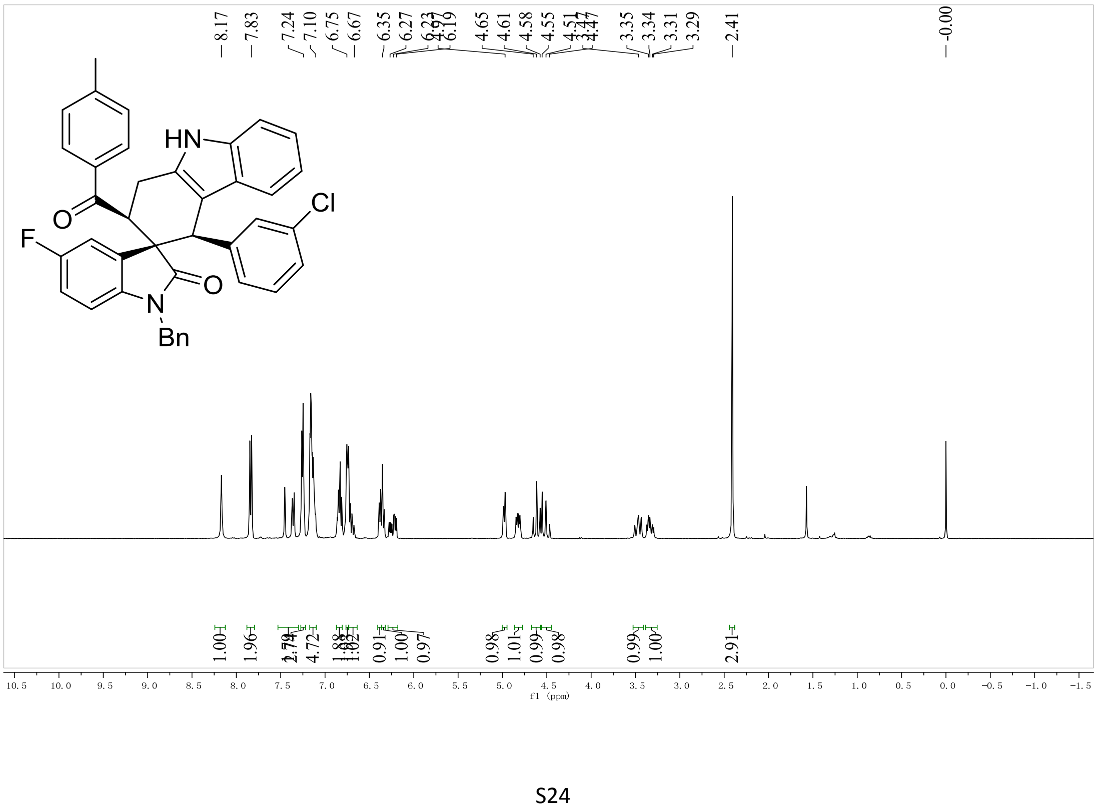

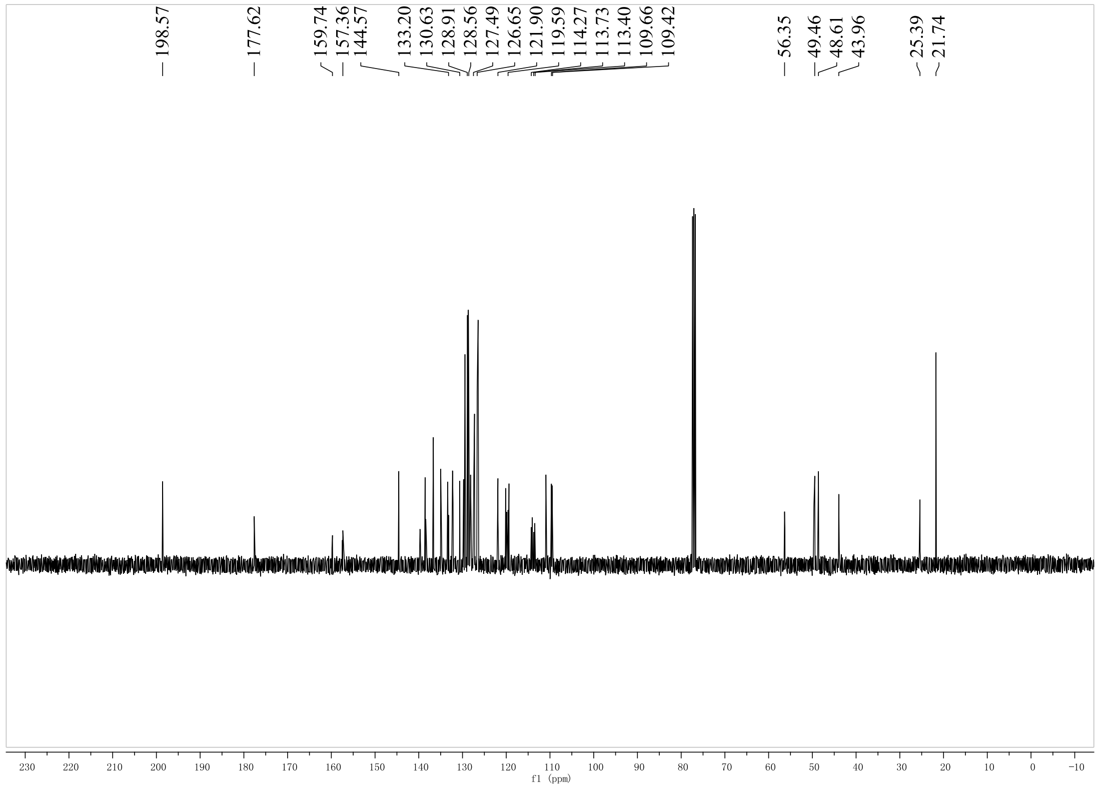
198.57
177.62
159.74
157.36
144.57
133.20
130.63
128.91
128.56
127.49
126.65
121.90
119.59
114.27
113.73
113.40
109.66
109.42
56.35
49.46
48.61
43.96
25.39
21.74
f1 (ppm)

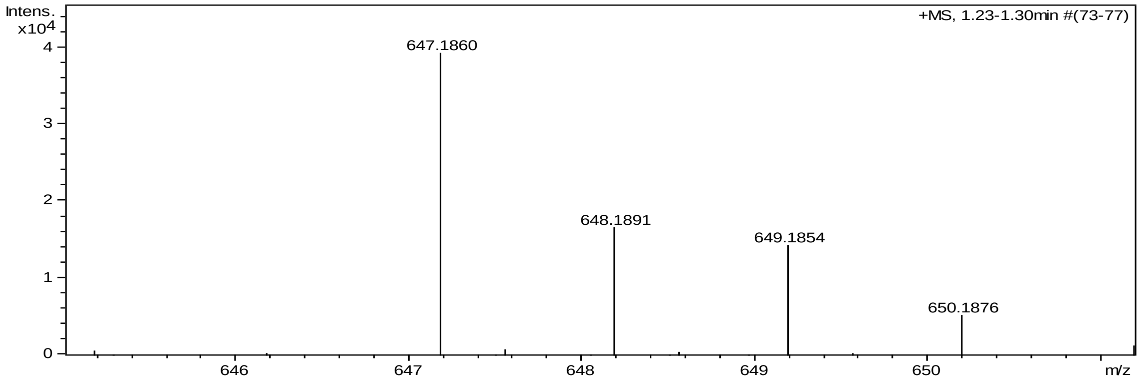
Intens.
x10^4
+MS, 1.23-1.30min #(73-77)
647.1860
648.1891
649.1854
650.1876
m/z

1'-Benzyl-4-(3-chlorophenyl)-5'-fluoro-2-(4-methylbenzoyl)-1,2,4,9-tetrahydrospiro[carbazole-3,3'-indolin]-2'-one (1f'):

purple solid, 5%, m.p. 212-214 °C; ^{1}H NMR (400 MHz, $CDCl_3$) δ: 7.99 (s, 1H, NH), 7.73 (d, J = 8.0 Hz, 2H, ArH), 7.32-7.30 (m, 2H, ArH), 7.28-7.25 (m, 2H, ArH), 7.21 (t, J = 8.0 Hz, 3H, ArH), 7.12-7.06 (m, 5H, ArH), 6.93 (t, J = 8.0 Hz, 1H, ArH), 6.89-6.86 (m, 1H, ArH), 6.85-6.76 (m, 3H, ArH), 6.61 (dd, J_1 = 8.4 Hz, J_2 = 4.4 Hz, 1H, ArH), 4.94 (d, J = 15.6 Hz, 1H, CH), 4.67 (s, 1H, CH), 4.57 (d, J = 8.8 Hz, 1H, CH), 4.53 (d, J = 10.0 Hz, 1H, CH_2), 3.75 (dd, J_1 = 16.8 Hz, J_2 = 6.4 Hz, 1H, CH), 3.11 (dd, J_1 = 16.8 Hz, J_2 = 8.4 Hz, 1H, CH), 2.38 (s, 3H, CH_3); ^{13}C NMR (400 MHz, $CDCl_3$) δ: 199.1, 176.1, 160.0, 157.6, 144.4, 141.5, 138.6, 138.6, 136.7, 135.6, 133.7, 133.5, 131.9, 129.9, 129.4, 129.0, 128.7, 128.6, 128.2, 127.5, 127.4, 127.3, 126.6, 121.8, 119.4, 118.9, 114.2 (d, J = 23.4 Hz), 112.8 (d, J = 25.9 Hz),, 110.8, 109.6, 109.3 (d, J = 8.1 Hz), 54.3, 47.1, 44.0, 43.9, 24.7, 21.6; IR (KBr) υ: 3330, 3300, 3259, 3146, 3083, 2958, 2878, 2842, 2174, 1863, 1625, 1617, 1542, 1438, 1334, 1255, 1157, 1119, 983, 917, 862, 781 cm^{-1}; MS (m/z): HRMS (ESI) Calcd. for $C_{40}H_{30}Cl^{35}FN_2O_2$ ($[M+Na]^+$): 647.1872, found: 647.1861. $C_{40}H_{30}Cl^{37}FN_2O_2$ ($[M+Na]^+$): 648.1906, found: 648.1890.

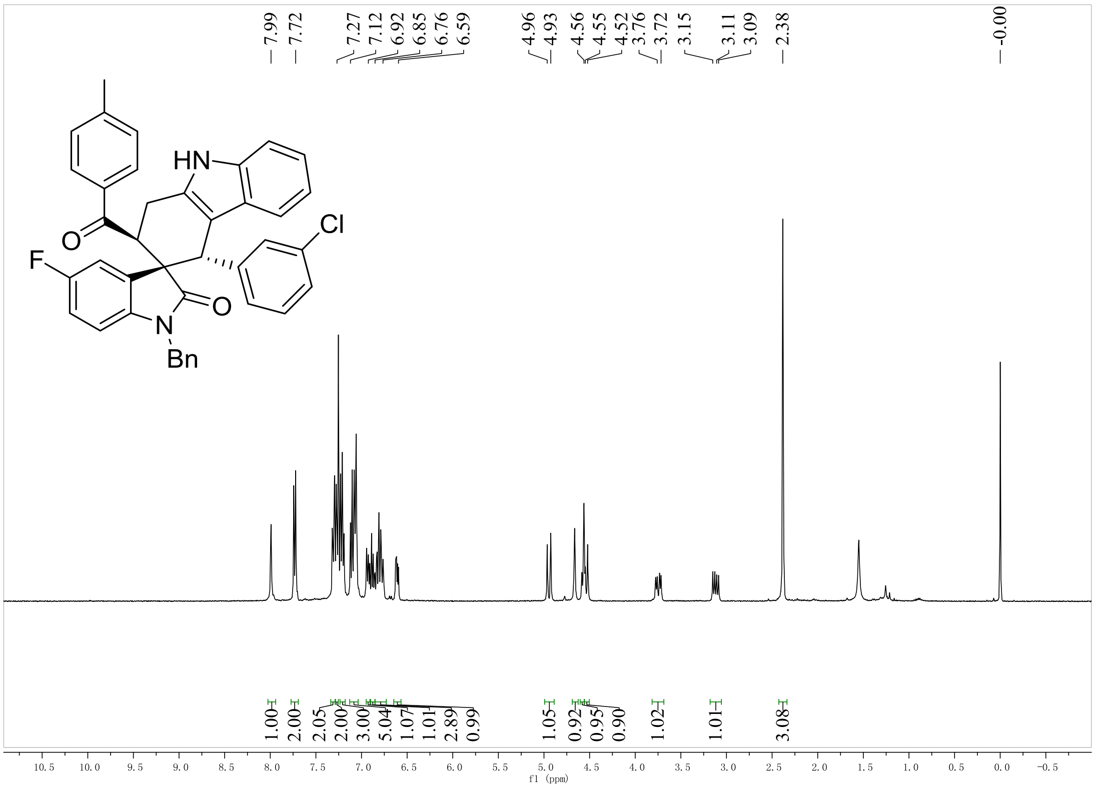

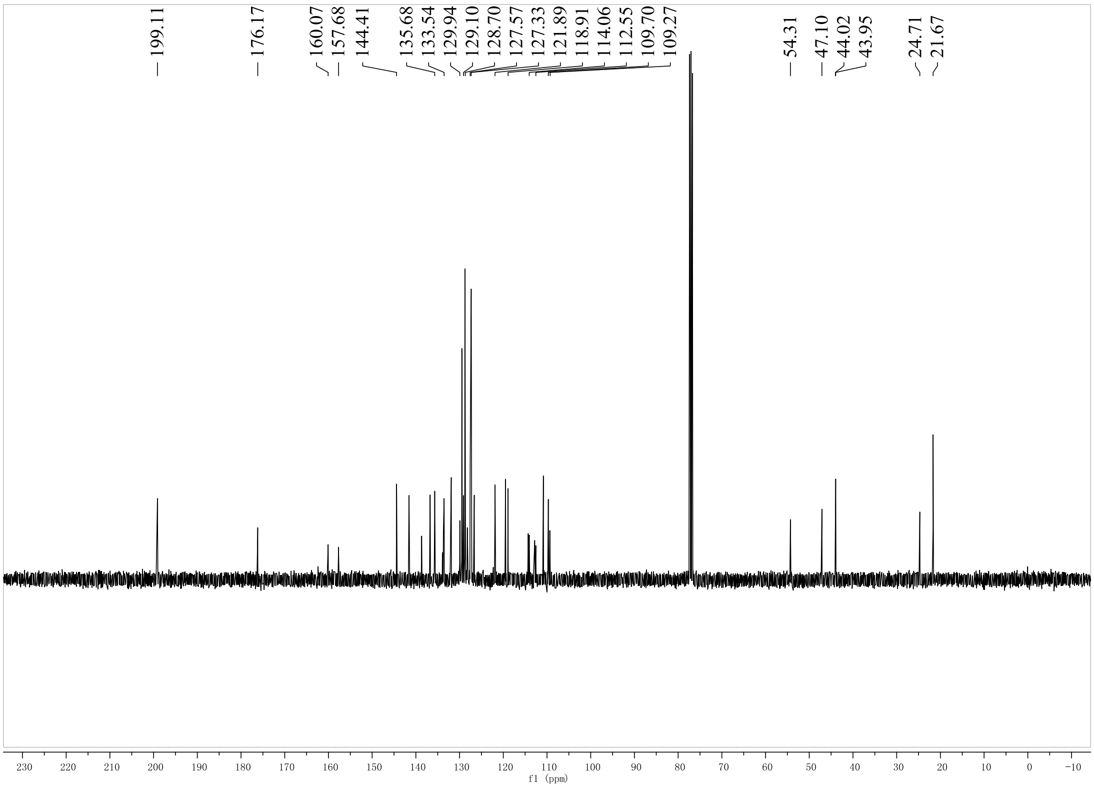

199.11
176.17
160.07
157.68
144.41
135.68
133.54
129.94
129.10
128.70
127.57
127.33
121.89
118.91
114.06
112.55
109.70
109.27
54.31
47.10
44.02
43.95
24.71
21.67
230 220 210 200 190 180 170 160 150 140 130 120 110 100 90 80 70 60 50 40 30 20 10 0 −10
f1 (ppm)

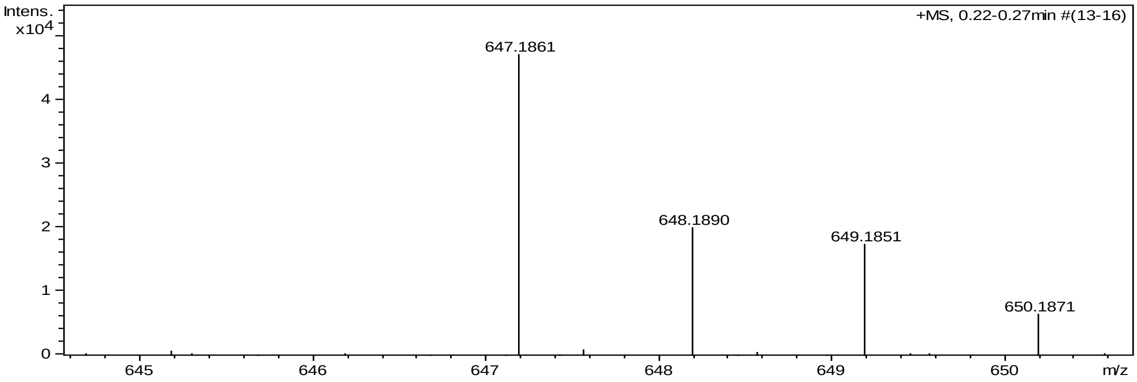

Intens.
x10^4
+MS, 0.22-0.27min #(13-16)
647.1861
648.1890
649.1851
650.1871
0
1
2
3
4
645
646
647
648
649
650
m/z

1'-Benzyl-2-(4-methylbenzoyl)-4-(4-nitrophenyl)-1,2,4,9-tetrahydrospiro[carbazole-3,3'-indolin]-2'-one (1g):

purple solid, 54%, m.p. 198-201 ºC; ^{1}H NMR (400 MHz, $CDCl_3$) δ: 8.22 (s, 1H, NH), 7.90 (dd, J_1 = 8.4 Hz, J_2 = 2.4 Hz, 1H, ArH), 7.84 (d, J = 8.0 Hz, 2H, ArH), 7.55 (dd, J_1 = 8.4 Hz, J_2 = 2.4 Hz, 1H, ArH), 7.46 (d, J = 8.0 Hz, 1H, ArH), 7.38 (d, J = 8.0 Hz, 1H, ArH), 7.27-7.26 (m, 2H, ArH), 7.19 (t, J = 7.2 Hz, 1H, ArH), 7.13-7.09 (m, 4H, ArH), 7.05 (d, J = 7.6 Hz, 1H, ArH), 6.86 (d, J = 7.6 Hz, 1H, ArH), 6.82 (t, J = 7.6 Hz, 3H, ArH), 6.54 (d, J = 8.8 Hz, 1H, ArH), 6.42 (d, J = 5.6 Hz, 1H, ArH), 6.24 (d, J = 8.0 Hz, 1H, ArH), 5.11 (s, 1H, CH), 4.84 (dd, J_1 = 12.4 Hz, J_2 = 5.6 Hz, 1H, CH), 4.55-4.54 (m, 2H, CH_2), 3.60-3.53 (m, 1H, CH), 3.36 (dd, J_1 = 16.8 Hz, J_2 = 5.6 Hz, 1H, CH), 2.42 (s, 3H, CH_3); ^{13}C NMR (400 MHz, $CDCl_3$) δ: 198.2, 177.4, 146.8, 144.4, 143.3, 136.5, 135.1, 133.4, 132.9, 131.4, 130.7, 129.3, 128.8, 128.4, 128.3, 127.4, 127.0, 126.1, 125.5, 122.8, 122.0, 121.9, 121.5, 119.6, 119.5, 110.9, 109.1, 109.1, 55.8, 49.4, 48.3, 43.8, 25.5, 21.7; IR(KBr) υ: 3300, 3270, 3217, 3148, 3043, 2967, 2831, 2800, 2169, 1855, 1637, 1600, 1549, 1434, 1338, 1267, 1138, 1171, 963, 955, 863, 754 cm^{-1}; MS (m/z): HRMS (ESI) Calcd. for $C_{40}H_{31}N_3O_4$ ([M+Na]$^+$): 640.2207, found: 640.2196.

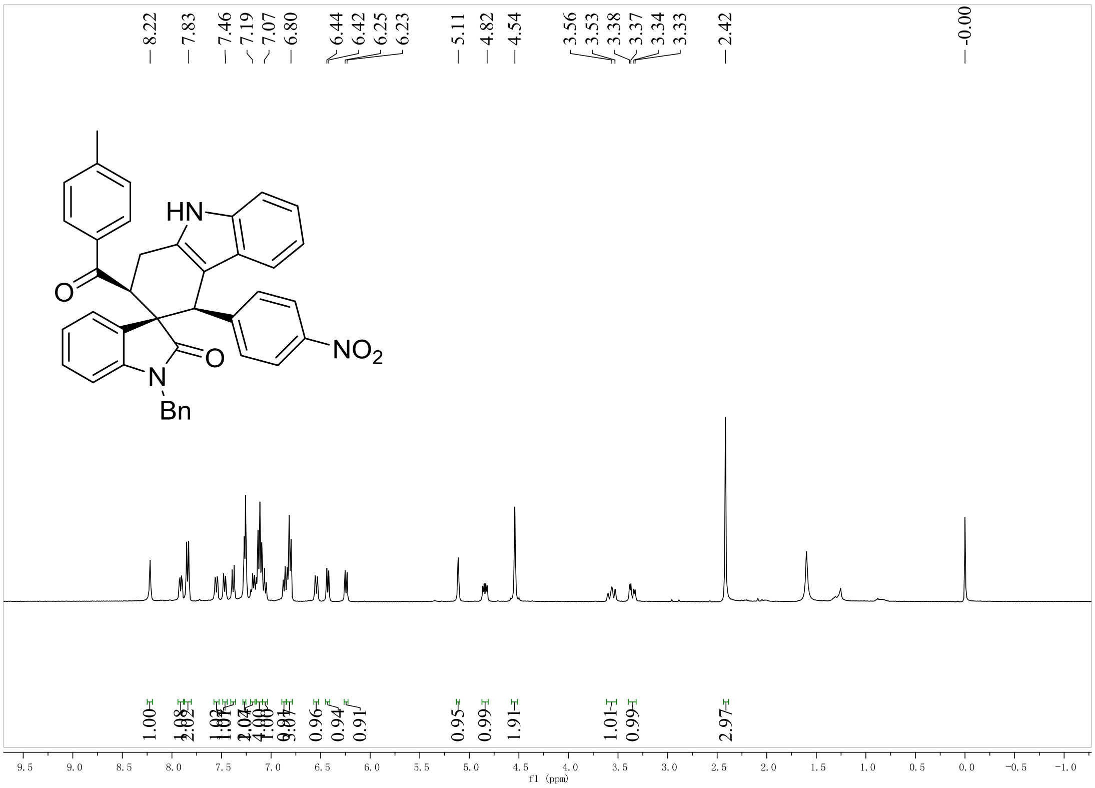

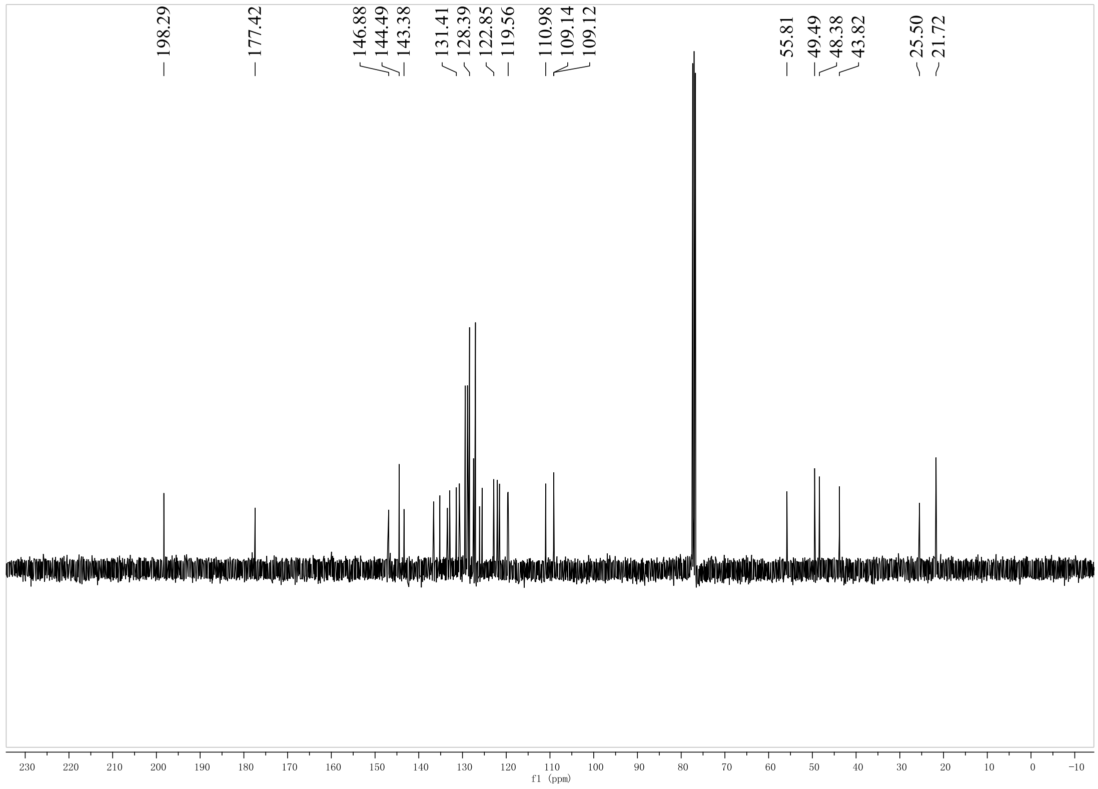

198.29
177.42
146.88
144.49
143.38
131.41
128.39
122.85
119.56
110.98
109.14
109.12
55.81
49.49
48.38
43.82
25.50
21.72
f1 (ppm)

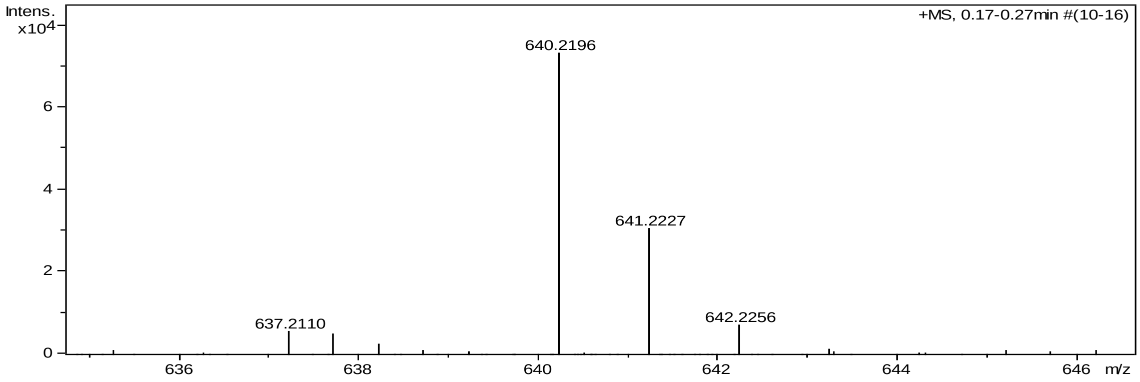

Intens.
x10^4
+MS, 0.17-0.27min #(10-16)
640.2196
641.2227
642.2256
637.2110
m/z

1'-Benzyl-2-(4-methylbenzoyl)-4-(4-nitrophenyl)-1,2,4,9-tetrahydrospiro[carbazole-3,3'-indolin]-2'-one (1g'):

purple solid, 9%, m.p. 211-213 ºC; ^{1}H NMR (400 MHz, $CDCl_3$) δ: 8.09 (s, 1H, NH), 7.89 (d, *J* = 7.6 Hz, 2H, ArH), 7.69 (d, *J* = 7.2 Hz, 2H, ArH), 7.29 (d, *J* = 7.6 Hz, 2H, ArH), 7.21-7.14 (m, 6H, ArH), 7.13-7.09 (m, 3H, ArH), 6.94-6.89 (m, 3H, ArH), 6.84 (t, *J* = 7.2 Hz, 1H, ArH), 6.70 (t, *J* = 7.2 Hz, 1H, ArH), 6.58 (d, *J* = 7.2 Hz, 1H, ArH), 5.12 (s, 1H, CH), 4.87 (d, *J* = 15.6 Hz, 1H, CH), 4.52 (d, *J* = 15.6 Hz, 1H, CH), 4.39 (s, 1H, CH), 3.92-3.86 (m, 1H, CH), 3.11-3.06 (m, 1H, CH), 2.38 (s, 3H, CH_3); ^{13}C NMR (400 MHz, $CDCl_3$) δ: 198.7, 175.9, 147.1, 146.8, 144.4, 142.6, 136.5, 135.5, 133.6, 132.3, 130.9, 129.4, 128.6, 128.5, 127.6, 127.2, 126.4, 125.0, 122.8, 122.7, 121.7, 119.4, 118.9, 110.8, 109.2, 109.0, 53.9, 46.3, 44.0, 43.7, 24.2, 21.6; IR (KBr) υ: 3331, 3300, 3267, 3175, 3067, 2938, 2866, 2817, 2167, 1855, 1632, 1618, 1544, 1467, 1378, 1263, 1154, 1117, 963, 944, 869, 771 cm^{-1}; MS (*m*/*z*): HRMS (ESI) Calcd. for $C_{40}H_{31}N_3O_4$ ($[M+Na]^+$): 640.2207, found: 640.2200.

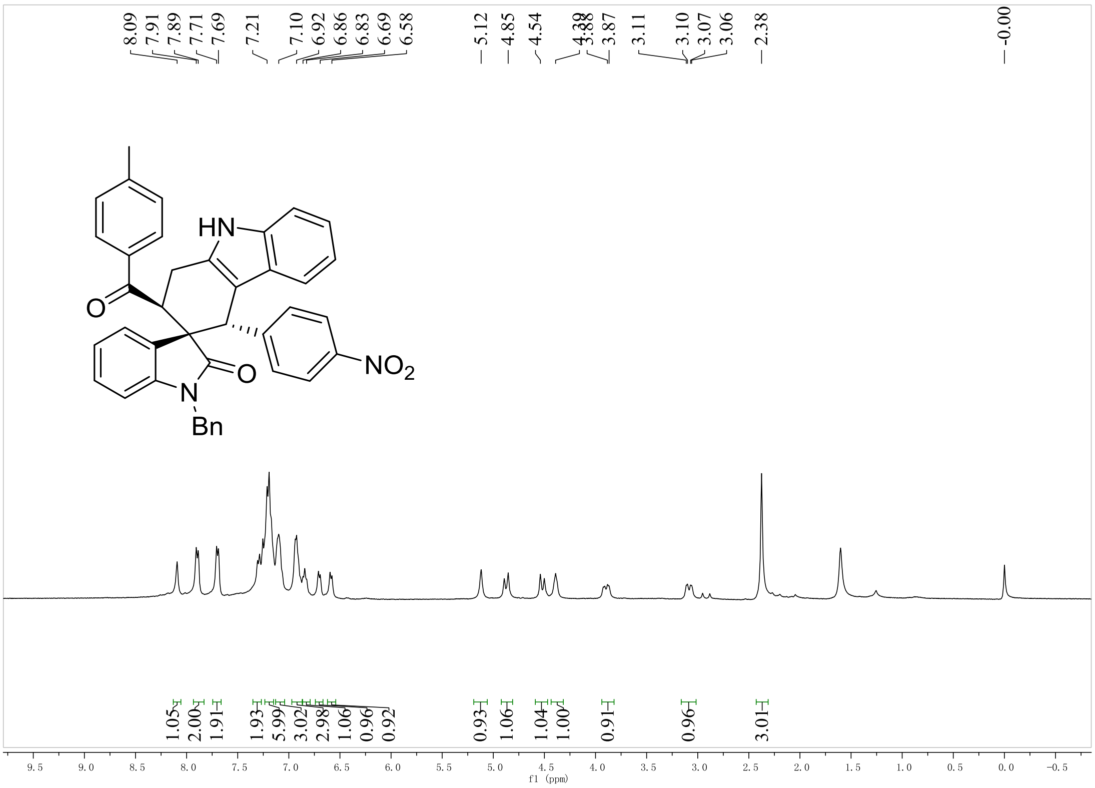

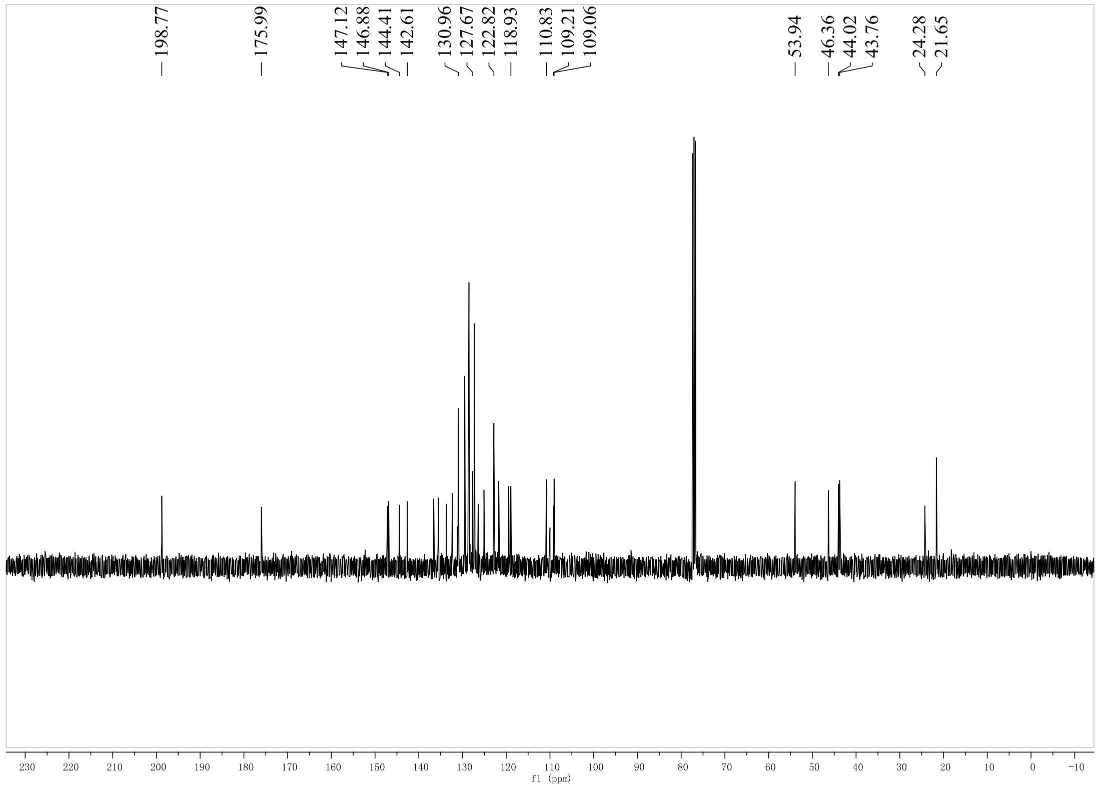
198.77
175.99
147.12
146.88
144.41
142.61
130.96
127.67
122.82
118.93
110.83
109.21
109.06
53.94
46.36
44.02
43.76
24.28
21.65
f1 (ppm)

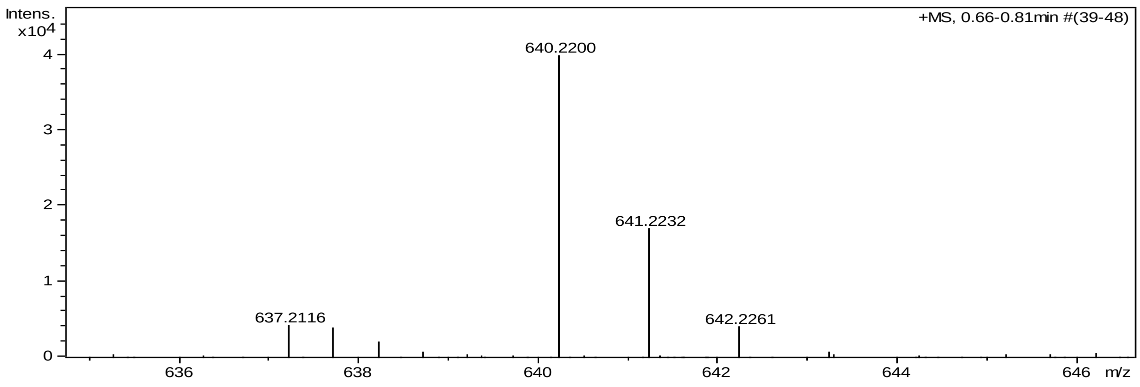
Intens.
x10^4
+MS, 0.66-0.81min #(39-48)
640.2200
641.2232
637.2116
642.2261
m/z

1'-Benzyl-5'-chloro-2-(4-chlorobenzoyl)-4-(4-nitrophenyl)-1,2,4,9-tetrahydrospiro[carbazole-3,3'-indolin]-2'-one (1h):

purple solid, 57%, m.p. 196-199 ºC; ^{1}H NMR (400 MHz, $CDCl_3$) δ: 8.26 (s, 1H, NH), 7.92 (dd, J_1 = 8.4 Hz, J_2 = 2.4 Hz, 1H, ArH), 7.86 (d, J = 8.8 Hz, 2H, ArH), 7.64 (dd, J_1 = 8.4 Hz, J_2 = 2.4 Hz, 1H, ArH), 7.44-7.40 (m, 4H, ArH), 7.23-7.12 (m, 4H, ArH), 7.07-7.05 (m, 2H, ArH), 6.87-6.81 (m, 3H, ArH), 6.61 (dd, J_1 = 8.8 Hz, J_2 = 1.6 Hz, 1H, ArH), 6.36 (d, J = 8.8 Hz, 1H, ArH), 6.26 (d, J = 8.0 Hz, 1H, ArH), 5.09 (s, 1H, CH), 4.78 (dd, J_1 = 12.4 Hz, J_2 = 5.6 Hz, 1H, CH), 4.54-4.53 (m, 2H, CH_2), 3.54-3.46 (m, 1H, CH), 3.34 (dd, J_1 = 16.8 Hz, J_2 = 5.6 Hz, 1H, CH); ^{13}C NMR (400 MHz, $CDCl_3$) δ: 197.4, 177.0, 147.0, 143.8, 142.0, 140.2, 136.6, 134.6, 133.9, 132.1, 131.2, 130.5, 130.1, 129.1, 129.1, 128.5, 128.4, 127.7, 127.5, 127.0, 125.9, 125.6, 123.0, 122.3, 121.8, 119.8, 119.7, 111.1, 110.0, 108.9, 55.8, 49.3, 48.5, 43.9, 25.3; IR (KBr) υ: 3354, 3310, 3267, 3149, 3066, 2955, 2871, 2846, 2176, 1848, 1655, 1600, 1531, 1414, 1317, 1248, 1167, 1130, 992, 917, 867, 768 cm^{-1}; MS (m/z): HRMS (ESI) Calcd. for $C_{39}H_{27}Cl_2{}^{35}N_3O_4$ ($[M+Na]^+$): 694.1271, found: 694.1256. $C_{39}H_{27}Cl_2{}^{37}N_3O_4$ ($[M+Na]^+$): 696.1241, found: 696.1238.

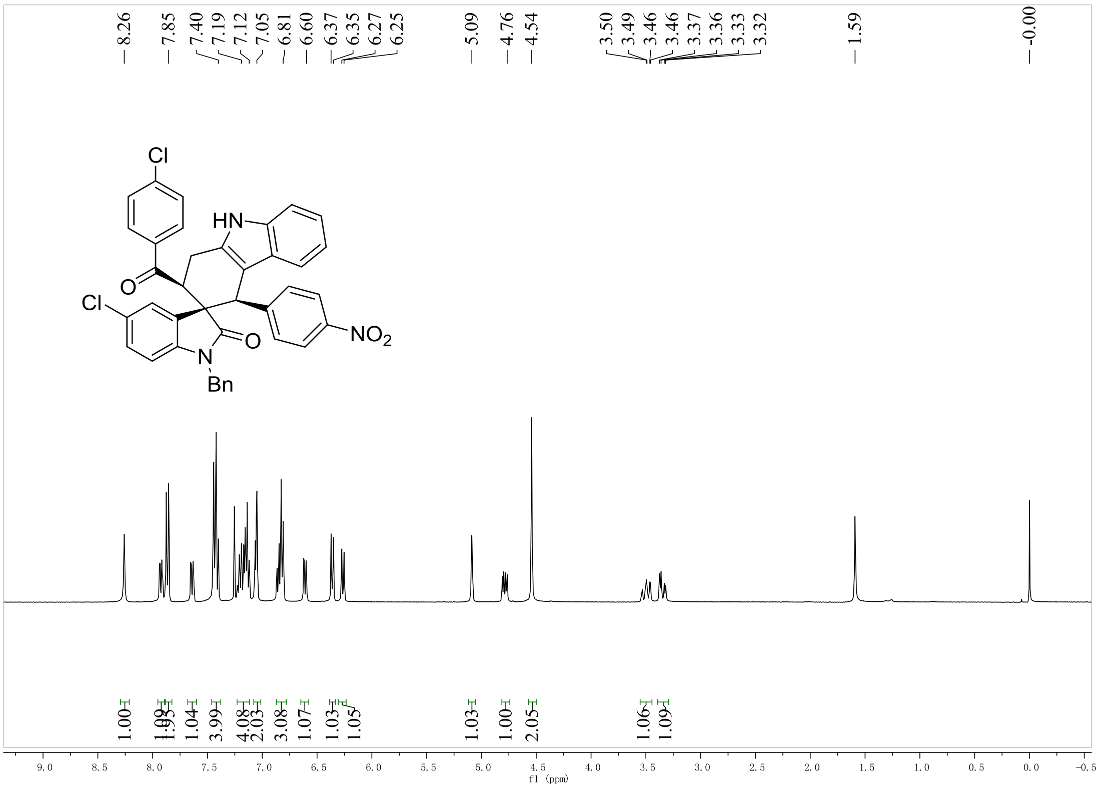

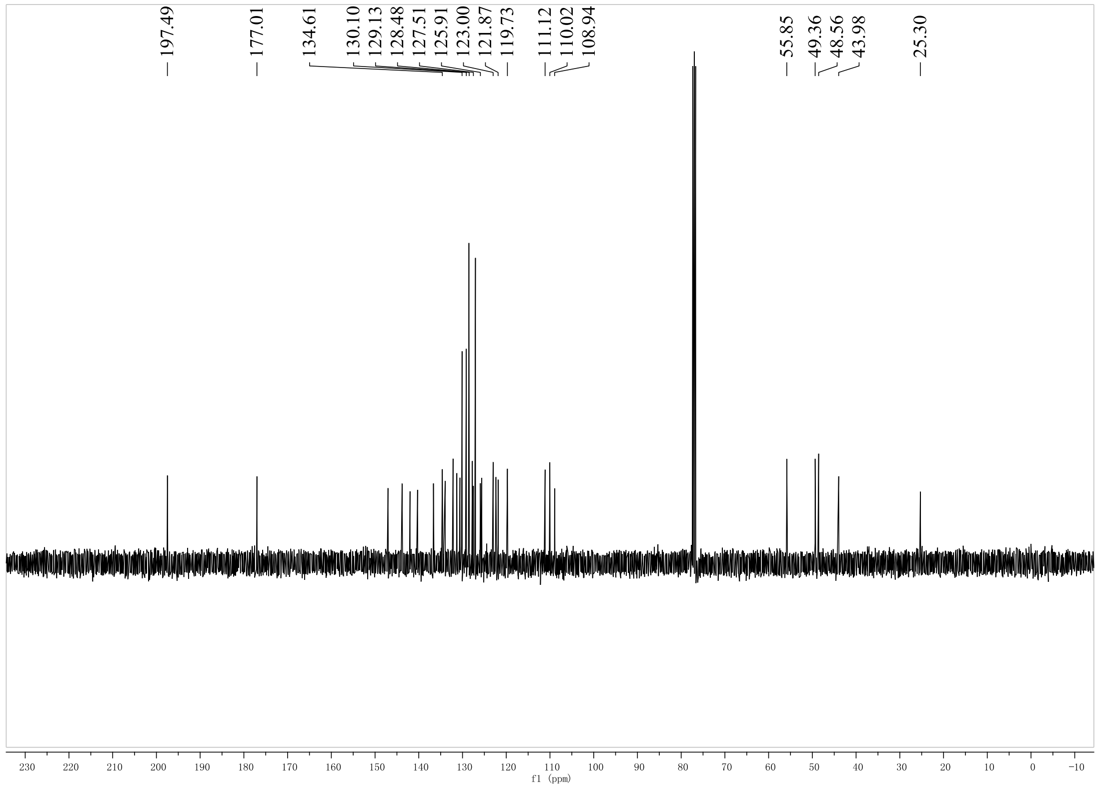
197.49
177.01
134.61
130.10
129.13
128.48
127.51
125.91
123.00
121.87
119.73
111.12
110.02
108.94
55.85
49.36
48.56
43.98
25.30
230 220 210 200 190 180 170 160 150 140 130 120 110 100 90 80 70 60 50 40 30 20 10 0 −10
f1 (ppm)

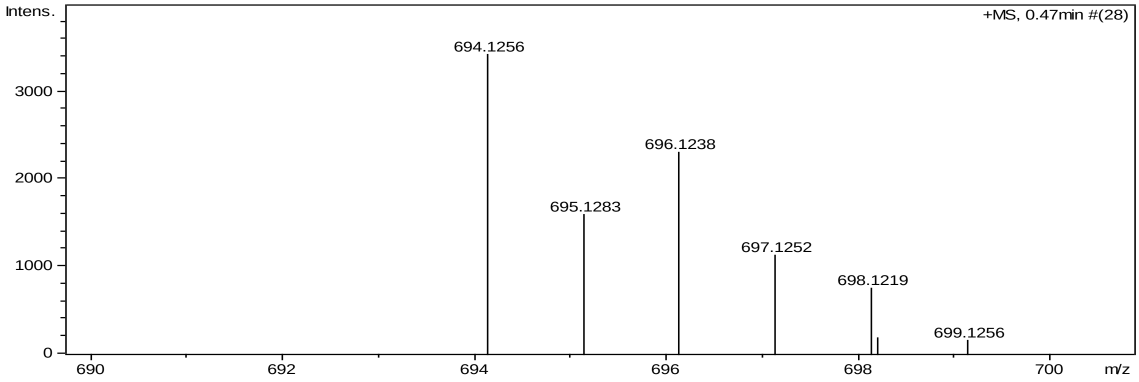
Intens.
+MS, 0.47min #(28)
694.1256
695.1283
696.1238
697.1252
698.1219
699.1256
0
1000
2000
3000
690 692 694 696 698 700 m/z

1'-Benzyl-5'-chloro-2-(4-chlorobenzoyl)-4-(3-nitrophenyl)-1,2,4,9-tetrahydrospiro[carbazole-3,3'-indolin]-2'-one (1i):

purple solid, 49%, m.p. 201-204 ºC; ^{1}H NMR (400 MHz, $CDCl_3$) δ: 8.21 (s, 1H, NH), 7.99 (t, *J* = 8.0 Hz, 1H, ArH), 7.90-7.87 (m, 2H, ArH), 7.46-7.40 (m, 3H, ArH), 7.31-7.26 (m, 2H, ArH), 7.24-7.06 (m, 5H, ArH), 7.05-6.96 (m, 2H, ArH), 6.86-6.77 (m, 3H, ArH), 6.34-6.23 (m, 2H, ArH), 5.10 (d, *J* =9.6 Hz, 1H, CH), 4.85-4.78 (m, 1H, CH), 4.64-4.57 (m, 1H, CH), 4.48-4.41 (m, 1H, CH), 3.57-3.49 (m, 1H, CH), 3.40-3.33 (m, 1H, CH); ^{13}C NMR (400 MHz, $CDCl_3$) δ: 197.6, 177.1, 147.5, 147.1, 142.1, 141.7, 140.2, 138.5, 136.7, 136.3, 135.9, 134.6, 133.9, 132.3, 130.1, 129.1, 128.6, 128.5, 128.4, 128.3, 127.7, 127.6, 127.6, 127.4, 126.9, 126.7, 125.9, 125.7, 125.6, 125.0, 124.5, 122.5, 122.3, 122.2, 122.1, 119.8, 119.8, 119.6, 119.2, 111.2, 111.1, 110.0, 109.9, 108.8, 55.8, 49.3, 48.5, 43.9, 25.2; IR (KBr) υ: 3355, 3238, 3146, 3067, 2955, 2848, 2168, 1848, 1637, 1617, 1555, 1447, 1362, 1276, 1155, 1168, 966, 903, 855, 769 cm^{-1}; MS (*m*/*z*): HRMS (ESI) Calcd. for $C_{39}H_{27}Cl_2{}^{35}N_3O_4$ ($[M+Na]^+$): 694.1271, found: 694.1255. $C_{39}H_{27}Cl_2{}^{37}N_3O_4$ ($[M+Na]^+$): 696.1241, found: 696.1239.

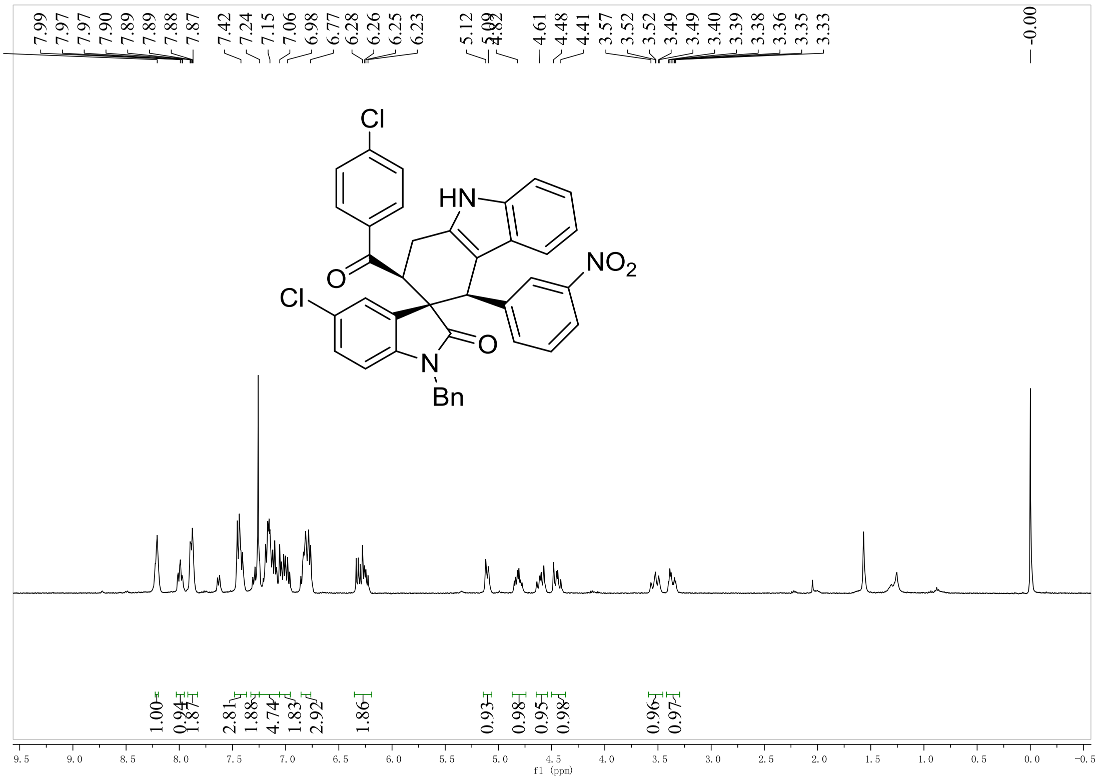

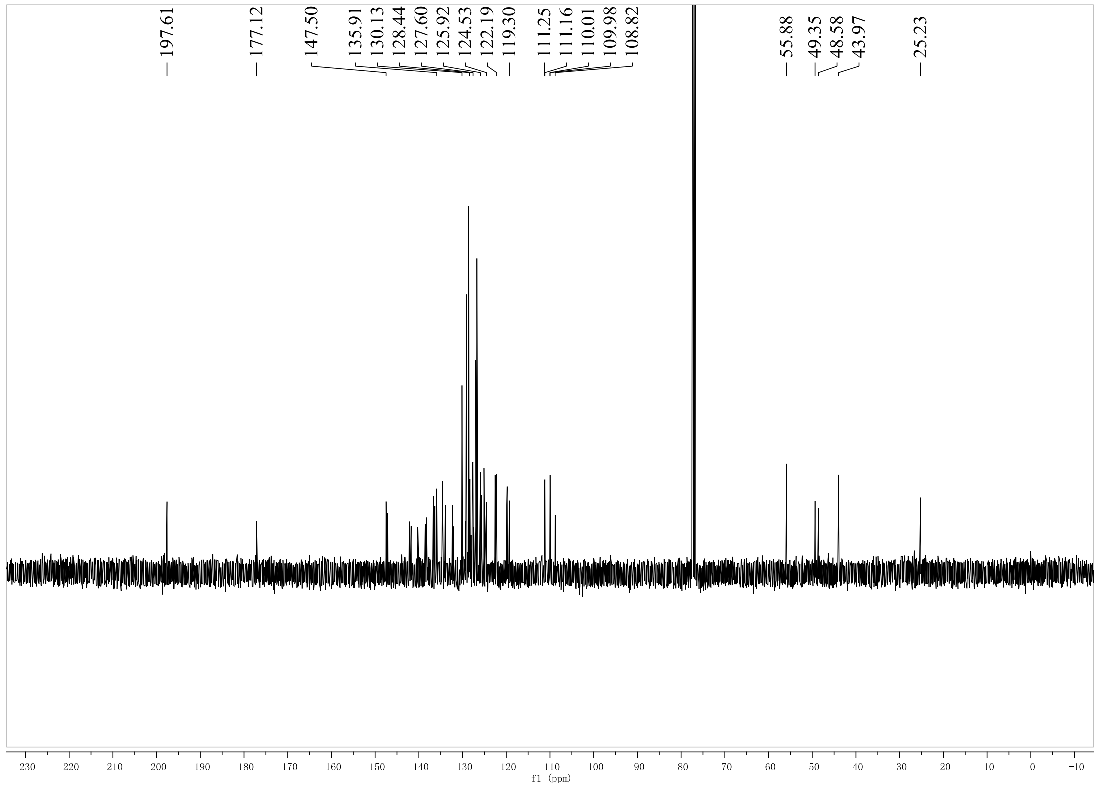
197.61
177.12
147.50
135.91
130.13
128.44
127.60
125.92
124.53
122.19
119.30
111.25
111.16
110.01
109.98
108.82
55.88
49.35
48.58
43.97
25.23
f1 (ppm)

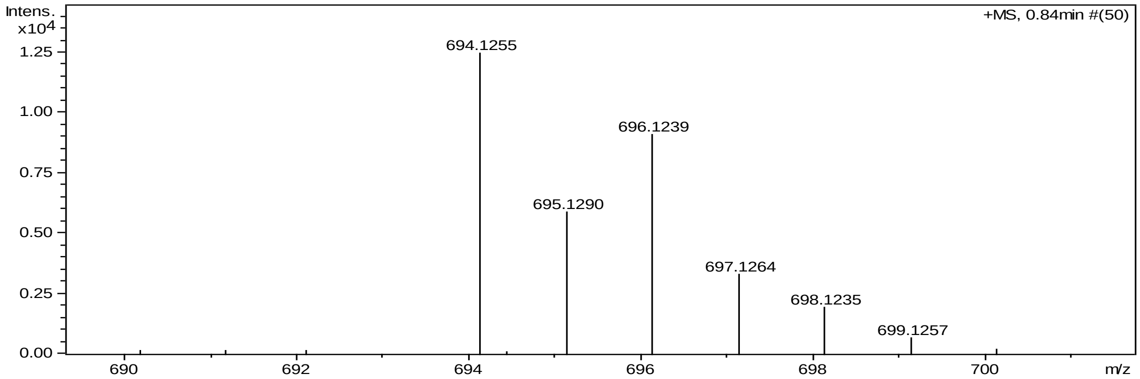
Intens.
x10^4
+MS, 0.84min #(50)
694.1255
696.1239
695.1290
697.1264
698.1235
699.1257
m/z

1'-Benzyl-5'-chloro-2-(4-chlorobenzoyl)-4-(3-nitrophenyl)-1,2,4,9-tetrahydrospiro[carbazole-3,3'-indolin]-2'-one (1i'):

purple solid, 6%, m.p. 216-219 ºC; ^{1}H NMR (400 MHz, $CDCl_3$) δ: 8.12-8.09 (m, 2H, NH), 7.94 (s, 1H, NH), 7.75 (d, J = 7.6 Hz, 2H, ArH), 7.41-7.35 (m, 5H, ArH), 7.29-7.26 (m, 2H, ArH), 7.18-7.12 (m, 5H, ArH), 6.94-6.88 (m, 2H, ArH), 6.75 (dd, J_1 = 19.2 Hz, J_2 = 7.6 Hz, 2H, ArH), 4.88 (d, J = 15.2 Hz, 1H, CH), 4.68 (s, 1H, CH), 4.59 (d, J = 15.2 Hz, 1H, CH), 4.56-4.53 (m, 1H, CH), 3.72-3.66 (m, 1H, CH), 3.24-3.17 (m, 1H, CH); ^{13}C NMR (400 MHz, $CDCl_3$) δ: 198.0, 175.8, 147.9, 141.7, 141.2, 140.1, 136.8, 135.9, 135.2, 134.1, 133.5, 131.8, 129.9, 129.1, 128.8, 128.3, 128.0, 127.8, 127.3, 126.2, 124.5, 124.5, 124.4, 122.5, 122.3, 119.8, 118.5, 111.1, 110.1, 109.0, 53.9, 47.1, 44.1, 44.0, 24.6 cm^{-1}; IR (KBr) υ: 3341, 3278, 3166, 3048, 2966, 2849, 2158, 1871, 1671, 1643, 1522, 1458, 1331, 1268, 1148, 1137, 954, 913, 832, 767 cm^{-1}; MS (m/z): HRMS (ESI) Calcd. for $C_{39}H_{27}Cl_2{}^{35}N_3O_4$ ($[M+Na]^+$): 694.1271, found: 694.1256. $C_{39}H_{27}Cl_2{}^{37}N_3O_4$ ($[M+Na]^+$): 696.1241, found: 696.1236.

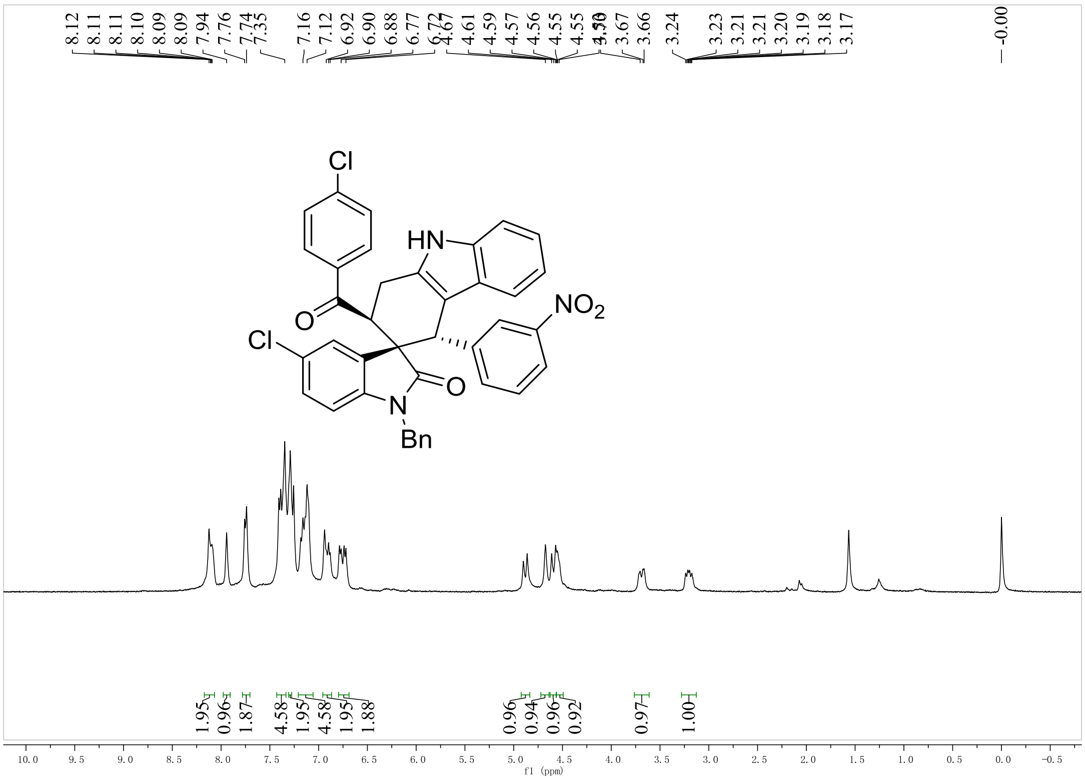

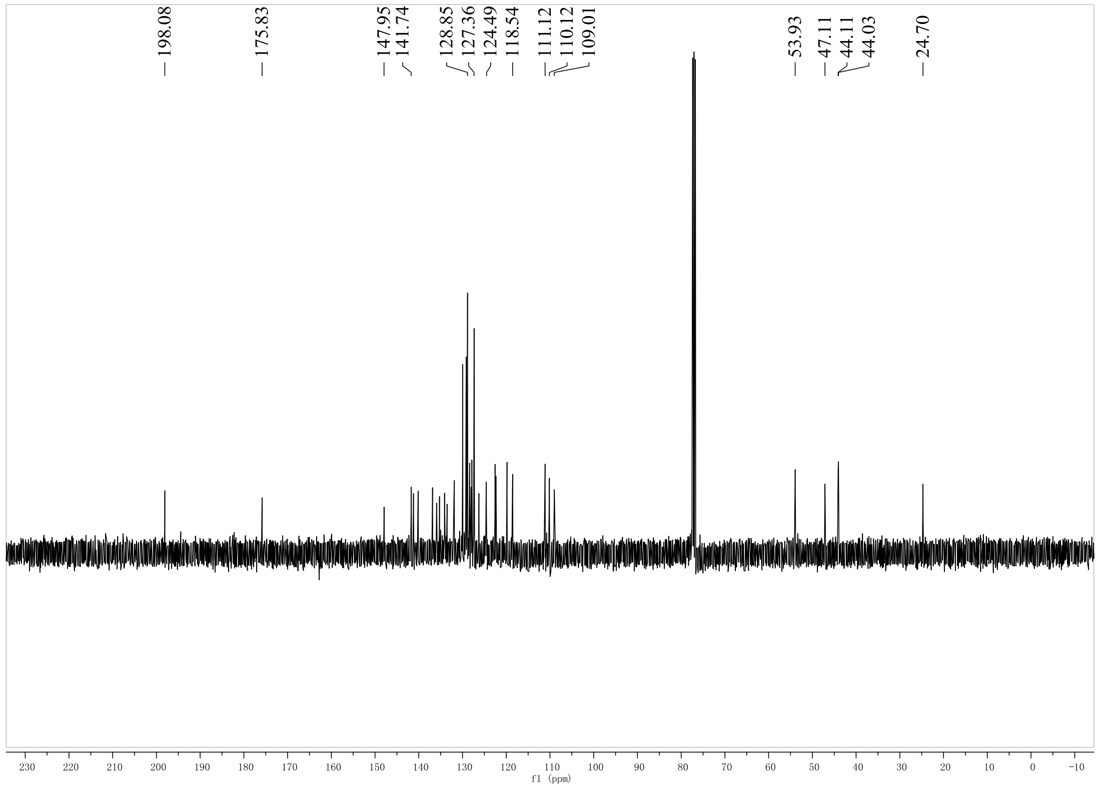
198.08
175.83
147.95
141.74
128.85
127.36
124.49
118.54
111.12
110.12
109.01
53.93
47.11
44.11
44.03
24.70
f1 (ppm)

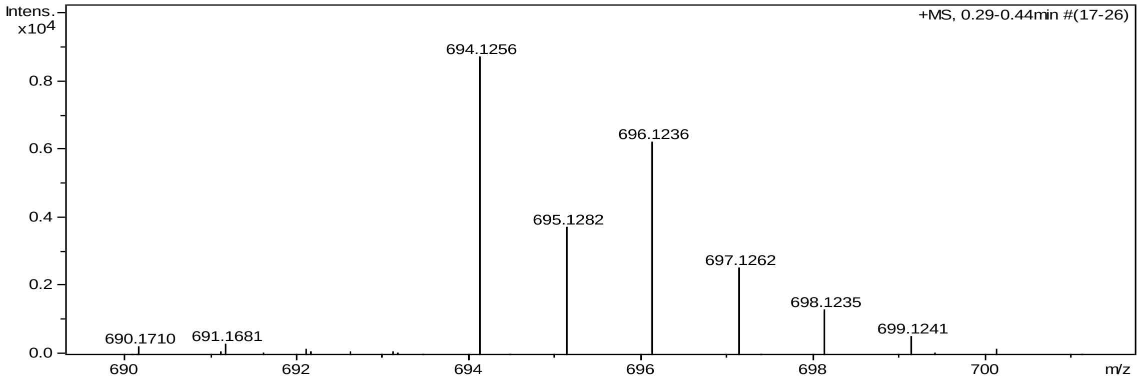
Intens.
x10^4
+MS, 0.29-0.44min #(17-26)
694.1256
696.1236
695.1282
697.1262
698.1235
699.1241
690.1710
691.1681
m/z

1'-Benzyl-5'-chloro-2-(4-methoxybenzoyl)-4-(4-methoxyphenyl)-1,2,4,9-tetrahydrospiro[carbazole-3,3'-indolin]-2'-one (1j):

purple solid, 65%, m.p. 189-192 ºC; ^{1}H NMR (400 MHz, $CDCl_3$) δ: 8.60 (d, J = 2.0 Hz, 1H, NH), 7.63 (d, J = 2.0 Hz, 1H, ArH), 7.48 (d, J = 7.6 Hz, 2H, ArH), 7.35 (t, J = 7.6 Hz, 2H, ArH), 7.28 (d, J = 7.6 Hz, 1H, ArH), 7.20 (d, J = 8.4 Hz, 3H, ArH), 7.16-7.13 (m, 2H, ArH), 6.96 (dd, J_1 = 8.4 Hz, J_2 = 2.0 Hz, 1H, ArH), 6.78 (d, J = 8.8 Hz, 3H, ArH), 6.65 (d, J = 8.8 Hz, 2H, ArH), 6.57 (t, J = 8.8 Hz, 2H, ArH), 6.13 (d, J = 8.4 Hz, 1H, ArH), 5.81 (s, 1H, CH), 5.23 (d, J = 15.6 Hz, 1H, CH), 5.17-5.16 (m, 1H, CH), 5.16-5.14 (m, 2H, CH_2), 3.79 (d, J = 15.6 Hz, 1H, CH), 3.78 (s, 3H, OCH_3), 3.72 (s, 3H, OCH_3); ^{13}C NMR (400 MHz, $CDCl_3$) δ: 195.0, 177.6 (d, J = 8.0 Hz), 162.6, 159.6, 147.1, 141.6, 140.7, 135.6, 134.4, 132.9, 130.8, 130.1, 129.5, 129.4, 129.1, 128.8, 128.8, 128.7, 128.5, 127.9, 127.6, 127.5, 127.5, 127.2, 127.2, 125.8, 124.2, 113.6, 112.9, 110.3, 109.2, 67.4, 65.6, 60.9, 55.4, 55.1, 44.5, 44.3; IR (KBr) υ: 3300, 3284, 3180, 3078, 2967, 2849, 2831, 2156, 1833, 1619, 1600, 1531, 1429, 1333, 1219, 1117, 1100, 955, 948, 860, 769 cm^{-1}; MS (m/z): HRMS (ESI) Calcd. for $C_{41}H_{33}Cl^{35}N_2O_4$ ([M+Na]$^+$): 675.2021, found: 675.2008. $C_{41}H_{33}Cl^{37}N_2O_4$ ([M+Na]$^+$): 676.2055, found: 676.2040.

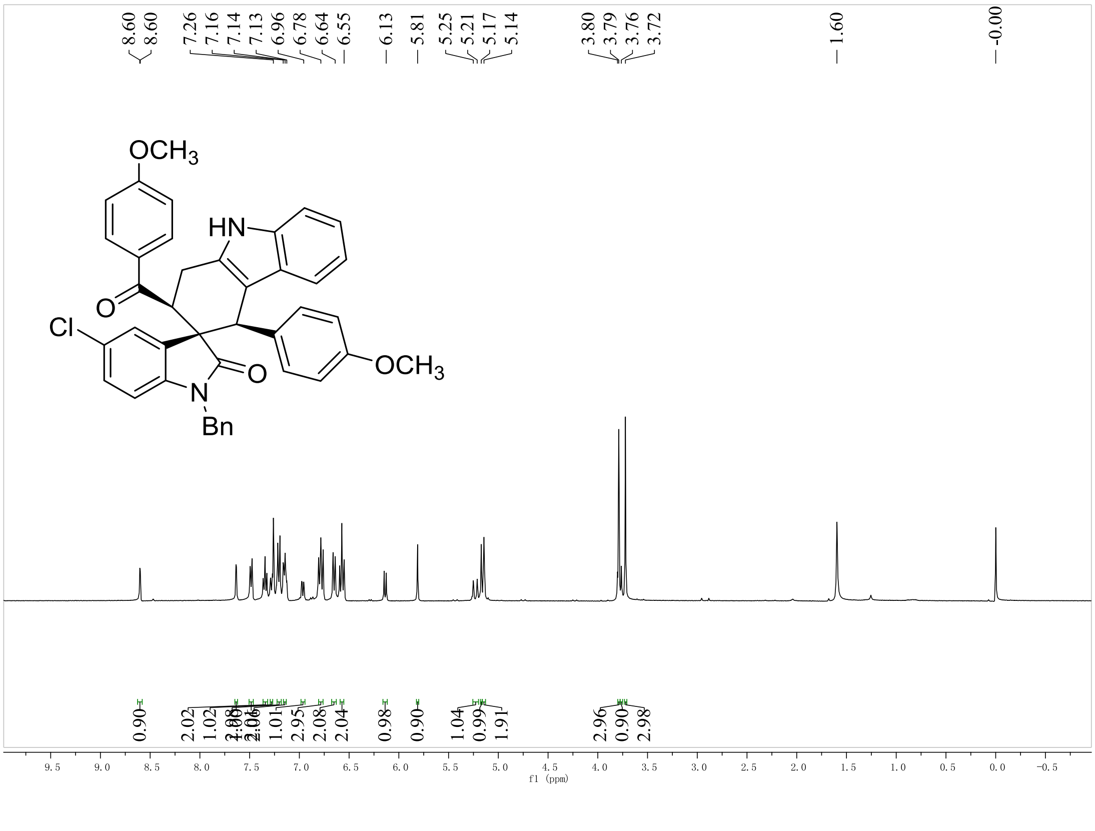

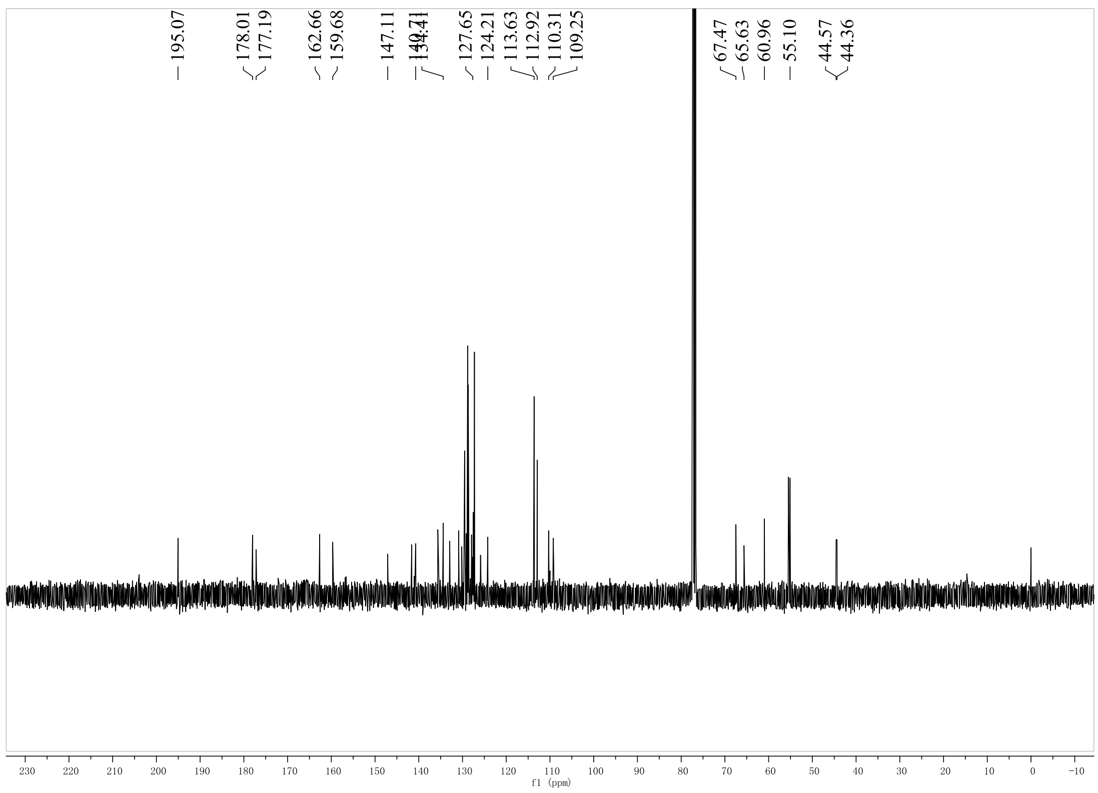
195.07
178.01
177.19
162.66
159.68
147.11
140.71
134.41
127.65
124.21
113.63
112.92
110.31
109.25
67.47
65.63
60.96
55.10
44.57
44.36
f1 (ppm)

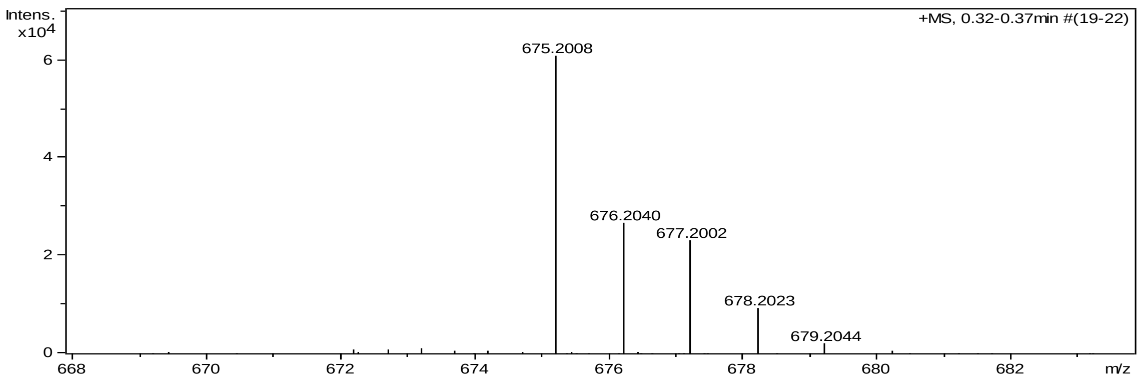
Intens.
x10^4
+MS, 0.32-0.37min #(19-22)
675.2008
676.2040
677.2002
678.2023
679.2044
m/z

1'-Benzyl-2'-oxo-4-phenyl-4,9-dihydrospiro[carbazole-2,3'-indoline]-3,3(1*H*)-dicarbonitrile (2a):

white solid, 51%, m.p. 201-204 ºC; ^{1}H NMR (400 MHz, $CDCl_3$) δ: 8.27 (s, 1H, NH), 7.52-7.46 (m, 3H, ArH), 7.43-7.38 (m, 4H, ArH), 7.35-7.28 (m, 5H, ArH), 7.20 (t, J =7.6 Hz, 1H, CH), 6.95-6.90 (m, 4H, ArH), 6.54 (d, J =8.0 Hz, 1H, CH), 5.14 (d, J =15.6 Hz, 1H, CH), 4.96 (d, J =15.6 Hz, 1H, CH), 4.89 (s, 1H, CH), 4.10 (dd, J_1 = 16.4 Hz, J_2 = 2.4 Hz, 1H, CH), 2.96 (d, J = 16.4 Hz, 1H, CH); ^{13}C NMR (400 MHz, $CDCl_3$) δ: 173.2, 136.6, 134.7, 133.6, 130.9, 129.4, 128.8, 128.0, 127.6, 125.0, 123.6, 122.7, 120.2, 120.1, 112.7, 111.0, 110.4, 106.9, 51.8, 47.6, 46.2, 44.7, 29.1; IR (KBr) υ: 3355, 3207, 3117, 3048, 2963, 2831, 2167, 1871, 1641, 1633, 1554, 1431, 1370, 1240, 1131, 1100, 972, 961, 881, 764 cm^{-1}; MS (m/z): HRMS (ESI) Calcd. for $C_{34}H_{24}N_4O$ ([M+Na]$^+$): 527.1842, found: 527.1849.

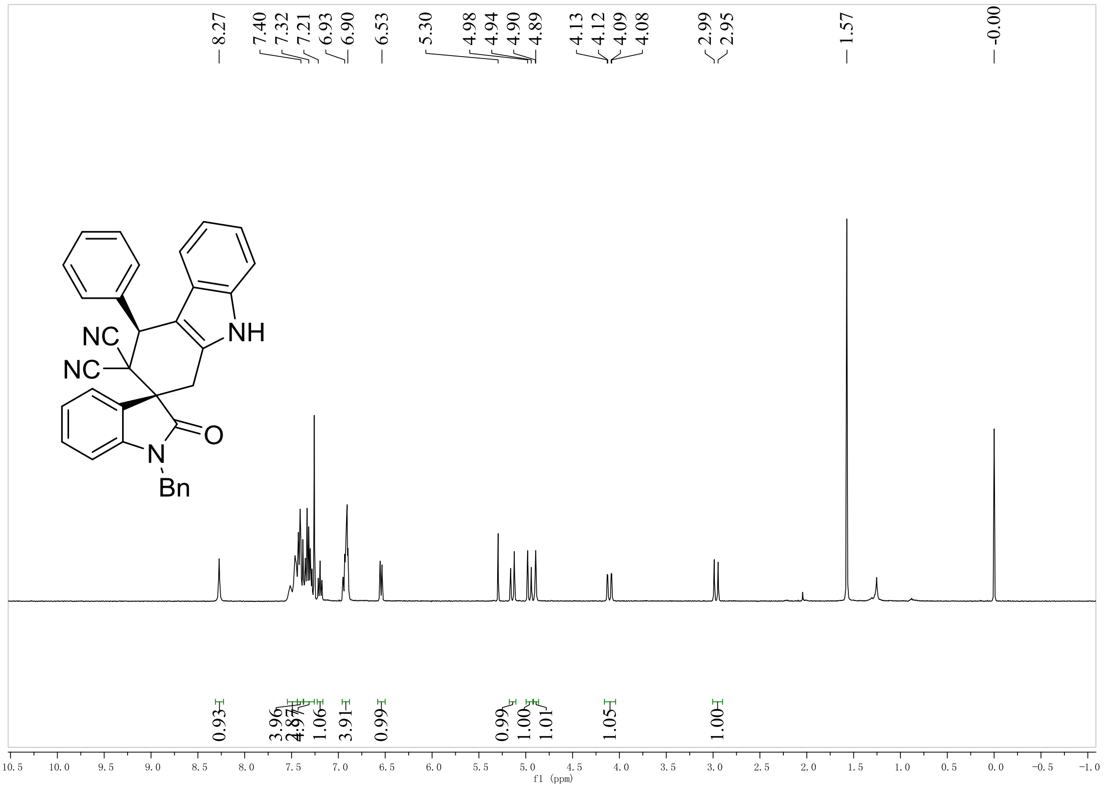

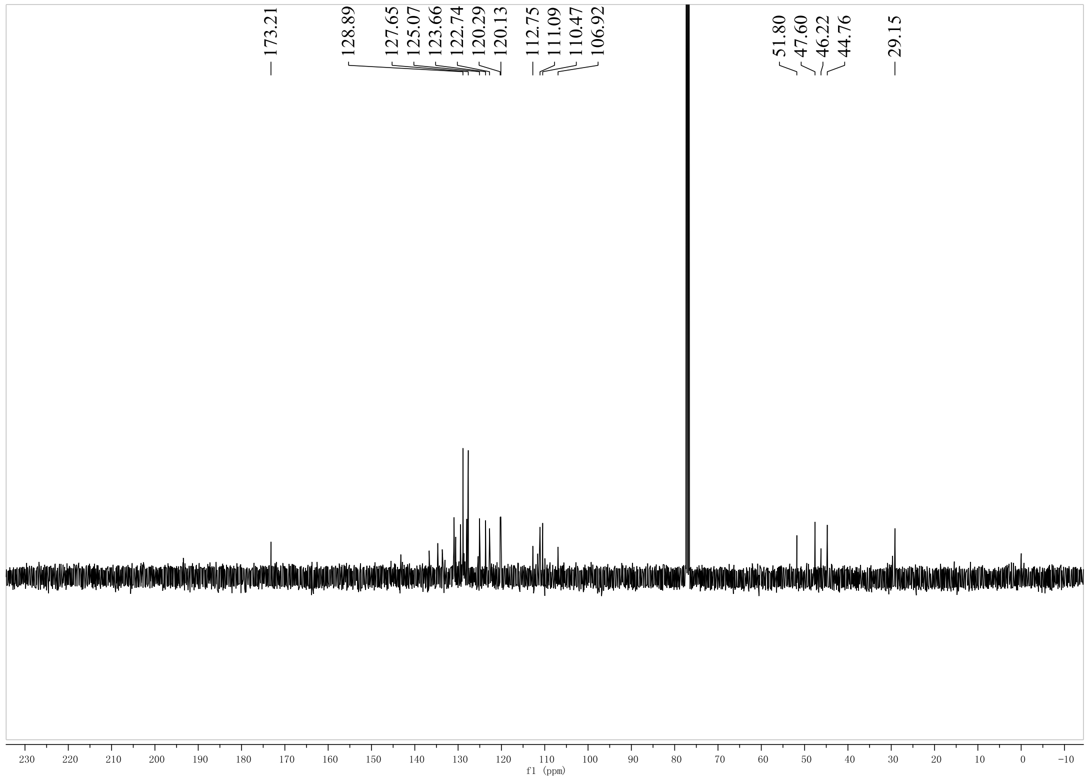
173.21
128.89
127.65
125.07
123.66
122.74
120.29
120.13
112.75
111.09
110.47
106.92
51.80
47.60
46.22
44.76
29.15
230 220 210 200 190 180 170 160 150 140 130 120 110 100 90 80 70 60 50 40 30 20 10 0 −10
f1 (ppm)

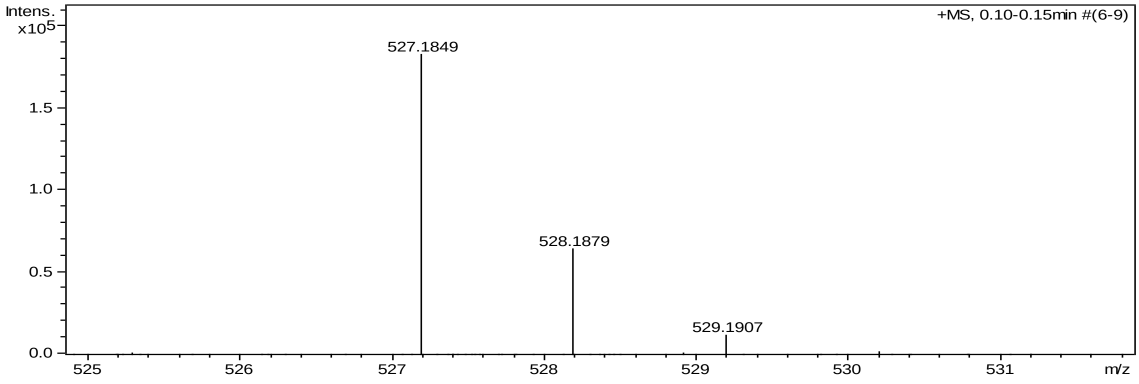
Intens.
x10^5
1.5
1.0
0.5
0.0
+MS, 0.10-0.15min #(6-9)
527.1849
528.1879
529.1907
525 526 527 528 529 530 531 m/z

1'-Benzyl-2'-oxo-4-phenyl-4,9-dihydrospiro[carbazole-2,3'-indoline]-3,3(1*H*)-dicarbonitrile (2a’):

white solid, 7%, m.p. 213-215 °C; ^{1}H NMR (400 MHz, $CDCl_3$) δ: 8.03 (s, 1H, NH), 7.86 (d, J = 7.6 Hz, 1H, ArH), 7.66-7.63 (m, 1H, ArH), 7.55-7.46 (m, 3H, ArH), 7.37-7.33 (m, 5H, ArH), 7.31-7.28 (m, 2H, ArH), 7.19 (t, J = 7.6 Hz, 1H, ArH), 7.11 (t, J = 7.6 Hz, 1H, ArH), 6.86 (d, J = 7.6 Hz, 2H, ArH), 6.46 (d, J = 8.0 Hz, 1H, ArH), 5.51 (s, 1H, CH), 5.03 (d, J =15.6 Hz, 1H, CH), 4.90 (d, J =15.6 Hz, 1H, CH), 3.85 (dd, J_1 = 17.6 Hz, J_2 = 2.0 Hz, 1H, CH), 3.15 (d, J =17.6 Hz, 1H, CH); ^{13}C NMR (400 MHz, $CDCl_3$) δ: 172.6, 142.5, 136.5, 134.7, 134.6, 130.8, 129.2, 129.1, 128.9, 128.6, 127.9, 127.3, 126.6, 125.7, 124.5, 123.9, 122.2, 120.3, 119.7, 113.0, 112.9, 110.8, 110.1, 107.7, 50.6, 47.7, 45.7, 44.4, 29.3; IR (KBr) υ: 3300, 3267, 3178, 3043, 2965, 2848, 2164, 1853, 1666, 1607, 1541, 1456, 1355, 1251, 1167, 1121, 969, 955, 870, 768 cm^{-1}; MS (m/z): HRMS (ESI) Calcd. for $C_{34}H_{24}N_4O$ ([M+Na]$^+$): 527.1842, found: 527.1846.

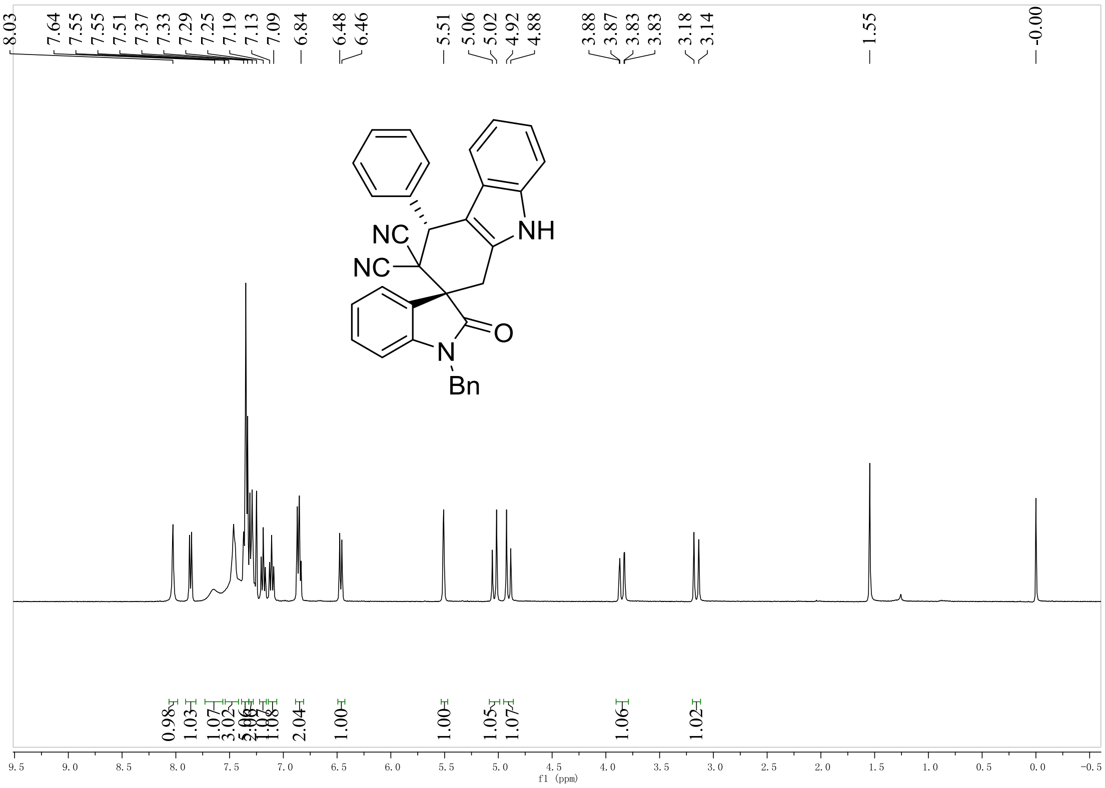

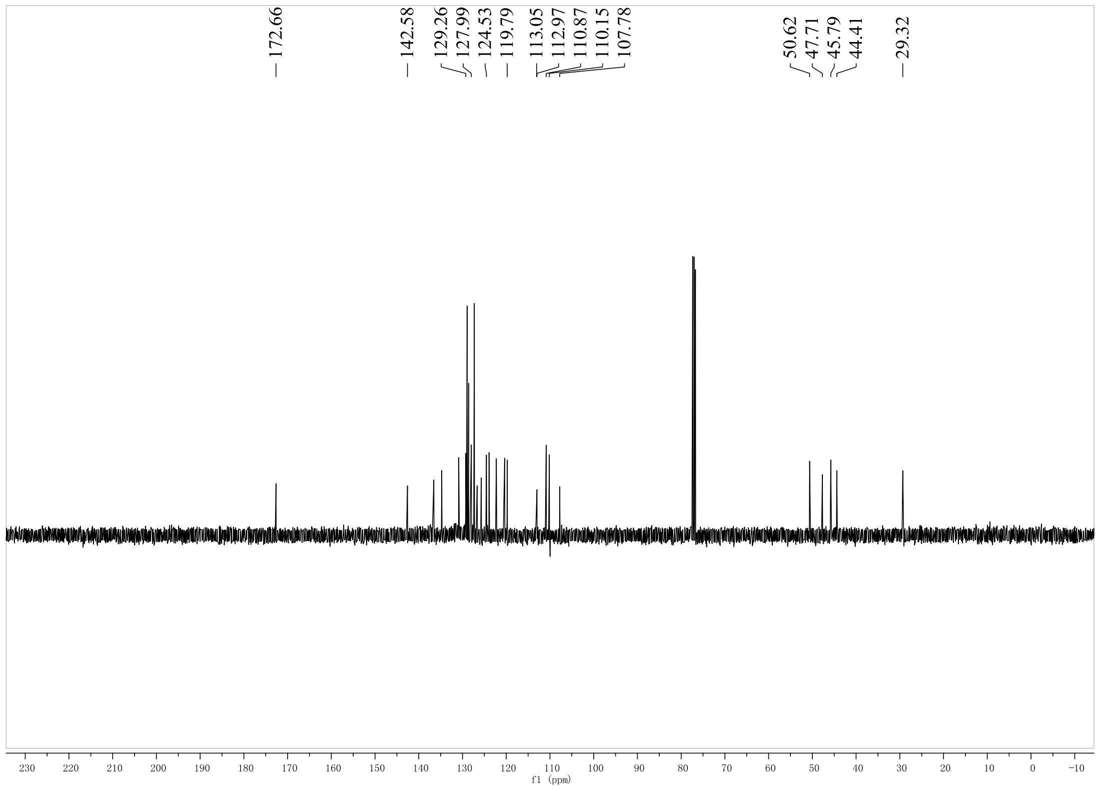
172.66
142.58
129.26
127.99
124.53
119.79
113.05
112.97
110.87
110.15
107.78
50.62
47.71
45.79
44.41
29.32
230 220 210 200 190 180 170 160 150 140 130 120 110 100 90 80 70 60 50 40 30 20 10 0 −10
f1 (ppm)

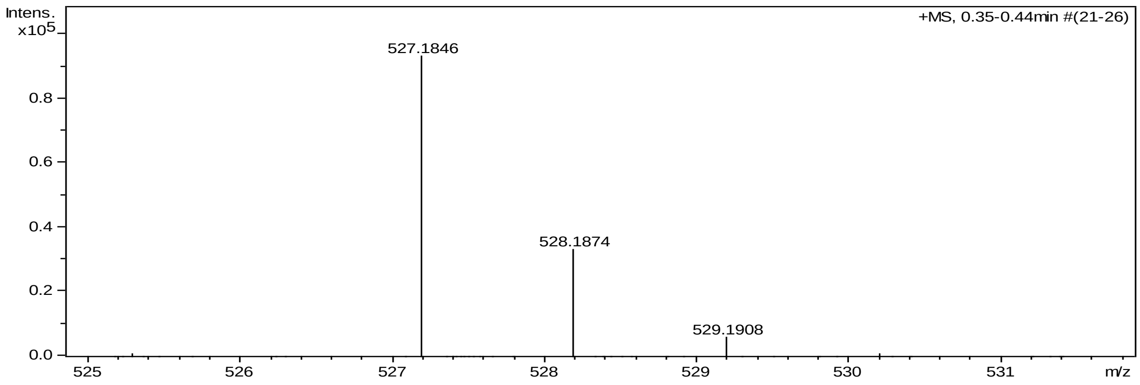
Intens.
x10^5
+MS, 0.35-0.44min #(21-26)
527.1846
528.1874
529.1908
0.0
0.2
0.4
0.6
0.8
525 526 527 528 529 530 531 m/z

1'-Benzyl-2'-oxo-4-(*p*-tolyl)-4,9-dihydrospiro[carbazole-2,3'-indoline]-3,3(1*H*)-dicarbonitrile (2b):

white solid, 62%, m.p. 195-198 ºC; ^{1}H NMR (400 MHz, $CDCl_3$) δ: 8.04 (s, 1H, NH), 7.85 (d, J = 7.6 Hz, 1H, ArH), 7.55-7.52 (m, 1H, ArH), 7.37-7.27 (m, 8H, ArH), 7.19 (t, J = 7.6 Hz, 2H, ArH), 7.11 (t, J = 7.2 Hz, 1H, ArH), 6.87 (t, J = 8.4 Hz, 2H, ArH), 6.51 (d, J = 8.0 Hz, 1H, ArH), 5.47 (s, 1H, CH), 5.03 (d, J =15.6 Hz, 1H, CH), 4.90 (d, J =15.6 Hz, 1H, CH), 3.84 (dd, J_1 = 17.6 Hz, J_2 = 2.0 Hz, 1H, CH), 3.15 (d, J = 17.6 Hz, 1H, CH), 2.41 (s, 3H, CH_3); ^{13}C NMR (400 MHz, $CDCl_3$) δ: 172.6, 142.5, 139.0, 136.5, 134.7, 131.5, 130.7, 129.3, 129.0, 128.9, 128.8, 127.9, 127.3, 126.7, 125.8, 124.5, 123.9, 122.2, 120.4, 119.7, 113.1, 110.7, 110.1, 108.0, 50.6, 47.8, 45.4, 44.4, 29.3, 21.3; IR (KBr) υ: 3367, 3310, 3245, 3087, 2956, 2833, 2155, 1860, 1671, 1655, 1543, 1445, 1361, 1267, 1158, 1141, 955, 901, 868, 783 cm^{-1}; MS (m/z): HRMS (ESI) Calcd. for $C_{35}H_{16}N_4O$ ($[M+Na]^+$): 541.1999, found: 541.2001.

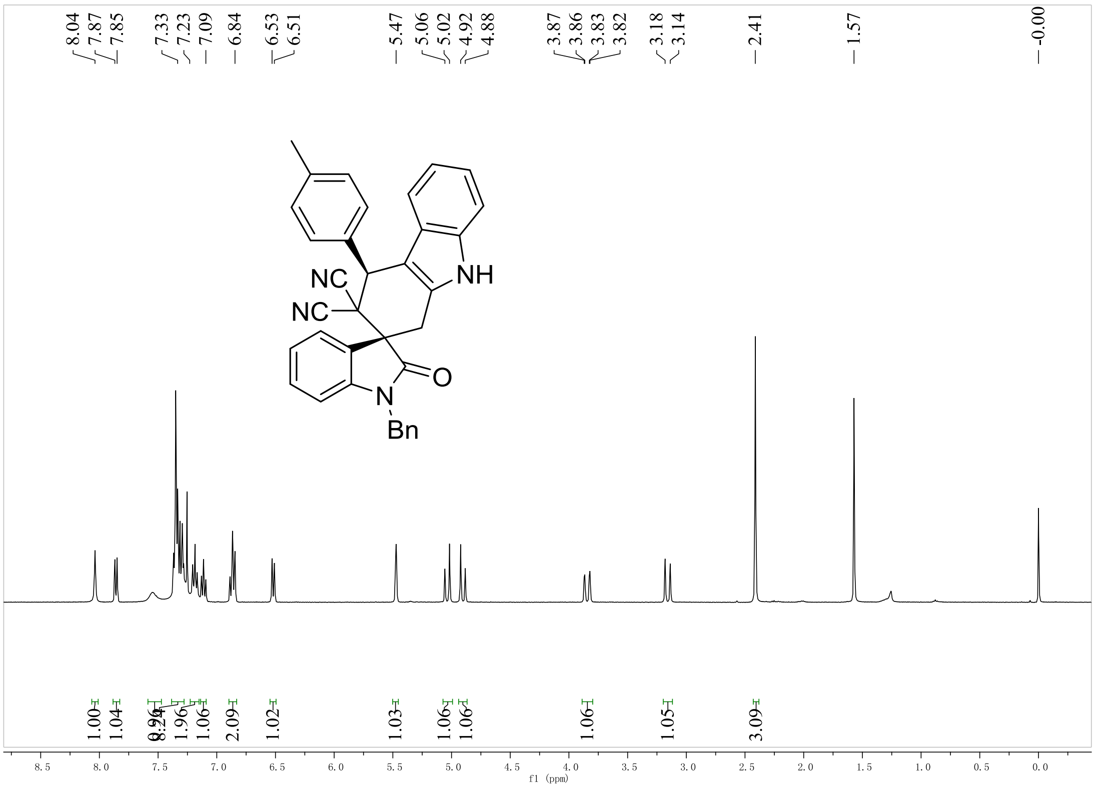

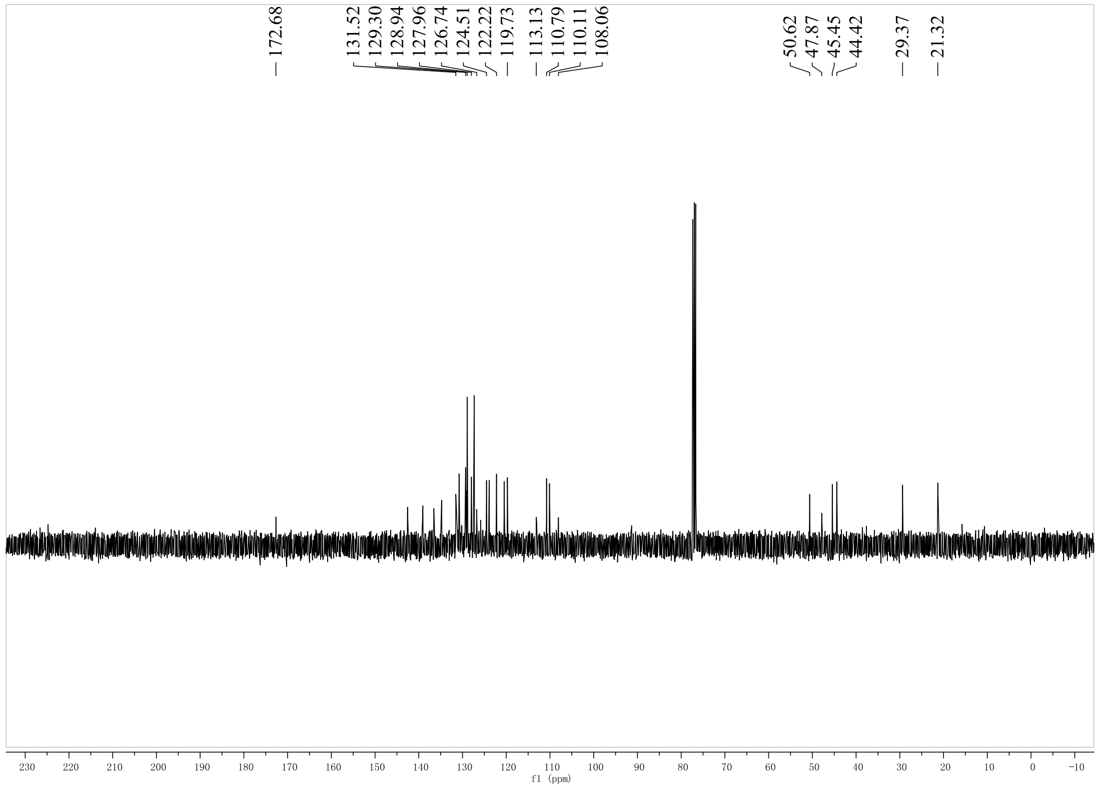
172.68
131.52
129.30
128.94
127.96
126.74
124.51
122.22
119.73
113.13
110.79
110.11
108.06
50.62
47.87
45.45
44.42
29.37
21.32
f1 (ppm)

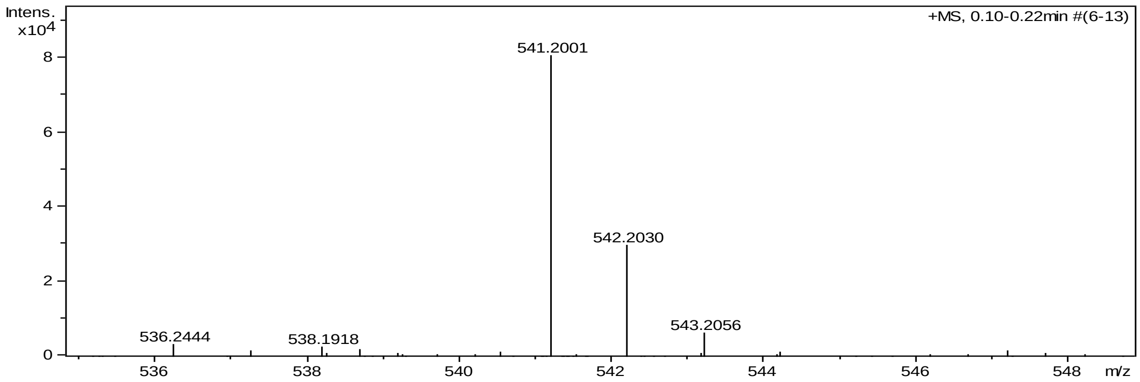
Intens.
x10^4
+MS, 0.10-0.22min #(6-13)
541.2001
542.2030
543.2056
536.2444
538.1918
m/z

1'-Benzyl-2'-oxo-4-(*p*-tolyl)-4,9-dihydrospiro[carbazole-2,3'-indoline]-3,3(1*H*)-dicarbonitrile (2b'):

white solid, 10%, m.p. 205-208 ºC; ^{1}H NMR (400 MHz, $CDCl_3$) δ: 8.00 (s, 1H, NH), 7.86 (d, *J* = 7.6 Hz, 1H, ArH), 7.55-7.52 (m, 1H, ArH), 7.37-7.33 (m, 5H, ArH), 7.31-7.27 (m, 3H, ArH), 7.27-7.25 (m, 1H, ArH), 7.19 (t, *J* = 7.6 Hz, 2H, ArH), 7.11 (t, *J* = 8.0 Hz, 1H, ArH), 6.87 (t, *J* = 8.8 Hz, 2H, ArH), 6.52 (d, *J* = 8.0 Hz, 1H, ArH), 5.47 (s, 1H, CH), 5.03 (d, *J* =15.6 Hz, 1H, CH), 4.90 (d, *J* =16.0 Hz, 1H, CH), 3.84 (dd, J_1 = 17.6 Hz, J_2 = 2.0 Hz, 1H, CH), 3.15 (d, *J* = 17.6 Hz, 1H, CH), 2.41 (s, 3H, CH_3); ^{13}C NMR (400 MHz, $CDCl_3$) δ: 172.6, 142.5, 139.0, 136.5, 134.7, 131.5, 130.7, 129.2, 129.0, 128.9, 128.9, 127.9, 127.3, 126.7, 125.8, 124.4, 123.8, 122.2, 120.4, 119.7, 113.1, 112.9, 110.7, 110.1, 108.0, 50.6, 47.8, 45.4, 44.4, 29.3, 21.3; IR (KBr) υ: 3343, 3245, 3171, 3067, 2955, 2848, 2161, 1843, 1643, 1618, 1565, 1455, 1348, 1231, 1155, 1131, 984, 922, 862, 755 cm^{-1}; MS (*m*/*z*): HRMS (ESI) Calcd. for $C_{35}H_{16}N_4O$ ([M+Na]$^+$): 541.1999, found: 541.1997.

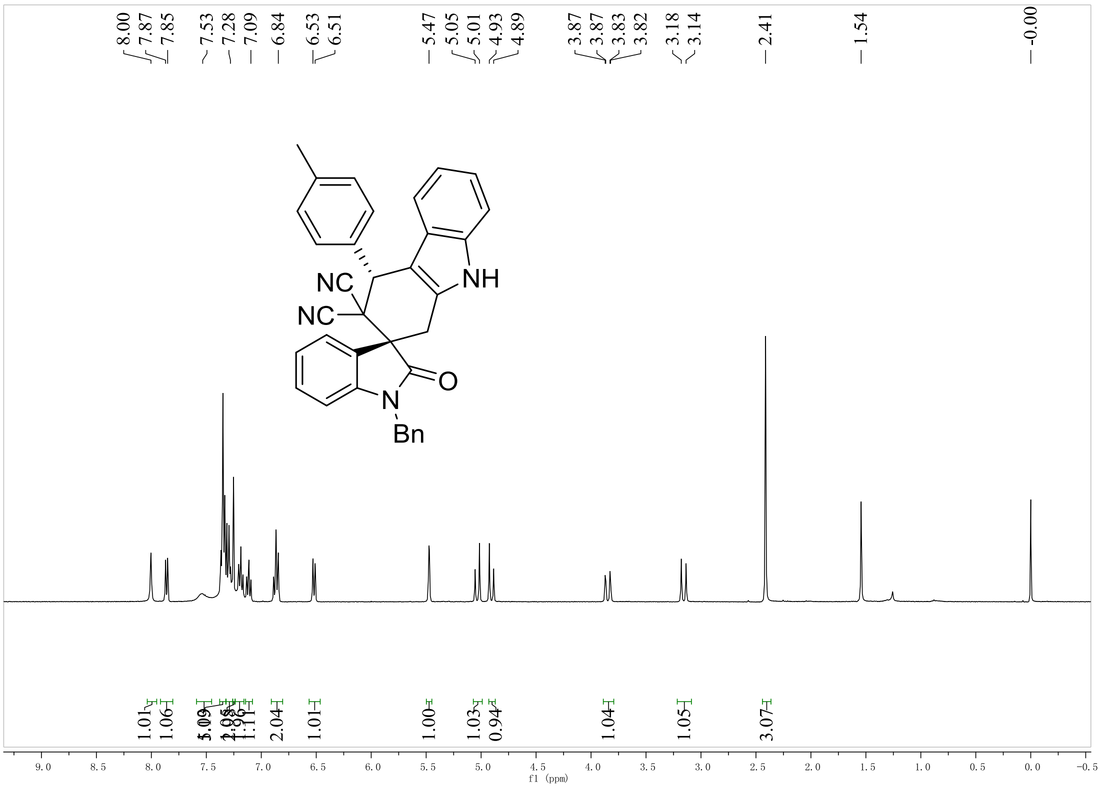

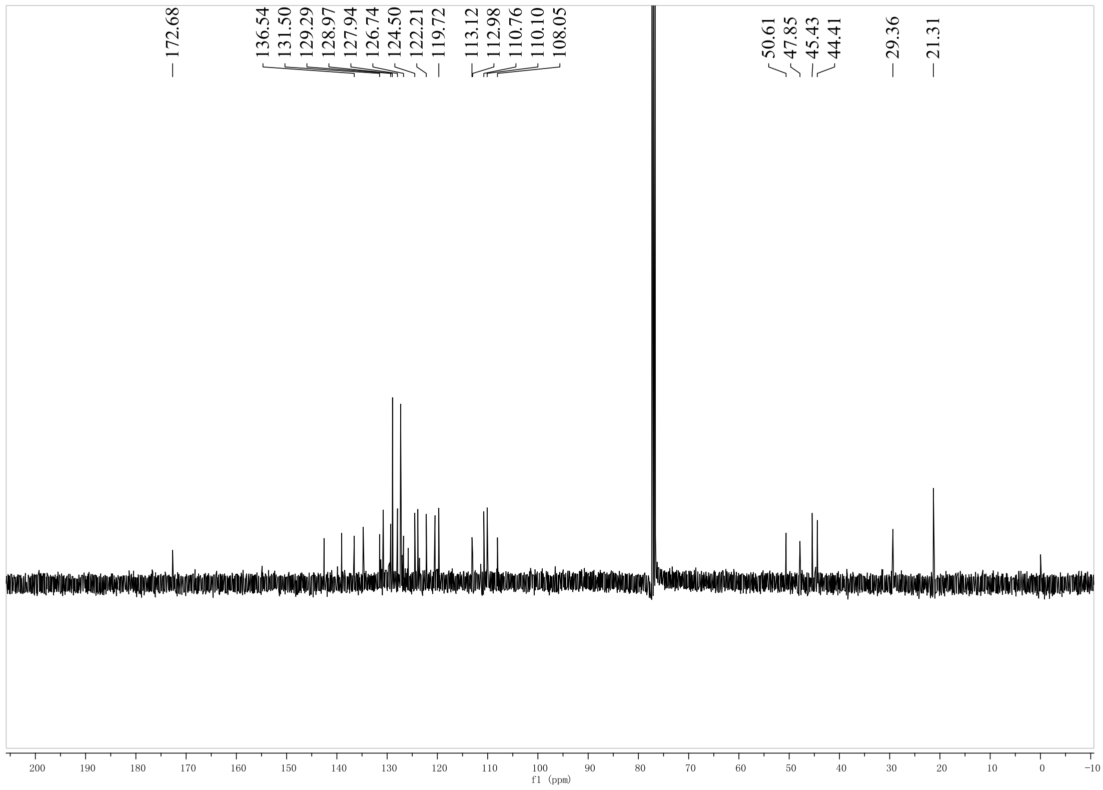
172.68
136.54
131.50
129.29
128.97
127.94
126.74
124.50
122.21
119.72
113.12
112.98
110.76
110.10
108.05
50.61
47.85
45.43
44.41
29.36
21.31
f1 (ppm)

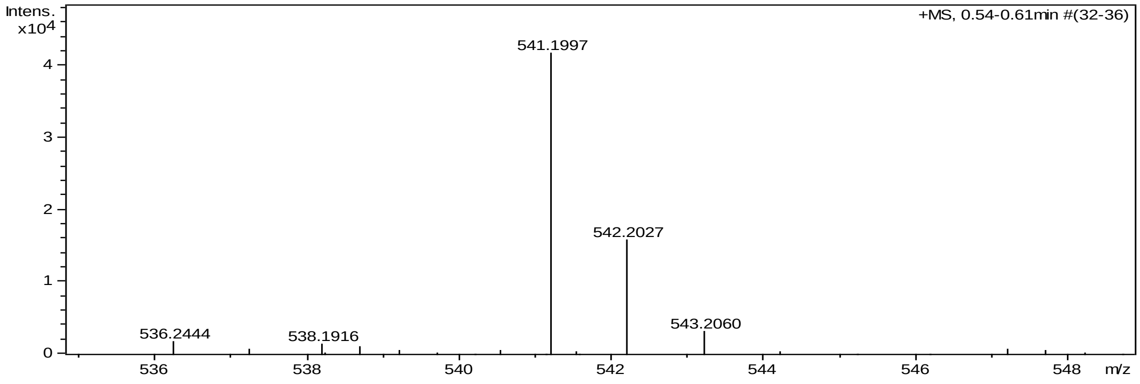
Intens.
x10^4
+MS, 0.54-0.61min #(32-36)
541.1997
542.2027
543.2060
536.2444
538.1916
m/z

1'-Benzyl-4-(4-fluorophenyl)-2'-oxo-4,9-dihydrospiro[carbazole-2,3'-indoline]-3,3(1*H*)-dicarbonitrile (2c):

white solid, 56%, m.p. 201-204 °C; ^{1}H NMR (400 MHz, $CDCl_3$) δ: 8.28 (s, 1H, NH), 7.52-7.50 (m, 1H, ArH), 7.41 (t, *J* = 7.6 Hz, 3H, ArH), 7.36-7.29 (m, 4H, ArH), 7.22 (t, *J* = 8.0 Hz, 1H, ArH), 7.15-7.14 (m, 1H, ArH), 7.06-7.04 (m, 1H, ArH), 6.96 (t, *J* = 8.0 Hz, 1H, ArH), 6.92-6.88 (m, 3H, ArH), 6.55 (d, *J* = 8.0 Hz, 1H, ArH), 5.14 (d, *J* = 11.6 Hz, 1H, CH), 4.95 (d, *J* = 11.6 Hz, 1H, CH), 4.90 (s, 1H, CH), 4.09 (dd, J_1= 16.8 Hz, J_2= 2.0 Hz, 1H, CH), 2.96 (d, *J* = 16.8 Hz, 1H, CH); ^{13}C NMR (400 MHz, $CDCl_3$) δ: 173.1, 143.2, 136.6, 134.6, 131.0, 130.6, 129.5, 129.4, 128.8, 128.0, 127.6, 125.1, 124.9, 123.6, 122.8, 120.2, 120.0, 112.6, 111.5, 111.2, 110.5, 106.6, 51.7, 46.9, 44.7, 29.6 (d, *J* = 5.0 Hz), 1.0 (d, *J* = 4.0 Hz); IR (KBr) υ: 3451, 3133, 3078, 3001, 2961, 1978, 1755, 1648, 1567, 1519, 1466, 1367, 1314, 1255, 1131, 970, 880, 783 cm^{-1}; MS (*m*/*z*): HRMS (ESI) Calcd. for $C_{34}H_{23}FN_4O$ ($[M+Na]^+$): 545.1748, found: 545.1746.

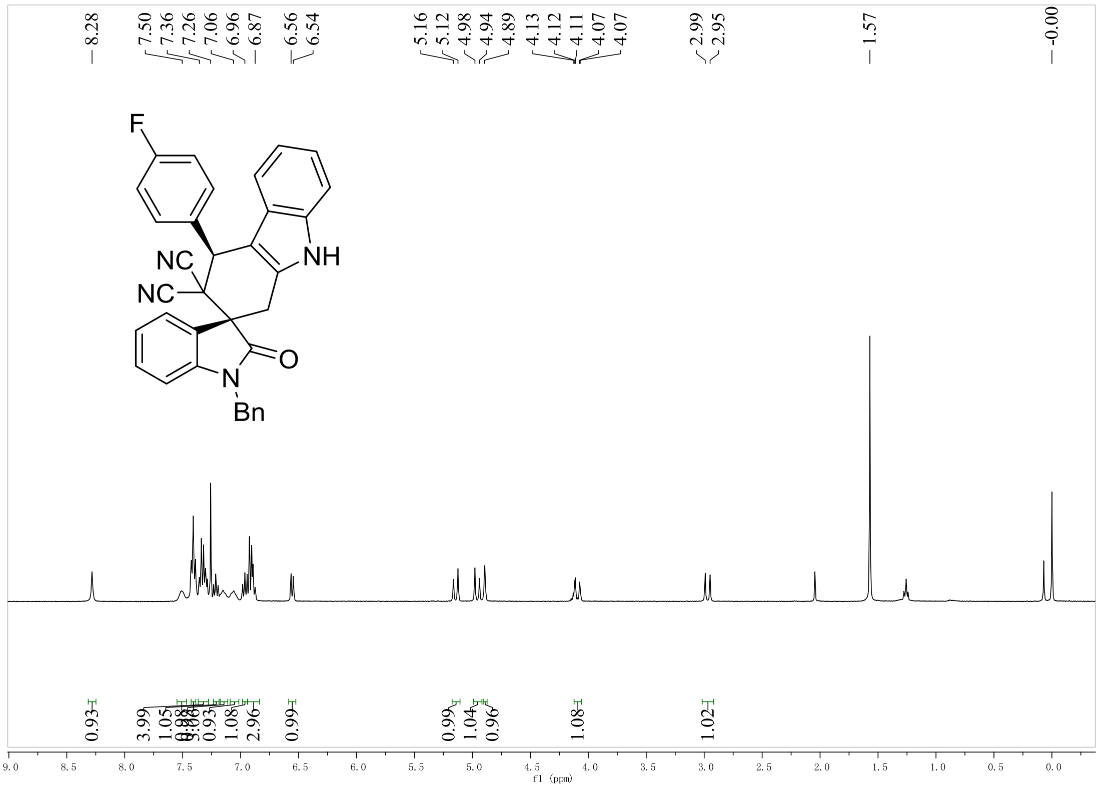

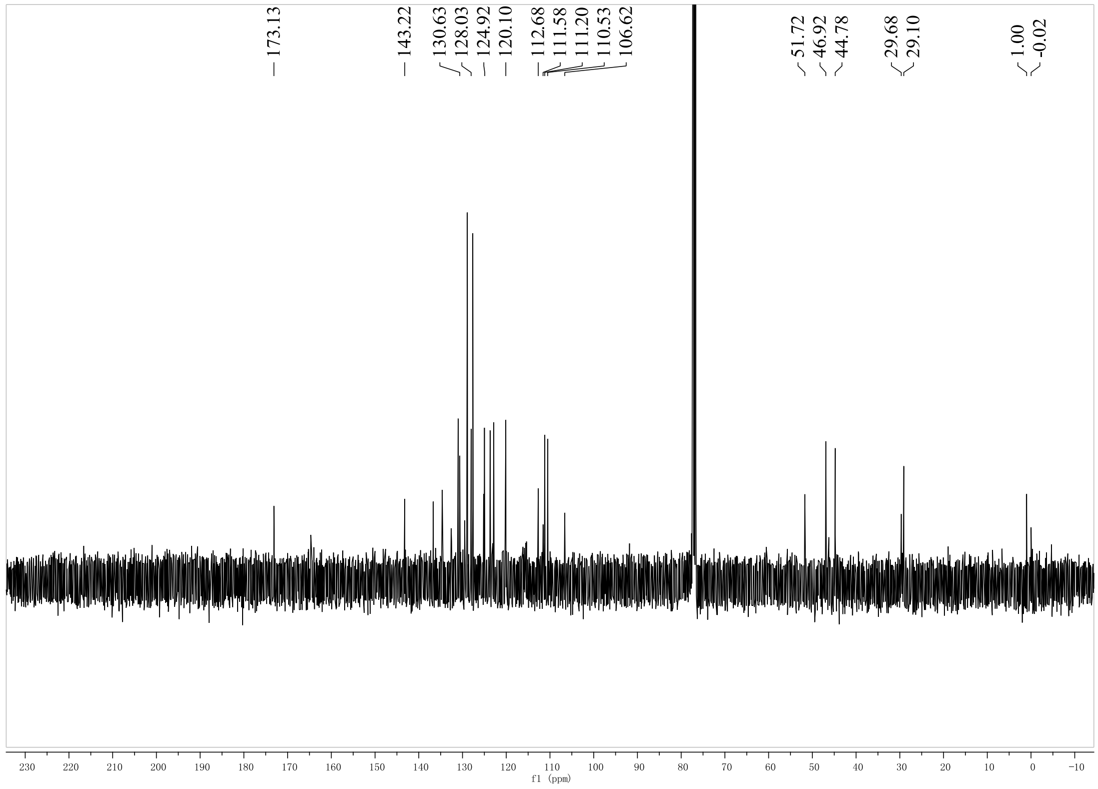
173.13
143.22
130.63
128.03
124.92
120.10
112.68
111.58
111.20
110.53
106.62
51.72
46.92
44.78
29.68
29.10
1.00
-0.02
f1 (ppm)

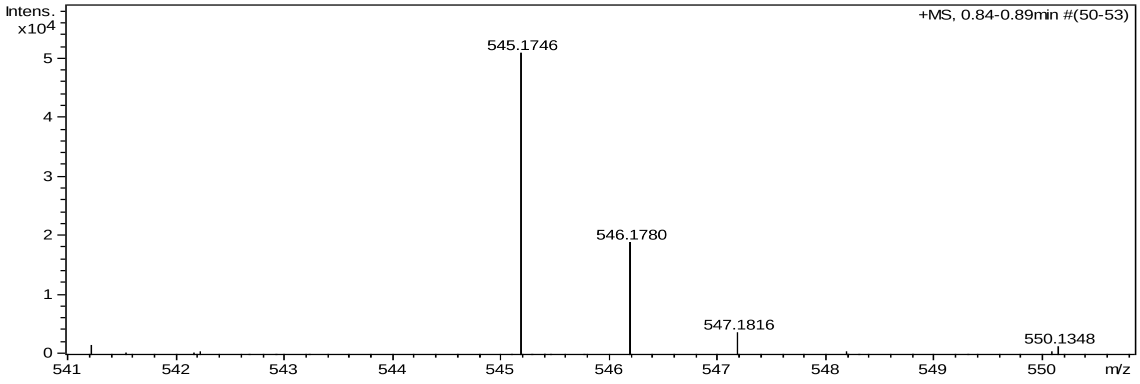
Intens.
x10^4
+MS, 0.84-0.89min #(50-53)
545.1746
546.1780
547.1816
550.1348
m/z

1'-Benzyl-4-(4-fluorophenyl)-2'-oxo-4,9-dihydrospiro[carbazole-2,3'-indoline]-3,3(1*H*)-dicarbonitrile (2c'):

white solid, 8%, m.p. 215-218 ºC; ^{1}H NMR (400 MHz, $CDCl_3$) δ: 8.07 (s, 1H, NH), 7.84 (d, J = 7.6 Hz, 1H, ArH), 7.62-7.45 (m, 2H, ArH), 7.37-7.28 (m, 7H, ArH), 7.19 (t, J = 7.6 Hz, 1H, ArH), 7.11 (t, J = 7.6 Hz, 2H, ArH), 6.90-6.85 (m, 2H, ArH), 6.47 (d, J = 8.0 Hz, 1H, ArH), 5.51 (s, 1H, CH), 5.01 (d, J = 16.0 Hz, 1H, CH), 4.87 (d, J = 16.0 Hz, 1H, CH), 3.81 (dd, J_1 = 17.6 Hz, J_2 = 2.0 Hz, 1H, CH), 3.14 (d, J = 17.6 Hz, 1H, CH); ^{13}C NMR (400 MHz, $CDCl_3$) δ: 172.6, 164.5, 162.1, 142.5, 136.5, 134.6, 130.9, 130.4, 129.2, 128.9, 128.0, 127.2, 126.5, 125.5, 124.4, 124.0, 122.3, 120.1, 119.9, 115.5, 115.5, 112.9, 112.9, 111.0, 110.2, 107.4, 50.5, 47.7, 45.1, 44.4, 29.2; IR (KBr) υ: 3417, 3078, 2978, 1843, 1765, 1678, 1617, 1583, 1457, 1355, 1317, 1248, 1131, 970, 861, 788 cm^{-1}; MS (m/z): HRMS (ESI) Calcd. for $C_{34}H_{23}FN_4O$ ($[M+Na]^+$): 545.1748, found: 545.1745.

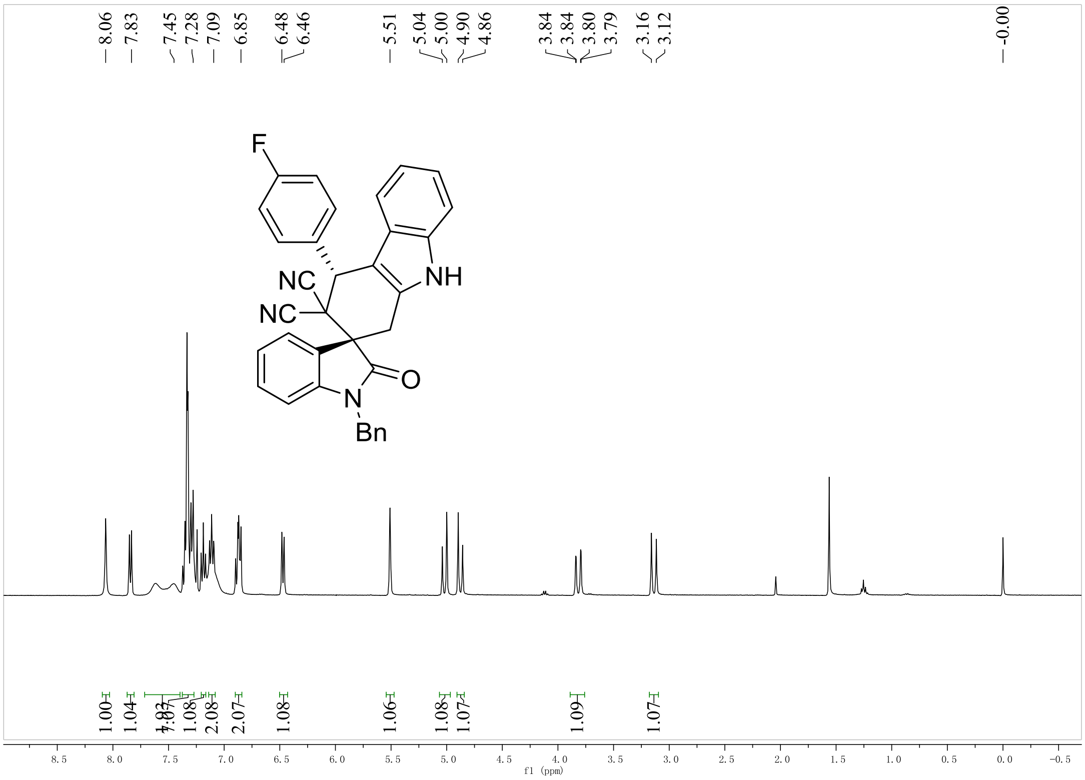

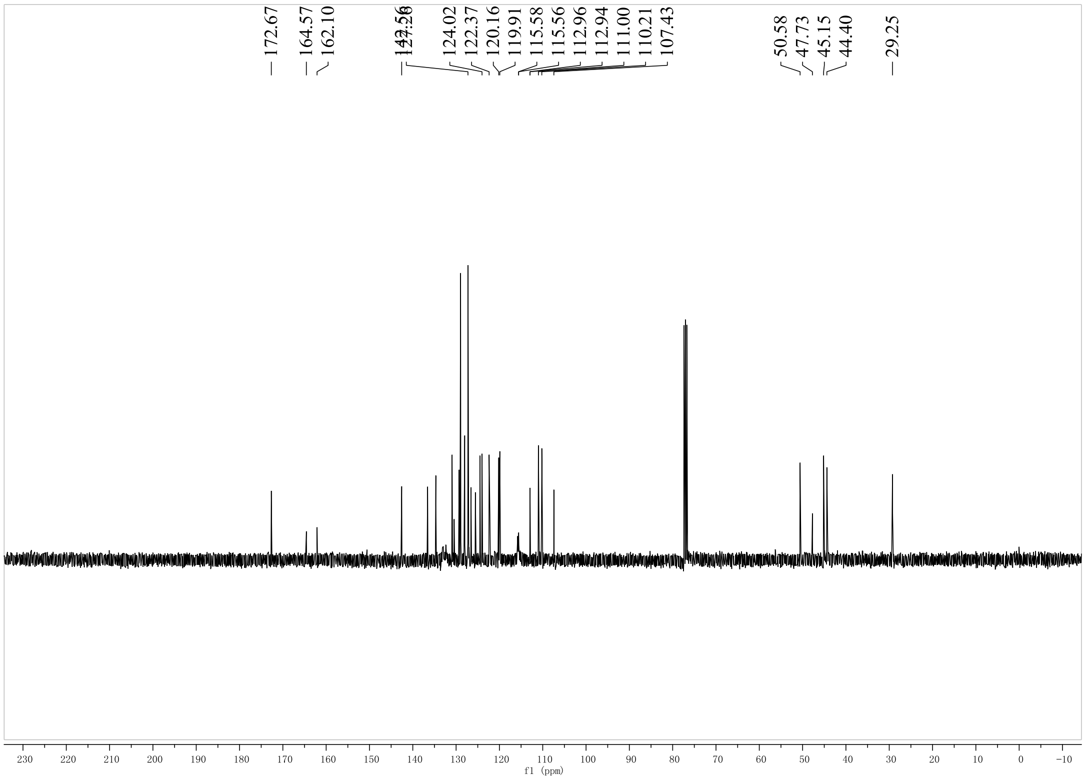
172.67
164.57
162.10
124.02
122.37
120.16
119.91
115.58
115.56
112.96
112.94
111.00
110.21
107.43
50.58
47.73
45.15
44.40
29.25
f1 (ppm)

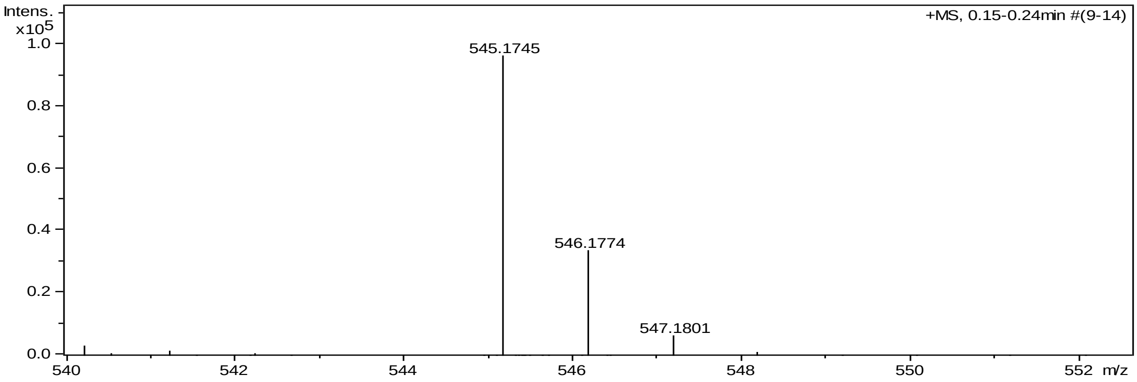
Intens.
x10^5
+MS, 0.15-0.24min #(9-14)
545.1745
546.1774
547.1801
m/z

1'-Benzyl-4-(4-nitrophenyl)-2'-oxo-4,9-dihydrospiro[carbazole-2,3'-indoline]-3,3(1*H*)-dicarbonitrile (2d):

White solid, 56%, m.p. 191-194 ºC; ^{1}H NMR (400 MHz, $CDCl_3$) δ: 8.63 (s, 1H, NH), 8.25 (dd, J_1 = 54.0 Hz, J_2 = 5.2 Hz, 2H, ArH), 7.64 (dd, J_1 = 54.0 Hz, J_2 = 4.0 Hz, 2H, ArH), 7.42-7.38 (m, 3H, ArH), 7.32-7.27 (m, 4H, ArH), 7.21 (t, J = 8.0 Hz, 1H, ArH), 6.97-6.92 (m, 3H, ArH), 6.87 (d, J = 8.0 Hz, 1H, ArH), 6.45 (d, J = 8.0 Hz, 1H, ArH), 5.13 (d, J = 15.6 Hz, 1H, CH), 5.02 (s, 1H, CH), 4.93 (d, J = 15.6 Hz, 1H, CH), 4.08 (d, J = 17.2 Hz, 1H, CH), 3.04 (d, J = 16.8 Hz, 1H, CH); ^{13}C NMR (400 MHz, $CDCl_3$) δ: 172.9, 148.7, 143.2, 141.1, 136.7, 134.5, 131.8, 131.2, 131.0, 128.9, 128.0, 127.6, 124.8, 124.7, 124.5, 123.8, 123.7, 123.1, 120.5, 119.5, 112.3, 111.5, 111.2, 110.6, 109.9, 105.2, 51.6, 47.2, 45.7, 44.8, 29.6; IR (KBr) υ: 3356, 3300, 3249, 3154, 3048, 2963, 1847, 1763, 1632, 1607, 1548, 1467, 1332, 1310, 1249, 1137, 959, 843 cm^{-1}; MS (*m*/*z*): HRMS (ESI) Calcd. for $C_{32}H_{29}NO_4$ ($[M+Na]^+$): 572.1693, found: 572.1690.

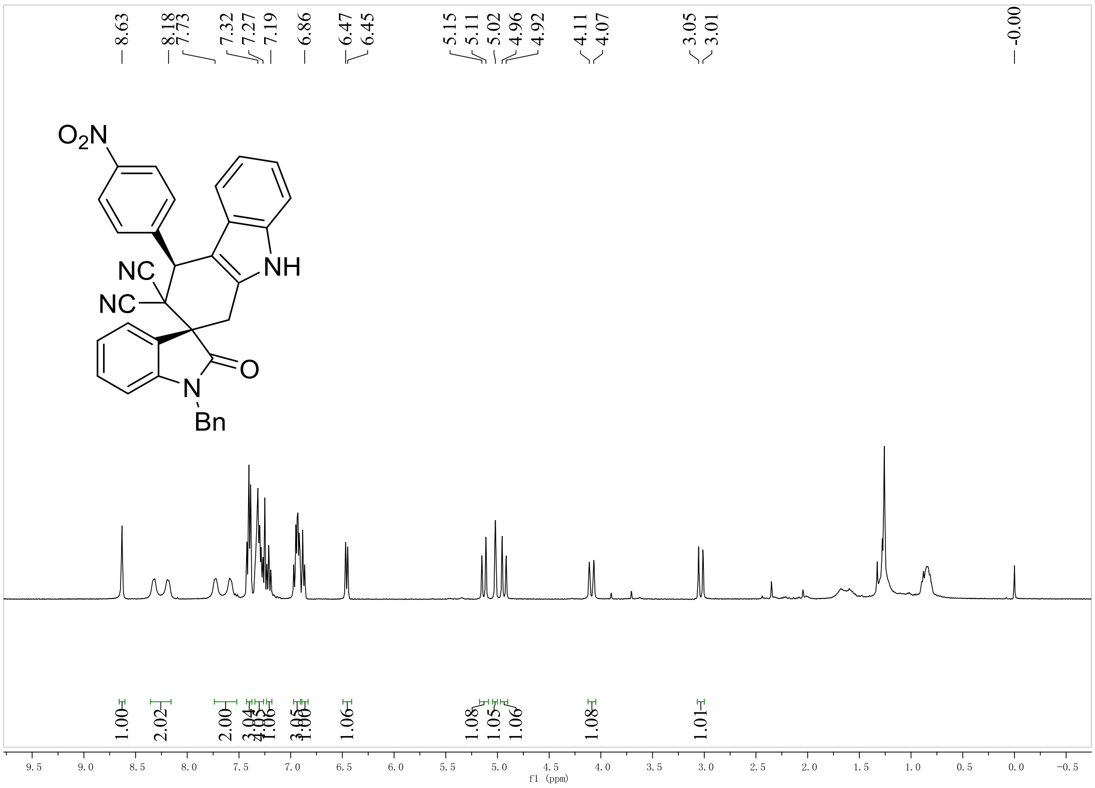

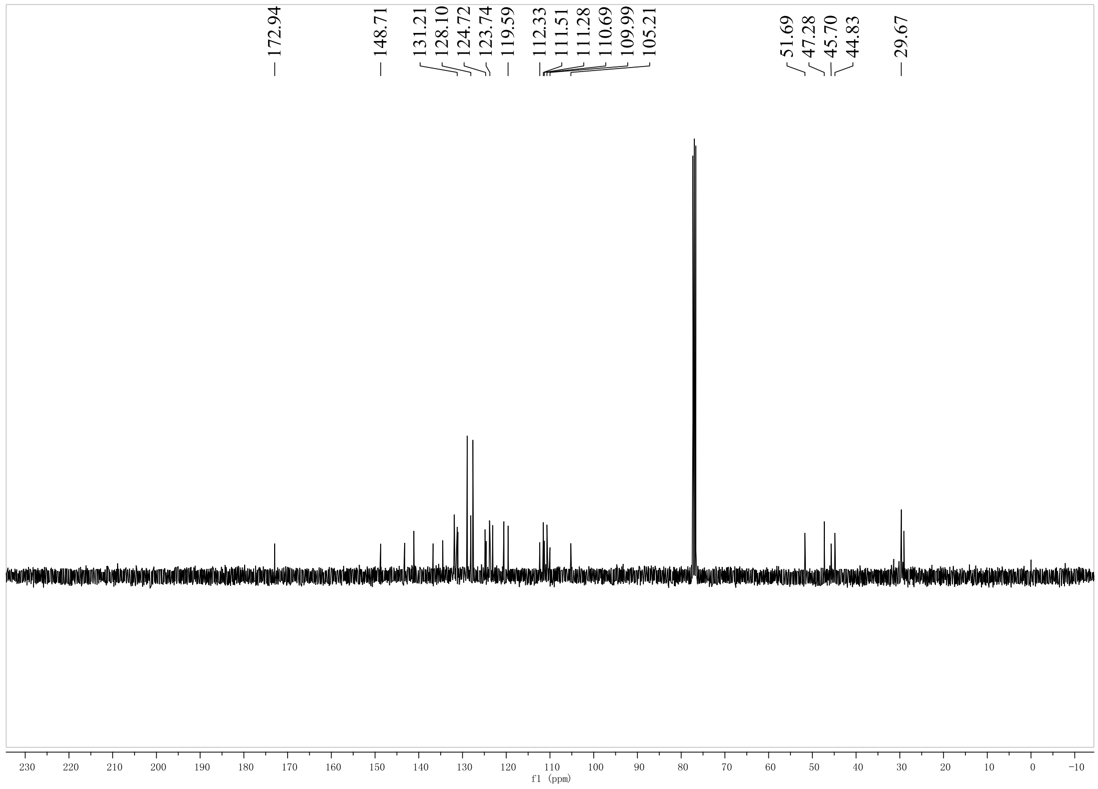

172.94
148.71
131.21
128.10
124.72
123.74
119.59
112.33
111.51
111.28
110.69
109.99
105.21
51.69
47.28
45.70
44.83
29.67
f1 (ppm)

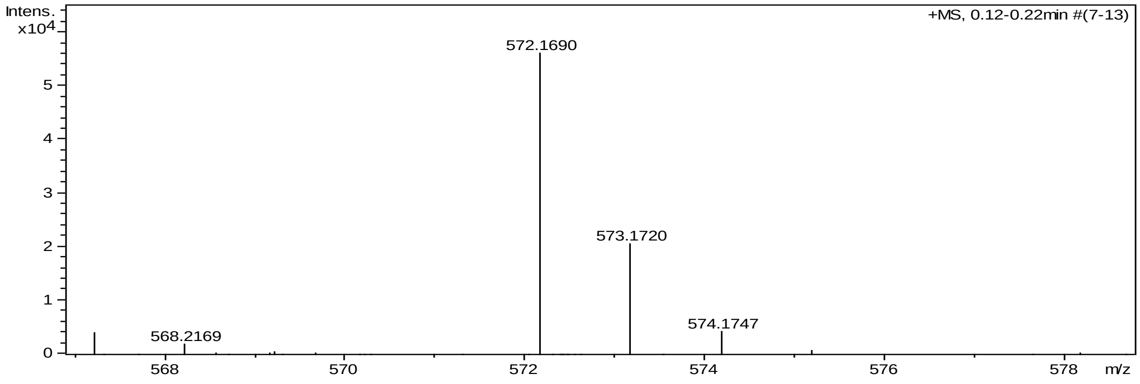

Intens.
x10^4
+MS, 0.12-0.22min #(7-13)
572.1690
573.1720
574.1747
568.2169
m/z

1'-Benzyl-4-(4-nitrophenyl)-2'-oxo-4,9-dihydrospiro[carbazole-2,3'-indoline]-3,3(1*H*)-dicarbonitrile (2d'):

White solid, 7%, m.p. 200-203 ºC; ^{1}H NMR (400 MHz, $CDCl_3$) δ: 8.12 (s, 1H, NH), 7.84 (d, *J* = 7.6 Hz, 2H, ArH), 7.41-7.37 (m, 1H, ArH), 7.36-7.35 (m, 4H, ArH), 7.16 (t, *J* = 7.2 Hz, 1H, ArH), 6.92-6.88 (m, 2H, ArH), 6.39 (d, *J* = 8.0 Hz, 1H, ArH), 5.66 (s, 1H, CH), 5.04 (d, *J* = 15.6 Hz, 1H, CH), 4.92 (d, *J* = 15.6 Hz, 1H, CH), 3.87 (dd, J_1= 17.6 Hz, J_2= 2.0 Hz, 1H, CH), 3.22 (d, *J* = 17.6 Hz, 1H, CH); ^{13}C NMR (400 MHz, $CDCl_3$) δ: 172.3, 148.5, 142.5, 142.0, 136.5, 134.5, 131.1, 129.5, 128.9, 128.0, 127.2, 126.2, 125.1, 124.4, 124.1, 123.7, 122.6, 120.2, 119.7, 112.4, 111.1, 110.3, 106.3, 50.4, 47.0, 45.5, 44.4, 29.2; IR (KBr) υ: 3418, 3240, 3179, 3054, 3004, 2976, 1857, 1767, 1648, 1638, 1564, 1433, 1378, 1334, 1258, 1165, 944, 831, 767 cm^{-1}; MS (*m*/*z*): HRMS (ESI) Calcd. for $C_{32}H_{29}NO_4$ ($[M+Na]^+$): 572.1693, found: 572.1692.

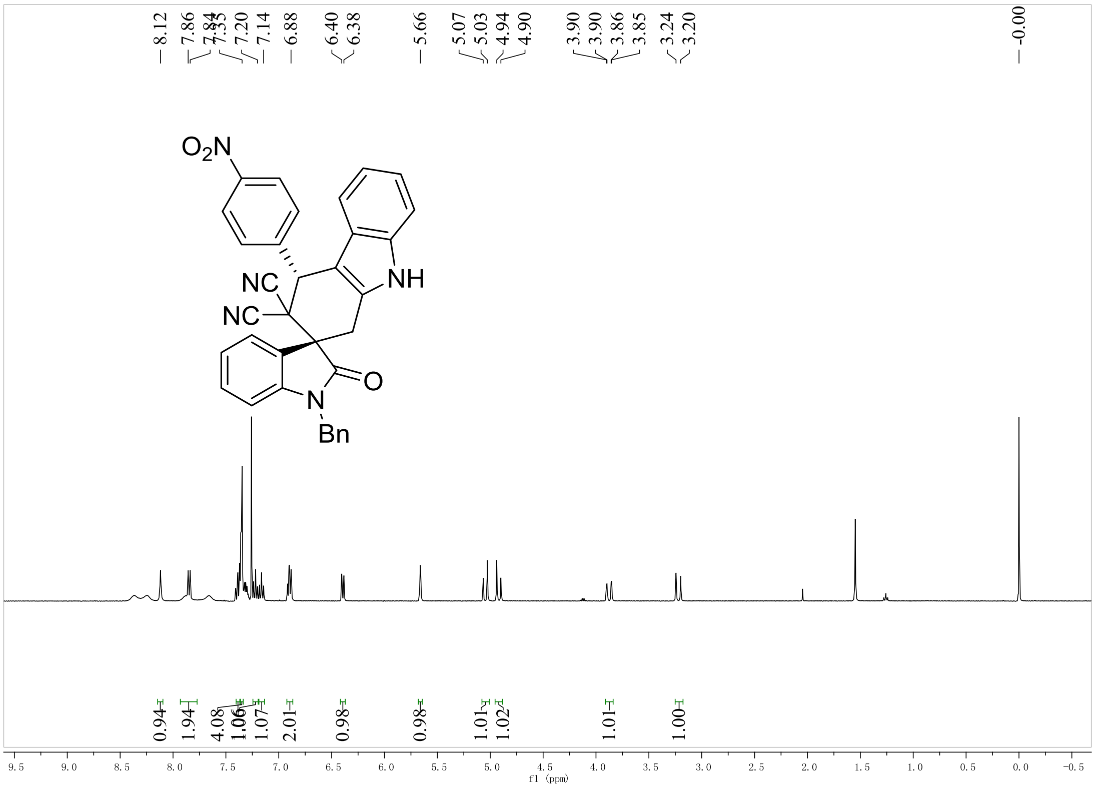

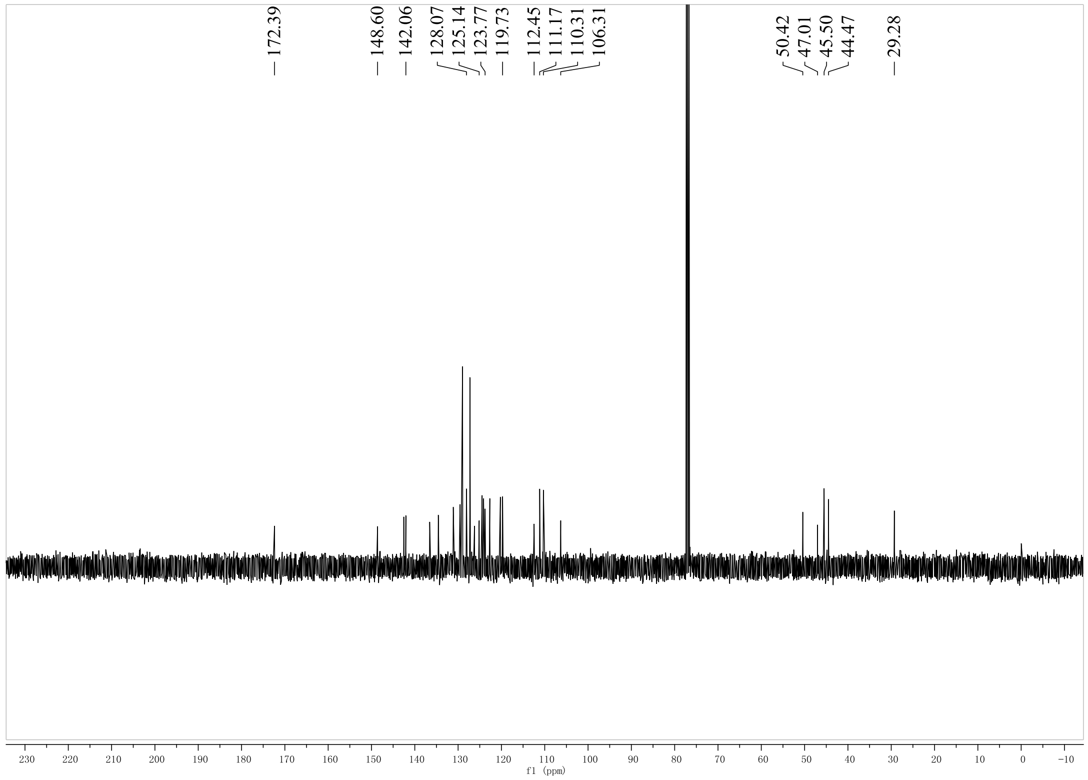

172.39
148.60
142.06
128.07
125.14
123.77
119.73
112.45
111.17
110.31
106.31
50.42
47.01
45.50
44.47
29.28
f1 (ppm)

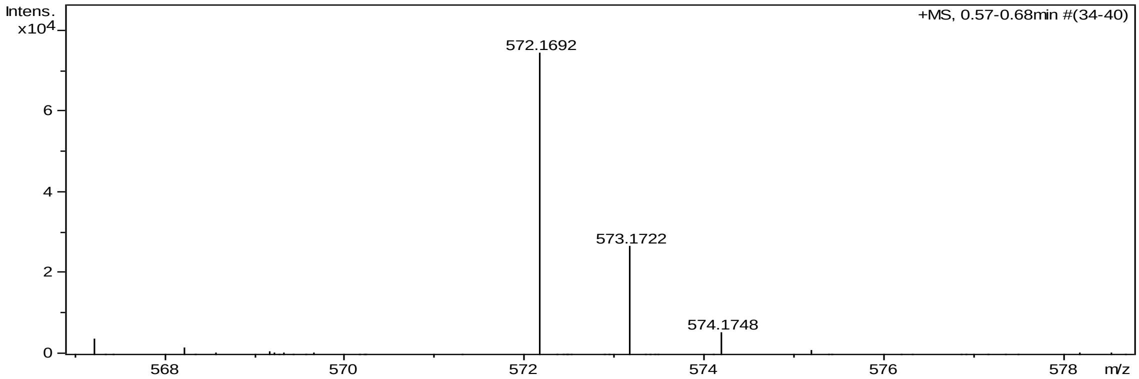

Intens.
x10^4
+MS, 0.57-0.68min #(34-40)
572.1692
573.1722
574.1748
m/z

3-((2-Azanylidene)-3-methyl)-1'-benzyl-2'-oxo-4-(*o*-tolyl)-1,3,4,9-tetrahydrospiro[carbazole-2,3'-indoline]-3-carbonitrile (2e):

white solid, 57%, m.p. 187-190 ºC; ^{1}H NMR (400 MHz, $CDCl_3$) δ: 8.32 (s, 1H, NH), 7.49 (d, *J* = 8.0 Hz, 1H, ArH), 7.41 (d, *J* = 6.8 Hz, 2H, ArH), 7.37-7.34 (m, 2H, ArH), 7.33-7.25 (m, 5H, ArH), 7.20-7.16 (m, 2H, ArH), 7.01 (d, *J* = 7.2 Hz, 1H, ArH), 6.96 (d, *J* = 7.6 Hz, 1H, ArH), 6.93-6.88 (m, 2H, ArH), 6.32 (d, *J* = 8.0 Hz, 1H, ArH), 5.47 (s, 1H, CH), 5.14 (d, *J* = 15.6 Hz, 1H, CH), 4.93 (d, *J* = 15.6 Hz, 1H, CH), 4.12 (dd, J_1 = 16.8 Hz, J_2 = 2.0 Hz, 1H, CH), 2.98 (d, *J* = 16.8 Hz, 1H, CH), 2.43 (s, 3H, CH_3); ^{13}C NMR (400 MHz, $CDCl_3$) δ: 173.2, 143.3, 137.4, 136.7, 134.6, 132.0, 131.3, 130.9, 130.9, 130.2, 129.0, 128.8, 128.0, 127.6, 126.4, 125.4, 125.1, 125.0, 123.6, 122.6, 120.0, 119.8, 112.9, 112.0, 111.1, 110.5, 107.9, 52.2, 44.8, 44.8, 41.5, 29.1, 19.9; IR (KBr) υ: 3300, 3248, 3167, 2932, 2847, 2166, 1841, 1638, 1628, 1531, 1466, 1354, 1218, 1168, 1131, 955, 834, 789 cm^{-1}; MS (*m/z*): HRMS (ESI) Calcd. for MS (*m/z*): HRMS (ESI) Calcd. for $C_{35}H_{16}N_4O$ ($[M+Na]^+$): 541.1999, found: 541.1989.

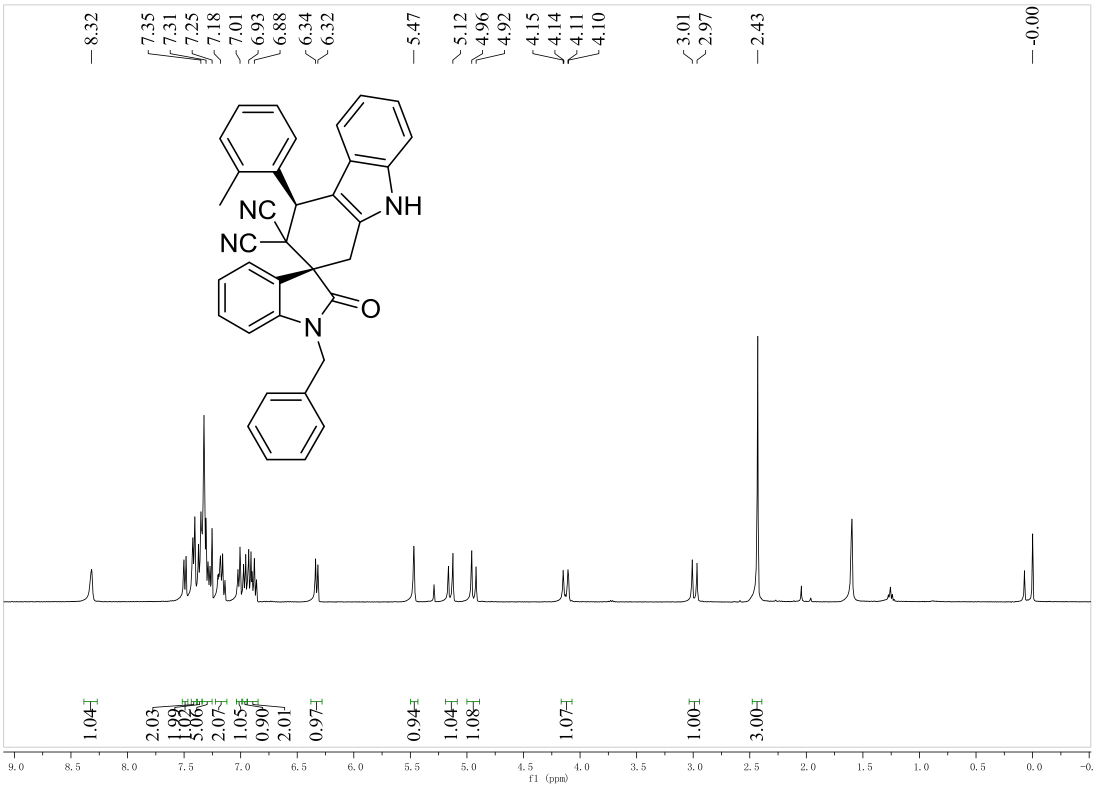

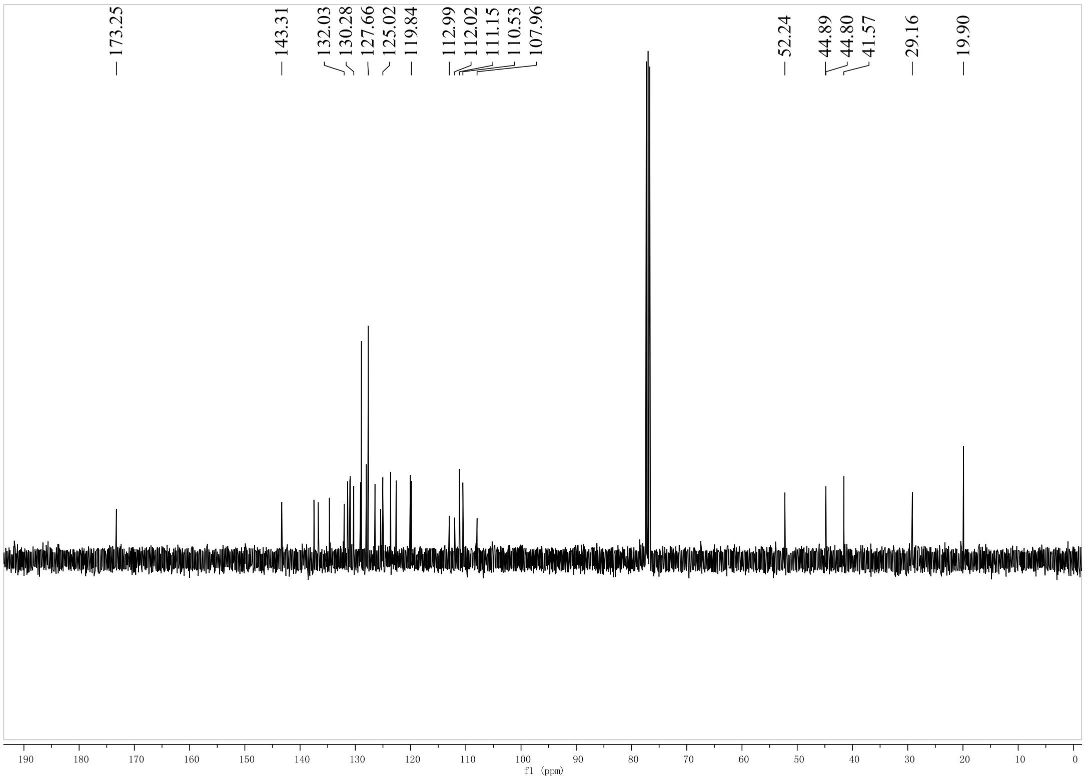
173.25
143.31
132.03
130.28
127.66
125.02
119.84
112.99
112.02
111.15
110.53
107.96
52.24
44.89
44.80
41.57
29.16
19.90
190 180 170 160 150 140 130 120 110 100 90 80 70 60 50 40 30 20 10 0
f1 (ppm)

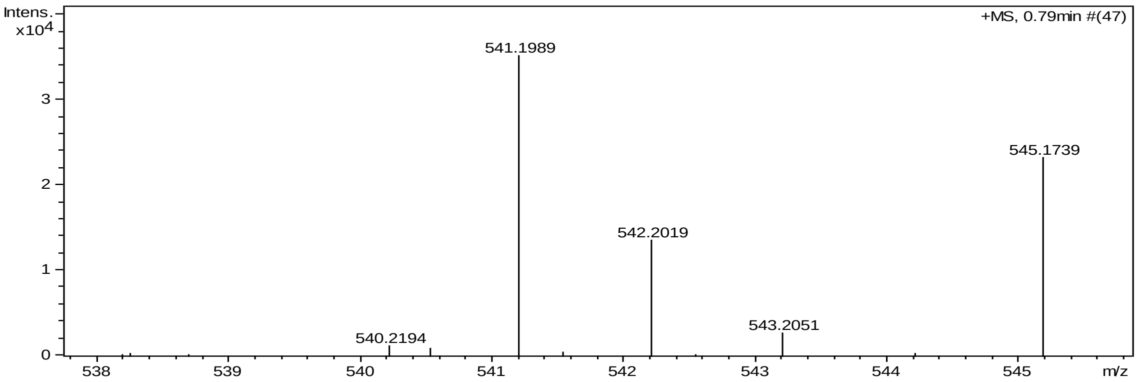
Intens.
x10^4
+MS, 0.79min #(47)
541.1989
545.1739
542.2019
540.2194
543.2051
538 539 540 541 542 543 544 545 m/z

1'-Benzyl-2'-oxo-4-(*o*-tolyl)-4,9-dihydrospiro[carbazole-2,3'-indoline]-3,3(1*H*)-dicarbonitrile (2e'):

white solid, 7%, m.p. 196-199 ºC; ^{1}H NMR (400 MHz, $CDCl_3$) δ: 8.00 (s, 1H, NH), 7.89 (d, *J* = 7.6 Hz, 1H, ArH), 7.53 (d, *J* = 7.6 Hz, 1H, ArH), 7.38-7.33 (m, 6H, ArH), 7.31-7.28 (m, 2H, ArH), 7.20 (t, *J* = 7.6 Hz, 2H, ArH), 7.09 (t, *J* = 7.6 Hz, 1H, ArH), 6.82 (t, *J* = 7.6 Hz, 1H, ArH), 6.28 (d, *J* = 8.0 Hz, 1H, ArH), 6.10 (s, 1H, CH), 5.07 (d, *J* = 15.6 Hz, 1H, CH), 4.90 (d, *J* =16.0 Hz, 1H, CH), 3.87 (d, *J* =16.4 Hz, 1H, CH), 3.18 (d, *J* =17.6 Hz, 1H, CH), 2.63 (s, 3H, CH_3); ^{13}C NMR (400 MHz, $CDCl_3$) δ: 172.9, 142.5, 138.0, 136.5, 134.7, 132.9, 131.0, 131.0, 130.8, 129.0, 128.9, 128.9, 128.8, 127.9, 127.4, 126.6, 126.2, 125.8, 124.5, 123.9, 122.1, 120.0, 119.7, 113.4, 110.7, 110.0, 108.9, 50.8, 46.5, 44.4, 39.5, 29.3, 20.1; IR (KBr) υ: 3289, 3231, 3178, 3055, 2948, 2867, 2148, 1855, 1648, 1637, 1546, 1417, 1376, 1236, 1138, 1137, 937, 948, 845, 736 cm^{-1}; MS (*m/z*): HRMS (ESI) Calcd. for $C_{35}H_{16}N_4O$ ([M+Na]$^+$): 541.1999, found: 541.1990.

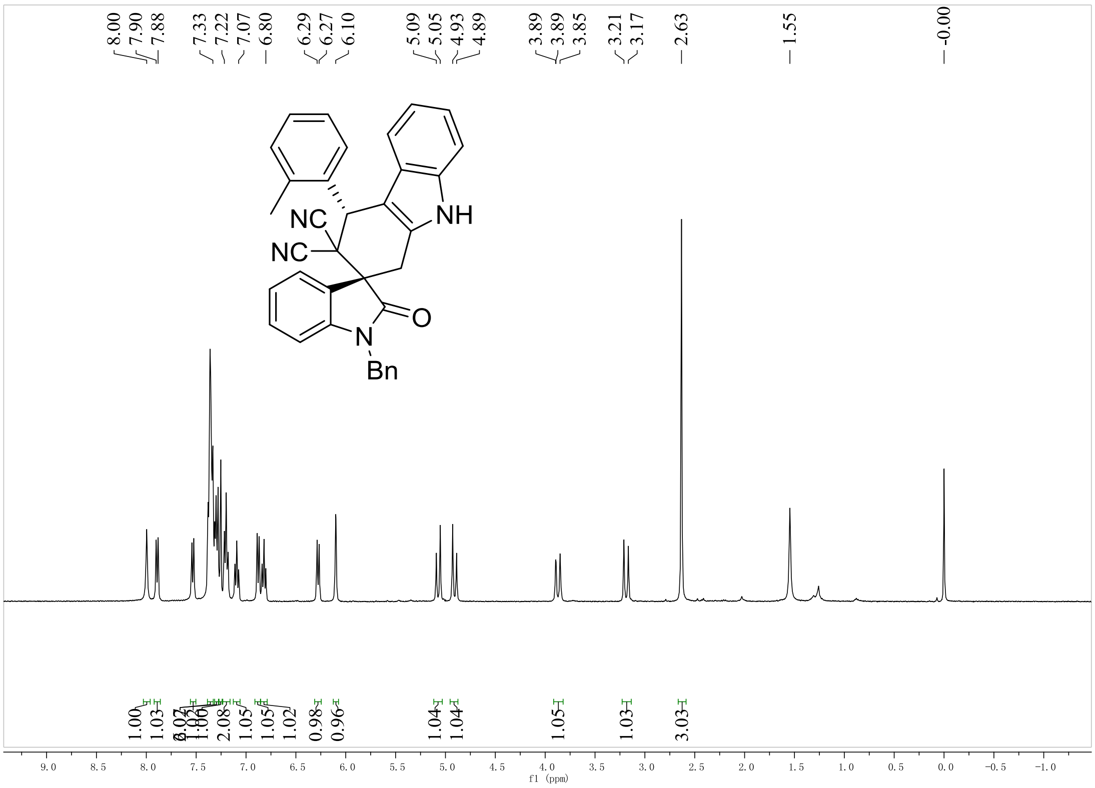

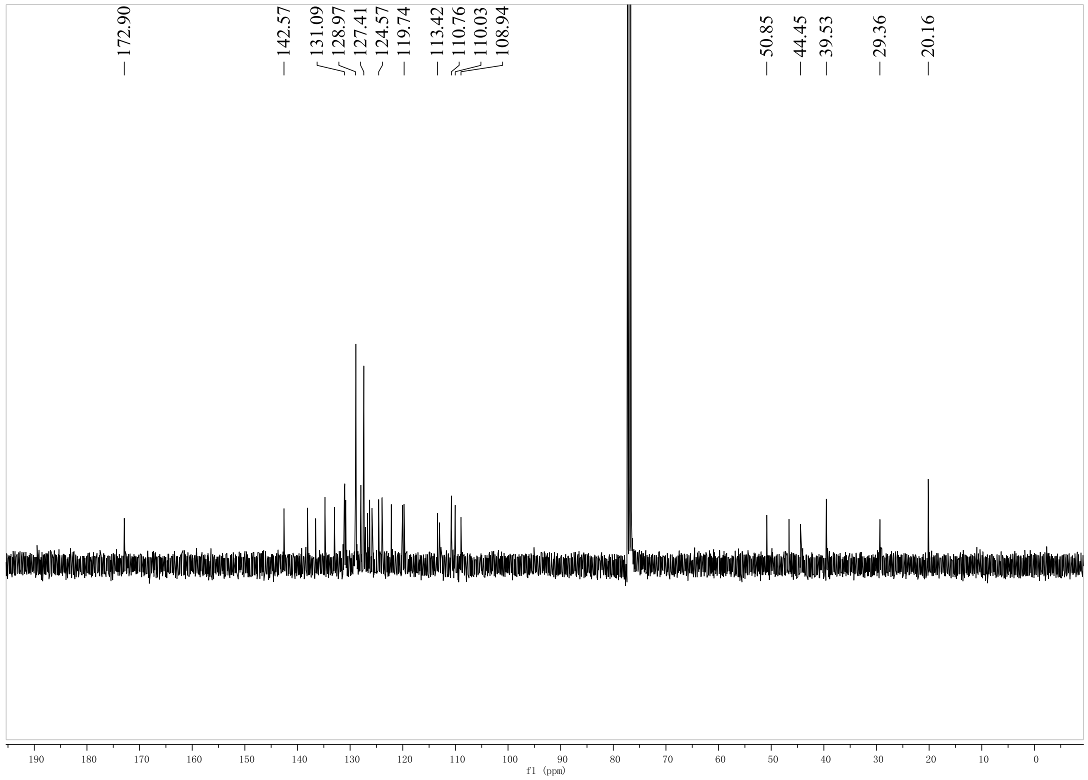

172.90
142.57
131.09
128.97
127.41
124.57
119.74
113.42
110.76
110.03
108.94
50.85
44.45
39.53
29.36
20.16
190
180
170
160
150
140
130
120
110
100
90
80
70
60
50
40
30
20
10
0
f1 (ppm)

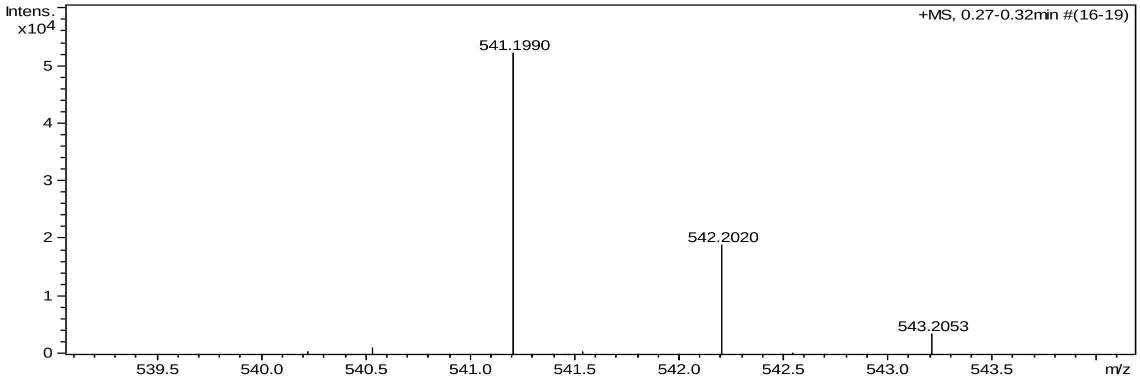

Intens.
x10⁴
+MS, 0.27-0.32min #(16-19)
541.1990
542.2020
543.2053
5
4
3
2
1
0
539.5
540.0
540.5
541.0
541.5
542.0
542.5
543.0
543.5
m/z

1'-Butyl-5'-chloro-4-(2-methoxyphenyl)-2'-oxo-4,9-dihydrospiro[carbazole-2,3'-indoline]-3,3(1*H*)-dicarbonitrile (2f):

white solid, 58%, m.p. 190-193 °C; ^{1}H NMR (400 MHz, $CDCl_3$) δ: 7.98 (s, 1H, NH), 7.81 (d, *J* = 2.0 Hz, 1H, ArH), 7.45-7.38 (m, 3H, ArH), 7.29 (d, *J* = 8.0 Hz, 1H, ArH), 7.10 (t, *J* = 8.0 Hz, 1H, ArH), 7.04 (d, *J* = 8.0 Hz, 1H, ArH), 6.95 (t, *J* = 7.6 Hz, 1H, ArH), 6.91 (d, *J* = 8.8 Hz, 1H, ArH), 6.85 (t, *J* = 7.6 Hz, 1H, ArH), 6.49 (d, *J* = 8.4 Hz, 1H, ArH), 6.23 (s, 1H, CH), 3.93-3.90 (m, 1H, CH), 3.88 (s, 3H, OCH_3), 3.79 (dd, J_1 =17.2 Hz, J_2 =2.0 Hz, 1H, CH), 3.66-3.60 (m, 1H, CH), 3.11 (d, *J* = 17.6 Hz, 1H, CH), 1.74-1.66 (m, 2H, CH_2), 1.49-1.42 (m, 2H, CH_2), 0.97 (t, *J* = 7.2 Hz, 3H, CH_3); ^{13}C NMR (400 MHz, $CDCl_3$) δ: 172.2, 140.4, 136.5, 135.2, 133.4, 133.3, 132.0, 131.2, 129.5, 128.9, 128.9, 128.8, 126.6, 125.5, 125.1, 122.2, 120.1, 119.8, 112.8, 112.5, 110.8, 109.1, 107.1, 50.3, 47.6, 45.1, 40.3, 29.3, 28.9, 21.2, 20.2, 13.6; IR (KBr) υ: 3300, 3278, 3158, 3033, 2967, 2856, 2148, 1940, 1654, 1600, 1548, 1417, 1343, 1243, 1155, 1127, 945, 855, 748 cm^{-1}; MS (*m/z*): HRMS (ESI) Calcd. for $C_{32}H_{27}Cl^{35}N_4O_2$ ($[M+Na]^+$): 557.1715, found: 557.1705. $C_{32}H_{27}Cl^{37}N_4O_2$ ($[M+Na]^+$): 558.1748, found: 558.1734.

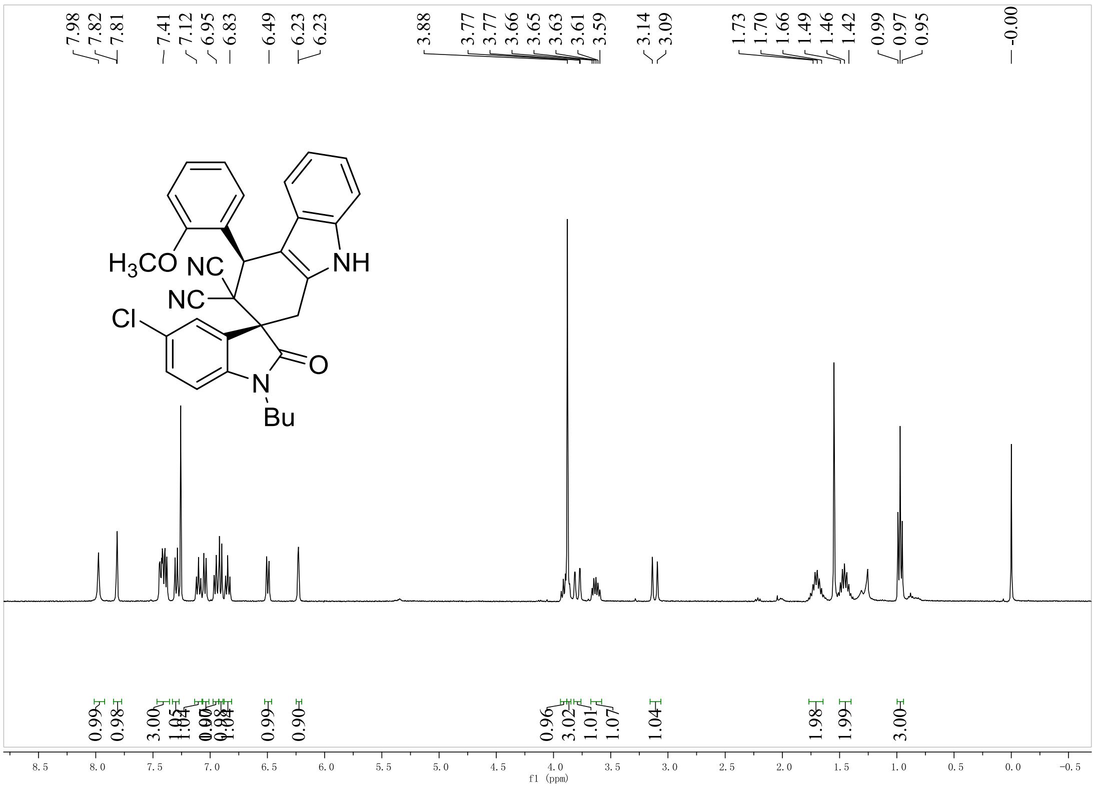

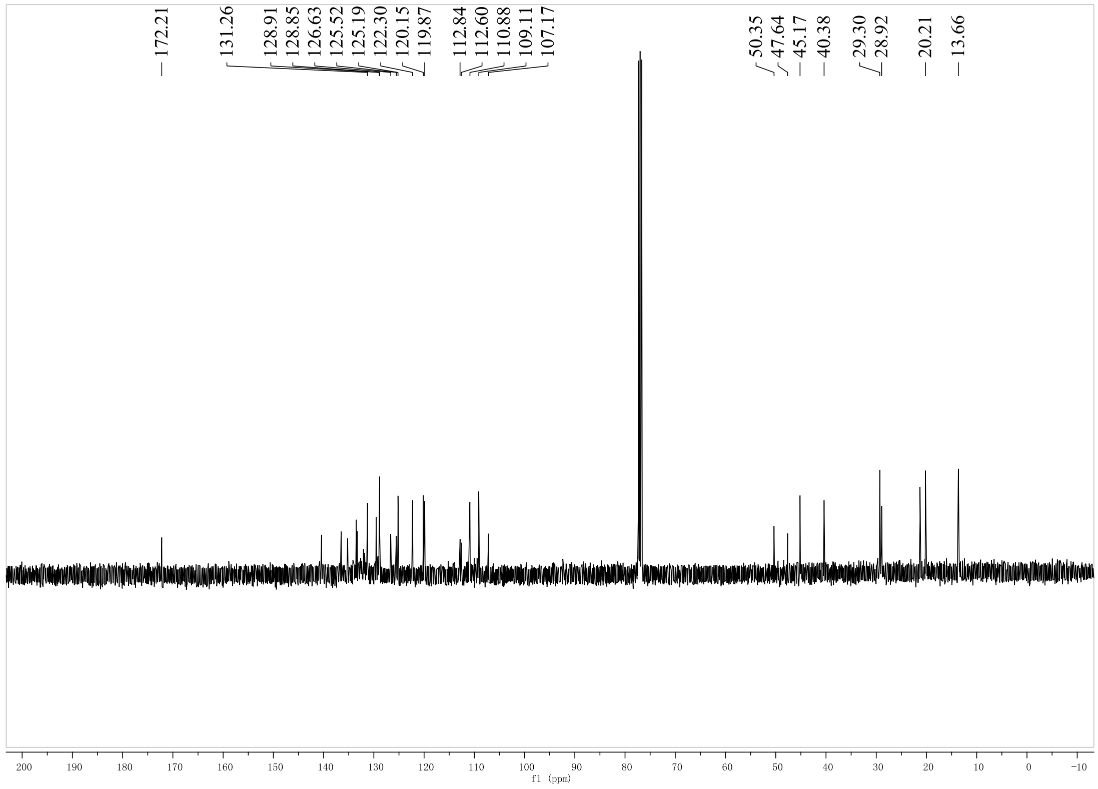
172.21
131.26
128.91
128.85
126.63
125.52
125.19
122.30
120.15
119.87
112.84
112.60
110.88
109.11
107.17
50.35
47.64
45.17
40.38
29.30
28.92
20.21
13.66
f1 (ppm)

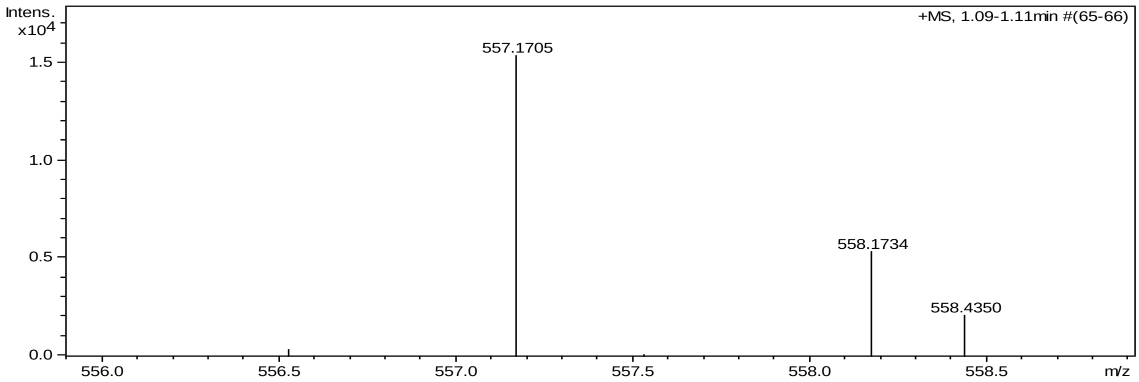
Intens.
x10^4
+MS, 1.09-1.11min #(65-66)
557.1705
558.1734
558.4350
m/z

1'-Butyl-4-(4-chlorophenyl)-5'-methyl-2'-oxo-4,9-dihydrospiro[carbazole-2,3'-indoline]-3,3(1 *H*)-dicarbonitrile (2g):

white solid, 54%, m.p. 186-189 ºC; ^{1}H NMR (400 MHz, $CDCl_3$) δ: 8.40 (s, 1H, NH), 7.47-7.43 (m, 2H, ArH), 7.41 (d, J = 8.4 Hz, 1H, ArH), 7.34-7.33 (m, 2H, ArH), 7.24-7.19 (m, 2H, ArH), 6.95 (dd, J_1 = 18.0 Hz, J_2 = 8.0 Hz, 2H, ArH), 6.70-6.69 (m, 1H, ArH), 6.55 (d, J = 8.0 Hz, 1H, ArH), 4.84 (d, J = 1.2 Hz, 1H, CH), 3.99 (dd, J_1 = 17.2 Hz, J_2 = 2.0 Hz, 1H, CH), 3.92 (q, J = 7.2 Hz, 1H, CH), 3.71-3.63 (m, 1H, CH), 2.93 (d, J = 17.2 Hz, 1H, CH), 2.18 (s, 3H, CH_3), 1.78-1.70 (m, 2H, CH_2), 1.48-1.42 (m, 2H, CH_2), 0.96 (t, J = 7.2 Hz, 3H, CH_3); ^{13}C NMR (400 MHz, $CDCl_3$) δ: 172.7, 141.1, 136.7, 135.4, 133.2, 132.3, 131.4, 130.9, 125.4, 125.2, 125.1, 122.6, 120.1, 120.0, 112.4, 111.6, 111.2, 109.4, 105.9, 51.6, 46.8, 46.3, 40.6, 29.3, 28.6, 21.2, 20.2, 13.6; IR (KBr) υ: 3300, 3259, 3167, 3017, 2955, 2837, 2148, 1865, 1681, 1631, 1548, 1467, 1355, 1248, 1131, 1101, 947, 902, 846 cm^{-1}; MS (*m*/*z*): HRMS (ESI) Calcd. for $C_{32}H_{27}Cl^{35}N_4O$ ([M+Na]$^+$): 541.1766, found: 541.1766. $C_{32}H_{27}Cl^{37}N_4O$ ([M+Na]$^+$): 542.1799, found: 542.1794.

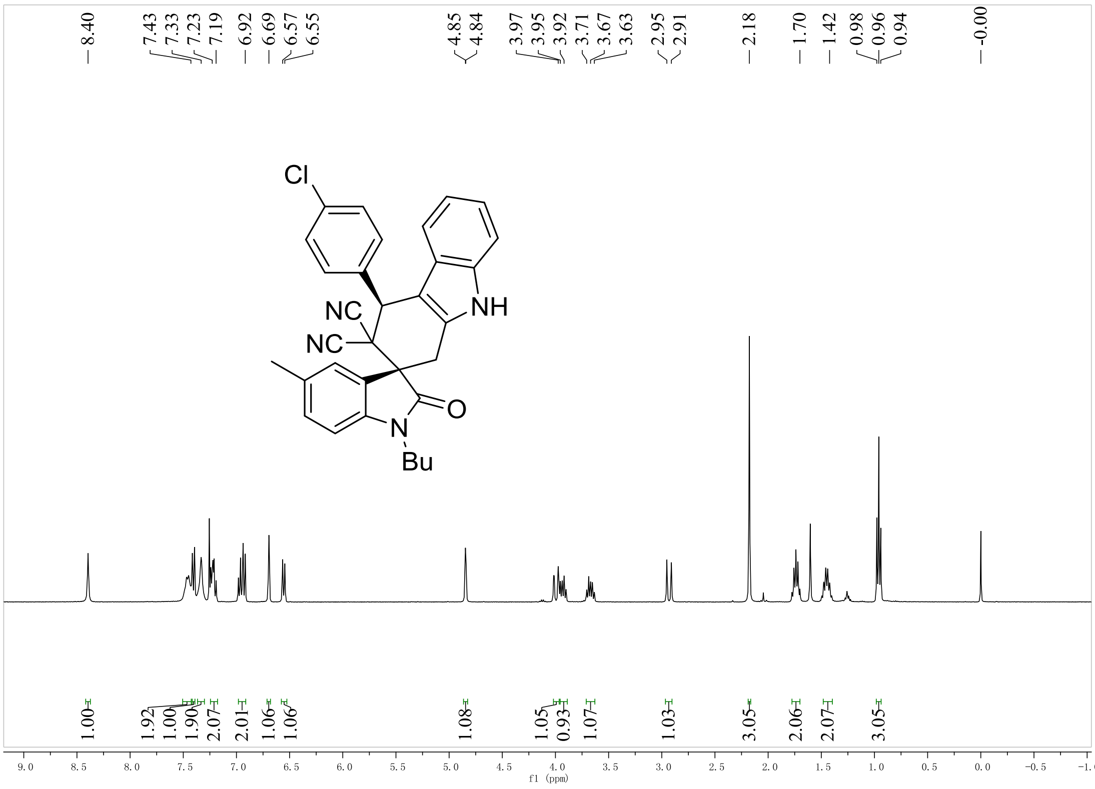

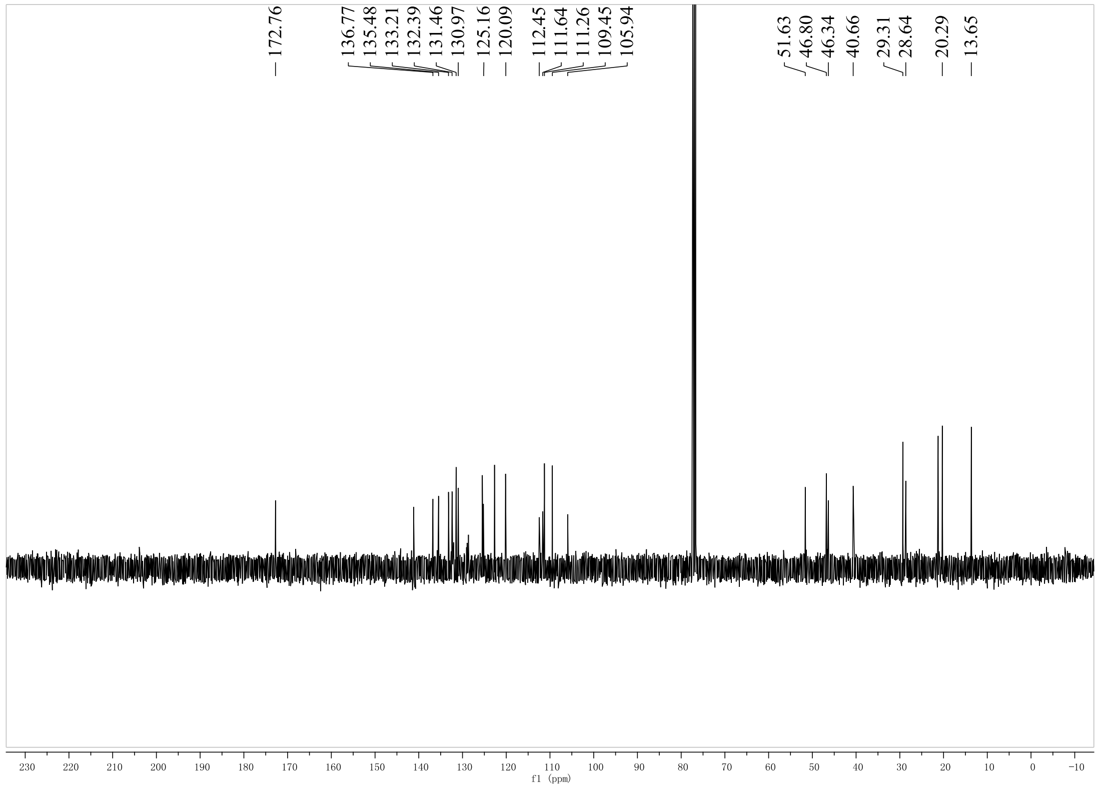
172.76
136.77
135.48
133.21
132.39
131.46
130.97
125.16
120.09
112.45
111.64
111.26
109.45
105.94
51.63
46.80
46.34
40.66
29.31
28.64
20.29
13.65
230 220 210 200 190 180 170 160 150 140 130 120 110 100 90 80 70 60 50 40 30 20 10 0 -10
f1 (ppm)

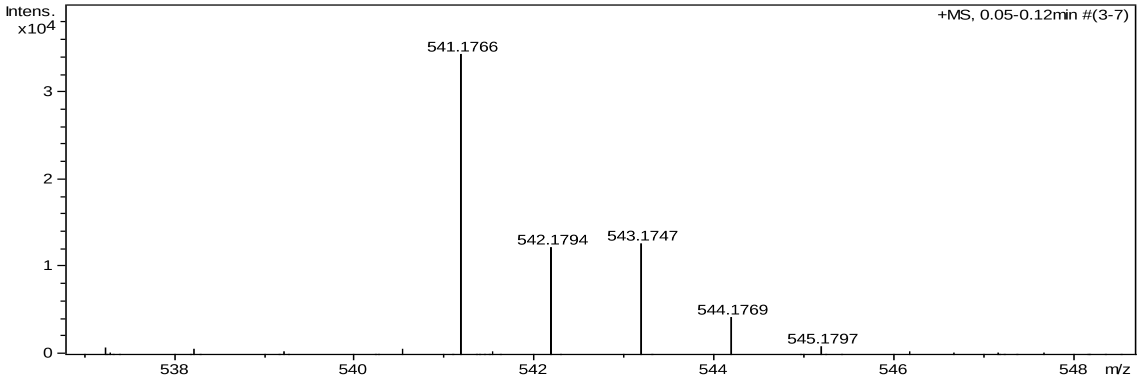
Intens.
x10^4
+MS, 0.05-0.12min #(3-7)
541.1766
542.1794
543.1747
544.1769
545.1797
0
1
2
3
538
540
542
544
546
548
m/z

1'-Butyl-4-(4-chlorophenyl)-5'-methyl-2'-oxo-4,9-dihydrospiro[carbazole-2,3'-indoline]-3,3(1*H*)-dicarbonitrile (2g'):

white solid, 6%, m.p. 193-197 °C; ^{1}H NMR (400 MHz, $CDCl_3$) δ: 8.09 (s, 1H, NH), 7.70-7.59 (m, 2H, ArH), 7.48-7.37 (m, 3H, ArH), 7.29 (t, J = 8.4 Hz, 2H, ArH), 7.11 (t, J = 8.0 Hz, 1H, ArH), 6.88 (t, J = 8.0 Hz, 2H, ArH), 6.47 (d, J = 8.0 Hz, 1H, ArH), 5.45 (s, 1H, CH), 3.84-3.75 (m, 2H, CH_2), 3.64 (q, J = 7.2 Hz, 1H, CH), 3.10 (d, J = 17.6 Hz, 1H, CH), 2.40 (s, 3H, CH_3), 1.71 (t, J = 7.2 Hz, 2H, CH_2), 1.43-1.41 (m, 2H, CH_2), 0.96 (t, J = 7.2 Hz, 3H, CH_3); ^{13}C NMR (400 MHz, $CDCl_3$) δ: 172.2, 140.4, 136.5, 135.2, 133.5, 133.3, 131.2, 129.5, 128.9, 128.8, 126.6, 125.5, 125.1, 122.3, 120.1, 119.8, 112.8, 112.5, 110.8, 109.1, 107.1, 50.3, 47.6, 45.1, 40.3, 29.3, 28.9, 21.2, 20.2, 13.6; IR (KBr) υ: 3300, 3248, 3170, 3049, 2966, 2849, 2170, 1864, 1654, 1600, 1560, 1455, 1348, 1267, 1131, 1100, 970, 917, 855 cm^{-1}; MS (m/z): HRMS (ESI) Calcd. for $C_{32}H_{27}Cl^{35}N_4O$ ($[M+Na]^+$): 541.1766, found: 541.1765. $C_{32}H_{27}Cl^{37}N_4O$ ($[M+Na]^+$): 542.1799, found: 542.1794.

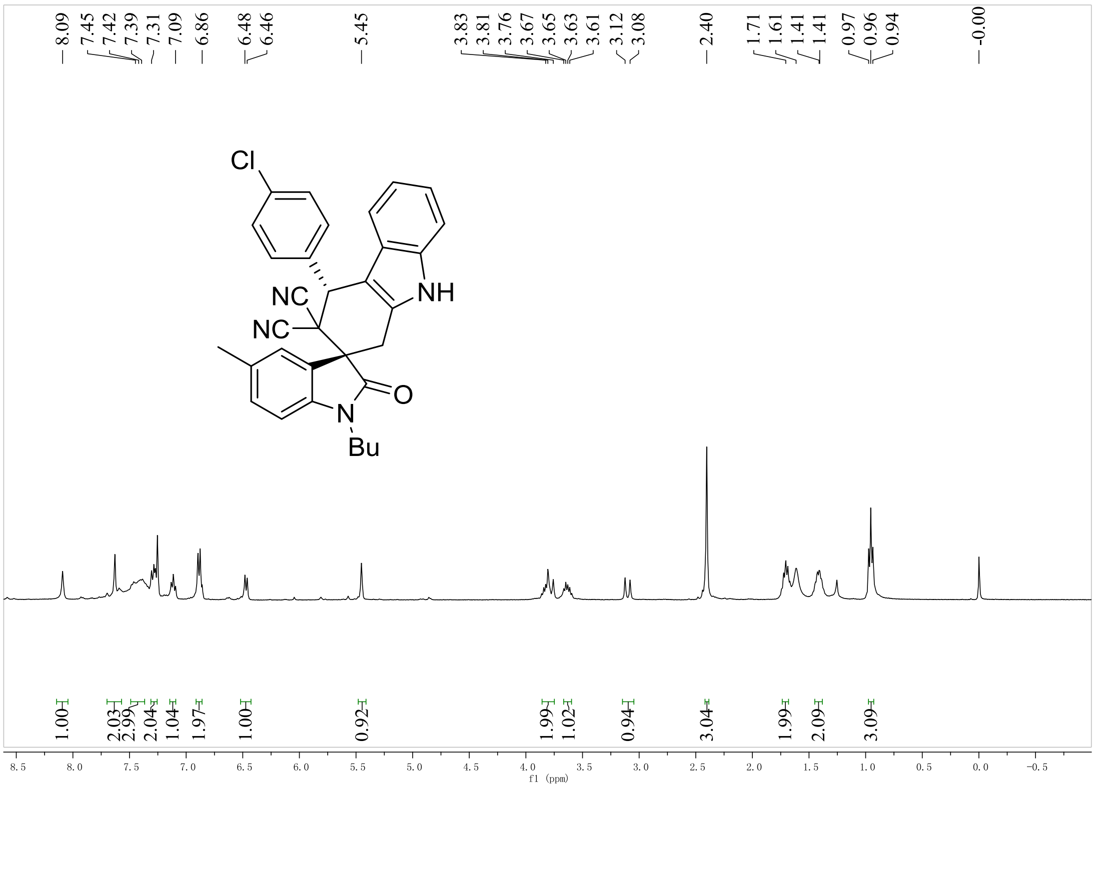

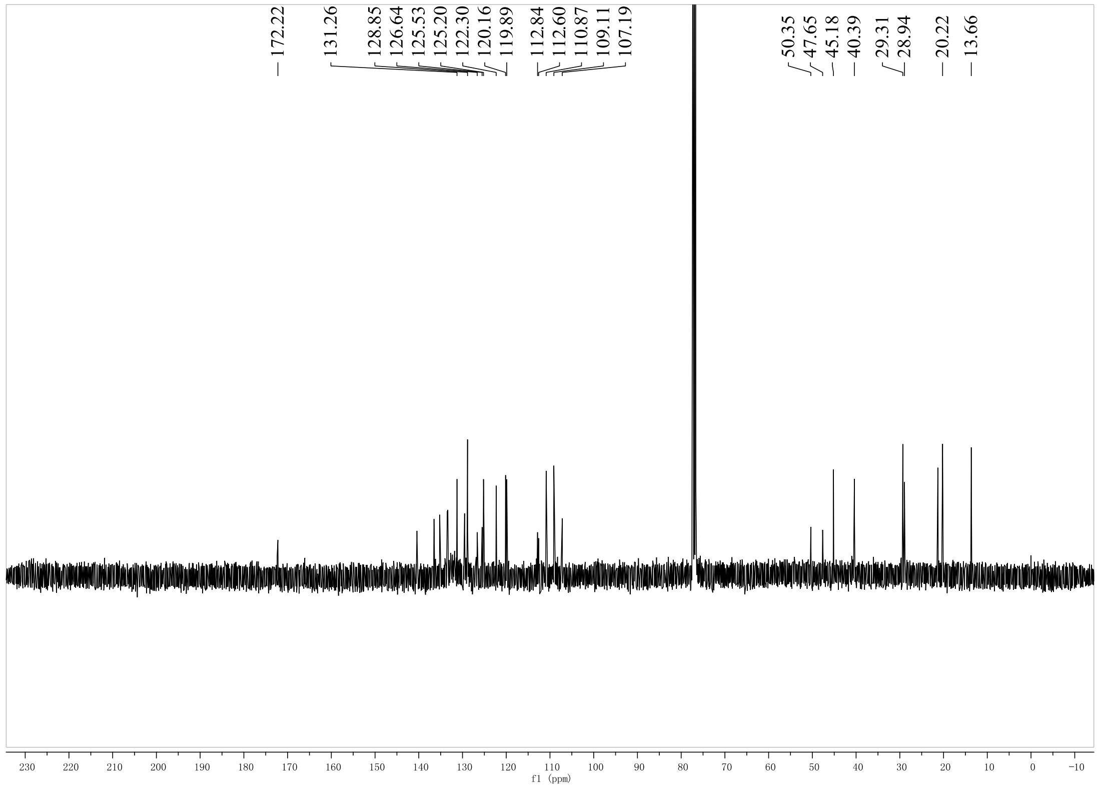
172.22
131.26
128.85
126.64
125.53
125.20
122.30
120.16
119.89
112.84
112.60
110.87
109.11
107.19
50.35
47.65
45.18
40.39
29.31
28.94
20.22
13.66
f1 (ppm)

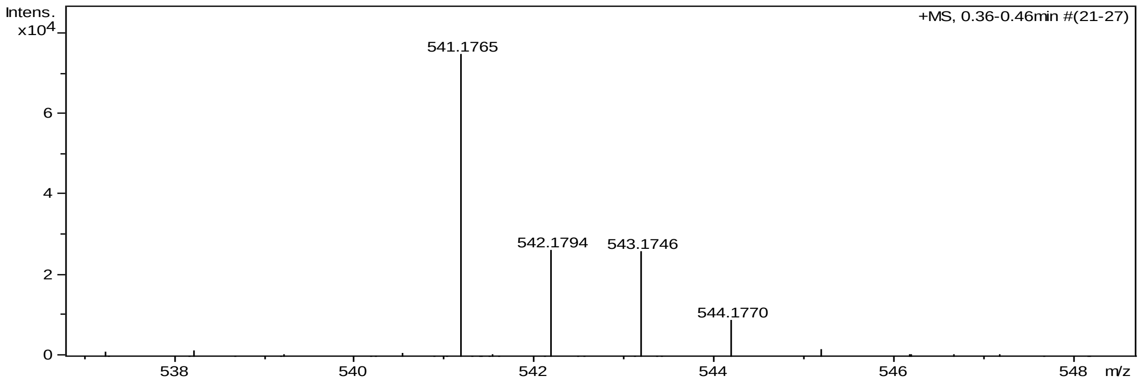
Intens.
x10^4
+MS, 0.36-0.46min #(21-27)
541.1765
542.1794
543.1746
544.1770
m/z

1',3'-Dimethyl-2,4-di-*p*-tolyl-2'*H*-spiro[carbazole-3,5'-pyrimidine]-2',4',6'(1'*H*,3'*H*)-trione (3a):

yellow solid, 75%, m.p. 190-192 ^{o}C; ^{1}H NMR (400 MHz, $CDCl_3$) δ: 7.59 (d, *J* = 7.6 Hz, 1H, ArH), 7.35-7.30 (m, 4H, ArH), 7.25-7.17 (m, 2H, ArH), 7.04 (s, 1H, ArH), 6.96-6.91 (m, 4H, ArH), 6.35 (d, *J* = 7.6 Hz, 1H, CH), 3.10 (s, 3H, CH_3), 3.07 (s, 3H, CH_3), 2.37 (s, 3H, CH_3), 2.34 (s, 3H, CH_3); ^{13}C NMR (400 MHz, $CDCl_3$) δ: 165.3, 162.7, 157.9, 149.7, 149.0, 147.3, 142.6, 139.1, 138.7, 137.1, 136.0, 133.7, 130.6, 130.3, 130.0, 129.1, 128.7, 128.3, 128.0, 126.3, 126.1, 125.5, 124.7, 124.2, 123.1, 120.8, 29.6, 28.8, 21.4, 21.3; IR (KBr) υ: 3219, 3158, 3043, 2966, 2900, 1843, 1755, 1648, 1617, 1537, 1466, 1358, 1318, 1266, 1150, 987, 899, 765 cm^{-1}; MS (*m*/*z*): HRMS (ESI) Calcd. for $C_{31}H_{25}N_3O_3$ ([M+Na]$^{+}$): 510.1788, found: 510.1788.

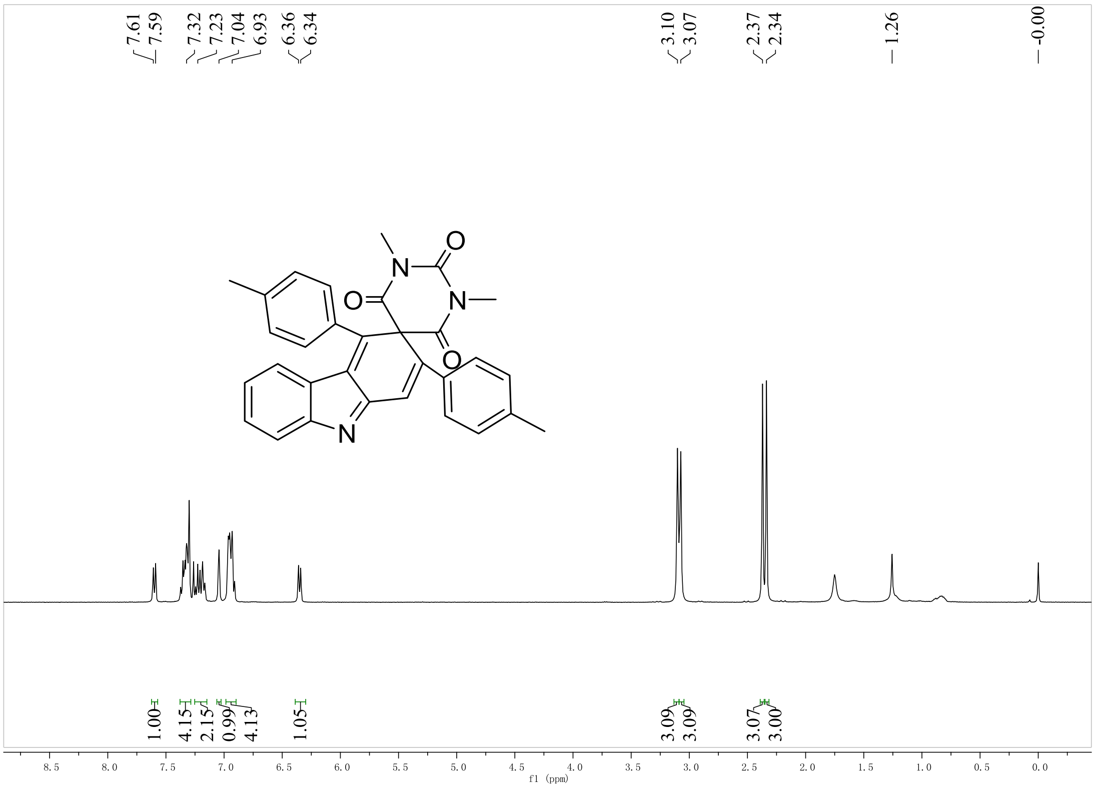

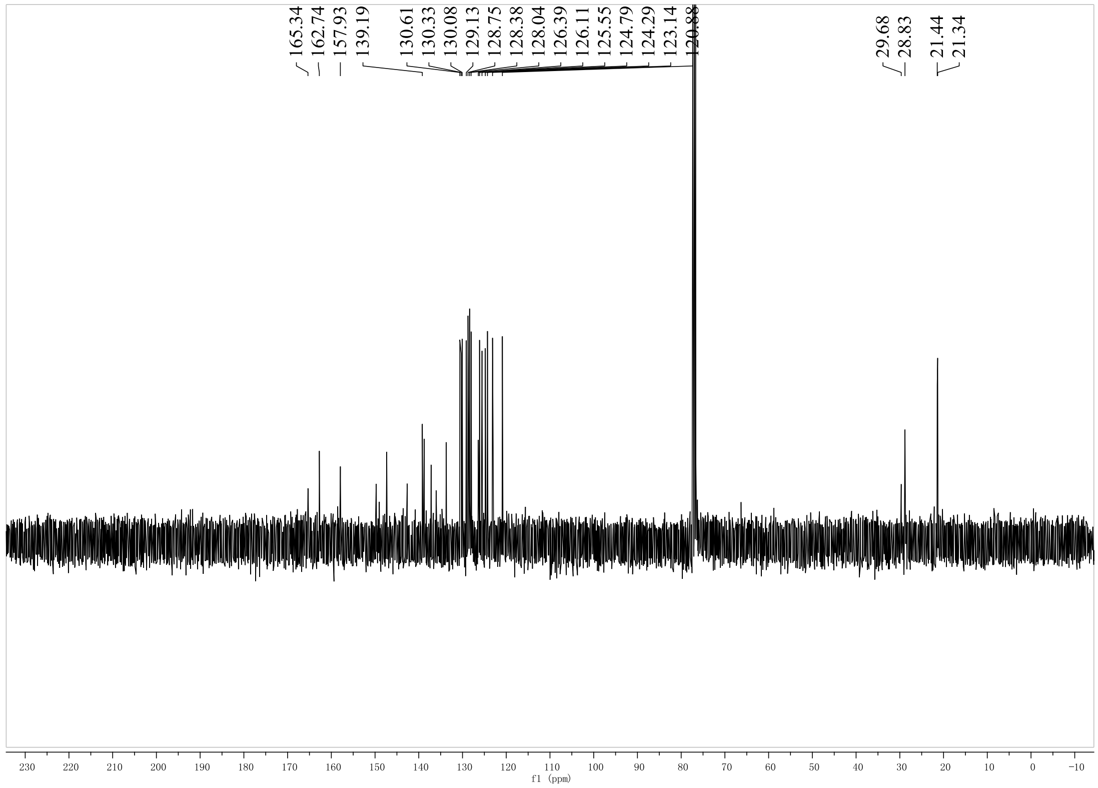
165.34
162.74
157.93
139.19
130.61
130.33
130.08
129.13
128.75
128.38
128.04
126.39
126.11
125.55
124.79
124.29
123.14
120.88
29.68
28.83
21.44
21.34
230
220
210
200
190
180
170
160
150
140
130
120
110
100
90
80
70
60
50
40
30
20
10
0
−10
f1 (ppm)

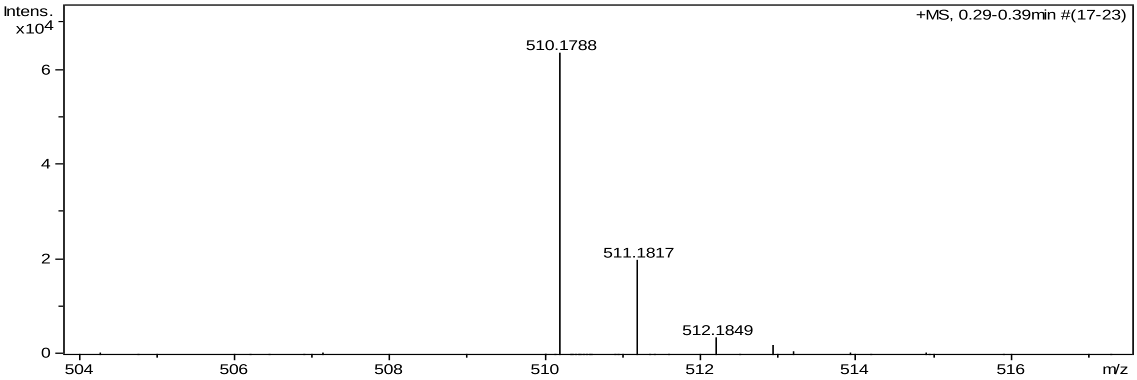
Intens.
x10^4
+MS, 0.29-0.39min #(17-23)
510.1788
511.1817
512.1849
6
4
2
0
504
506
508
510
512
514
516
m/z

2,4-Bis(3-chlorophenyl)-1',3'-dimethyl-2'H-spiro[carbazole-3,5'-pyrimidine]-2',4',6'(1'H,3'H)-trione (3b):

yellow solid, 67%, m.p. 194-197 ºC; ^{1}H NMR (400 MHz, $CDCl_3$) δ: 7.62 (d, *J* = 8.0 Hz, 1H, ArH), 7.53-7.50 (m, 1H, ArH), 7.44 (t, *J* = 8.0 Hz, 1H, ArH), 7.36 (t, *J* = 8.0 Hz, 2H, ArH), 7.30 (t, *J* = 8.0 Hz, 1H, ArH), 7.28-7.27 (m, 1H, ArH), 7.20-7.18 (m, 2H, ArH), 7.06 (t, *J* = 7.6 Hz, 2H, ArH), 6.98 (t, *J* = 7.6 Hz, 1H, ArH), 6.36 (d, *J* = 8.0 Hz, 1H, CH), 3.16 (s, 3H, CH_3), 3.09 (s, 3H, CH_3); ^{13}C NMR (400 MHz, $CDCl_3$) δ: 164.8, 162.0, 157.9, 149.3, 145.2, 139.9, 138.6, 136.6, 135.4, 135.3, 134.9, 130.8, 130.7, 130.2, 130.2, 129.5, 127.9, 127.8, 127.0, 126.1, 126.0, 125.8, 125.7, 123.1, 121.2, 66.0, 29.0; IR (KBr) υ: 3255, 3175, 3048，2978, 1876, 1756, 1667, 1601, 1556, 1478, 1362, 1258, 1217, 1180, 964, 832, 745 cm^{-1}; MS (*m/z*): HRMS (ESI) Calcd. for $C_{29}H_{19}Cl_2{}^{35}N_3O_3$ ($[M+Na]^+$): 550.0696, found: 550.0692. $C_{29}H_{19}Cl_2{}^{37}N_3O_3$ ($[M+Na]^+$): 552.0666, found: 552.0668.

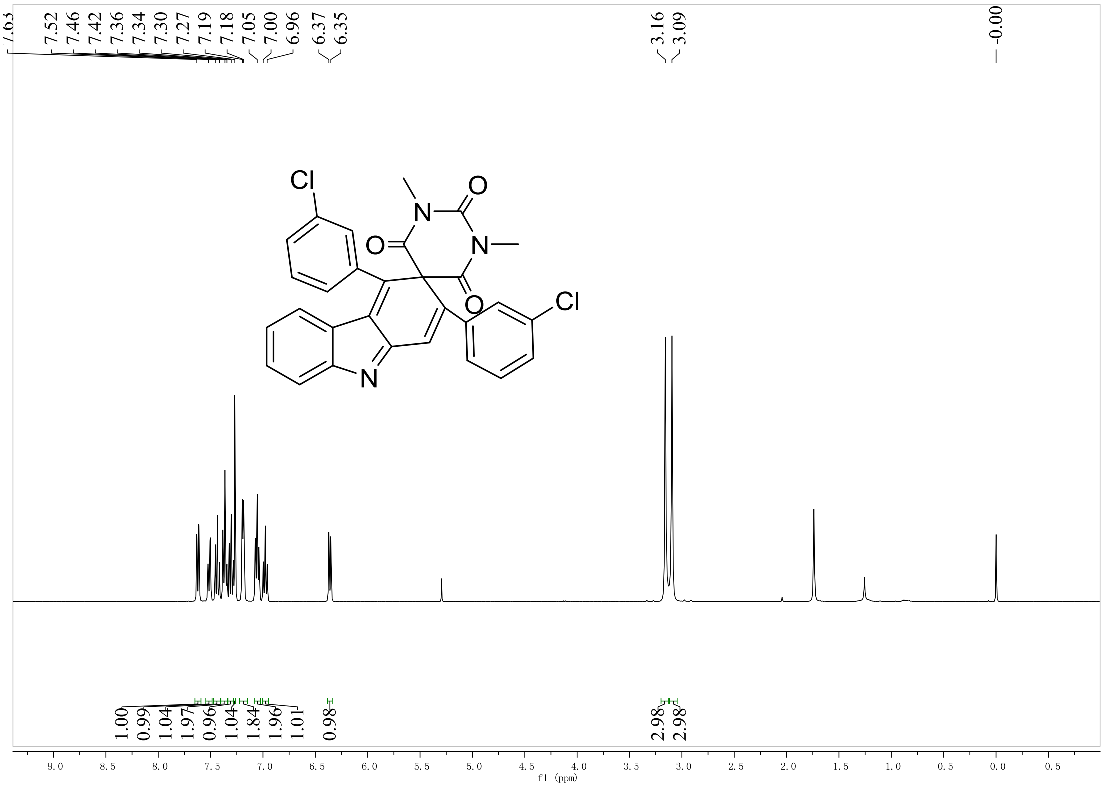

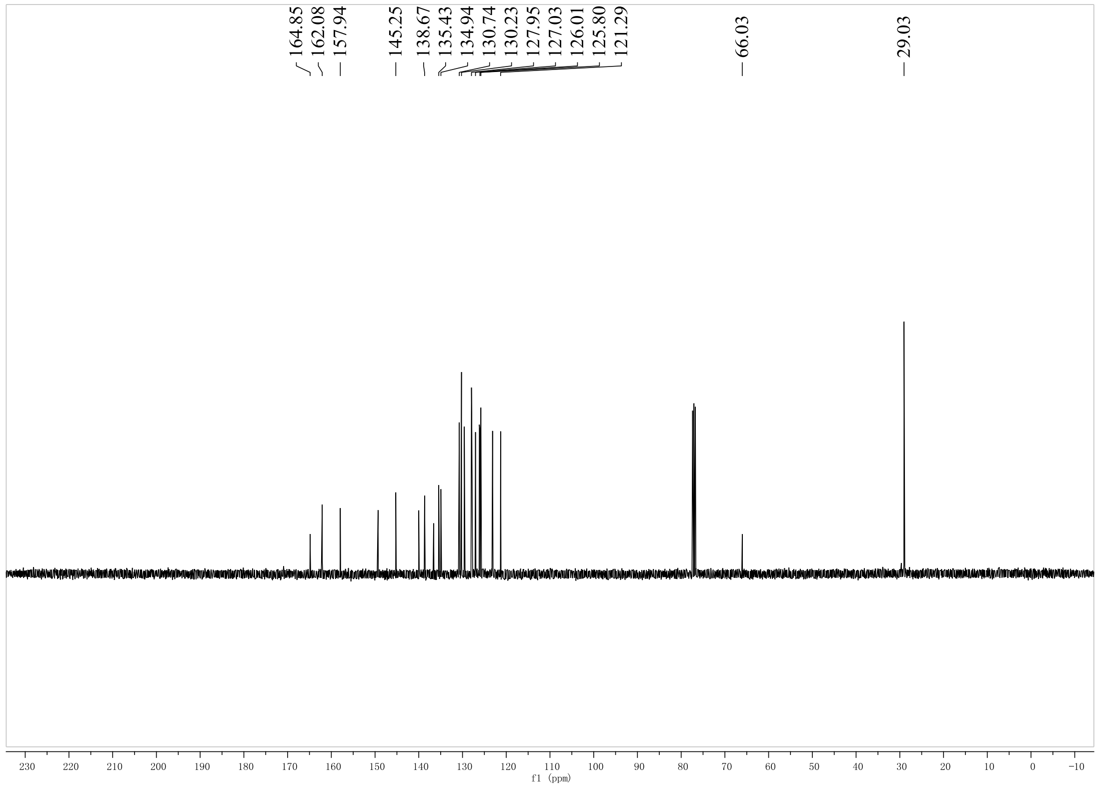
164.85
162.08
157.94
145.25
138.67
135.43
134.94
130.74
130.23
127.95
127.03
126.01
125.80
121.29
66.03
29.03
230 220 210 200 190 180 170 160 150 140 130 120 110 100 90 80 70 60 50 40 30 20 10 0 −10
f1 (ppm)

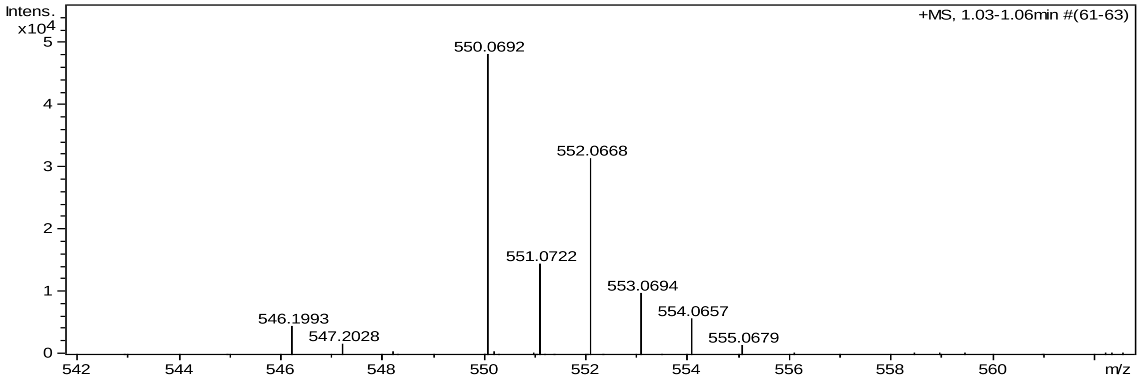
Intens.
x10^4
+MS, 1.03-1.06min #(61-63)
550.0692
552.0668
551.0722
553.0694
554.0657
546.1993
547.2028
555.0679
542 544 546 548 550 552 554 556 558 560
m/z

1',3'-Dimethyl-2,4-bis(3-nitrophenyl)-2'H-spiro[carbazole-3,5'-pyrimidine]-2',4',6'(1'H,3'H)-trione (3c):

yellow solid, 58%, m.p. 200-203 ºC; ^{1}H NMR (400 MHz, $CDCl_3$) δ: 8.42 (d, *J* = 8.0 Hz, 1H, ArH), 8.27 (d, *J* = 8.0 Hz, 1H, ArH), 8.07-8.05 (m, 2H, ArH), 7.75 (t, *J* = 8.0 Hz, 1H, ArH), 7.66-7.56 (m, 4H, ArH), 7.42-7.37 (m, 2H, ArH), 6.97 (t, *J* = 8.0 Hz, 1H, ArH), 6.23 (d, *J* = 7.2 Hz, 1H, CH), 3.19 (s, 3H, CH_3), 3.08 (s, 3H, CH_3); ^{13}C NMR (400 MHz, $CDCl_3$) δ: 161.5, 157.9, 148.9, 148.6, 148.2, 143.8, 138.3, 138.2, 137.2, 135.1, 134.6, 134.1, 131.4, 130.8, 130.4, 127.9, 126.4, 125.4, 125.0, 124.3, 122.9, 122.8, 122.1, 121.7, 65.7, 29.2, 29.1; IR (KBr) υ: 3217, 3167, 3005，2967, 1945, 1832, 1746, 1676, 1523, 1411, 1348, 1234, 1221, 1167, 969, 848, 772 cm^{-1}; MS (*m/z*): HRMS (ESI) Calcd. for $C_{29}H_{19}N_5O_7$ ($[M+Na]^+$): 572.1177, found: 572.1172.

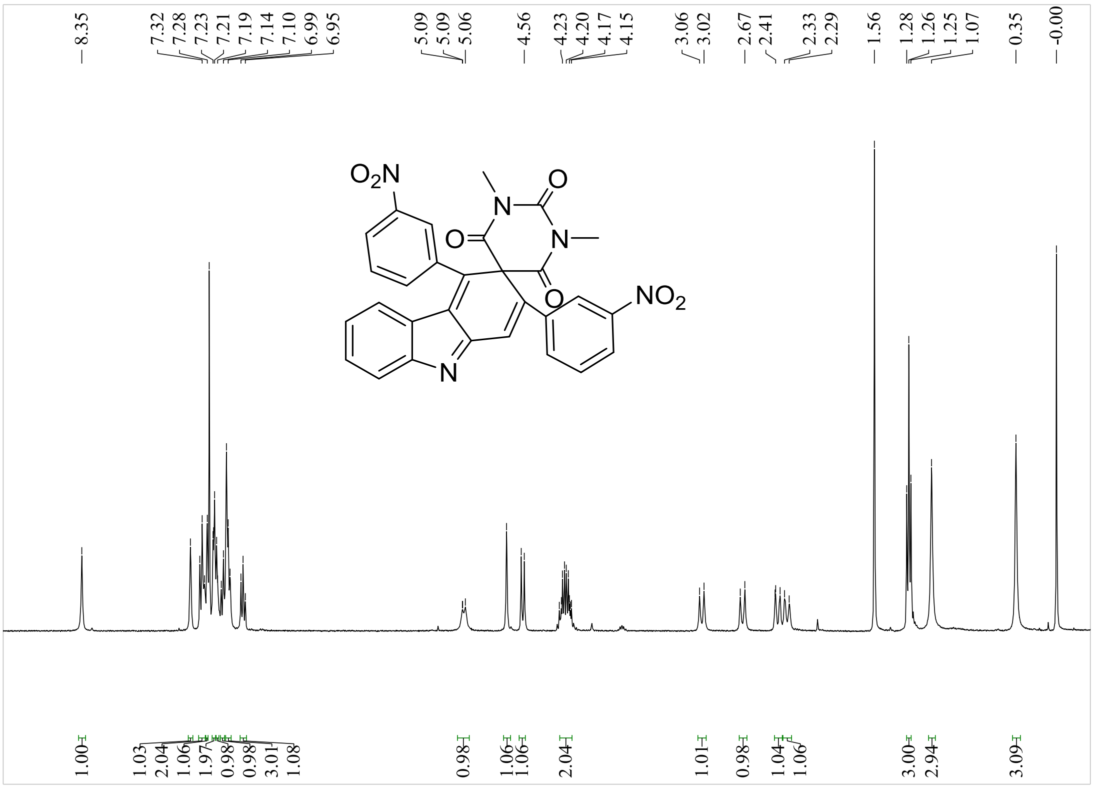

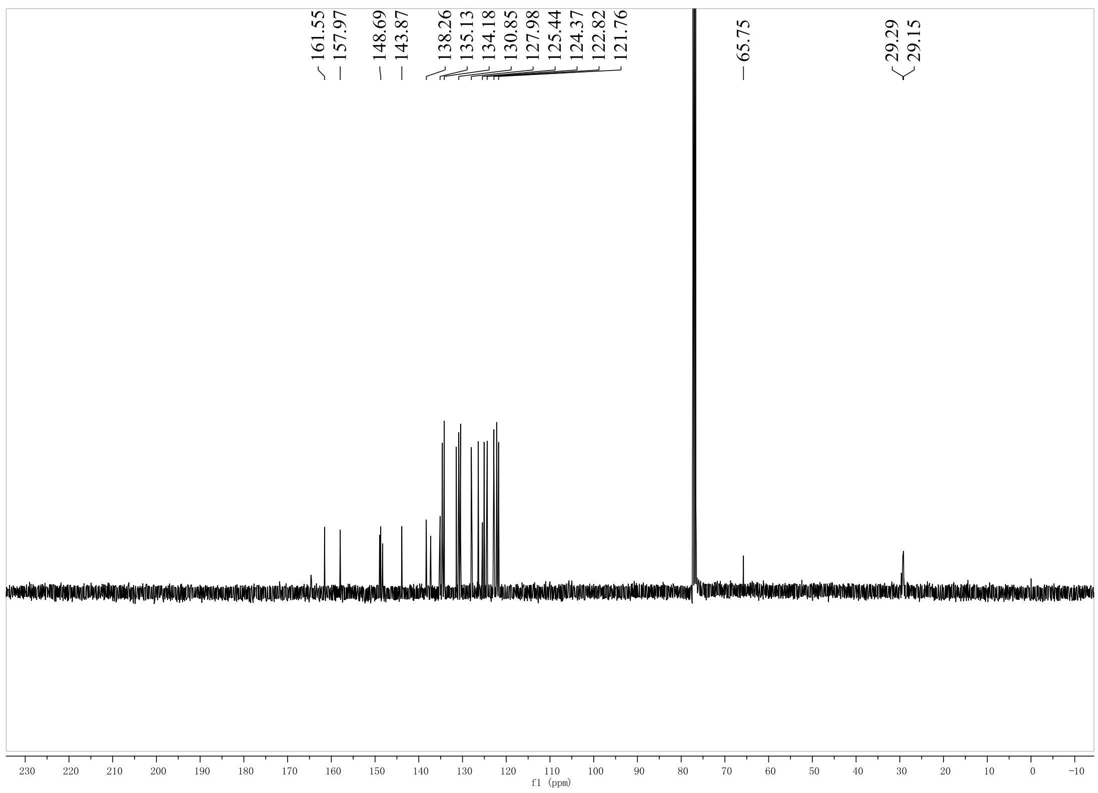
161.55
157.97
148.69
143.87
138.26
135.13
134.18
130.85
127.98
125.44
124.37
122.82
121.76
65.75
29.29
29.15
f1 (ppm)

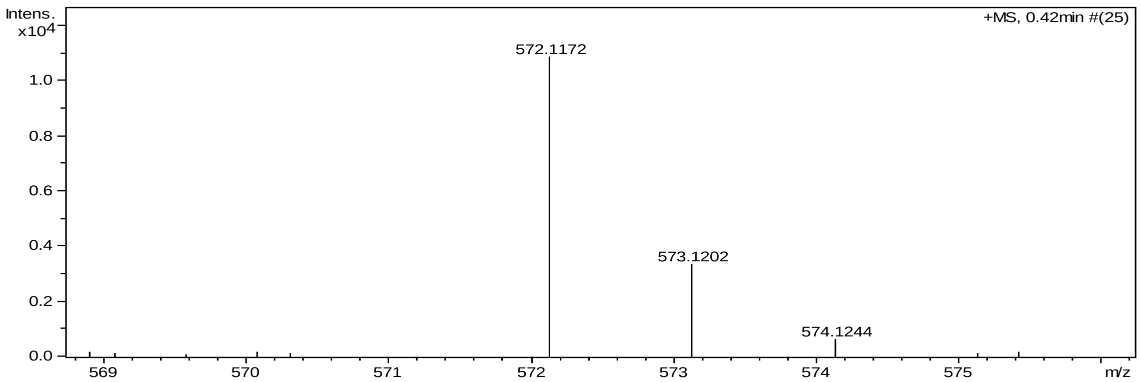
Intens.
x10^4
+MS, 0.42min #(25)
572.1172
573.1202
574.1244
m/z

1',3'-Dimethyl-2,4-diphenyl-1,2,4,9-tetrahydro-2'*H*-spiro[carbazole-3,5'-pyrimidine]-2',4',6'(1'*H*,3'*H*)-trione (4a):

purple solid, 82%, m.p. 205-208 ^{o}C; ^{1}H NMR (400 MHz, $CDCl_3$) δ: 8.02 (s, 1H, NH), 7.30-7.27 (m, 2H, ArH), 7.25-7.24 (m, 2H, ArH), 7.24-7.22 (m, 2H, ArH), 7.19-7.15 (m, 3H, ArH), 7.15-7.10 (m, 1H, ArH), 7.06 (t, *J* = 7.2 Hz, 1H, ArH), 6.94 (d, *J* = 7.2 Hz, 1H, ArH), 6.80 (t, *J* = 8.0 Hz, 1H, ArH), 6.41 (d, *J* = 8.0 Hz, 1H, ArH), 5.24 (s, 1H, CH), 4.13 (dd, J_1= 12.0 Hz, J_2= 5.6 Hz, 1H, CH), 3.90-3.82 (m, 1H, CH), 3.05 (dd, J_1= 16.4 Hz, J_2= 5.6 Hz, 1H, CH), 2.97 (s, 3H, CH_3), 2.81 (s, 3H, CH_3); ^{13}C NMR (400 MHz, $CDCl_3$) δ: 171.7, 167.8, 150.0, 138.9, 137.2, 136.0, 134.6, 129.3, 128.9, 128.7, 128.2, 128.2, 128.1, 128.1, 128.1, 126.3, 121.0, 119.7, 118.9, 110.6, 107.5, 62.5, 51.1, 48.2, 28.3, 27.8, 27.7; IR (KBr) υ: 3407, 3078, 2981, 1873, 1744, 1658, 1667, 1582, 1466, 1356, 1321, 1221, 1180, 912, 833 cm^{-1}; MS (*m*/*z*): HRMS (ESI) Calcd. for $C_{29}H_{25}N_3O_3$ ([M+Na]$^{+}$): 486.1788, found: 486.1794.

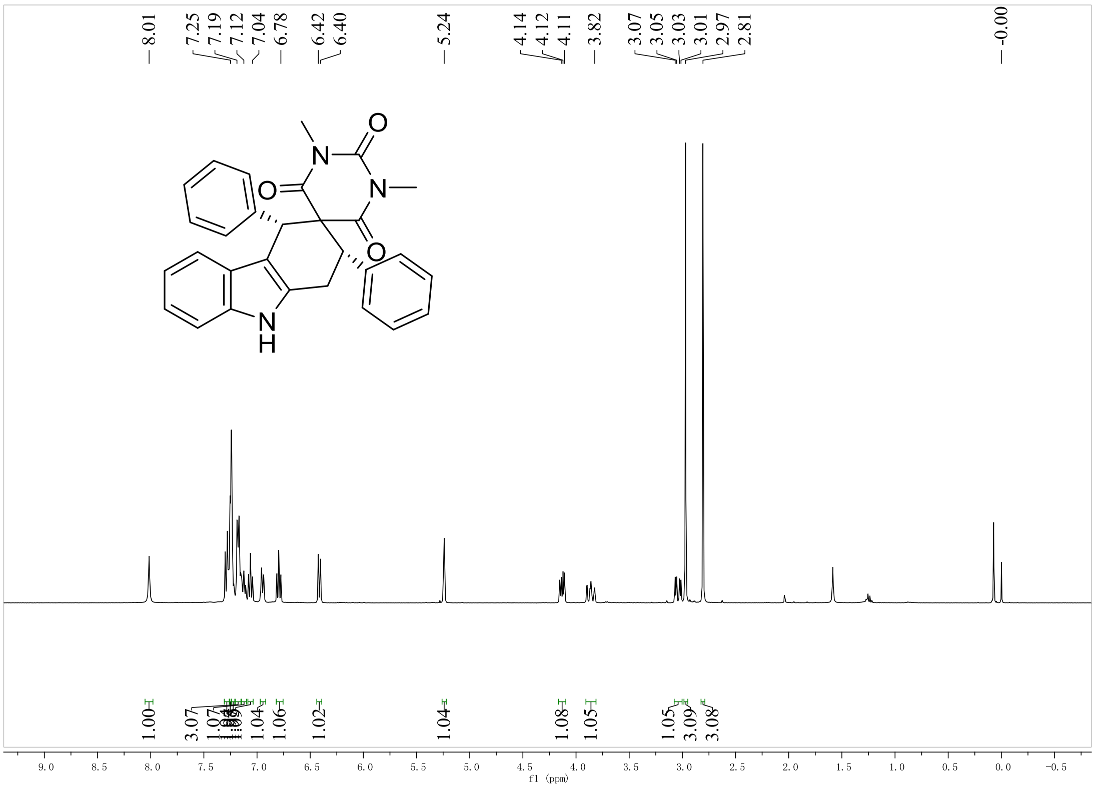

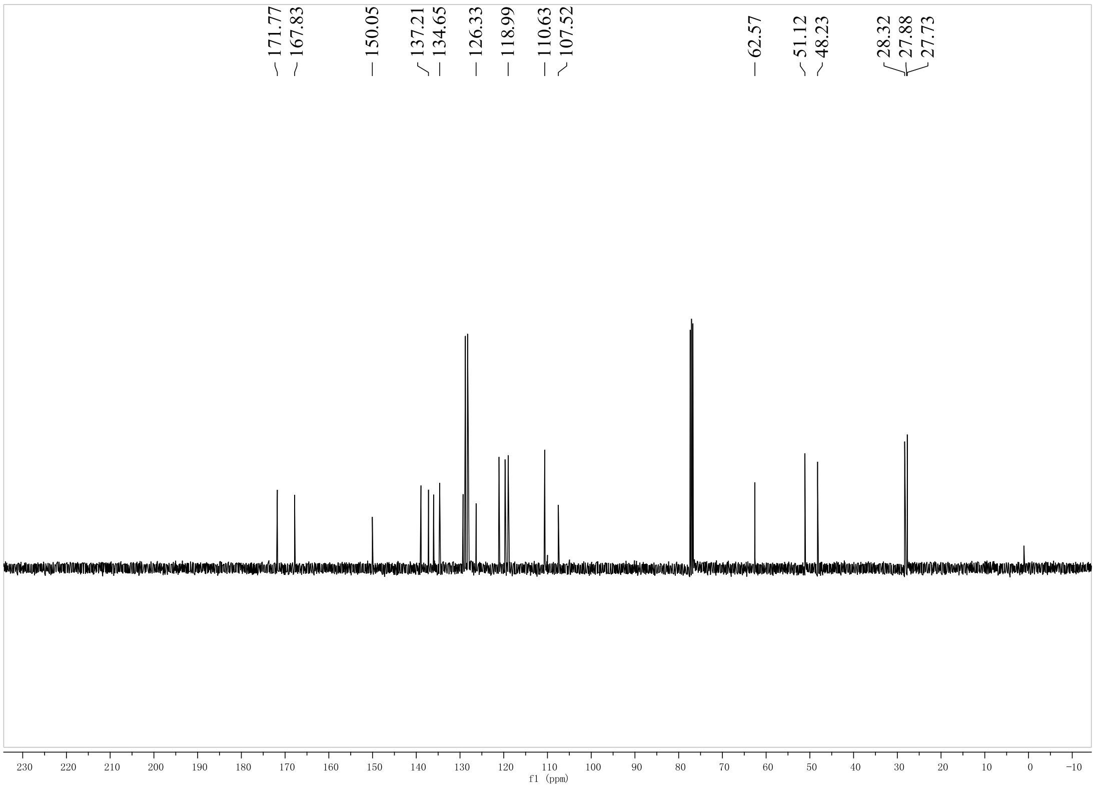

171.77
167.83
150.05
137.21
134.65
126.33
118.99
110.63
107.52
62.57
51.12
48.23
28.32
27.88
27.73
f1 (ppm)

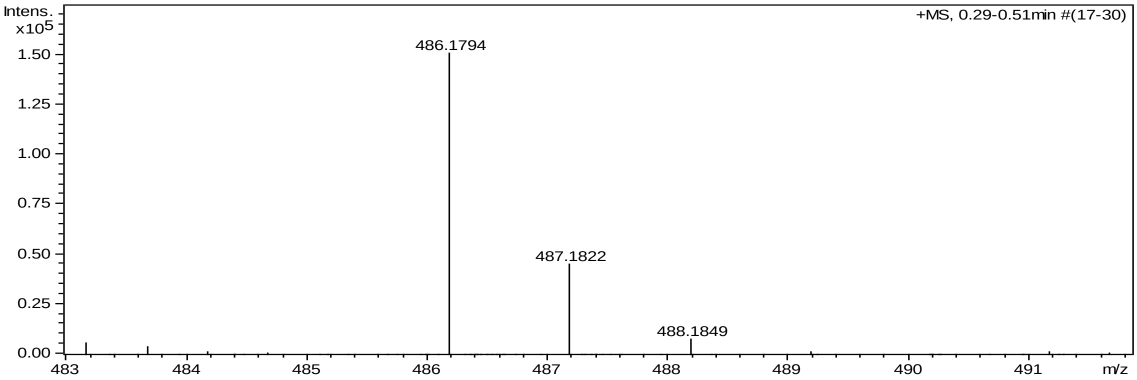

Intens.
x10^5
+MS, 0.29-0.51min #(17-30)
486.1794
487.1822
488.1849
m/z

1',3'-Dimethyl-2,4-di-*p*-tolyl-1,2,4,9-tetrahydro-2'*H*-spiro[carbazole-3,5'-pyrimidine]-2',4',6'(1'*H*,3'*H*)-trione (4b):

purple solid, 80%, m.p. 206-209 °C; ^{1}H NMR (400 MHz, $CDCl_3$) δ: 7.99 (s, 1H, NH), 7.28 (d, *J* = 8.4 Hz, 1H, ArH), 7.07-7.03 (m, 7H, ArH), 6.92 (d, *J* = 8.0 Hz, 1H, ArH), 6.82-6.78 (m, 2H, ArH), 6.43 (d, *J* = 8.0 Hz, 1H, ArH), 5.19 (s, 1H, CH), 4.09 (dd, J_1= 12.0 Hz, J_2= 5.6 Hz, 1H, CH), 3.87-3.79 (m, 1H, CH), 3.00 (dd, J_1= 16.8 Hz, J_2= 5.6 Hz, 1H, CH), 2.98 (s, 3H, CH_3), 2.82 (s, 3H, CH_3), 2.29 (s, 3H, CH_3), 2.28 (s, 3H, CH_3); ^{13}C NMR (400 MHz, $CDCl_3$) δ: 171.9, 167.9, 150.2, 137.7, 137.6, 136.0, 135.9, 134.6, 134.0, 129.4, 129.0, 128.9, 128.8, 128.7, 128.1, 126.3, 120.9, 119.7, 118.8, 110.5, 107.7, 62.6, 50.8, 47.7, 28.3, 27.9, 27.7, 21.1, 21.0; IR (KBr) υ: 3464, 3087, 2967, 1866, 1753, 1662, 1631, 1583, 1462, 1355, 1317, 1280, 1175, 961, 870 cm^{-1}; MS (*m*/*z*): HRMS (ESI) Calcd. for $C_{31}H_{29}N_3O_3$ ($[M+Na]^+$): 514.2101, found: 514.2106.

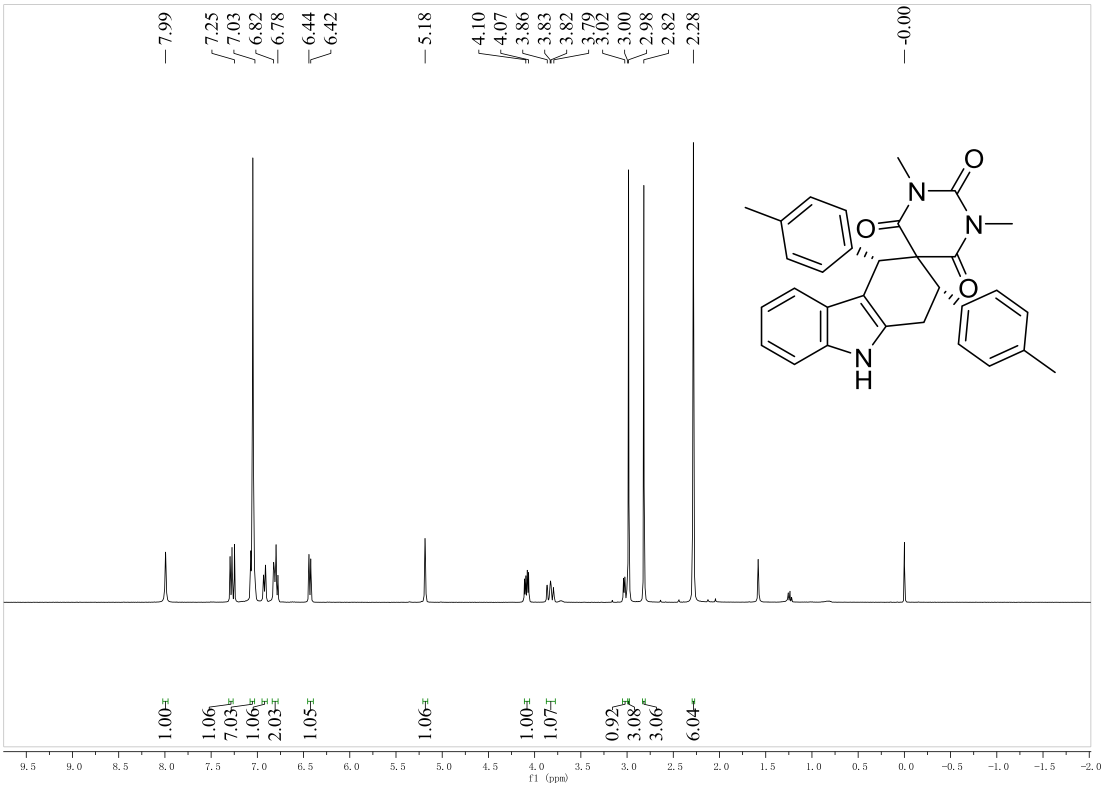

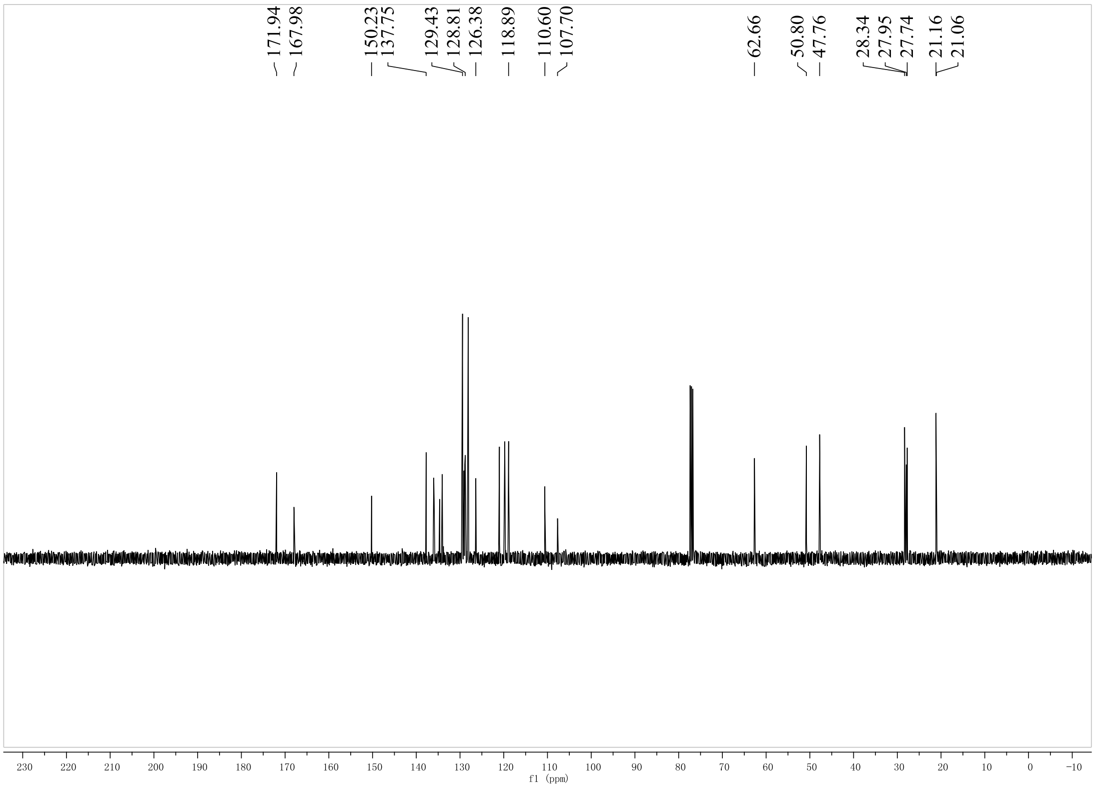
171.94
167.98
150.23
137.75
129.43
128.81
126.38
118.89
110.60
107.70
62.66
50.80
47.76
28.34
27.95
27.74
21.16
21.06
230 220 210 200 190 180 170 160 150 140 130 120 110 100 90 80 70 60 50 40 30 20 10 0 −10
f1 (ppm)

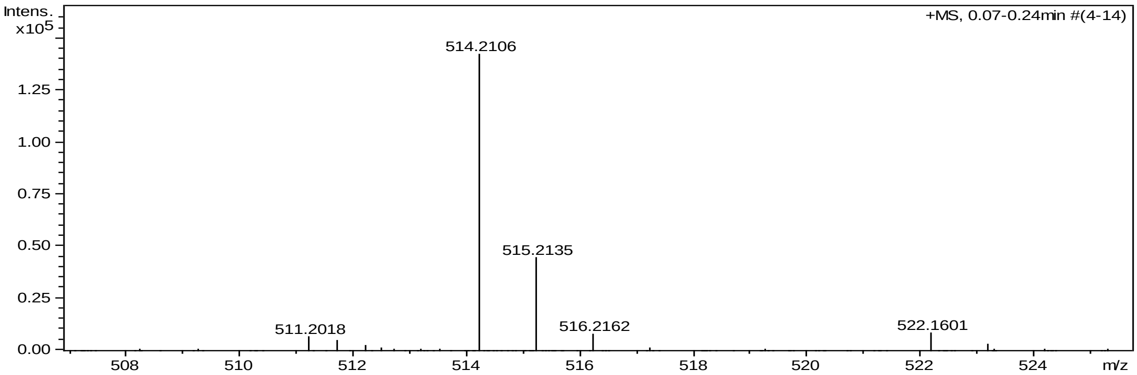
Intens.
x10[5]
+MS, 0.07-0.24min #(4-14)
514.2106
515.2135
511.2018
516.2162
522.1601
1.25
1.00
0.75
0.50
0.25
0.00
508 510 512 514 516 518 520 522 524 m/z

1',3'-Dimethyl-2,4-di-*o*-tolyl-1,2,4,9-tetrahydro-2'*H*-spiro[carbazole-3,5'-pyrimidine]-2',4',6'(1'*H*,3'*H*)-trione (4c):

purple solid, 75%, m.p. 212-214 °C; ^{1}H NMR (400 MHz, $CDCl_3$) δ: 8.01 (s, 1H, NH), 7.27 (d, *J* = 8.4 Hz, 1H, ArH), 7.20-7.13 (m, 3H, ArH), 7.13-7.09 (m, 2H, ArH), 7.06 (d, *J* = 7.2 Hz, 1H, ArH), 7.03 (t, *J* = 7.2 Hz, 1H, ArH), 6.96 (t, *J* = 7.2 Hz, 1H, ArH), 6.88 (d, *J* = 7.2 Hz, 1H, ArH), 6.73 (t, *J* = 7.2 Hz, 1H, ArH), 6.12 (d, *J* = 8.0 Hz, 1H, ArH), 5.47 (s, 1H, CH), 4.57 (dd, J_1= 12.0 Hz, J_2= 5.6 Hz, 1H, CH), 3.84-3.76 (m, 1H, CH), 3.04 (d, *J* = 5.6 Hz, 1H, CH), 3.01 (s, 3H, CH_3), 2.93 (s, 3H, CH_3), 2.48 (s, 3H, CH_3), 2.37 (s, 3H, CH_3); ^{13}C NMR (400 MHz, $CDCl_3$) δ: 171.2, 169.1, 150.3, 139.0, 137.1, 136.4, 136.0, 135.2, 135.1, 131.2, 130.7, 129.9, 128.0, 127.4, 126.7, 126.4, 125.6, 125.4, 121.1, 119.1, 119.1, 110.6, 108.5, 60.0, 48.3, 43.2, 29.5, 28.6, 28.1, 19.9, 19.5; IR (KBr) υ: 3466, 3010, 2953, 1937, 1841, 1678, 1655, 1572, 1469, 1361, 1310, 1255, 1169, 987, 863 cm^{-1}; MS (*m/z*): HRMS (ESI) Calcd. for $C_{31}H_{29}N_3O_3$ ($[M+Na]^+$): 514.2101, found: 514.2107.

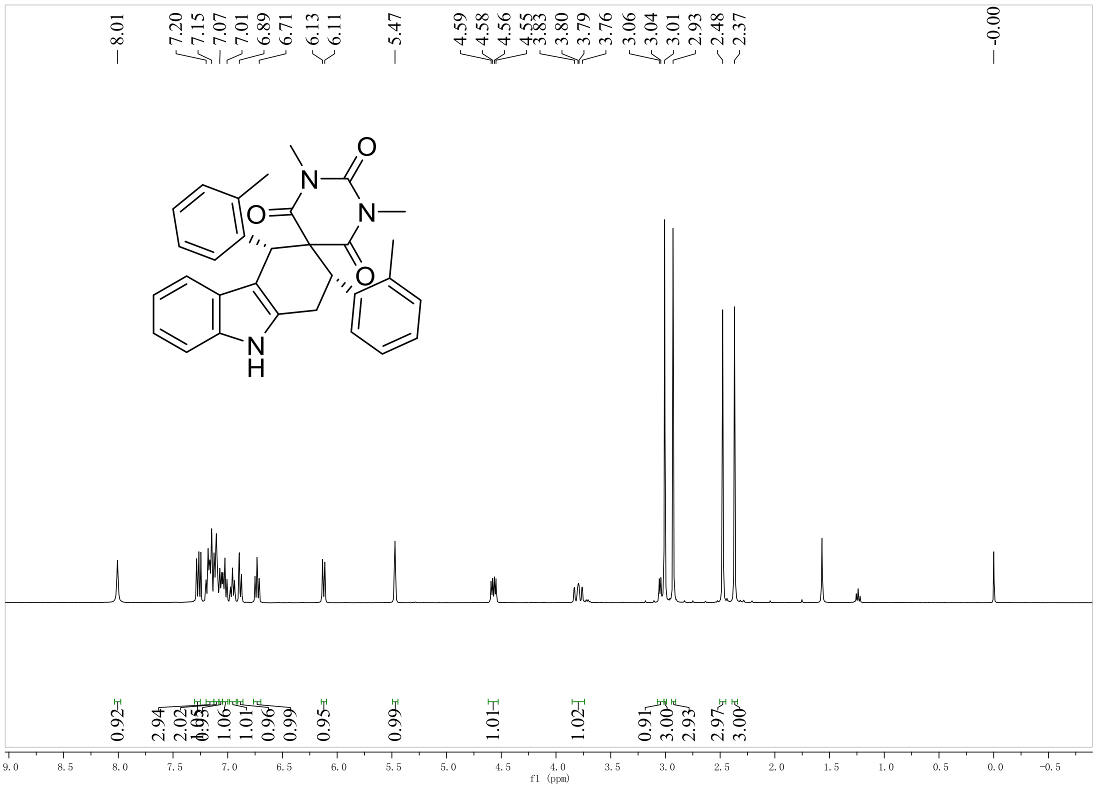

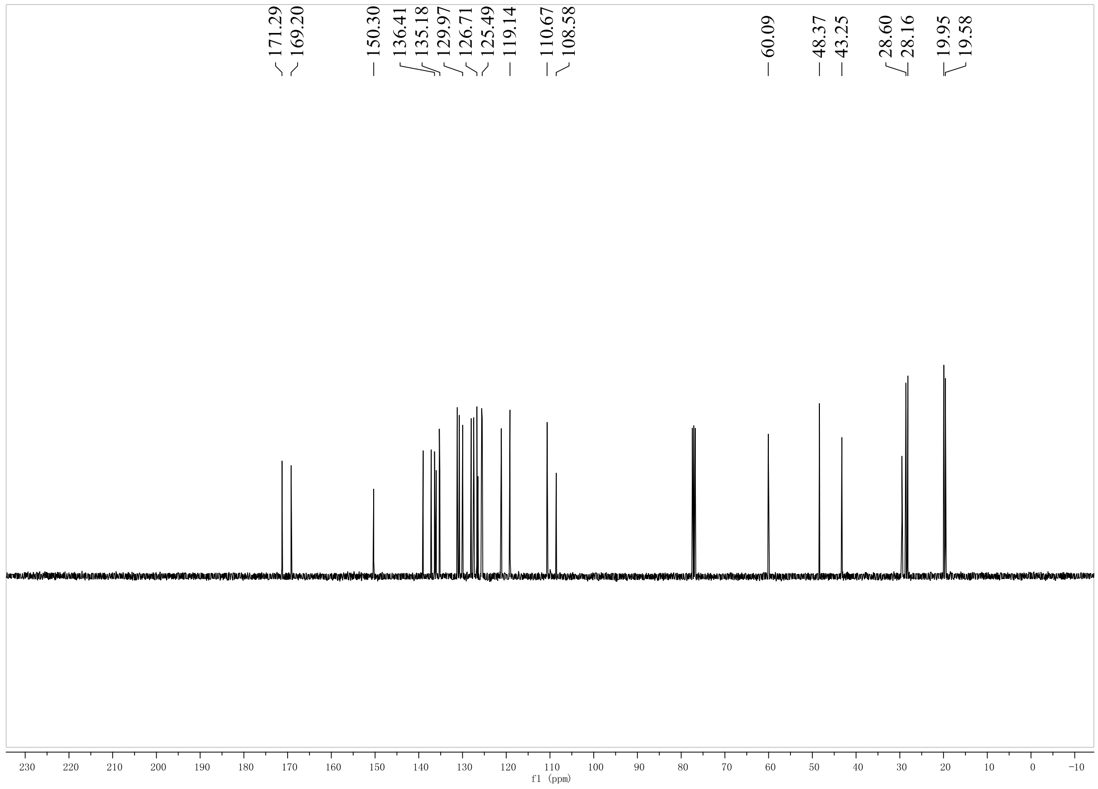

171.29
169.20
150.30
136.41
135.18
129.97
126.71
125.49
119.14
110.67
108.58
60.09
48.37
43.25
28.60
28.16
19.95
19.58
f1 (ppm)

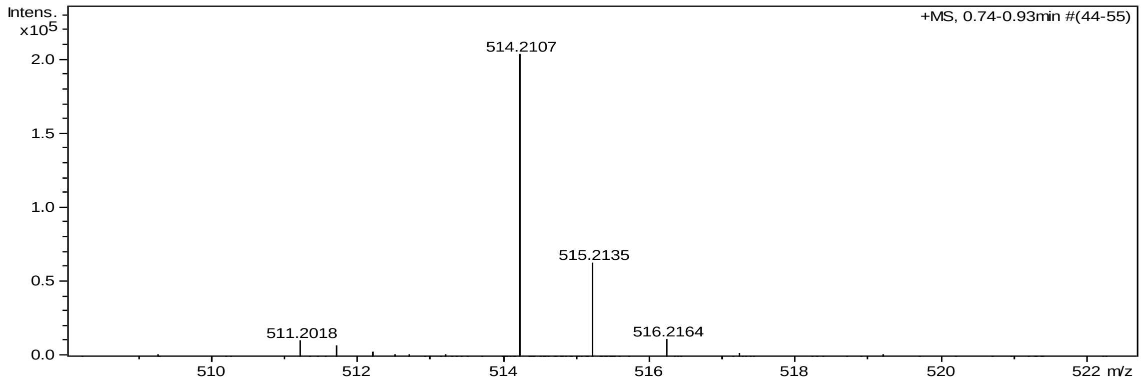

Intens.
x10^5
+MS, 0.74-0.93min #(44-55)
514.2107
515.2135
511.2018
516.2164
m/z

2,4-Bis(4-methoxyphenyl)-1',3'-dimethyl-1,2,4,9-tetrahydro-2'H-spiro[carbazole-3,5'-pyrimidine]-2',4',6'(1'H,3'H)-trione (4d):

purple solid, 78%, m.p. 213-215 °C; ^{1}H NMR (400 MHz, $CDCl_3$) δ: 8.01 (s, 1H, NH), 7.28 (d, J = 8.0 Hz, 1H, ArH), 7.10-7.04 (m, 4H, ArH), 6.85 (d, J = 8.8 Hz, 1H, ArH), 6.81 (d, J = 8.0 Hz, 1H, ArH), 6.78-6.76 (m, 3H, ArH), 6.45 (dd, J_1 = 8.8 Hz, J_2 = 2.0 Hz, 1H, ArH), 6.43 (d, J = 8.0 Hz, 1H, ArH), 5.17 (s, 1H, CH), 4.07 (dd, J_1 = 12.0 Hz, J_2 = 5.6 Hz, 1H, CH), 3.81 (d, J = 15.6 Hz, 1H, CH), 3.76 (s, 3H, OCH_3), 3.75 (s, 3H, OCH_3), 3.05 (s, 3H, CH_3), 2.97 (d, J = 5.6 Hz, 1H, CH), 2.85 (s, 3H, CH_3); ^{13}C NMR (400 MHz, $CDCl_3$) δ: 172.0, 168.0, 159.1, 159.1, 150.1, 136.0, 134.5, 130.8, 130.4, 129.9, 129.3, 129.1, 126.3, 120.9, 119.7, 118.9, 114.0, 113.7, 113.1, 110.5, 107.8, 62.9, 55.1, 55.1, 50.3, 47.3, 28.3, 28.0, 27.7; IR (KBr) υ: 3439, 3078, 2988, 1967, 1854, 1676, 1631, 1542, 1453, 1361, 1349, 1255, 1167, 987, 869 cm^{-1}; MS (m/z): HRMS (ESI) Calcd. for $C_{31}H_{29}N_3O_5$ ($[M+Na]^+$): 546.1999, found: 546.2001.

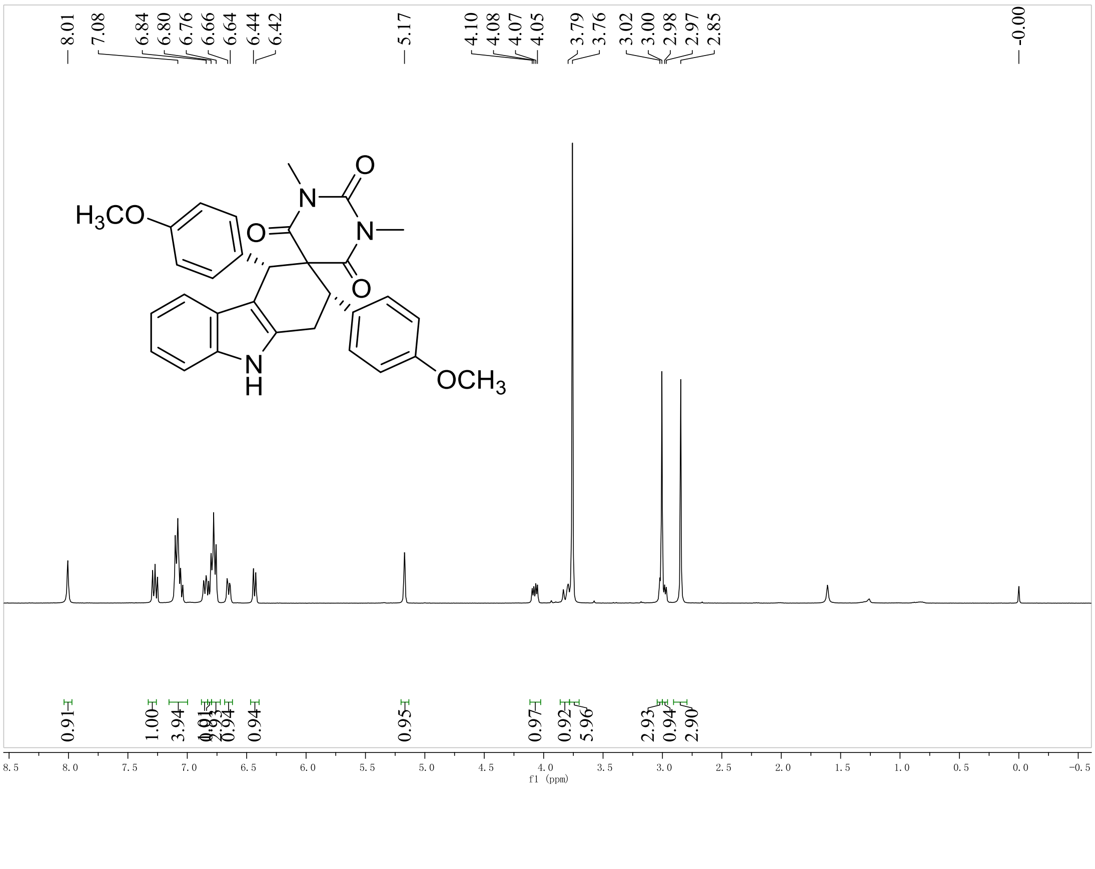

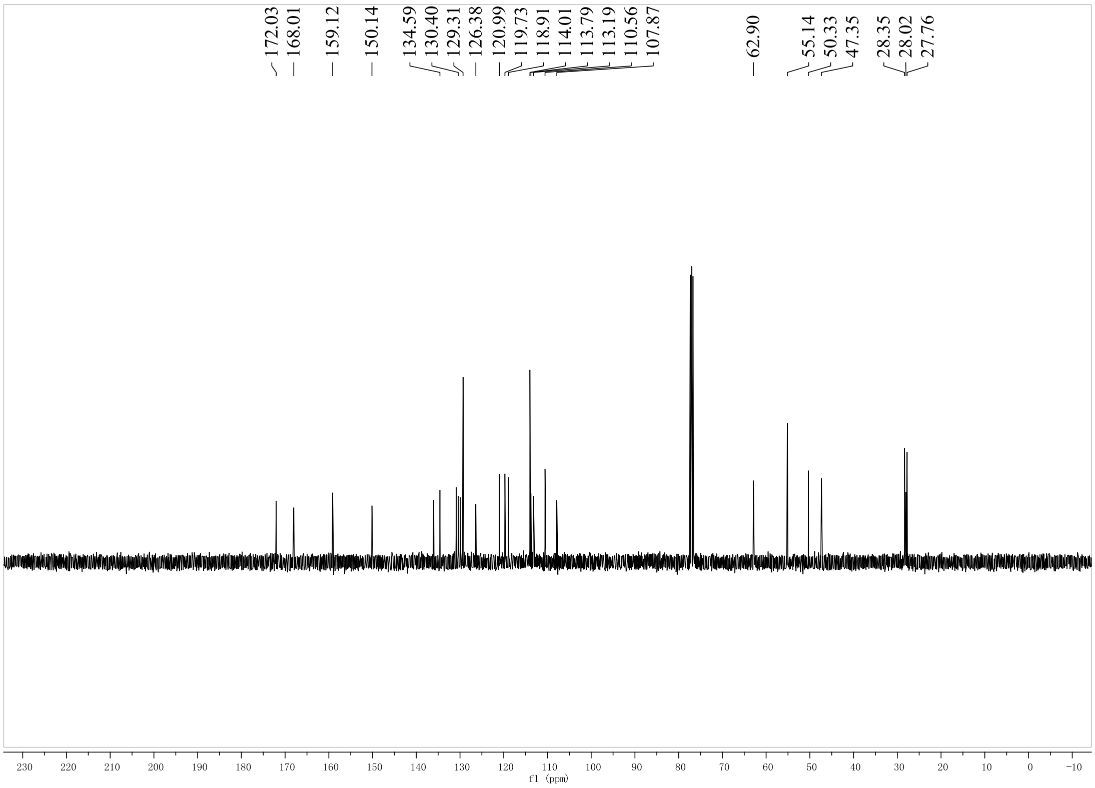

172.03
168.01
159.12
150.14
134.59
130.40
129.31
126.38
120.99
119.73
118.91
114.01
113.79
113.19
110.56
107.87
62.90
55.14
50.33
47.35
28.35
28.02
27.76
230 220 210 200 190 180 170 160 150 140 130 120 110 100 90 80 70 60 50 40 30 20 10 0 −10
f1 (ppm)

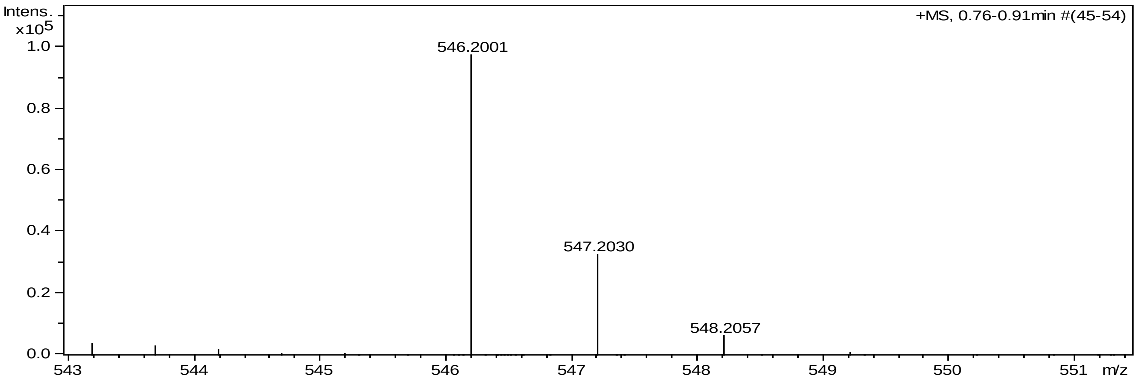

Intens.
x10^5
+MS, 0.76-0.91min #(45-54)
546.2001
547.2030
548.2057
1.0
0.8
0.6
0.4
0.2
0.0
543 544 545 546 547 548 549 550 551 m/z

2,4-Bis(3-methoxyphenyl)-1',3'-dimethyl-1,2,4,9-tetrahydro-2'*H*-spiro[carbazole-3,5'-pyrimidine]-2',4',6'(1'*H*,3'*H*)-trione (4e):

purple solid, 70%, m.p. 215-217 °C; ^{1}H NMR (400 MHz, $CDCl_3$) δ: 8.00 (s, 1H, NH), 7.28 (d, J = 8.4 Hz, 1H, ArH), 7.23-7.16 (m, 2H, ArH), 7.05-7.01 (m, 2H, ArH), 6.85 (d, J = 8.4 Hz, 2H, ArH), 6.82-6.75 (m, 3H, ArH), 6.72 (t, J = 7.6 Hz, 1H, ArH), 6.38 (d, J = 8.0 Hz, 1H, ArH), 5.62 (s, 1H, CH), 4.83 (dd, J_1 = 12.4 Hz, J_2 = 5.6 Hz, 1H, CH), 3.81 (s, 3H, OCH_3), 3.77 (s, 3H, OCH_3), 3.77-3.72 (m, 1H, CH), 3.04-2.99 (m, 1H, CH), 2.99 (s, 3H, CH_3), 2.84 (s, 3H, CH_3); ^{13}C NMR (400 MHz, $CDCl_3$) δ: 170.7, 169.1, 157.1, 157.0, 150.8, 136.0, 135.9, 131.5, 129.1, 128.9, 128.4, 127.1, 126.5, 125.3, 120.8, 120.8, 120.0, 119.5, 118.8, 111.3, 110.4, 109.9, 109.9, 107.8, 59.3, 55.7, 55.3, 44.0, 28.4, 28.3, 27.7; IR (KBr) υ: 3417, 3083, 2990, 1871, 1743, 1645, 1631, 1581, 1467, 1355, 1371, 1283, 1145, 879, 786 cm^{-1}; MS (m/z): HRMS (ESI) Calcd. for $C_{31}H_{29}N_3O_5$ ($[M+Na]^+$): 546.1999, found: 546.1992.

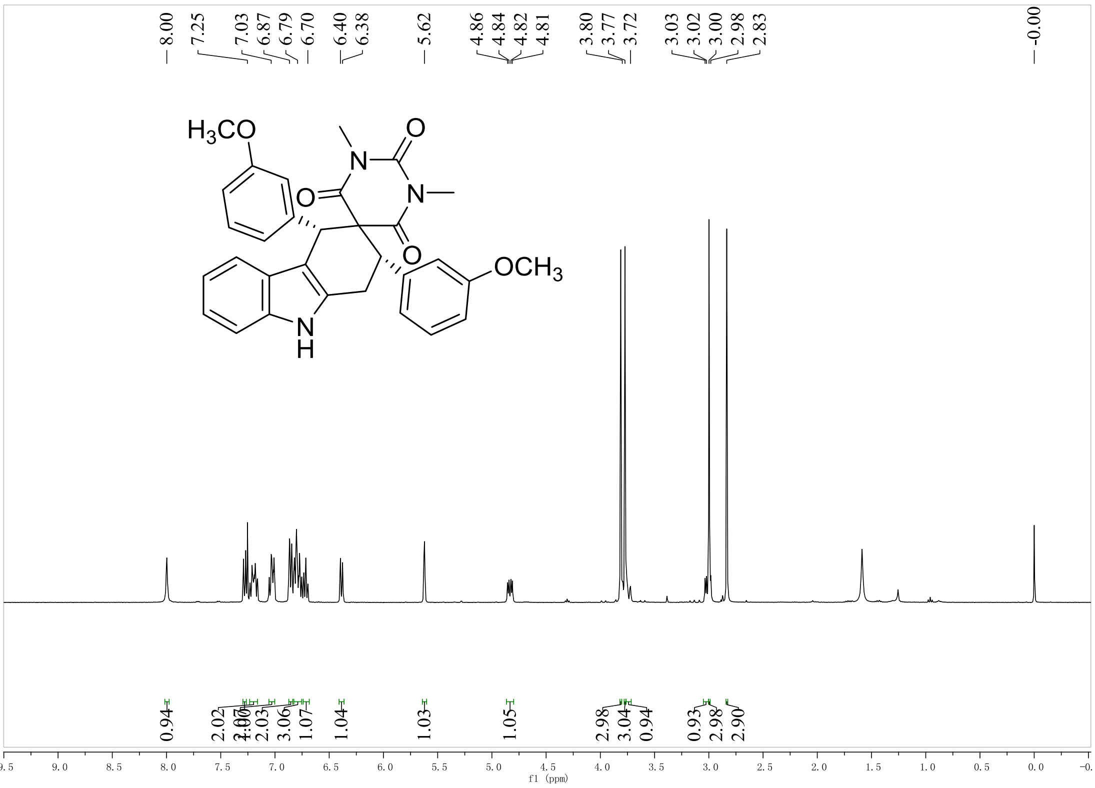

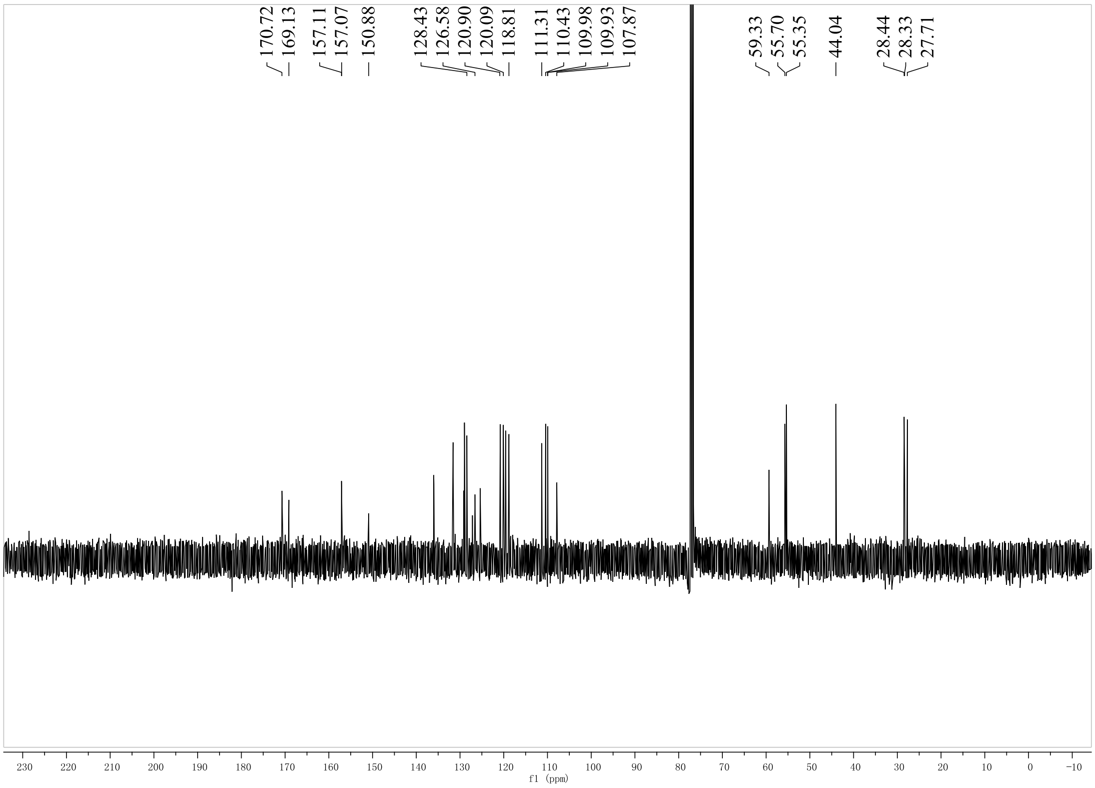
170.72
169.13
157.11
157.07
150.88
128.43
126.58
120.90
120.09
118.81
111.31
110.43
109.98
109.93
107.87
59.33
55.70
55.35
44.04
28.44
28.33
27.71
230 220 210 200 190 180 170 160 150 140 130 120 110 100 90 80 70 60 50 40 30 20 10 0 −10
f1 (ppm)

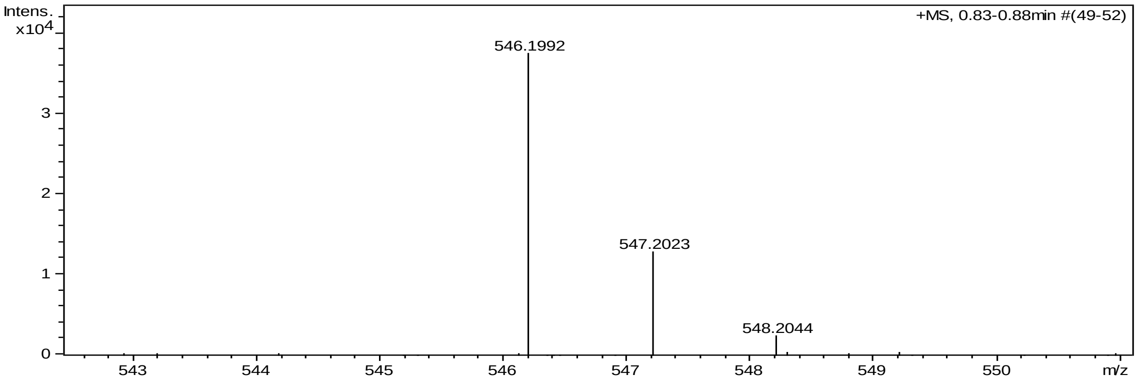
Intens.
x10^4
+MS, 0.83-0.88min #(49-52)
546.1992
547.2023
548.2044
0
1
2
3
543
544
545
546
547
548
549
550
m/z

2,4-Bis(4-chlorophenyl)-1',3'-dimethyl-1,2,4,9-tetrahydro-2'*H*-spiro[carbazole-3,5'-pyrimidine]-2',4',6'(1'*H*,3'*H*)-trione (4f):

purple solid, 75%, m.p. 203-205 °C; ^{1}H NMR (400 MHz, $CDCl_3$) δ: 8.07 (s, 1H, NH), 7.30 (d, *J* = 8.0 Hz, 1H, ArH), 7.28-7.25 (m, 1H, ArH), 7.25-7.23 (m, 1H, ArH), 7.23-7.22 (m, 1H, ArH), 7.09 (d, *J* = 8.4 Hz, 5H, ArH), 6.87 (dd, J_1 = 8.4 Hz, J_2 = 2.0 Hz, 1H, ArH), 6.83 (t, *J* = 7.6 Hz, 1H, ArH), 6.38 (d, *J* = 8.0 Hz, 1H, ArH), 5.20 (s, 1H, CH), 4.09 (dd, J_1 = 12.0 Hz, J_2 = 5.2 Hz, 1H, CH), 3.82-3.71 (m, 2H, CH_2), 3.02 (s, 3H, CH_3), 2.85 (s, 3H, CH_3); ^{13}C NMR (400 MHz, $CDCl_3$) δ: 171.4, 167.5, 149.7, 137.1, 136.0, 135.7, 134.3, 134.1, 134.0, 130.7, 130.1, 129.6, 129.0, 128.5, 128.4, 125.9, 121.3, 119.5, 119.2, 110.7, 106.9, 62.1, 50.3, 47.5, 28.5, 27.8, 27.7; IR (KBr) υ: 3304, 3113, 3081, 2967, 1746, 1655, 1613, 1569, 1482, 1366, 1312, 1276, 1158, 944, 863 cm^{-1}; MS (*m*/*z*): HRMS (ESI) Calcd. for $C_{29}H_{23}Cl_2{}^{35}N_3O_3$ ($[M+Na]^+$): 554.1009, found: 554.1001. $C_{29}H_{23}Cl_2{}^{37}N_3O_3$ ($[M+Na]^+$): 556.0979, found: 556.0981.

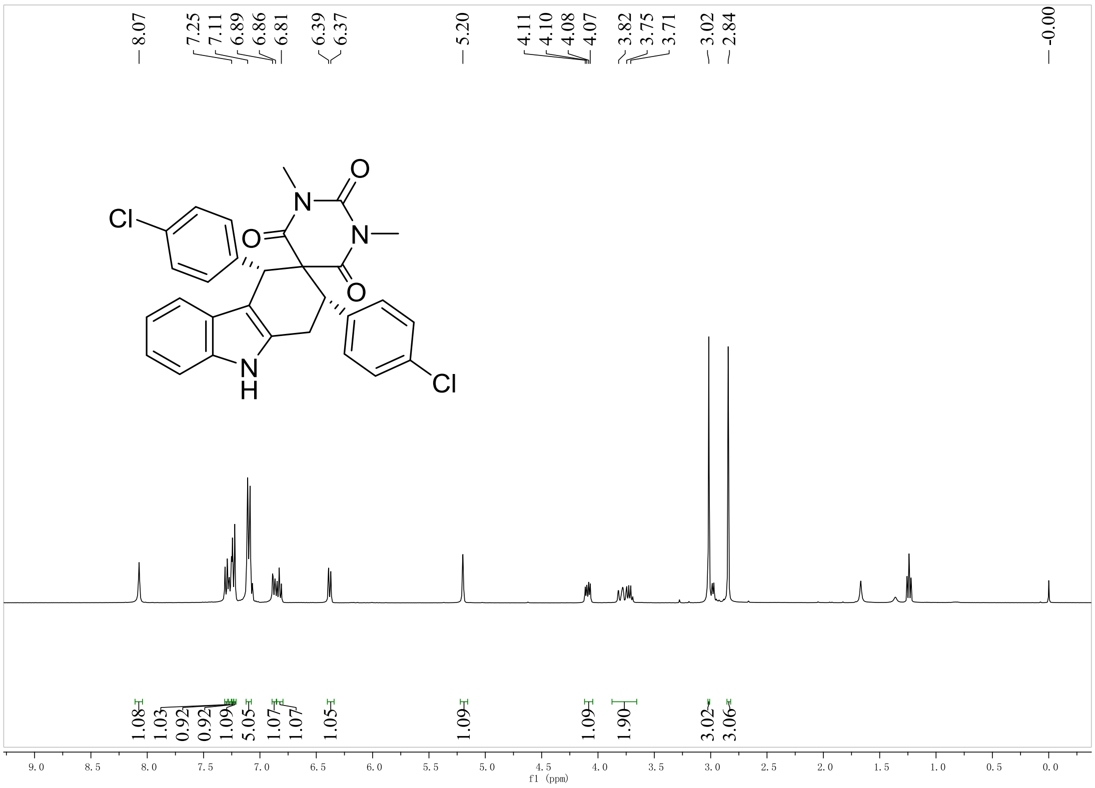

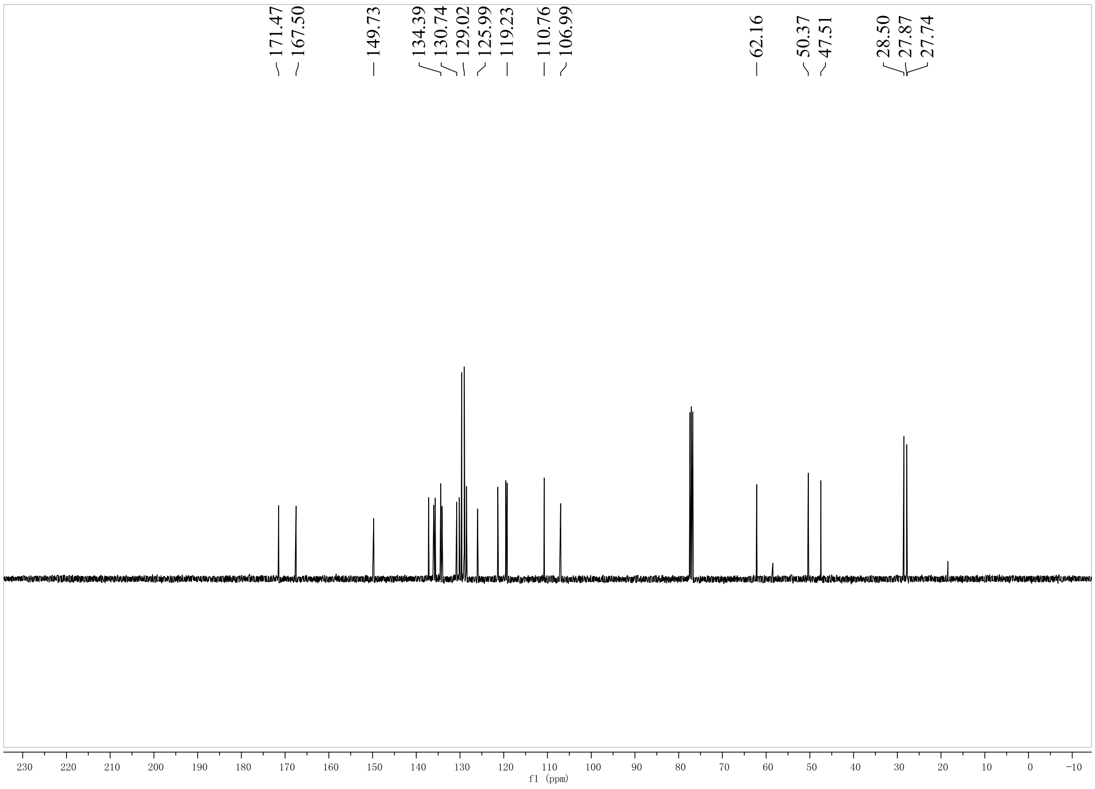
171.47
167.50
149.73
134.39
130.74
129.02
125.99
119.23
110.76
106.99
62.16
50.37
47.51
28.50
27.87
27.74
230 220 210 200 190 180 170 160 150 140 130 120 110 100 90 80 70 60 50 40 30 20 10 0 −10
f1 (ppm)

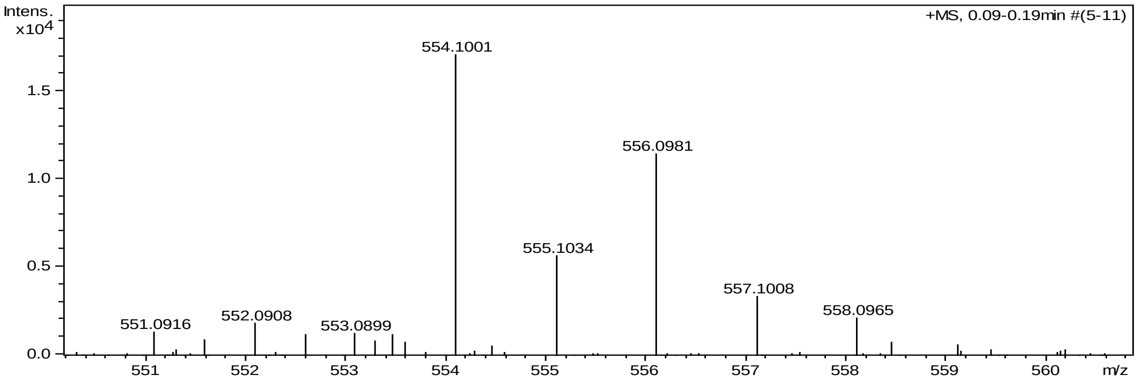
Intens.
x10^4
+MS, 0.09-0.19min #(5-11)
554.1001
556.0981
555.1034
557.1008
558.0965
551.0916
552.0908
553.0899
1.5
1.0
0.5
0.0
551 552 553 554 555 556 557 558 559 560 m/z

2,4-Bis(3-chlorophenyl)-1',3'-dimethyl-1,2,4,9-tetrahydro-2'*H*-spiro[carbazole-3,5'-pyrimidine]-2',4',6'(1'*H*,3'*H*)-trione (4g):

purple solid, 67%, m.p. 211-213 °C; ^{1}H NMR (400 MHz, $CDCl_3$) δ: 8.07 (s, 1H, NH), 7.42 (t, *J* = 7.6 Hz, 2H, ArH), 7.28 (d, *J* = 8.4 Hz, 1H, ArH), 7.24-7.20 (m, 1H, ArH), 7.17-7.12 (m, 3H, ArH), 7.04 (t, *J* = 8.4 Hz, 2H, ArH), 6.91 (dd, J_1 = 8.0 Hz, J_2 = 1.6 Hz, 1H, ArH), 6.76 (t, *J* = 7.6 Hz, 1H, ArH), 6.18 (d, *J* = 8.0 Hz, 1H, ArH), 5.71 (s, 1H, CH), 5.00 (dd, J_1 = 12.0 Hz, J_2 = 5.6 Hz, 1H, CH), 3.77 (t, *J* = 16.8 Hz, 1H, CH), 3.13 (dd, J_1 = 16.0 Hz, J_2 = 5.6 Hz, 1H, CH), 3.09 (s, 3H, CH_3), 2.93 (s, 3H, CH_3); ^{13}C NMR (400 MHz, $CDCl_3$) δ: 169.6, 168.8, 150.3, 138.5, 136.0, 135.4, 134.9, 134.5, 134.4, 131.8, 130.6, 129.6, 129.6, 128.6, 127.5, 126.5, 126.3, 126.0, 121.3, 119.2, 119.0, 110.7, 107.1, 58.9, 48.3, 43.1, 29.3, 28.6, 28.1; IR (KBr) υ: 3356, 3168, 3044, 2973, 1767, 1654, 1638, 1566, 1452, 1374, 1331, 1250, 1147, 967, 839 cm^{-1}; MS (*m*/*z*): HRMS (ESI) Calcd. for $C_{29}H_{23}Cl_2{}^{35}N_3O_3$ ([M+Na]$^+$): 554.1009, found: 554.1002. $C_{29}H_{23}Cl_2{}^{37}N_3O_3$ ([M+Na]$^+$): 556.0979, found: 556.0979.

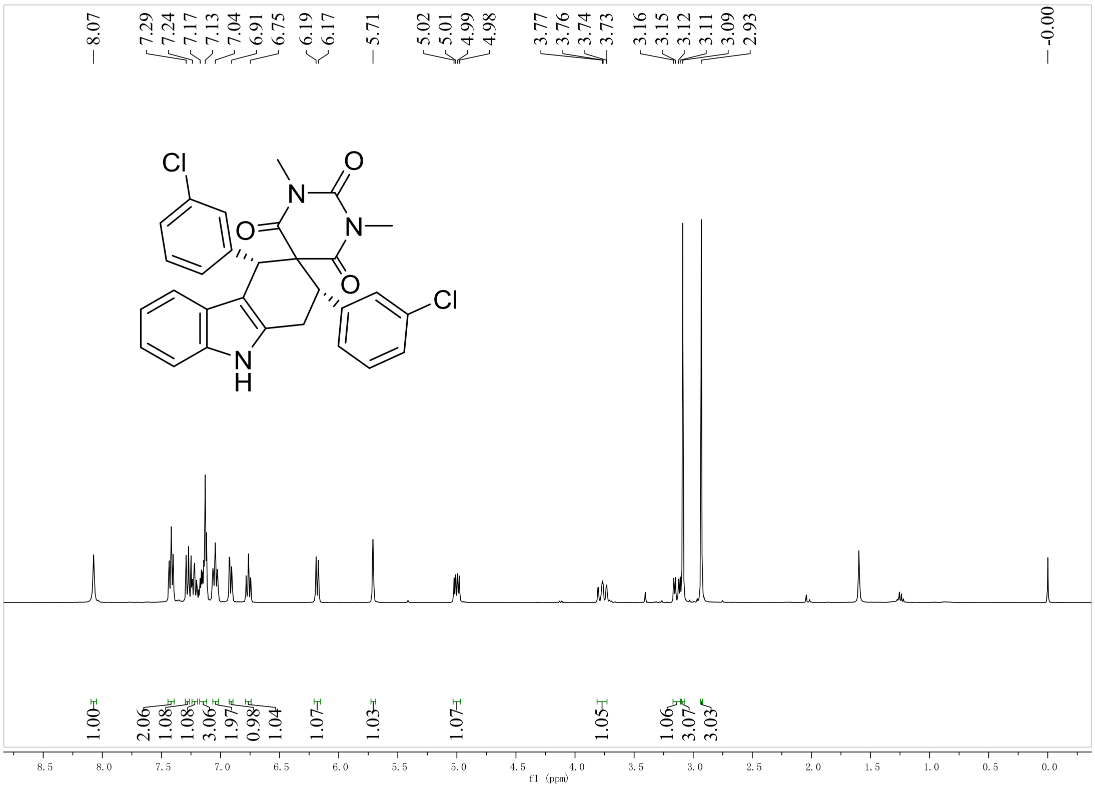

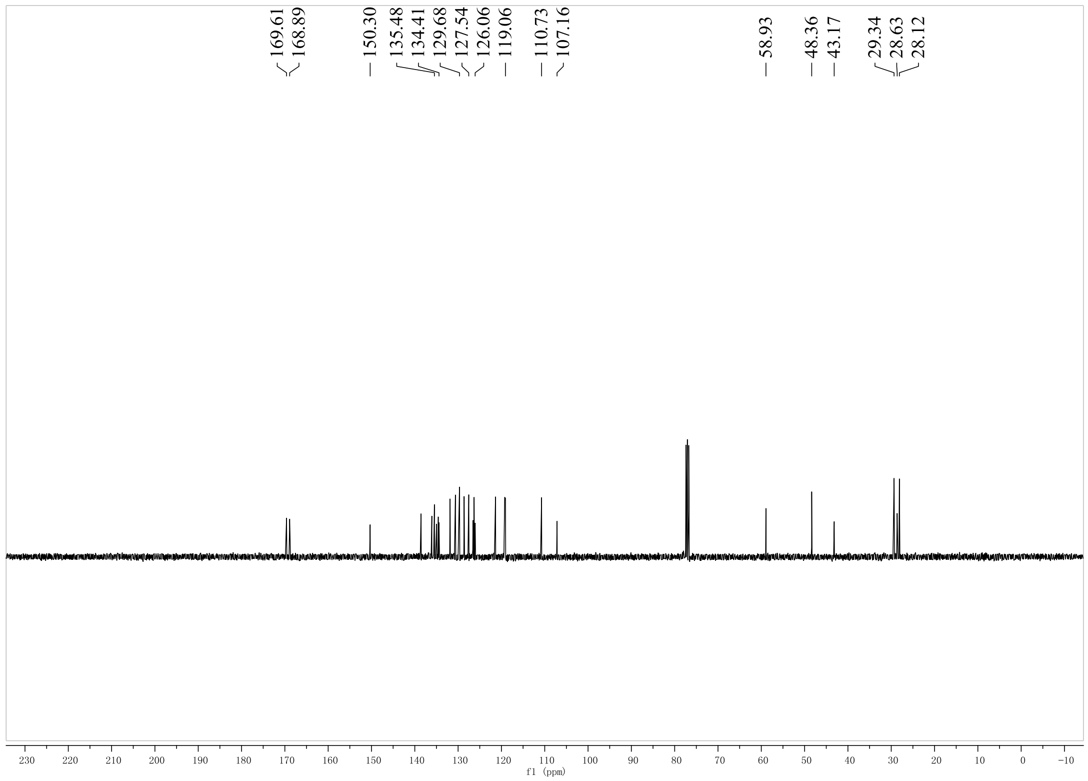
169.61
168.89
150.30
135.48
134.41
129.68
127.54
126.06
119.06
110.73
107.16
58.93
48.36
43.17
29.34
28.63
28.12
f1 (ppm)

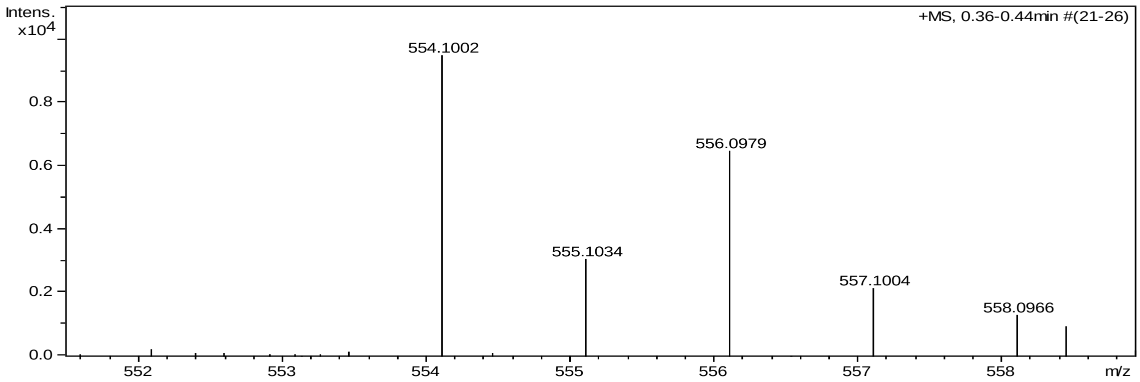
Intens.
x10^4
+MS, 0.36-0.44min #(21-26)
554.1002
556.0979
555.1034
557.1004
558.0966
m/z

2,4-Bis(4-fluorophenyl)-1',3'-dimethyl-1,2,4,9-tetrahydro-2'*H*-spiro[carbazole-3,5'-pyrimidine]-2',4',6'(1'*H*,3'*H*)-trione (4h):

purple solid, 62%, m.p. 214-216 °C; ^{1}H NMR (400 MHz, $CDCl_3$) δ: 8.03 (s, 1H, NH), 7.25 (d, *J* = 8.0 Hz, 1H, ArH), 7.14-7.05 (m, 4H, ArH), 6.98-6.90 (m, 4H, ArH), 6.83-6.78 (m, 2H, ArH), 6.37 (d, *J* = 8.0 Hz, 1H, ArH), 5.20 (s, 1H, CH), 4.08 (dd, J_1 = 12.0 Hz, J_2 = 5.6 Hz, 1H, CH), 3.79-3.72 (m, 1H, CH), 3.01 (s, 3H, CH_3), 2.93 (d, *J* = 5.6 Hz, 1H, CH), 2.84 (s, 3H, CH_3); ^{13}C NMR (400 MHz, $CDCl_3$) δ: 171.6, 167.6, 163.6, 163.5, 161.1, 161.0, 149.8, 136.0, 134.5, 134.5, 134.4, 133.0, 132.9, 131.1, 131.0, 130.4, 130.3, 129.9, 129.8, 126.1, 121.2, 119.5, 119.1, 115.8, 115.6, 115.4, 115.2, 115.0, 110.7, 107.3, 62.5, 50.2, 47.3, 28.4, 27.9, 27.8; IR (KBr) υ: 3403, 3162, 3079, 2977, 1781, 1663, 1640, 1531, 1442, 1338, 1308, 1271, 1165, 978, 834 cm^{-1}; MS (*m/z*): HRMS (ESI) Calcd. for $C_{29}H_{23}F_2N_3O_3$ ($[M+Na]^+$): 522.1600, found: 522.1601.

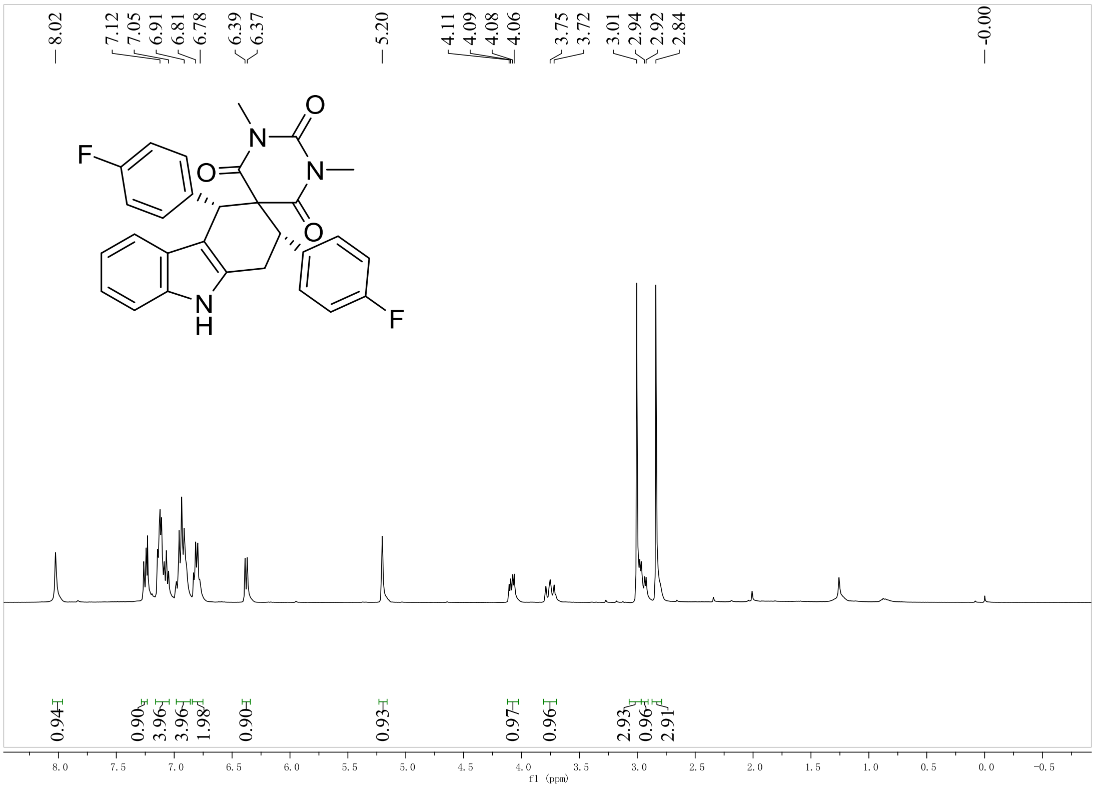

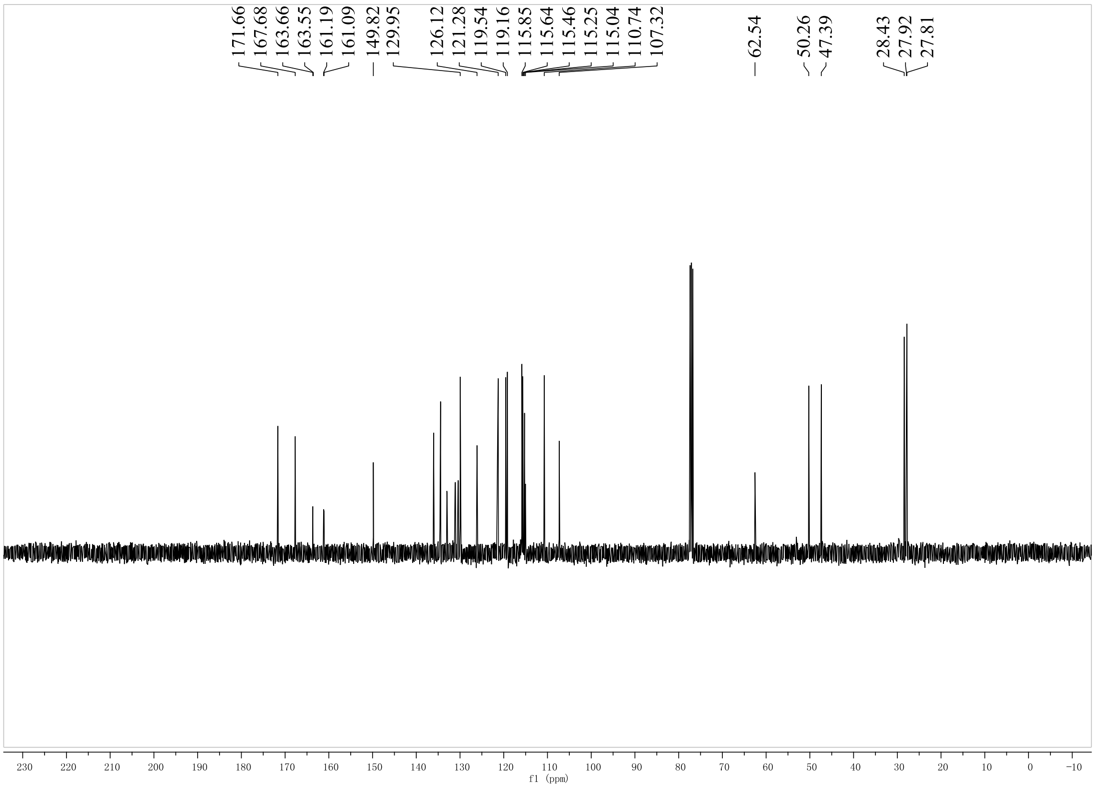
171.66
167.68
163.66
163.55
161.19
161.09
149.82
129.95
126.12
121.28
119.54
119.16
115.85
115.64
115.46
115.25
115.04
110.74
107.32
62.54
50.26
47.39
28.43
27.92
27.81
f1 (ppm)

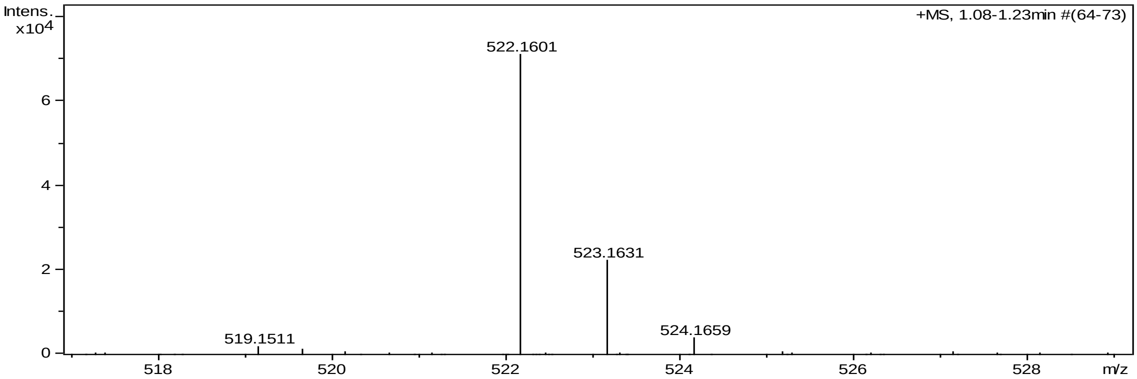
Intens.
x10^4
+MS, 1.08-1.23min #(64-73)
522.1601
523.1631
519.1511
524.1659
m/z

2,4-Diphenyl-1,2,4,9-tetrahydrospiro[carbazole-3,1'-cyclopentane]-2',5'-dione (5a):

purple solid, 74%, m.p. 178-180 °C; ^{1}H NMR (400 MHz, $CDCl_3$) δ: 8.02 (s, 1H, NH), 7.30 (d, *J* = 8.4 Hz, 1H, ArH), 7.28-7.27 (m, 2H, ArH), 7.26-7.25 (m, 1H, ArH), 7.22 (d, *J* = 8.0 Hz, 1H, ArH), 7.19-7.17 (m, 2H, ArH), 7.14 (t, *J* = 7.6 Hz, 1H, ArH), 7.06 (t, *J* = 7.2 Hz, 1H, ArH), 6.92 (d, *J* = 8.0 Hz, 1H, ArH), 6.79 (t, *J* = 7.2 Hz, 1H, ArH), 6.39 (d, *J* = 8.0 Hz, 1H, ArH), 4.87 (s, 1H, CH), 3.97-3.90 (m, 1H, CH), 3.66 (dd, J_1 = 12.4 Hz, J_2 = 4.8 Hz, 1H, CH), 2.95 (dd, J_1 = 16.0 Hz, J_2 = 4.8 Hz, 1H, CH), 2.01-1.90 (m, 2H, CH_2), 1.39-1.31 (m, 1H, CH), 1.26-1.22 (m, 1H, CH); ^{13}C NMR (400 MHz, $CDCl_3$) δ: 218.2, 216.7, 139.1, 137.8, 136.1, 134.7, 129.9, 129.5, 128.8, 128.7, 128.5, 128.2, 127.8, 127.5, 126.3, 121.0, 119.7, 118.9, 110.7, 107.8, 65.9, 48.8, 47.4, 37.3, 36.5, 26.5; IR (KBr) υ: 3200, 3173，3064, 2978, 1861, 1745, 1677, 1631, 1567, 1467, 1382, 1311, 1254, 1132, 960， 841, 781 cm^{-1}; MS (*m/z*): HRMS (ESI) Calcd. for $C_{28}H_{23}NO_2$ ([M+Na]$^+$): 428.1621, found: 428.1626.

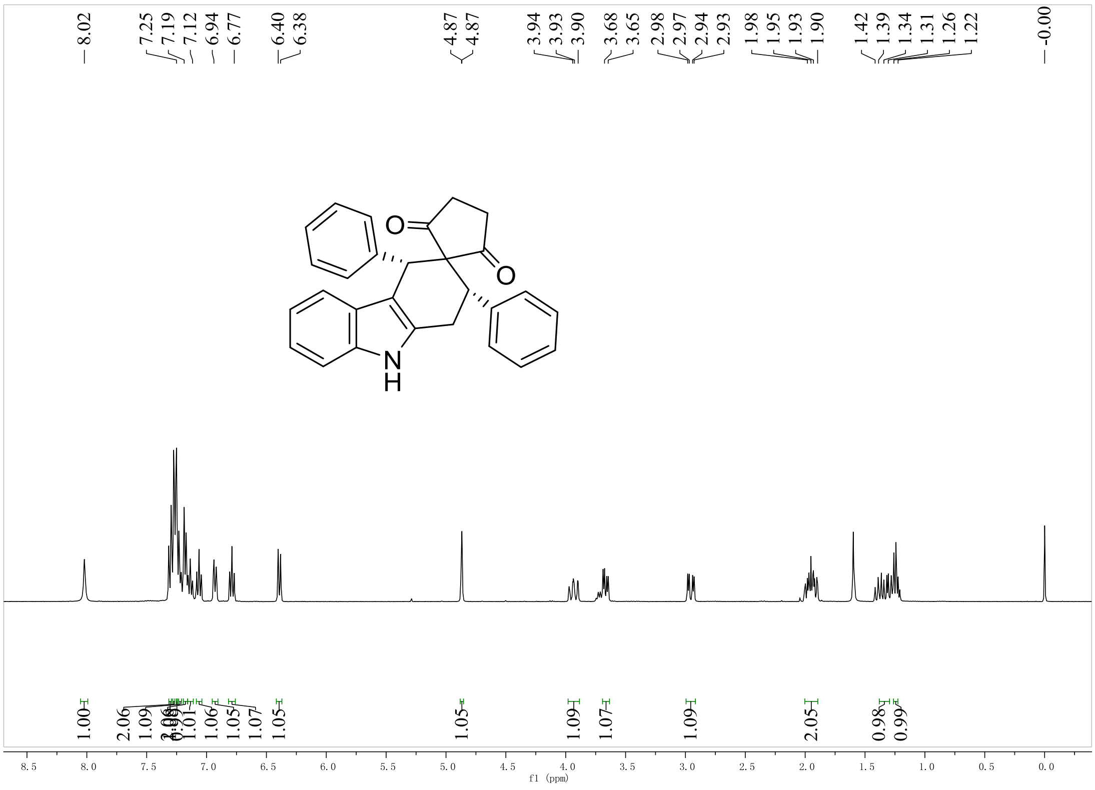

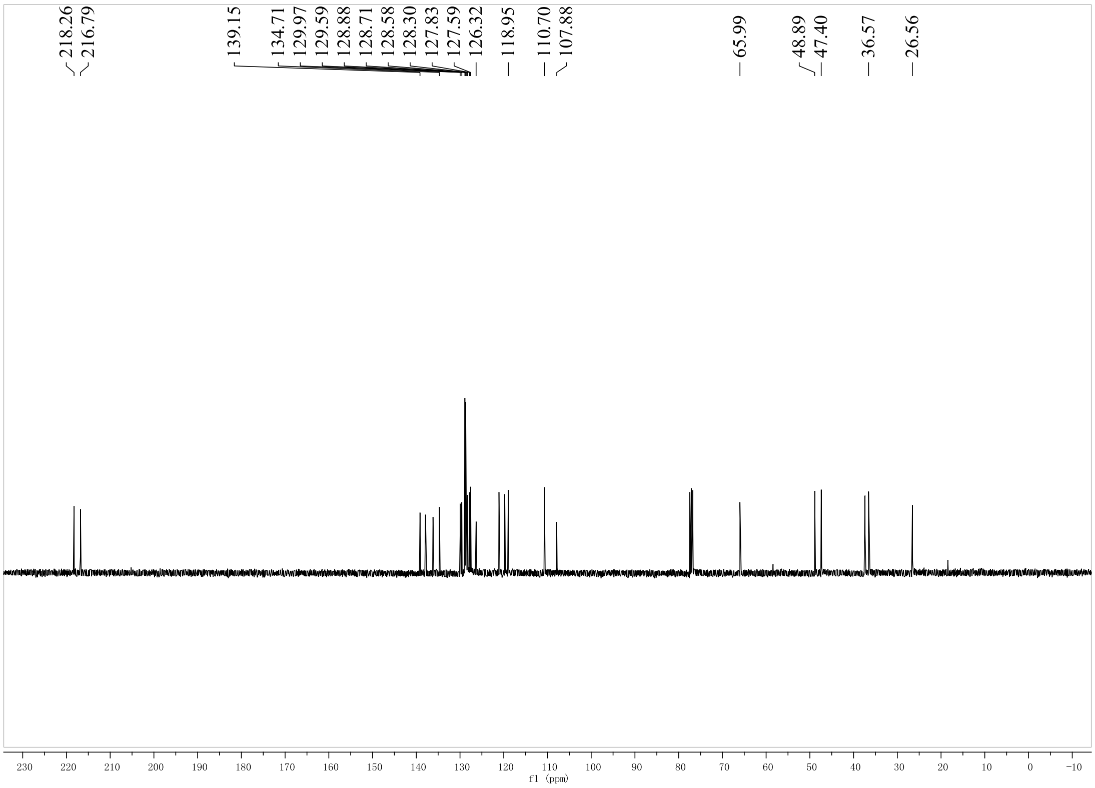
218.26
216.79
139.15
134.71
129.97
129.59
128.88
128.71
128.58
128.30
127.83
127.59
126.32
118.95
110.70
107.88
65.99
48.89
47.40
36.57
26.56
230
220
210
200
190
180
170
160
150
140
130
120
110
100
90
80
70
60
50
40
30
20
10
0
−10
f1 (ppm)

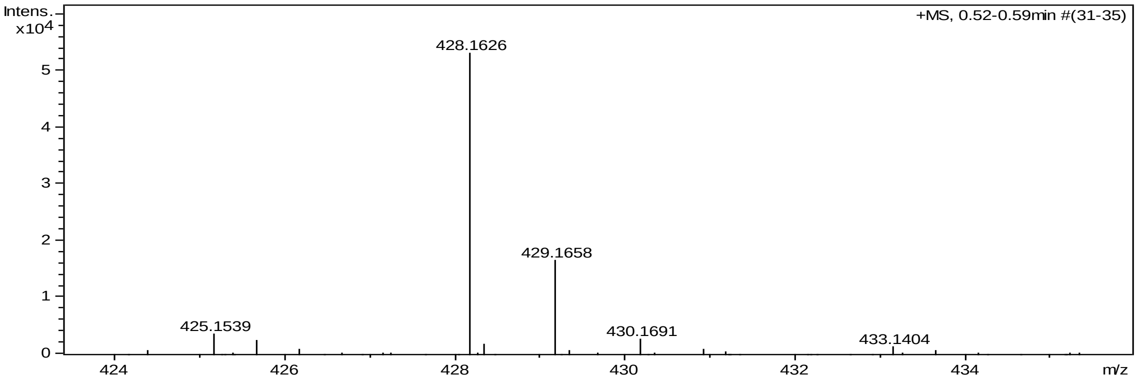
Intens.
x10^4
+MS, 0.52-0.59min #(31-35)
428.1626
429.1658
425.1539
430.1691
433.1404
0
1
2
3
4
5
424
426
428
430
432
434
m/z

2,4-Di-p-tolyl-1,2,4,9-tetrahydrospiro[carbazole-3,1'-cyclopentane]-2',5'-dione (5b):

purple solid, 78%, m.p. 176-178 ºC; ^{1}H NMR (400 MHz, $CDCl_3$) δ: 7.99 (s, 1H, NH), 7.28 (d, *J* = 8.4 Hz, 1H, ArH), 7.13 (dd, J_1 = 7.6 Hz, J_2 = 1.2 Hz, 1H, ArH), 7.08-7.06 (m, 5H, ArH), 7.05-7.03 (m, 1H, ArH), 6.93 (d, *J* = 8.0 Hz, 1H, ArH), 6.79 (t, *J* = 7.2 Hz, 2H, ArH), 6.42 (d, *J* = 8.0 Hz, 1H, ArH), 4.82 (s, 1H, CH), 3.93-3.85 (m, 1H, CH), 3.61 (dd, J_1 = 12.4 Hz, J_2 = 5.2 Hz, 1H, CH), 2.91 (dd, J_1 = 16.4 Hz, J_2 = 5.2 Hz, 1H, CH), 2.28 (s, 3H, CH_3), 2.27 (s, 3H, CH_3), 2.01-1.91 (m, 2H, CH_2), 1.43-1.27 (m, 2H, CH_2); ^{13}C NMR (400 MHz, $CDCl_3$) δ: 218.3, 216.8, 137.3, 137.0, 136.1, 134.7, 134.6, 129.7, 129.4, 129.3, 129.3, 128.7, 128.5, 126.4, 121.0, 119.8, 118.8, 110.5, 108.2, 66.0, 48.3, 46.9, 37.4, 36.6, 26.7, 21.1, 20.9; IR (KBr) υ: 3213, 3172，3063, 2956, 1876, 1732, 1654, 1632, 1578, 1456, 1367, 1257, 1237, 1169, 899, 763 cm^{-1}; MS (*m*/*z*): HRMS (ESI) Calcd. for $C_{30}H_{27}NO_2$ ($[M+Na]^+$): 456.1934, found: 456.1936.

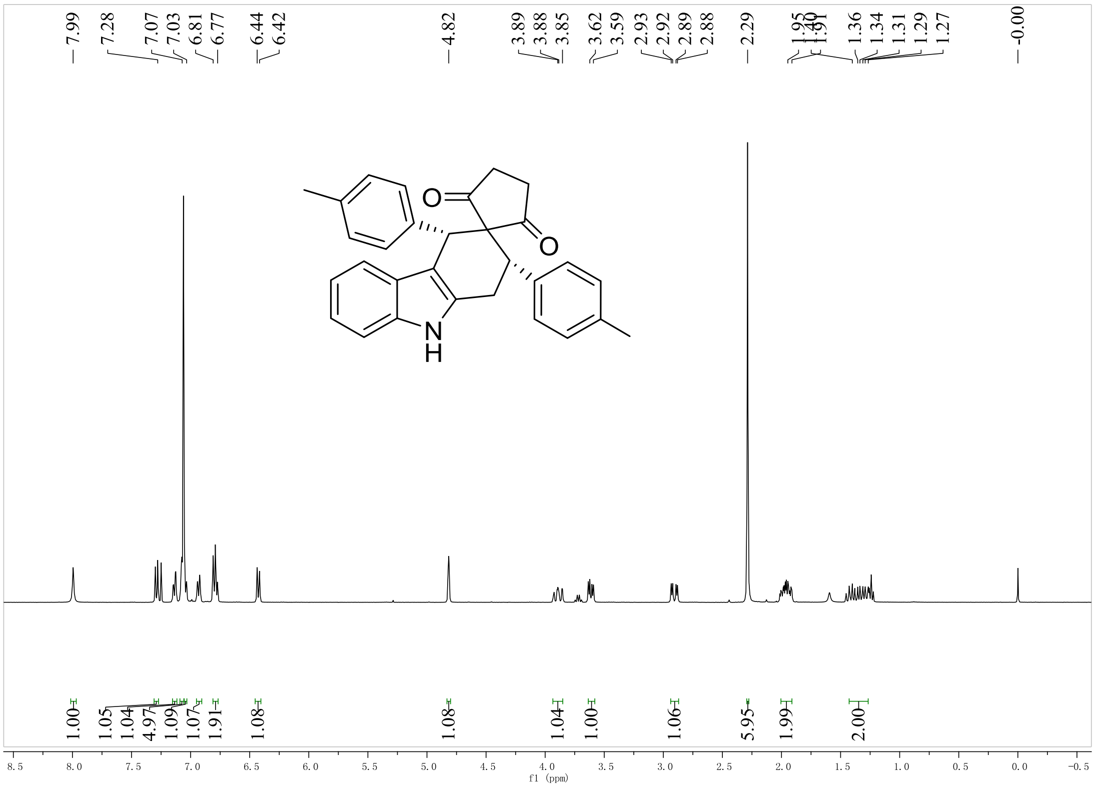

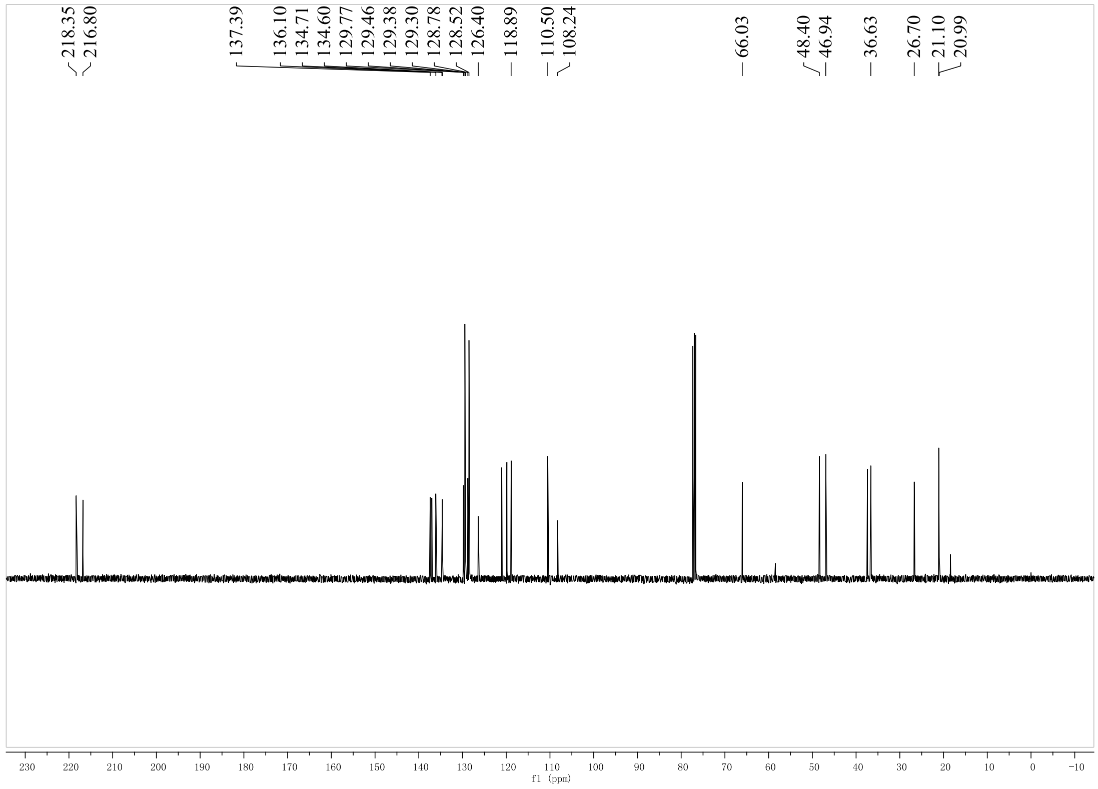

218.35
216.80
137.39
136.10
134.71
134.60
129.77
129.46
129.38
129.30
128.78
128.52
126.40
118.89
110.50
108.24
66.03
48.40
46.94
36.63
26.70
21.10
20.99
f1 (ppm)

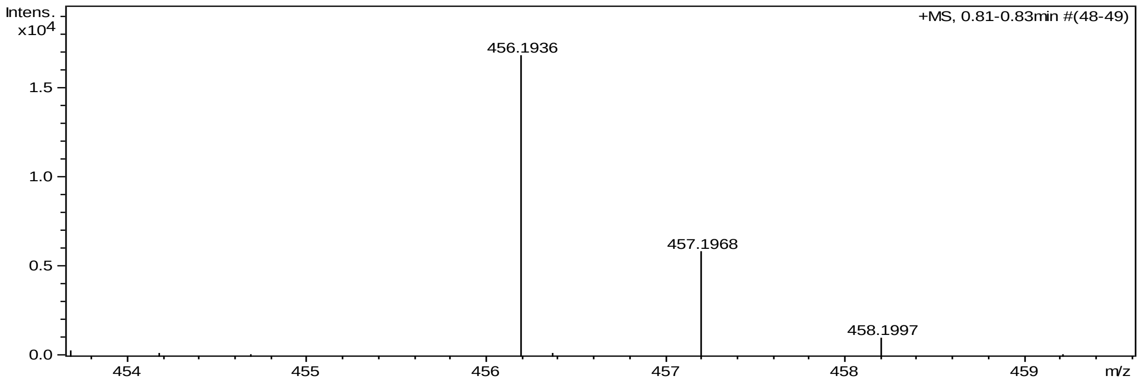

Intens.
x10^4
+MS, 0.81-0.83min #(48-49)
456.1936
457.1968
458.1997
m/z

2,4-Bis(4-(dimethylamino)phenyl)-1,2,4,9-tetrahydrospiro[carbazole-3,1'-cyclopentane]-2',5'-dione (5c):

purple solid, 73%, m.p. 180-182 ºC; ^{1}H NMR (400 MHz, $CDCl_3$) δ: 7.98 (s, 1H, NH), 7.28 (d, J = 8.0 Hz, 1H, ArH), 7.11 (dd, J_1 = 8.4 Hz, J_2 = 2.0 Hz, 1H, ArH), 7.06-7.03 (m, 3H, ArH), 6.78 (q, J = 7.6 Hz, 2H, ArH), 6.60 (d, J = 8.4 Hz, 3H, ArH), 6.50 (d, J = 8.0 Hz, 2H, ArH), 4.76 (s, 1H, CH), 3.90-3.83 (m, 1H, CH), 3.56 (dd, J_1 = 12.0 Hz, J_2 = 5.2 Hz, 1H, CH), 2.90 (s, 3H, CH_3), 2.89 (s, 3H, CH_3), 2.88 (s, 3H, CH_3), 2.87 (s, 3H, CH_3), 2.86 (d, J = 5.2 Hz, 1H, CH), 2.02-1.94 (m, 2H, CH_2), 1.50-1.36 (m, 2H, CH_2); ^{13}C NMR (400 MHz, $CDCl_3$) δ: 219.1, 217.5, 136.0, 134.7, 130.5, 130.1, 129.2, 126.6, 120.8, 120.0, 118.7, 112.5, 110.3, 108.7, 66.2, 47.8, 46.3, 40.5, 40.4, 37.4, 36.6, 26.8; IR (KBr) υ: 3217, 3189, 3046, 2983, 2817, 2156, 1833, 1678, 1611, 1534, 1429, 1351, 1269, 1138, 1131, 962, 888, 817, 764 cm^{-1}; MS (m/z): HRMS (ESI) Calcd. for $C_{32}H_{33}N_3O_2$ ($[M+Na]^+$): 514.2465, found: 514.2462.

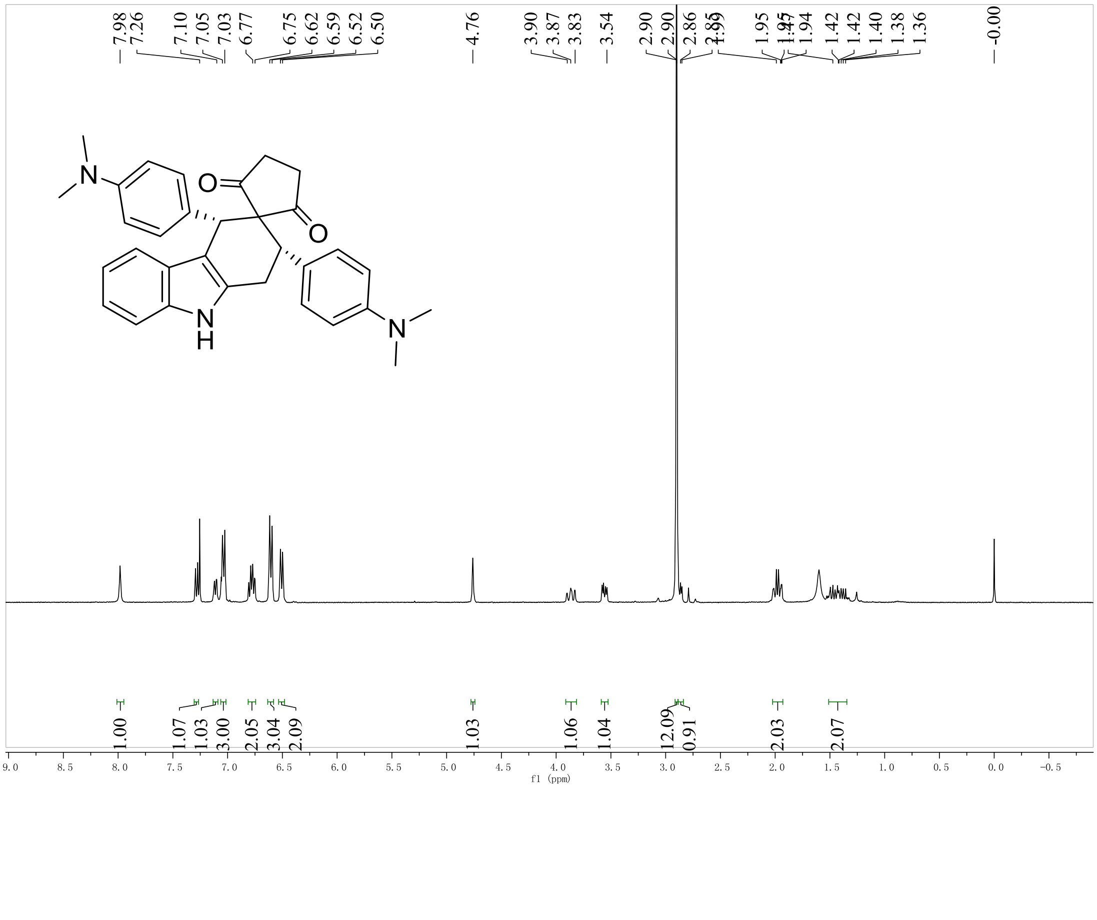

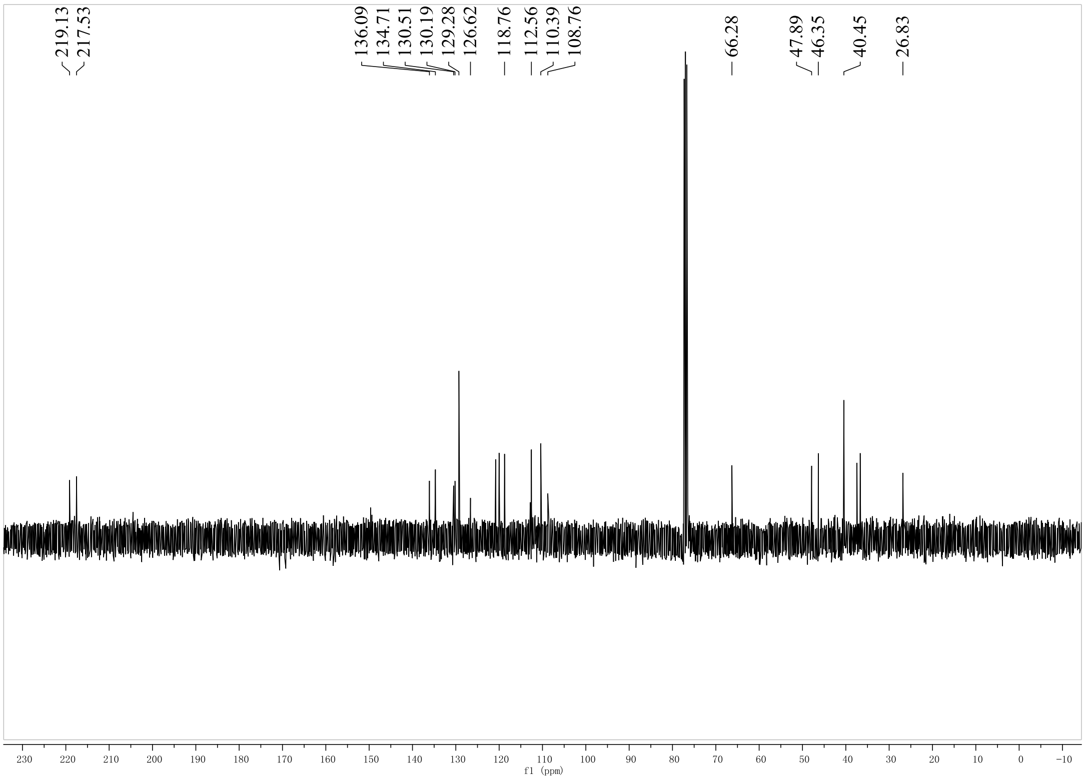
219.13
217.53
136.09
134.71
130.51
130.19
129.28
126.62
118.76
112.56
110.39
108.76
66.28
47.89
46.35
40.45
26.83
f1 (ppm)

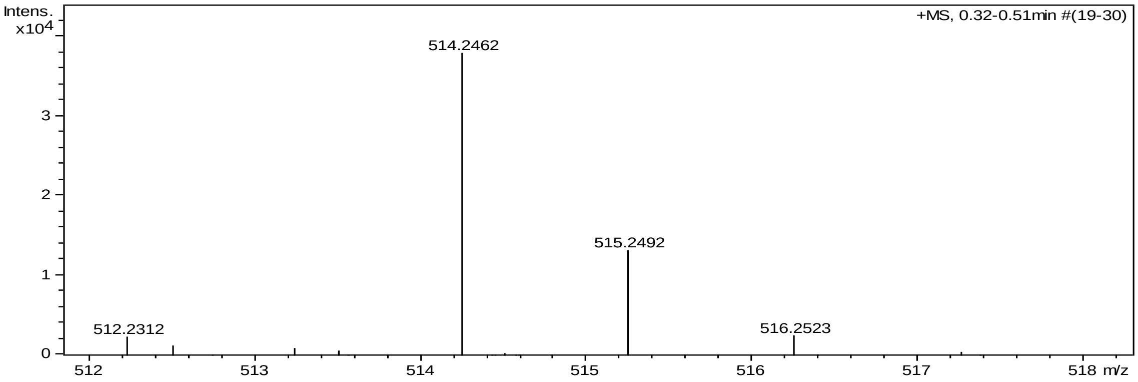
Intens.
x10^4
+MS, 0.32-0.51min #(19-30)
514.2462
515.2492
512.2312
516.2523
m/z

2,4-Bis(3-nitrophenyl)-1,2,4,9-tetrahydrospiro[carbazole-3,1'-cyclopentane]-2',5'-dione (5d): purple solid, 59%, m.p. 190-192 °C; ^{1}H NMR (400 MHz, $CDCl_3$) δ: 8.19 (s, 1H, NH), 8.15-8.13 (m, 2H, ArH), 8.04-7.97 (m, 1H, ArH), 7.75-7.58 (m, 1H, ArH), 7.58-7.42 (m, 3H, ArH), 7.36-7.26 (m, 2H, ArH), 7.13-7.06 (m, 1H, ArH), 6.83-6.77 (m, 1H, ArH), 6.26 (t, J = 8.4 Hz, 1H, ArH), 5.00 (s, 1H, CH), 3.97-3.79 (m, 2H, CH_2), 3.30 (dd, J_1 = 16.4 Hz, J_2 = 3.6 Hz, 1H, CH), 2.09-1.98 (m, 2H, CH_2), 1.55-1.34 (m, 2H, CH_2); ^{13}C NMR (400 MHz, $CDCl_3$) δ: 216.4, 215.2, 148.4, 148.0, 140.8, 139.9, 136.3, 136.2, 136.1, 135.3, 135.0, 134.6, 134.1, 130.1, 129.7, 129.5, 125.5, 124.8, 123.9, 123.4, 123.1, 123.0, 122.9, 121.7, 119.6, 119.5, 119.2, 118.8, 111.1, 110.9, 106.6, 65.1, 48.4, 46.8, 37.2, 36.3, 26.3; IR (KBr) υ: 3218, 3178, 3049, 2967, 2867, 2133, 1871, 1649, 1600, 1548, 1467, 1358, 1261, 1180, 1135, 997, 941, 878, 749 cm^{-1}; MS (m/z): HRMS (ESI) Calcd. for $C_{28}H_{21}N_3O_6$ ($[M+Na]^+$): 518.1323, found: 518.1326.

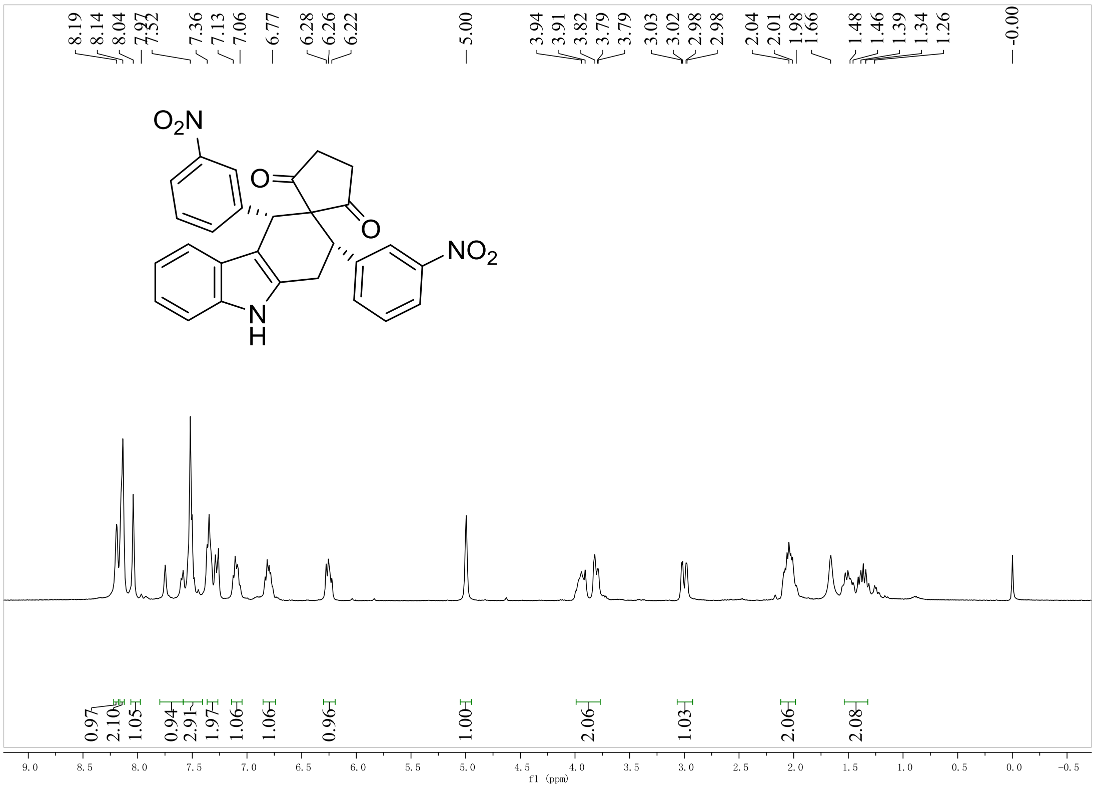

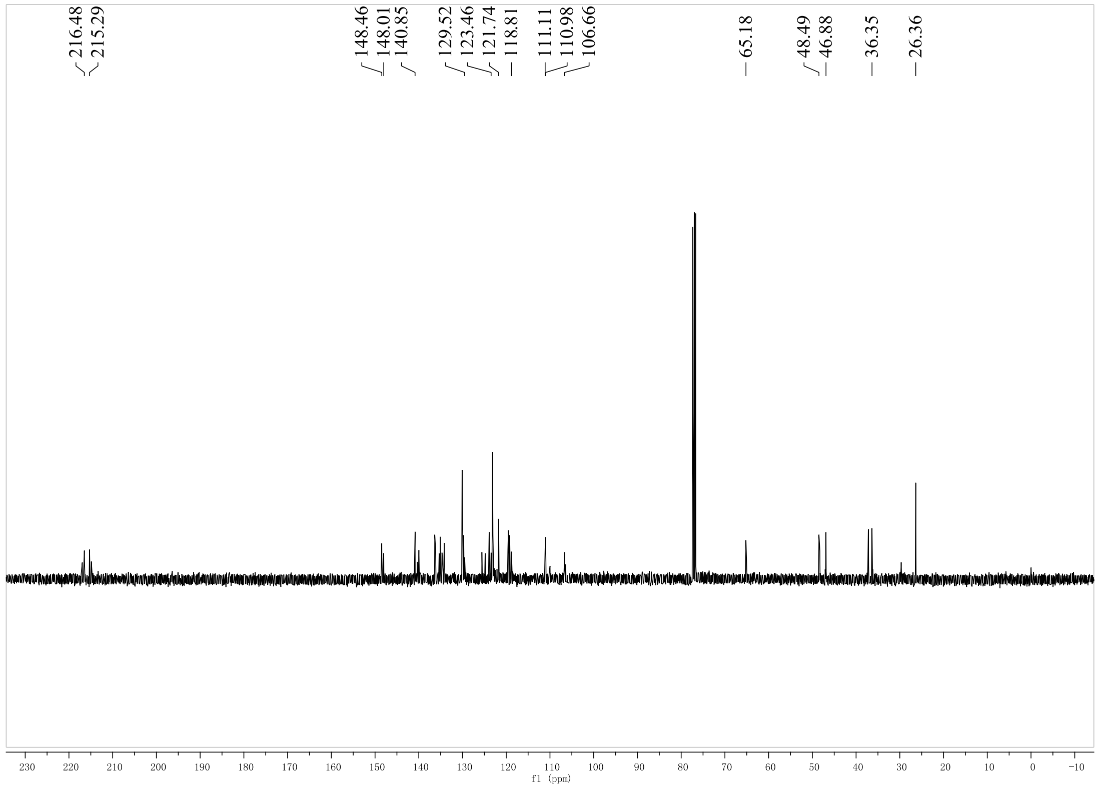
216.48
215.29
148.46
148.01
140.85
129.52
123.46
121.74
118.81
111.11
110.98
106.66
65.18
48.49
46.88
36.35
26.36
230 220 210 200 190 180 170 160 150 140 130 120 110 100 90 80 70 60 50 40 30 20 10 0 −10
f1 (ppm)

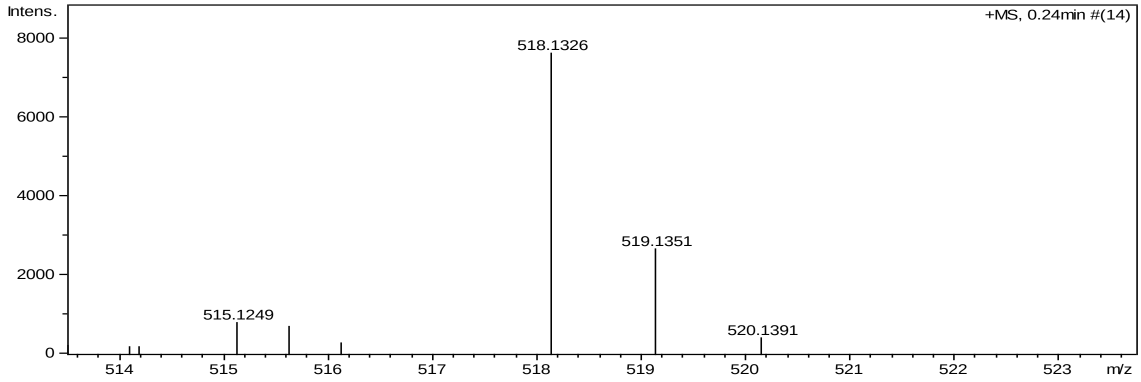
Intens.
+MS, 0.24min #(14)
8000
6000
4000
2000
0
518.1326
519.1351
515.1249
520.1391
514 515 516 517 518 519 520 521 522 523
m/z

2,4-Bis(3-chlorophenyl)-1,2,4,9-tetrahydrospiro[carbazole-3,1'-cyclopentane]-2',5'-dione (5e):

purple solid, 64%, m.p. 183-186 ºC; ^{1}H NMR (400 MHz, $CDCl_3$) δ: 8.04 (s, 1H, NH), 7.52 (d, *J* = 8.0 Hz, 1H, ArH), 7.27-7.26 (m, 2H, ArH), 7.24-7.22 (m, 2H, ArH), 7.18 (d, *J* = 7.2 Hz, 1H, ArH), 7.17-7.05 (m, 3H, ArH), 6.92-6.84 (m, 1H, ArH), 6.82-6.81 (m, 1H, ArH), 6.38 (d, *J* = 8.0 Hz, 1H, ArH), 4.82 (d, *J* = 21.6 Hz, 1H, CH), 3.87 (t, *J* = 14.8 Hz, 1H, CH), 3.63-3.61 (m, 1H, CH), 2.93 (dd, J_1 = 16.4 Hz, J_2 = 4.4 Hz, 1H, CH), 2.11-2.02 (m, 2H, CH_2), 1.56-1.46 (m, 1H, CH), 1.44-1.36 (m, 1H, CH); ^{13}C NMR (400 MHz, $CDCl_3$) δ: 217.4, 215.8, 140.9, 139.9, 136.0, 134.7, 134.2, 133.9, 130.1, 129.9, 129.5, 129.3, 128.6, 128.2, 128.1, 127.8, 127.0, 126.9, 125.9, 121.4, 121.4, 119.5, 119.4, 119.2, 110.6, 107.4, 107.2, 65.3, 48.4, 46.9, 37.3, 36.4, 26.4; IR (KBr) υ: 3211, 3167, 3049, 2971, 2855, 2149, 1854, 1633, 1617, 1559, 1473, 1351, 1249, 1139, 982, 966, 873, 755 cm^{-1}; MS (*m/z*): HRMS (ESI) Calcd. for $C_{28}H_{21}Cl_2{}^{35}NO_2$ ([M+Na]$^+$): 496.0842, found: 496.0844. $C_{28}H_{21}Cl_2{}^{37}NO_2$ ([M+Na]$^+$): 498.0812, found: 498.0816.

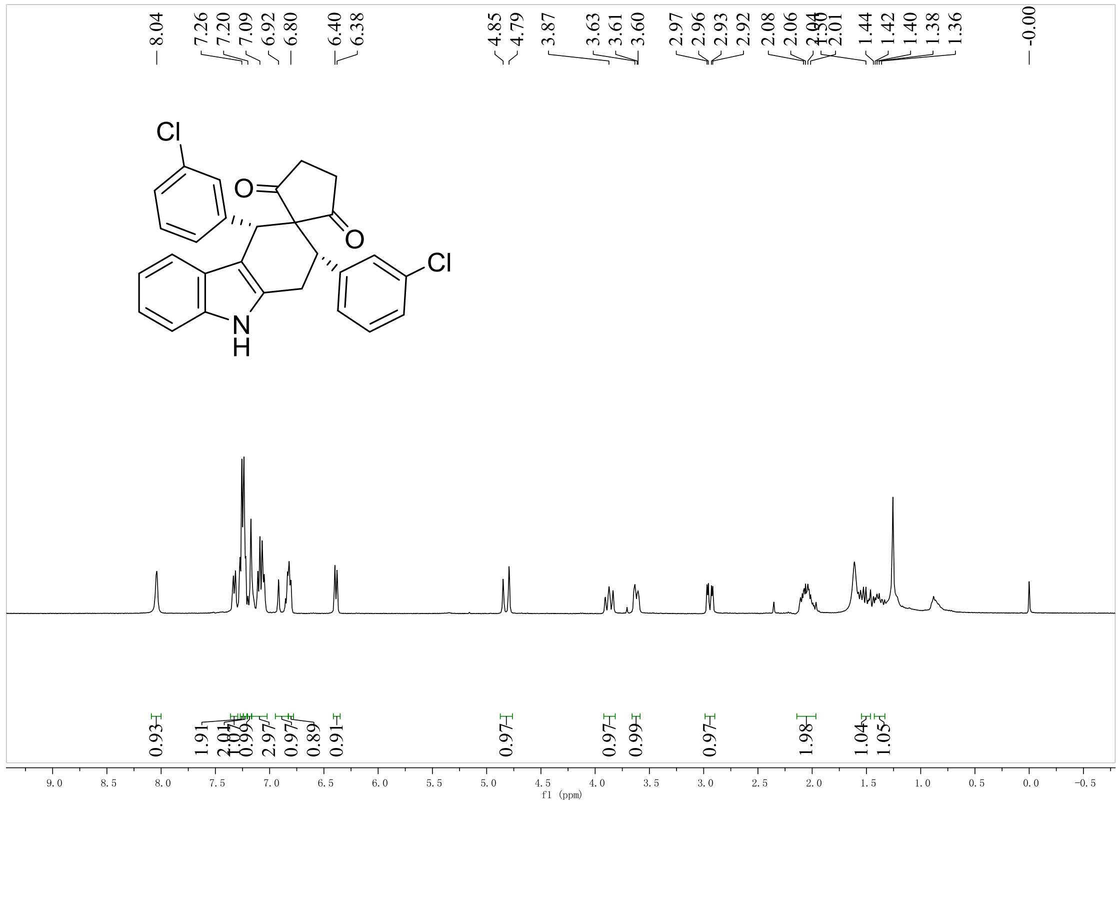

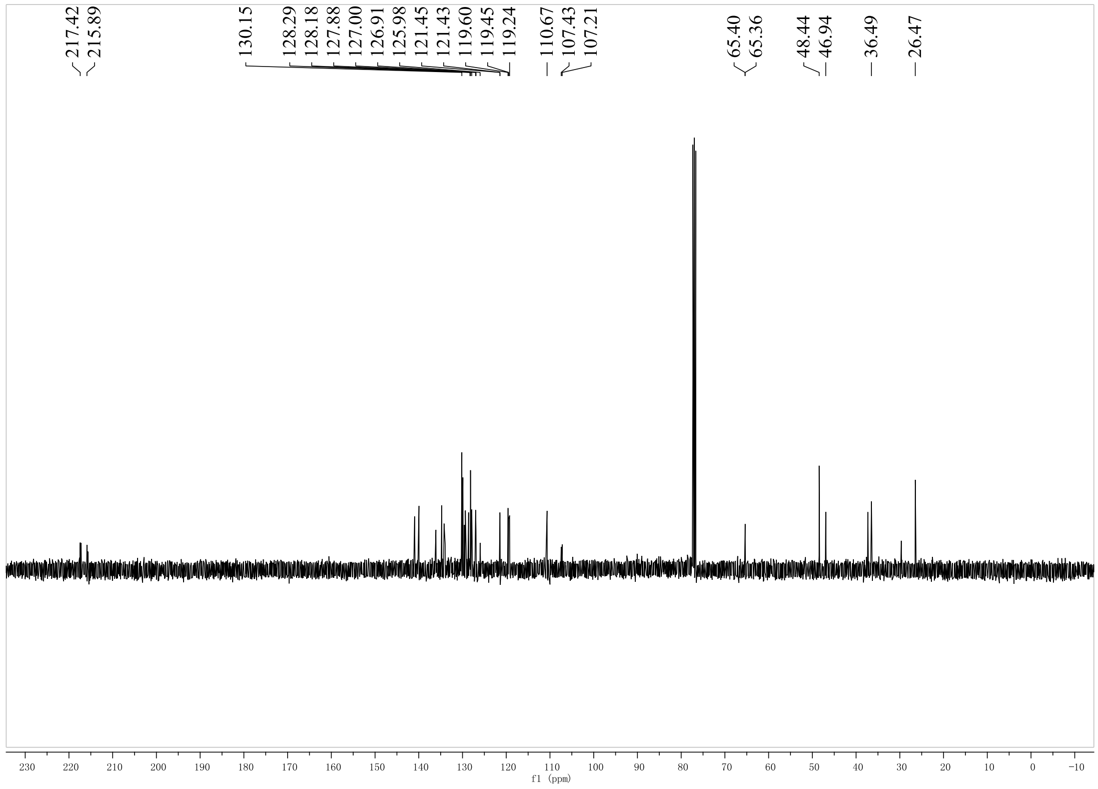
217.42
215.89
130.15
128.29
128.18
127.88
127.00
126.91
125.98
121.45
121.43
119.60
119.45
119.24
110.67
107.43
107.21
65.40
65.36
48.44
46.94
36.49
26.47
f1 (ppm)

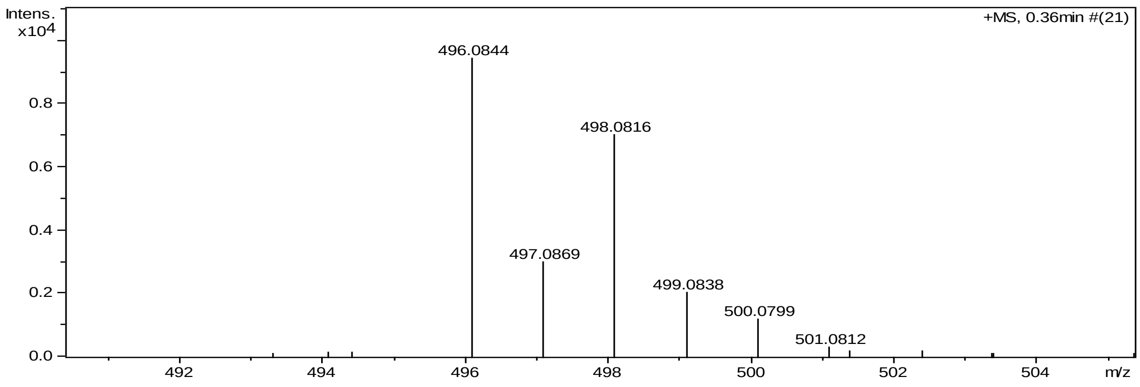
Intens.
x10^4
+MS, 0.36min #(21)
496.0844
498.0816
497.0869
499.0838
500.0799
501.0812
m/z

2,4-Bis(4-chlorophenyl)-1,2,4,9-tetrahydrospiro[carbazole-3,1'-cyclopentane]-2',5'-dione (5f):

purple solid, 73%, m.p. 180-183 ºC; ^{1}H NMR (400 MHz, $CDCl_3$) δ: 8.03 (s, 1H, NH), 7.31 (d, J = 8.4 Hz, 1H, ArH), 7.30-7.27 (m, 1H, ArH), 7.25-7.24 (m, 2H, ArH), 7.19 (d, J = 8.4 Hz, 1H, ArH), 7.14-7.07 (m, 4H, ArH), 6.83 (q, J = 7.6 Hz, 2H, ArH), 6.38 (d, J = 7.6 Hz, 1H, ArH), 4.83 (s, 1H, CH), 3.86 (t, J = 13.2 Hz, 1H, ArH), 3.63 (dd, J_1 = 12.0 Hz, J_2 = 4.8 Hz, 1H, CH), 2.92 (dd, J_1 = 16.4 Hz, J_2 = 4.8 Hz, 1H, CH), 2.06-1.98 (m, 2H, CH_2), 1.55-1.44 (m, 1H, CH), 1.42-1.35 (m, 1H, CH); ^{13}C NMR (400 MHz, $CDCl_3$) δ: 217.7, 216.1, 137.3, 136.3, 136.0, 134.2, 133.8, 133.5, 131.3, 130.7, 129.9, 129.0, 128.8, 128.4, 125.9, 121.3, 119.5, 119.2, 110.6, 107.4, 65.6, 48.1, 46.6, 37.3, 36.5, 26.4; IR (KBr) υ: 3217, 3167, 3034, 2980, 2848, 2167, 1850, 1632, 1600, 1535, 1470, 1362, 1278, 1143, 1101, 972, 900, 824, 768 cm^{-1}; MS (m/z): HRMS (ESI) Calcd. for $C_{28}H_{21}Cl_2{}^{35}NO_2$ ($[M+Na]^+$): 496.0842, found: 496.0842. $C_{28}H_{21}Cl_2{}^{37}NO_2$ ($[M+Na]^+$): 498.0812, found: 498.0818.

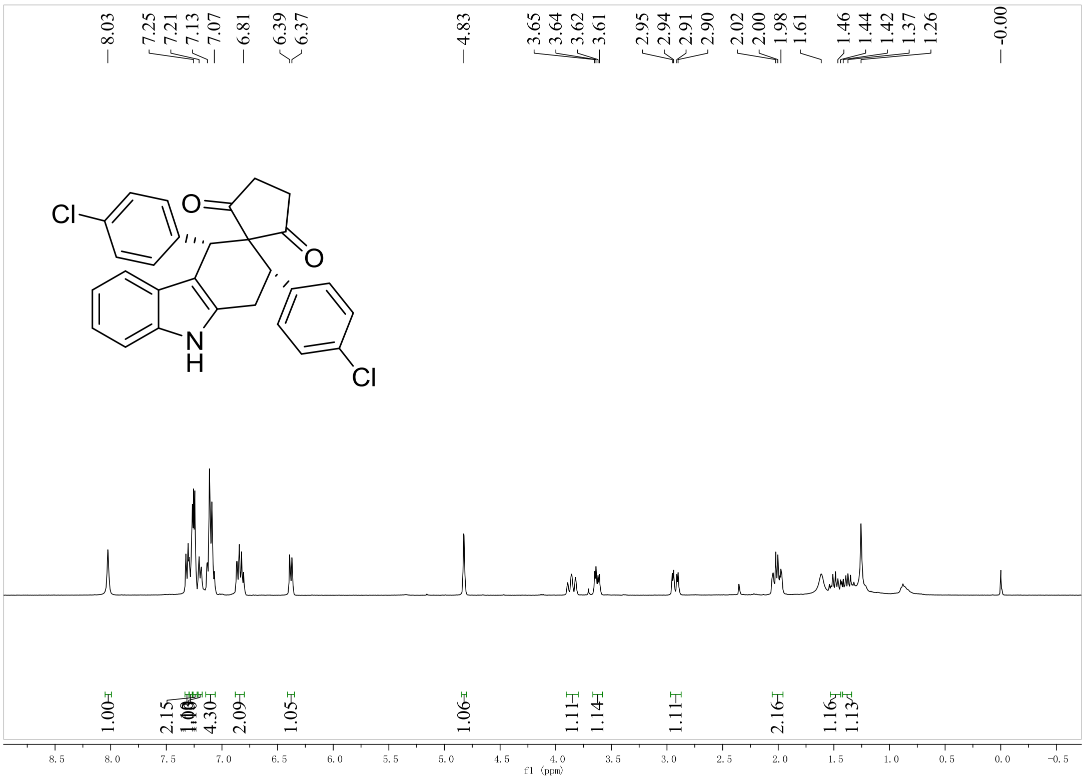

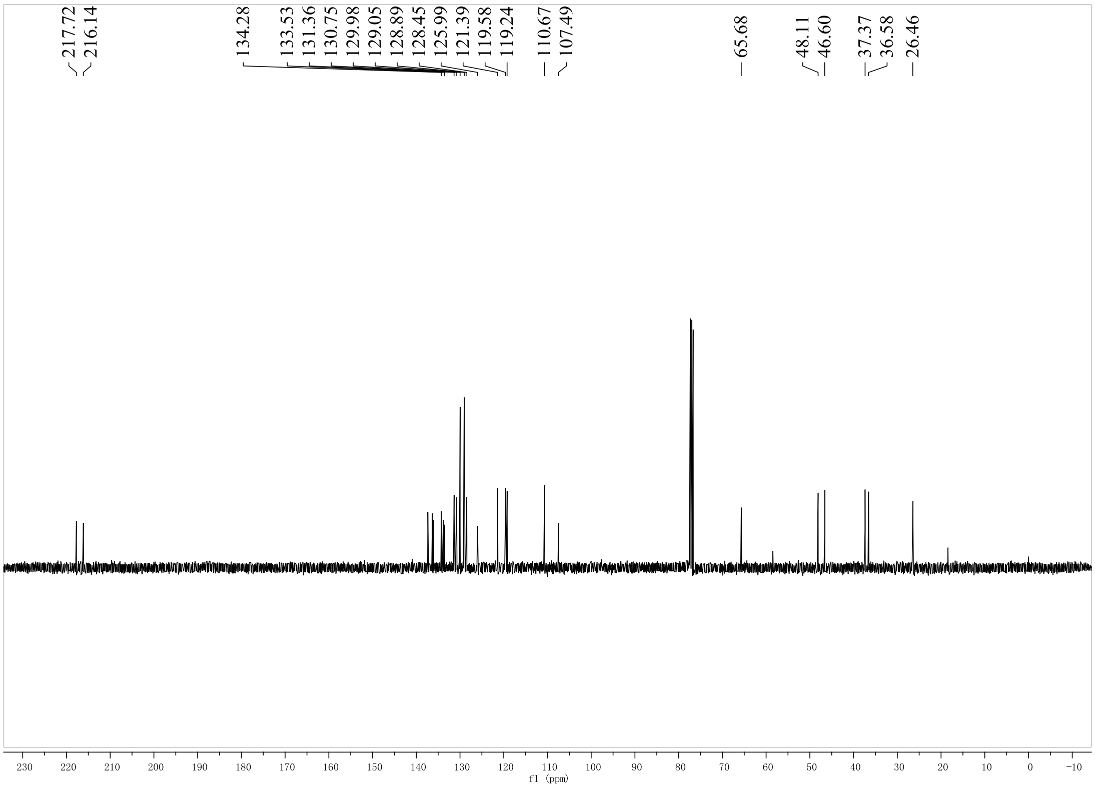
217.72
216.14
134.28
133.53
131.36
130.75
129.98
129.05
128.89
128.45
125.99
121.39
119.58
119.24
110.67
107.49
65.68
48.11
46.60
37.37
36.58
26.46
230 220 210 200 190 180 170 160 150 140 130 120 110 100 90 80 70 60 50 40 30 20 10 0 -10
f1 (ppm)

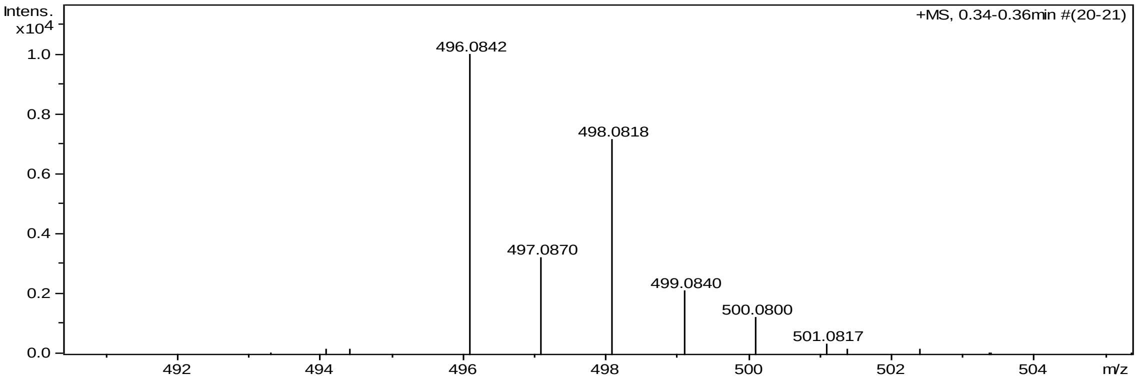
Intens.
x10^4
+MS, 0.34-0.36min #(20-21)
496.0842
498.0818
497.0870
499.0840
500.0800
501.0817
1.0
0.8
0.6
0.4
0.2
0.0
492
494
496
498
500
502
504
m/z

2,4-Bis(4-fluorophenyl)-1,2,4,9-tetrahydrospiro[carbazole-3,1'-cyclopentane]-2',5'-dione (5g):

purple solid, 65%, m.p. 179-182 ºC; ^{1}H NMR (400 MHz, $CDCl_3$) δ: 8.02 (s, 1H, NH), 7.31 (d, *J* = 8.0 Hz, 1H, ArH), 7.24-7.22 (m, 1H, ArH), 7.16-7.13 (m, 2H, ArH), 7.08 (t, *J* = 7.6 Hz, 1H, ArH), 7.01-6.95 (m, 3H, ArH), 6.92-6.88 (m, 1H, ArH), 6.87-6.83 (m, 1H, ArH), 6.80 (d, *J* = 8.0 Hz, 1H, ArH), 6.37 (d, *J* = 8.0 Hz, 1H, ArH), 4.84 (s, 1H, CH), 3.87-3.83 (m, 1H, CH), 3.65 (dd, J_1 = 12.0 Hz, J_2 = 4.8 Hz, 1H, CH), 2.93 (dd, J_1 = 16.4 Hz, J_2 = 4.8 Hz, 1H, CH), 2.03-1.96 (m, 2H, CH_2), 1.48-1.33 (m, 2H, CH_2); ^{13}C NMR (400 MHz, $CDCl_3$) δ: 217.9, 216.3, 163.3, 160.8, 136.1, 134.2, 131.6, 131.6, 130.9, 130.9, 130.2, 130.2, 126.0, 121.3, 119.6, 119.1, 115.8, 115.6, 110.6, 107.8, 65.8, 47.9, 46.4, 37.3, 36.5, 26.6; IR (KBr) υ: 3217, 3168, 3049, 2973, 2850, 2146, 1853, 1617, 1600, 1554, 1423, 1331, 1245, 1178, 1116, 973, 888, 807, 759 cm^{-1}; MS (*m/z*): HRMS (ESI) Calcd. for $C_{28}H_{21}F_2NO_2$ ($[M+Na]^+$): 464.1433, found: 464.1436.

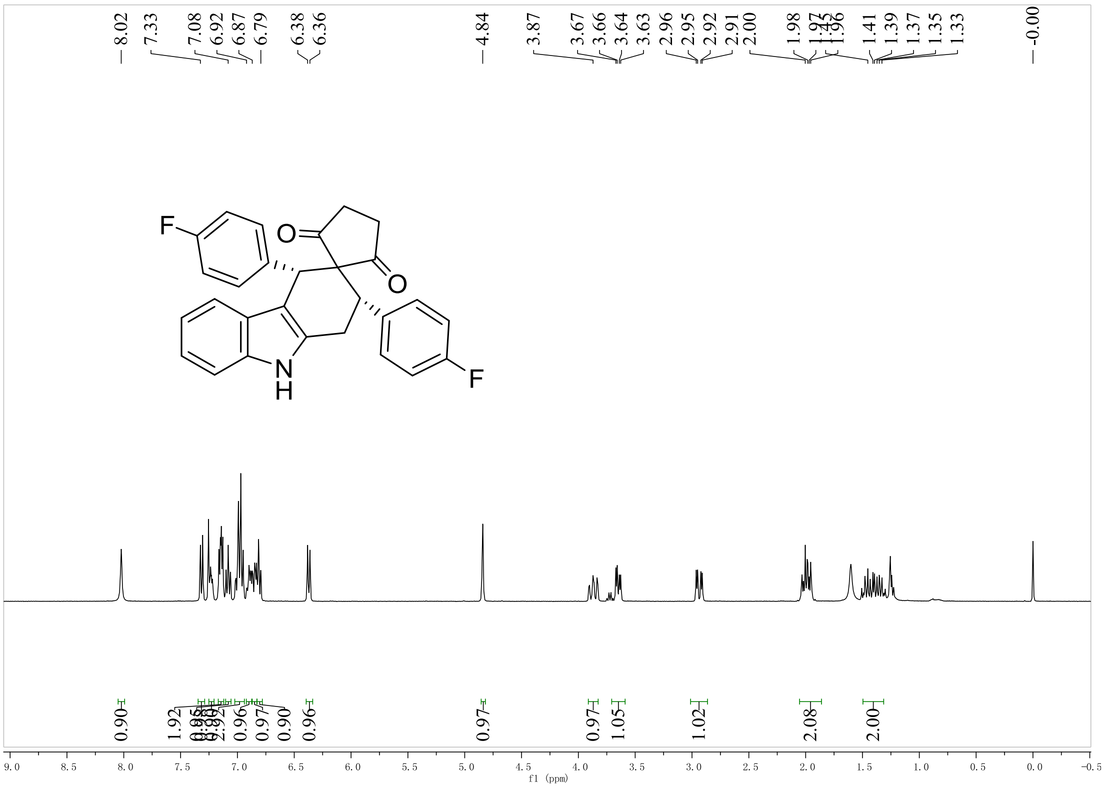

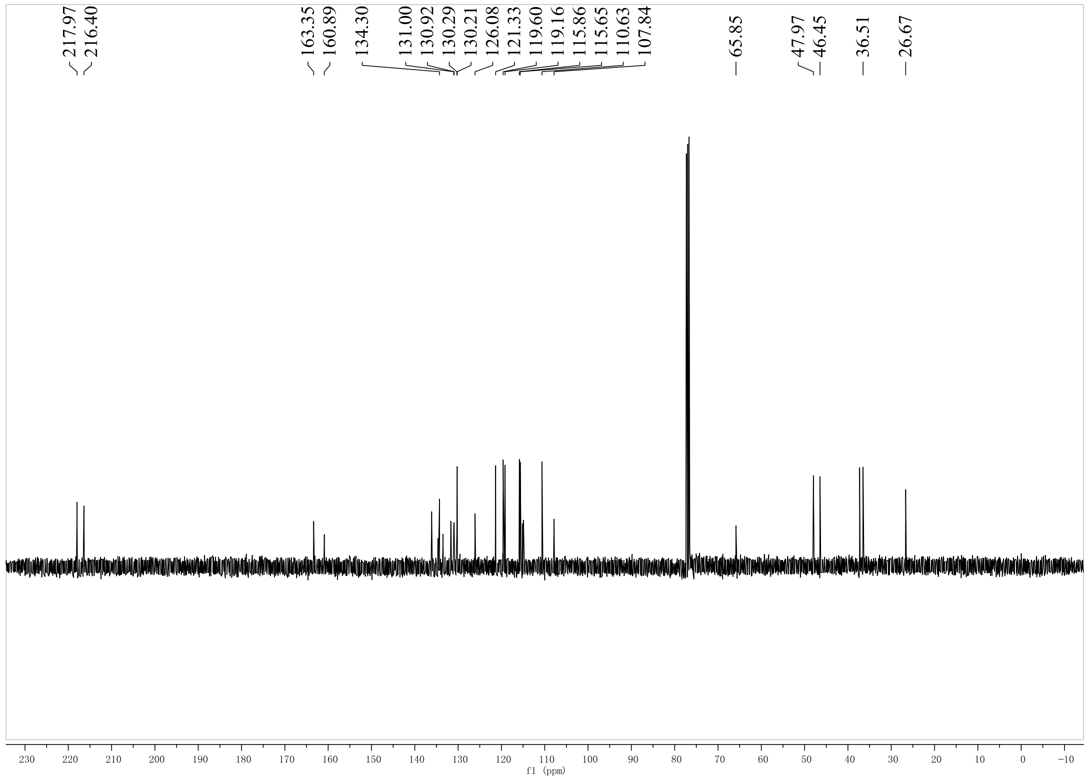
217.97
216.40
163.35
160.89
134.30
131.00
130.92
130.29
130.21
126.08
121.33
119.60
119.16
115.86
115.65
110.63
107.84
65.85
47.97
46.45
36.51
26.67
f1 (ppm)

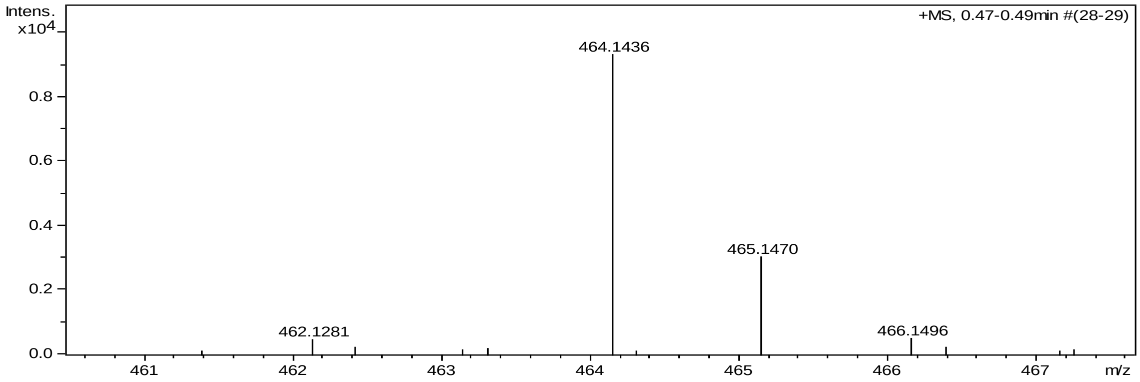
Intens.
x10^4
+MS, 0.47-0.49min #(28-29)
464.1436
465.1470
462.1281
466.1496
m/z

2,4-Diphenyl-1,2,4,9-tetrahydrospiro[carbazole-3,1'-cyclohexane]-2',6'-dione (5h):

purple solid, 76%, m.p. 182-185 ºC; ^{1}H NMR (400 MHz, $CDCl_3$) δ: 7.99 (s, 1H, NH), 7.33-7.28 (m, 4H, ArH), 7.26-7.23 (m, 3H, ArH), 7.16-7.14 (m, 3H, ArH), 7.04 (t, *J* = 7.6 Hz, 1H, ArH), 6.89 (d, *J* = 7.6 Hz, 1H, ArH), 6.33 (d, *J* = 8.0 Hz, 1H, ArH), 5.15 (s, 1H, CH), 3.83 (t, *J* = 12.0 Hz, 2H, CH_2), 2.97 (t, *J* = 12.4 Hz, 1H, CH), 2.04-1.93 (m, 2H, CH_2), 1.66-1.59 (m, 1H, CH), 1.50-1.43 (m, 1H, CH), 0.80-0.74 (m, 2H, CH_2); ^{13}C NMR (400 MHz, $CDCl_3$) δ: 213.9, 210.9, 139.8, 138.4, 136.0, 134.8, 130.2, 129.9, 129.1, 128.7, 128.5, 128.2, 127.6, 127.4, 126.5, 120.7, 119.7, 118.7, 110.4, 108.7, 71.7, 50.2, 48.8, 42.9, 41.5, 28.2, 14.7; IR (KBr) υ: 3407, 3271, 3063, 2962, 1871, 1763, 1645, 1600, 1538, 1462, 1345, 1321, 1266, 1148, 961, 844 cm^{-1}; MS (*m*/*z*): HRMS (ESI) Calcd. for $C_{29}H_{25}NO_2$ ($[M+Na]^+$): 442.1778, found: 442.1783.

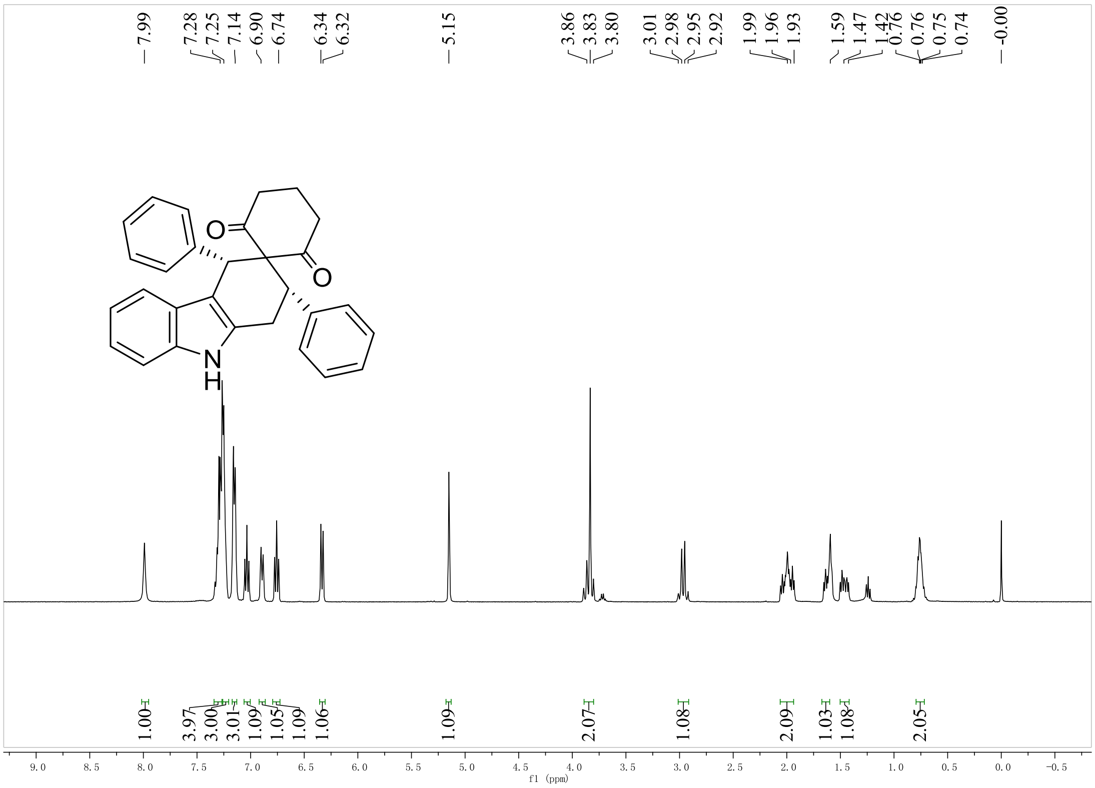

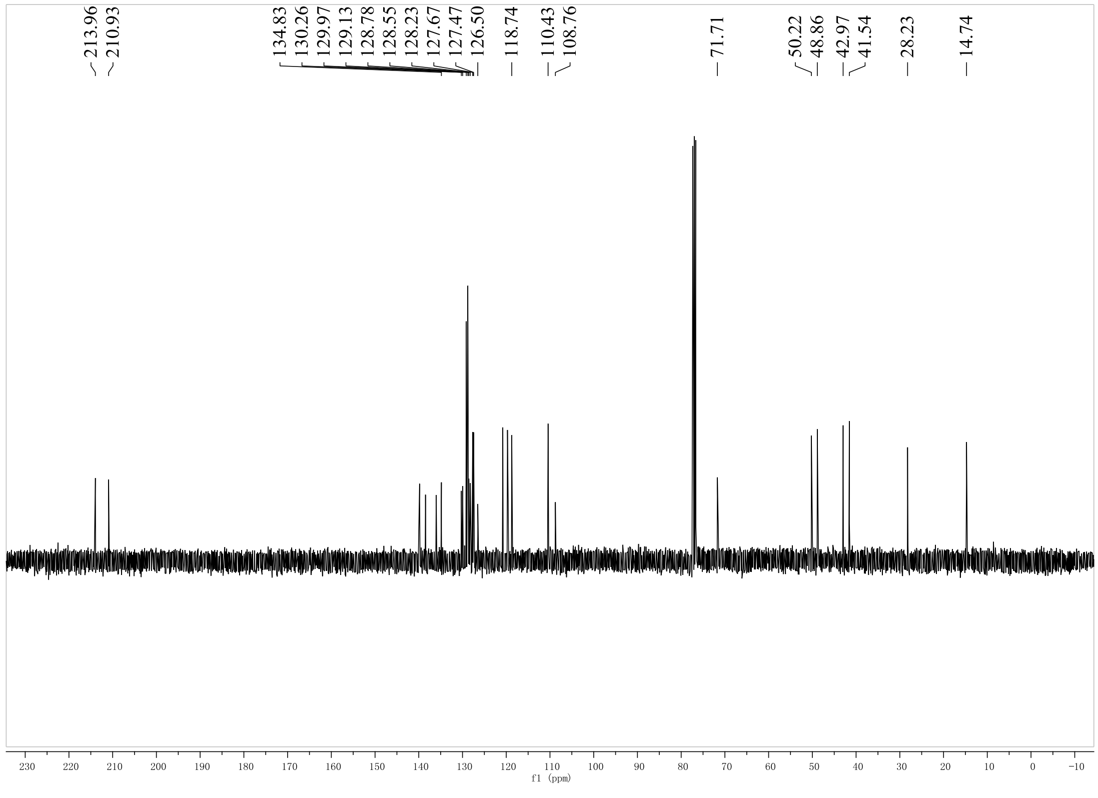
213.96
210.93
134.83
130.26
129.97
129.13
128.78
128.55
128.23
127.67
127.47
126.50
118.74
110.43
108.76
71.71
50.22
48.86
42.97
41.54
28.23
14.74
f1 (ppm)

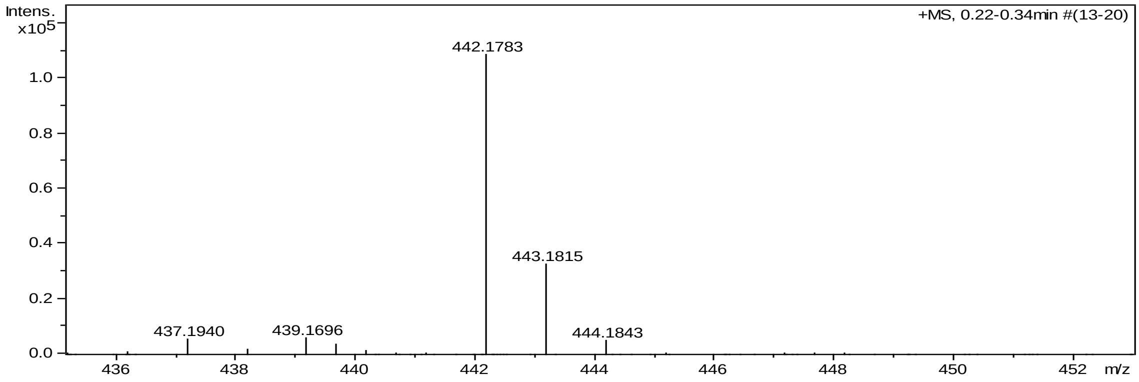
Intens.
x10^5
+MS, 0.22-0.34min #(13-20)
442.1783
443.1815
437.1940
439.1696
444.1843
m/z

2,4-Di-p-tolyl-1,2,4,9-tetrahydrospiro[carbazole-3,1'-cyclohexane]-2',6'-dione (5i):

purple solid, 75%, m.p. 182-185 °C; ^{1}H NMR (400 MHz, $CDCl_3$) δ: 7.96 (s, 1H, NH), 7.28 (d, *J* = 8.0 Hz, 1H, ArH), 7.14-7.11 (m, 2H, ArH), 7.09-7.07 (m, 2H, ArH), 7.04-7.01 (m, 3H, ArH), 6.95 (d, *J* = 8.0 Hz, 1H, ArH), 6.78-6.75 (m, 2H, ArH), 6.37 (d, *J* = 8.0 Hz, 1H, ArH), 5.10 (s, 1H, CH), 3.83-3.73 (m, 2H, CH_2), 2.92 (q, *J* = 12.8 Hz, 1H, CH), 2.31 (s, 3H, CH_3), 2.30 (s, 3H, CH_3), 2.07-1.94 (m, 2H, CH_2), 1.69-1.62 (m, 1H, CH), 1.53-1.46 (m, 1H, CH), 0.83-0.75 (m, 2H, CH_2); ^{13}C NMR (400 MHz, $CDCl_3$) δ: 214.2, 211.1, 137.2, 136.9, 136.8, 135.9, 135.3, 134.8, 130.1, 130.0, 129.8, 129.4, 129.4, 129.3, 128.9, 128.7, 126.5, 120.6, 119.7, 118.6, 110.4, 108.9, 71.8, 49.8, 48.4, 43.0, 41.5, 28.3, 21.1, 21.0, 14.8; IR (KBr) υ: 3441, 3245, 3001，2946, 1867, 1750, 1638, 1617, 1549, 1461, 1355, 1321, 1249, 1157, 969，864, 771 cm^{-1}; MS (*m*/*z*): HRMS (ESI) Calcd. for $C_{31}H_{29}NO_2$ ($[M+Na]^+$): 470.2091, found: 470.2094.

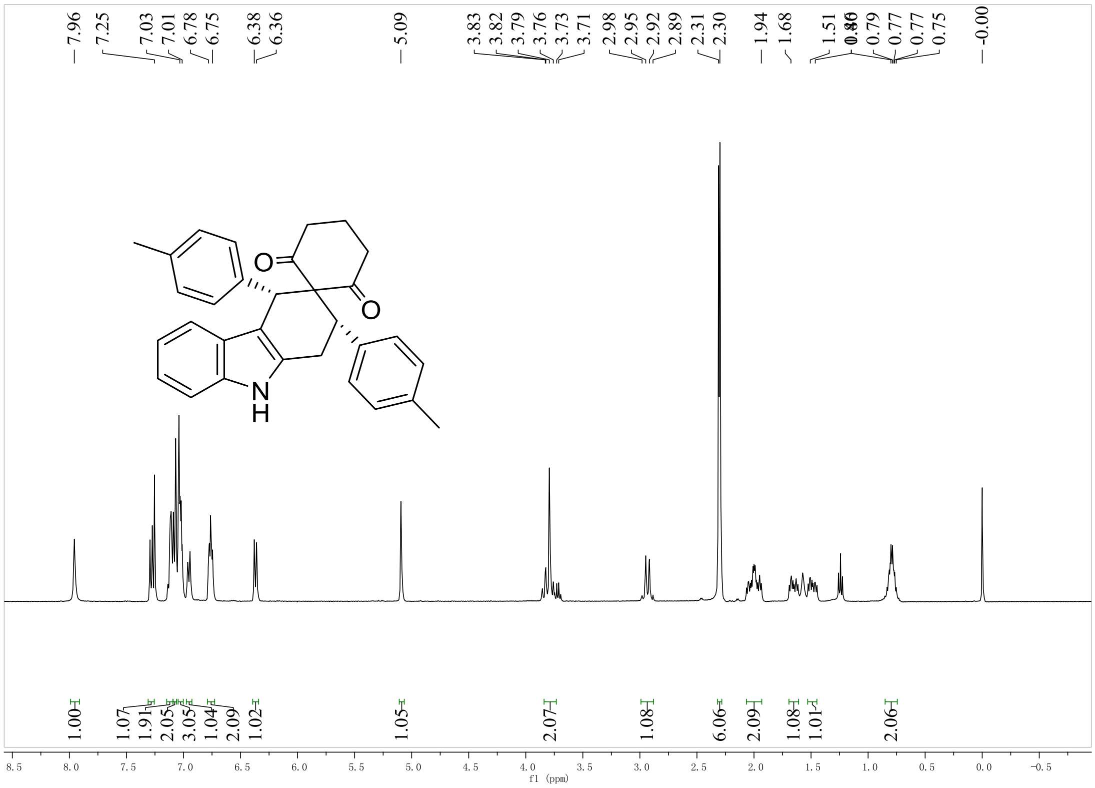

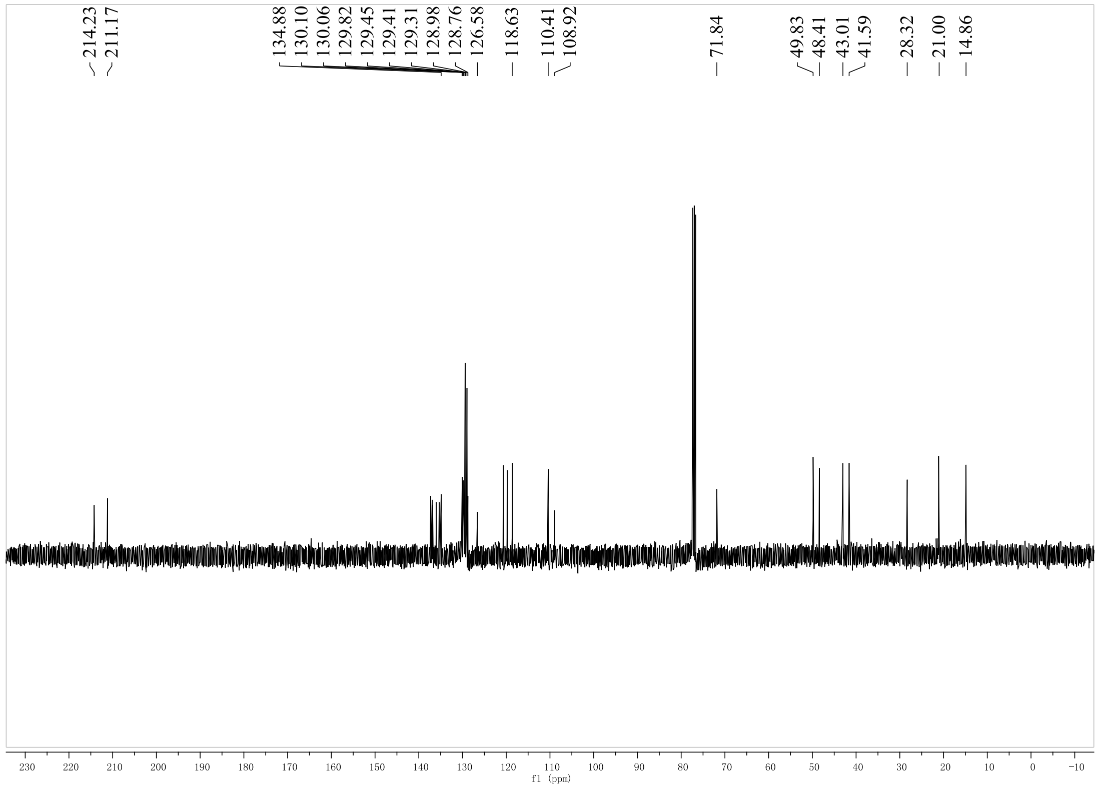
214.23
211.17
134.88
130.10
130.06
129.82
129.45
129.41
129.31
128.98
128.76
126.58
118.63
110.41
108.92
71.84
49.83
48.41
43.01
41.59
28.32
21.00
14.86
f1 (ppm)

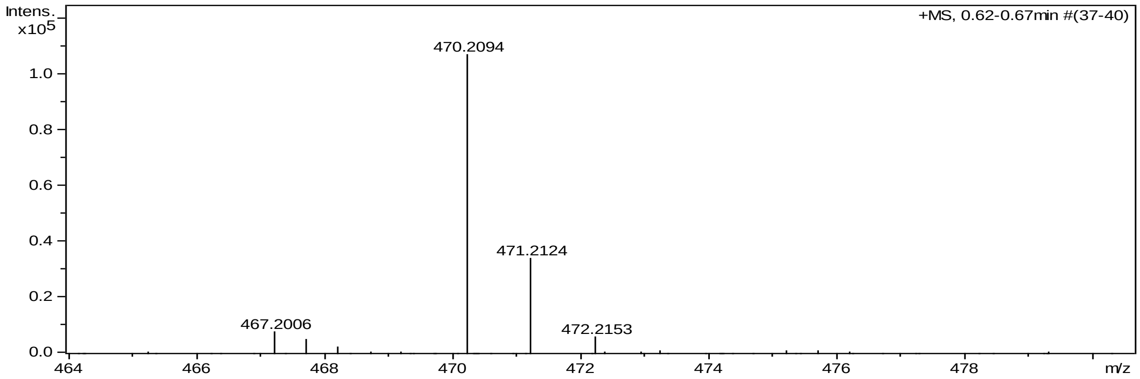
Intens.
x10^5
+MS, 0.62-0.67min #(37-40)
470.2094
471.2124
467.2006
472.2153
m/z

2,4-Di-*o*-tolyl-1,2,4,9-tetrahydrospiro[carbazole-3,1'-cyclohexane]-2',6'-dione (5j):

purple solid, 78%, m.p. 188-191 ºC; ^{1}H NMR (400 MHz, $CDCl_3$) δ: 7.99 (s, 1H, NH), 7.24-7.20 (m, 3H, ArH), 7.16-7.12 (m, 2H, ArH), 7.12-7.09 (m, 2H, ArH), 6.98 (dd, J_1 = 18.4 Hz, J_2 = 8.0 Hz, 2H, ArH), 6.89 (d, J = 8.0 Hz, 1H, ArH), 6.70 (t, J = 8.0 Hz, 1H, ArH), 6.09 (d, J = 7.6 Hz, 1H, ArH), 5.38 (s, 1H, CH), 4.25 (q, J = 5.6 Hz, 1H, CH), 3.74 (t, J = 15.2 Hz, 1H, CH), 2.90 (dd, J_1 = 16.0 Hz, J_2 = 5.2 Hz, 1H, CH), 2.40 (s, 3H, CH_3), 2.35 (s, 3H, CH_3), 2.30-2.29 (m, 1H, CH), 2.02-1.94 (m, 1H, CH), 1.91-1.84 (m, 1H, CH), 1.73-1.66 (m, 1H, CH), 1.24-1.19 (m, 1H, CH), 0.89-0.84 (m, 1H, CH); ^{13}C NMR (400 MHz, $CDCl_3$) δ: 212.8, 212.5, 139.4, 137.0, 136.7, 136.4, 135.9, 135.3, 131.2, 131.1, 130.8, 127.3, 127.1, 127.0, 126.6, 126.2, 126.1, 120.8, 119.2, 118.8, 110.4, 110.0, 69.3, 46.7, 43.9, 43.6, 41.1, 29.9, 20.0, 19.8, 15.3; IR (KBr) υ: 3403, 3169, 3064, 2971, 1846, 1732, 1654, 1645, 1581, 1472, 1331, 1300, 1280, 1157, 969, 867, 782 cm^{-1}; MS (m/z): HRMS (ESI) Calcd. for $C_{31}H_{29}NO_2$ ($[M+Na]^+$): 470.2091, found: 470.2096.

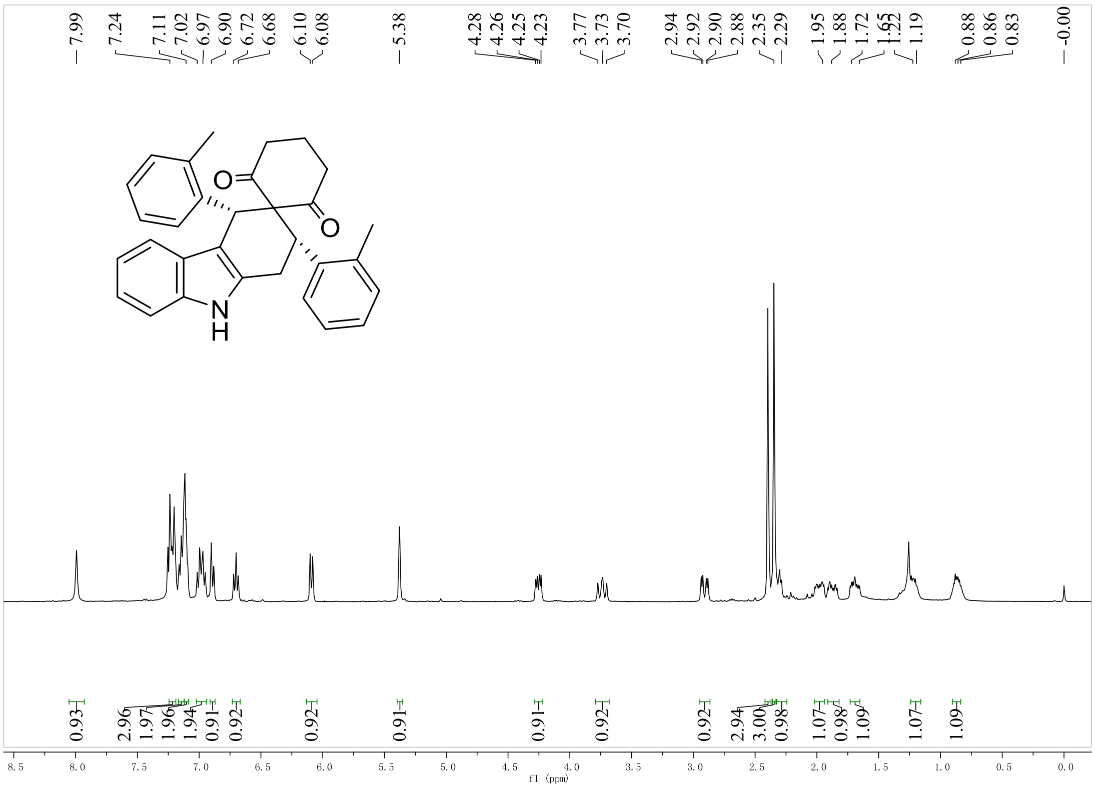

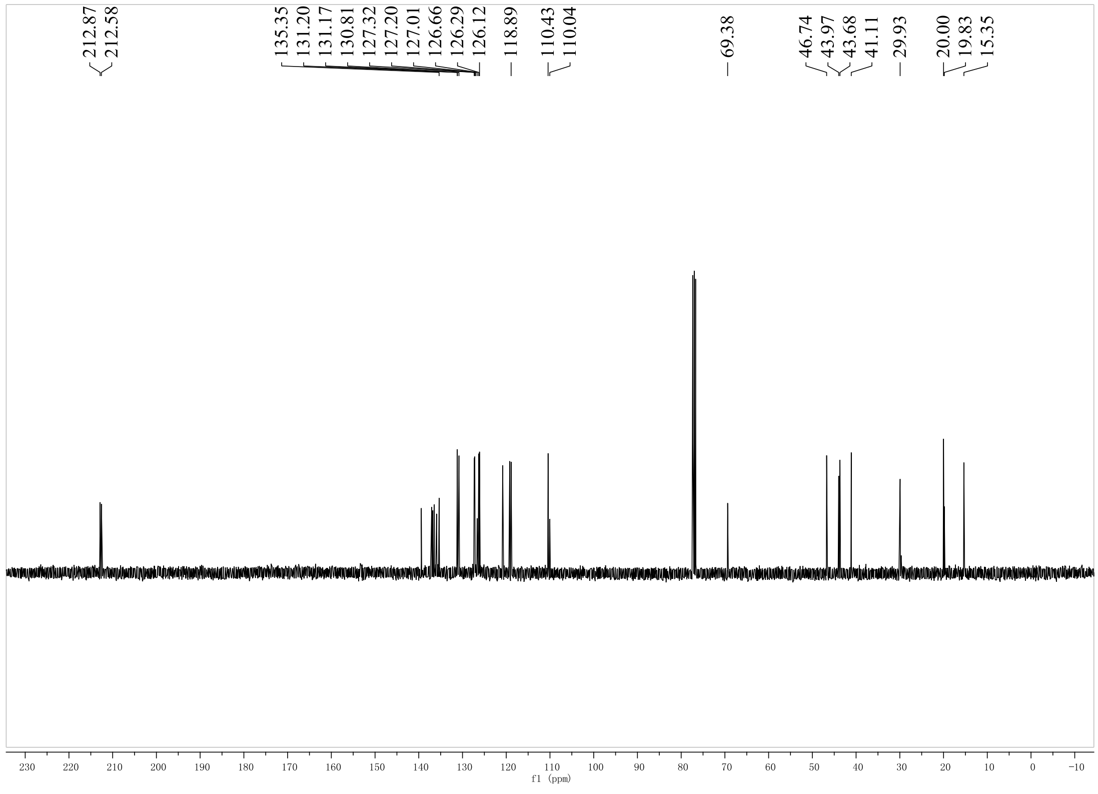
212.87
212.58
135.35
131.20
131.17
130.81
127.32
127.20
127.01
126.66
126.29
126.12
118.89
110.43
110.04
69.38
46.74
43.97
43.68
41.11
29.93
20.00
19.83
15.35
f1 (ppm)

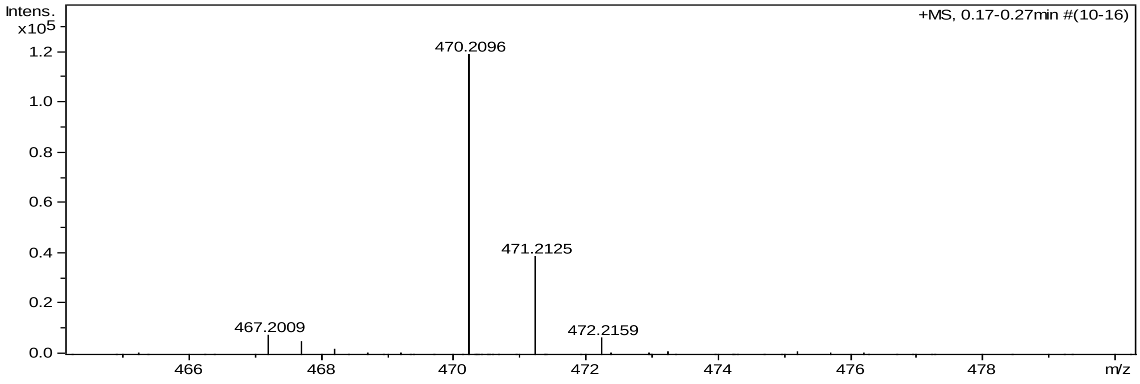
Intens.
x10^5
+MS, 0.17-0.27min #(10-16)
470.2096
471.2125
467.2009
472.2159
m/z

2,4-Bis(3-chlorophenyl)-1,2,4,9-tetrahydrospiro[carbazole-3,1'-cyclohexane]-2',6'-dione (5k): purple solid, 71%, m.p. 187-190 ºC; ^{1}H NMR (400 MHz, $CDCl_3$) δ: 7.99 (s, 1H, NH), 7.32-7.26 (m, 3H, ArH), 7.25-7.19 (m, 2H, ArH), 7.15-7.04 (m, 4H, ArH), 6.89-6.79 (m, 2H, ArH), 6.35-6.34 (m, 1H, ArH), 5.10 (d, J = 16.0 Hz, 1H, CH), 3.79-3.77 (m, 2H, CH_2), 2.97-2.92 (m, 1H, CH), 2.13-2.05 (m, 2H, CH_2), 1.76-1.64 (m, 1H, CH), 1.53-1.48 (m, 1H, CH), 0.88-0.87 (m, 2H, CH_2); ^{13}C NMR (400 MHz, $CDCl_3$) δ: 217.4, 215.8, 140.9, 139.9, 136.0, 134.7, 134.2, 133.9, 130.1, 129.9, 129.5, 129.3, 128.6, 128.6, 128.2, 128.1, 128.1, 127.8, 127.7, 127.0, 126.9, 125.9, 121.4, 121.4, 119.5, 119.4, 119.4, 119.2, 110.7, 110.6, 107.4, 107.2, 65.3, 48.4, 46.9, 37.3, 36.4, 29.6, 26.4; IR (KBr) υ: 3431, 3244, 3061, 2946, 1857, 1768, 1645, 1600, 1548, 1431, 1360, 1331, 1266, 1146, 862, 792 cm^{-1}; MS (m/z): HRMS (ESI) Calcd. for $C_{29}H_{23}Cl_2{}^{35}NO_2$ ($[M+Na]^+$): 510.0998, found: 510.0998. $C_{29}H_{23}Cl_2{}^{37}NO_2$ ($[M+Na]^+$): 512.0969, found: 512.0976.

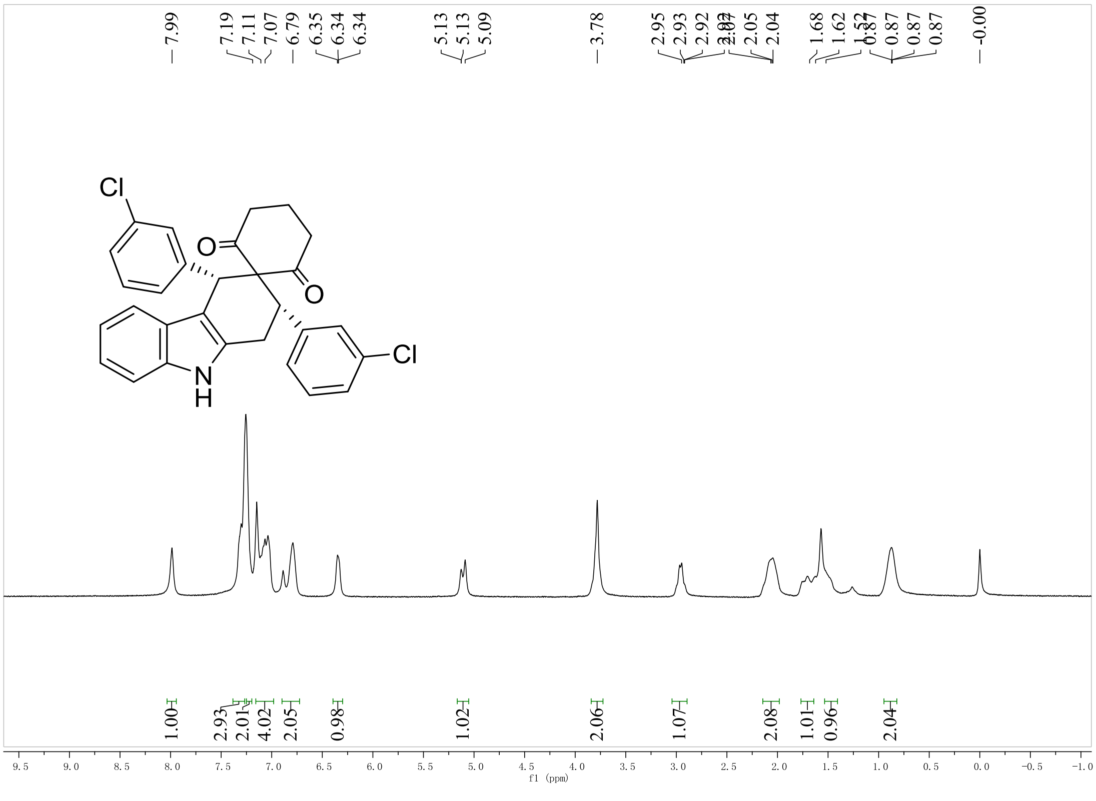

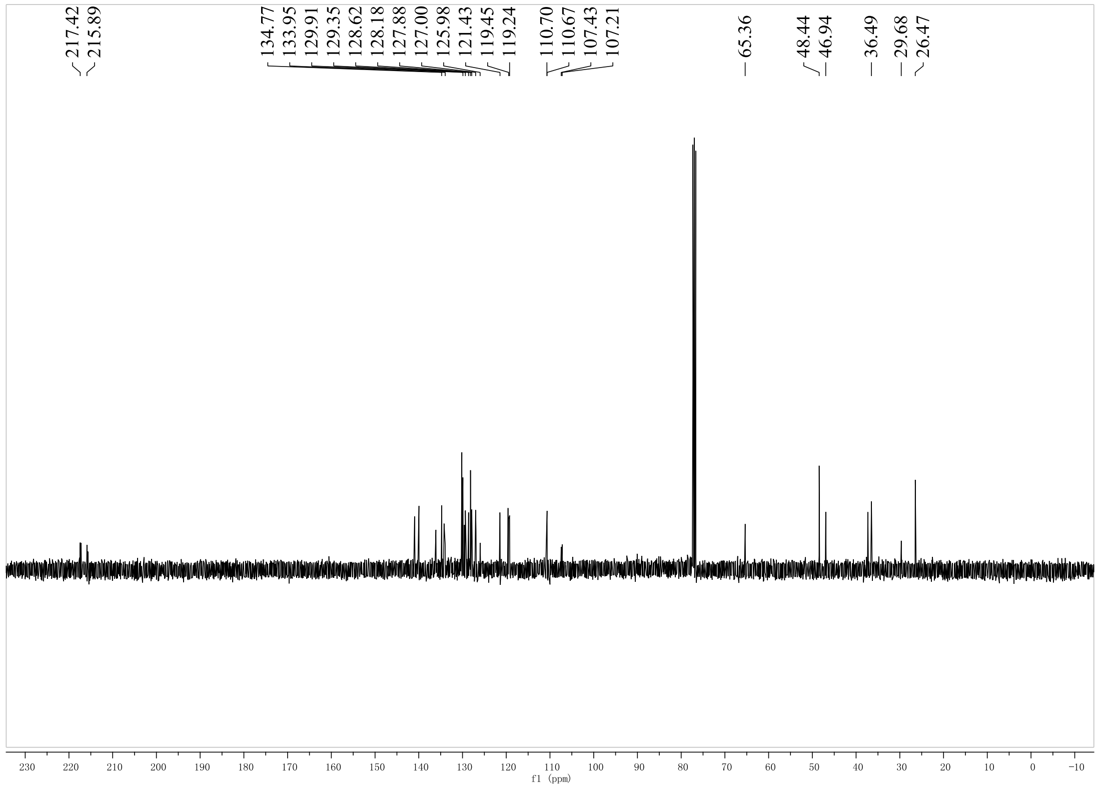
217.42
215.89
134.77
133.95
129.91
129.35
128.62
128.18
127.88
127.00
125.98
121.43
119.45
119.24
110.70
110.67
107.43
107.21
65.36
48.44
46.94
36.49
29.68
26.47
f1 (ppm)

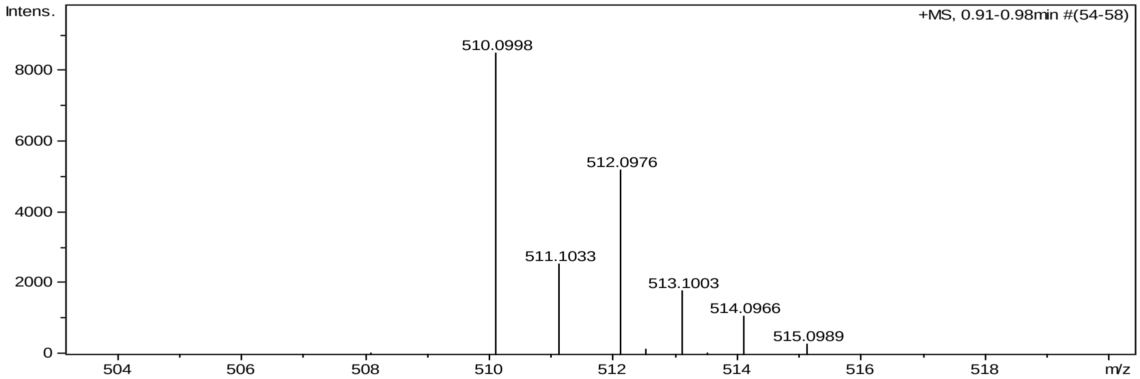
Intens.
+MS, 0.91-0.98min #(54-58)
510.0998
512.0976
511.1033
513.1003
514.0966
515.0989
m/z

3,3'-(*p*-Tolylmethylene)bis(2-methyl-1H-indole) (6a):

purple solid, 75%, m.p. 176-178 ºC; ^{1}H NMR (400 MHz, $CDCl_3$) δ: 7.56 (s, 2H, NH), 7.17-7.14 (m, 4H, ArH), 7.03-6.99 (m, 6H, ArH), 6.85-6.84 (m, 2H, ArH), 5.93 (s, 1H, CH), 2.31 (s, 3H, CH_3), 1.98 (s, 3H, CH_3), 1.97 (s, 3H, CH_3); ^{13}C NMR (400 MHz, $CDCl_3$) δ: 140.5, 135.3, 135.0, 131.8, 128.9, 128.9, 128.8, 120.5, 119.4, 118.9, 113.5, 110.0, 38.8, 21.1, 12.3; IR (KBr) υ: 3400, 2918, 1547, 1526, 1348, 1287, 1260, 750 cm^{-1}; MS (*m*/*z*): HRMS (ESI) Calcd. for $C_{26}H_{24}N_2$ ($[M+Na]^+$): 387.1832, found: 387.1833.

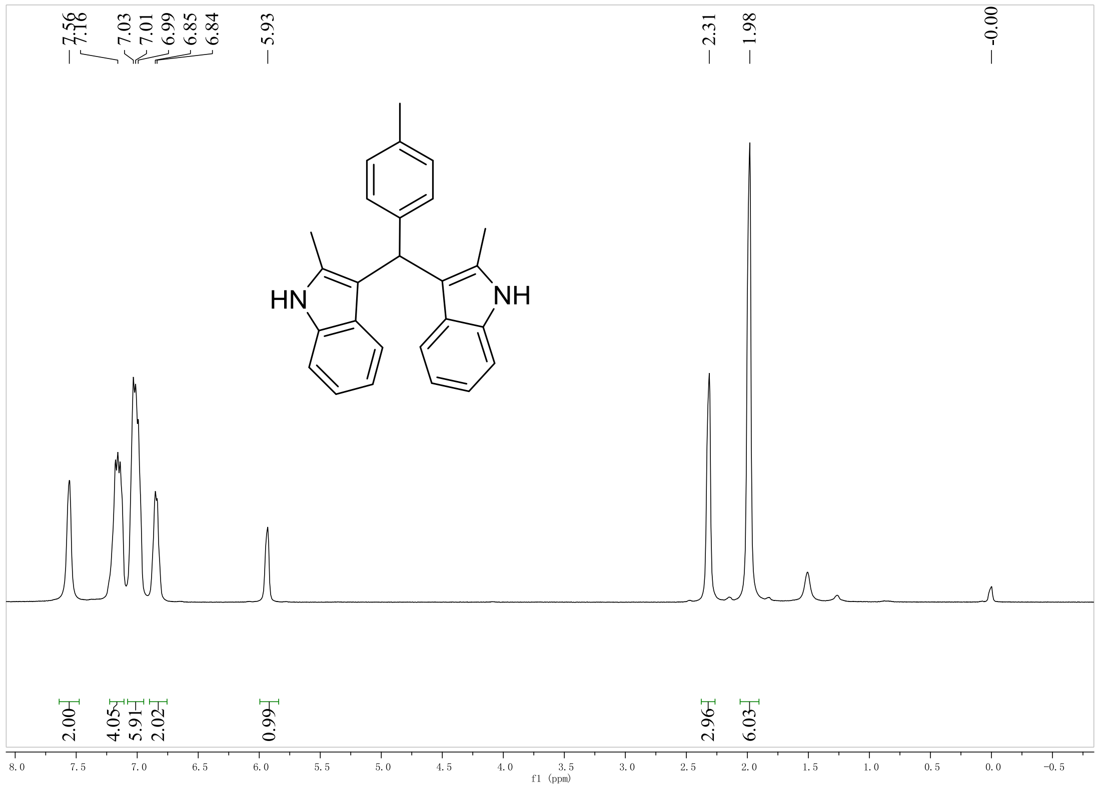

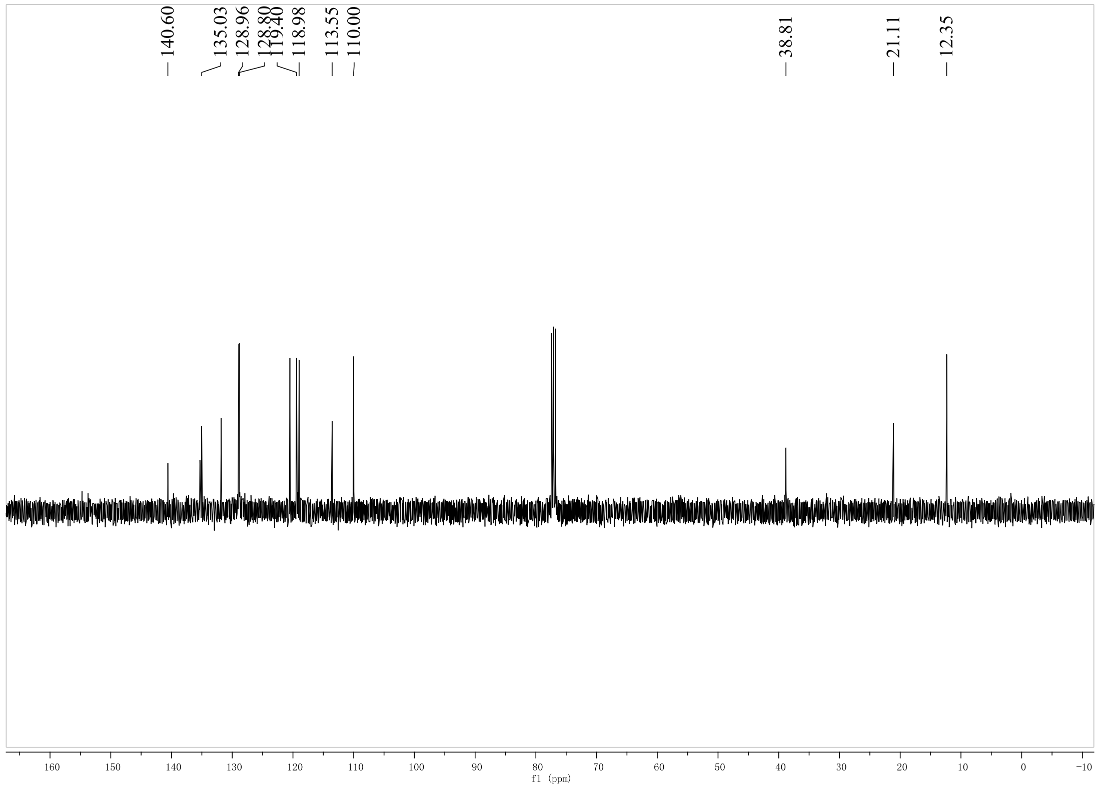
140.60
135.03
128.96
128.80
119.40
118.98
113.55
110.00
38.81
21.11
12.35
f1 (ppm)

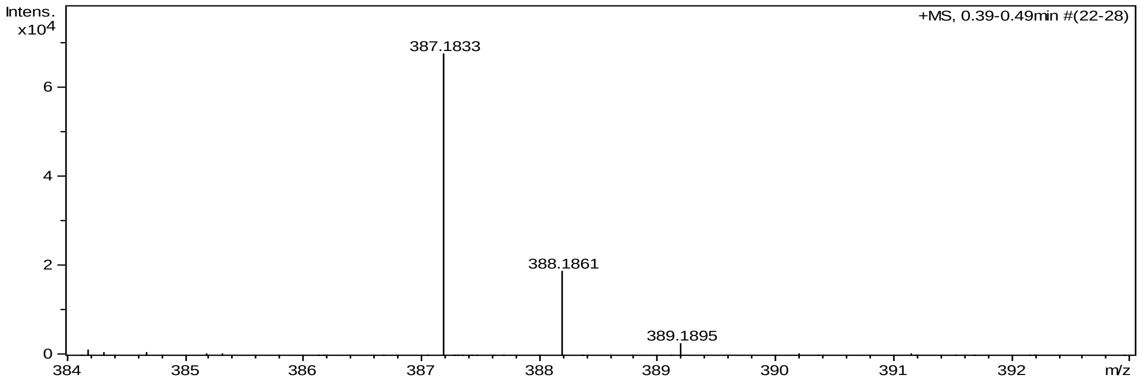
Intens.
x10^4
+MS, 0.39-0.49min #(22-28)
387.1833
388.1861
389.1895
m/z

3,3'-((2-Methoxyphenyl)methylene)bis(2-methyl-1H-indole) (6b):

purple solid, 72%, m.p. 185-187 °C; ^{1}H NMR (400 MHz, $CDCl_3$) δ: 7.66 (s, 2H, NH), 7.20 (d, *J* = 7.6 Hz, 3H, ArH), 7.16 (d, *J* = 7.6 Hz, 1H, ArH), 7.00 (t, *J* = 7.6 Hz, 2H, ArH), 6.94 (d, *J* = 8.0 Hz, 2H, ArH), 6.88 (d, *J* = 8.0 Hz, 1H, ArH), 6.81 (d, *J* = 7.6 Hz, 3H, ArH), 6.22 (s, 1H, CH), 3.68 (s, 3H, OCH_3), 2.00 (s, 3H, CH_3), 1.99 (s, 3H, CH_3); ^{13}C NMR (400 MHz, $CDCl_3$) δ: 157.8, 135.8, 135.0, 131.7, 129.9, 128.9, 120.5, 119.3, 119.0, 113.6, 113.4, 109.9, 55.2, 55.2, 38.3, 12.3; IR (KBr) υ: 3412, 3056, 2921, 2850, 1616, 1455, 1339, 1288, 1250, 744 cm^{-1}; MS (*m*/*z*): HRMS (ESI) Calcd. for $C_{26}H_{24}N_2O$ ([M+Na]$^+$): 403.1781, found: 403.1788.

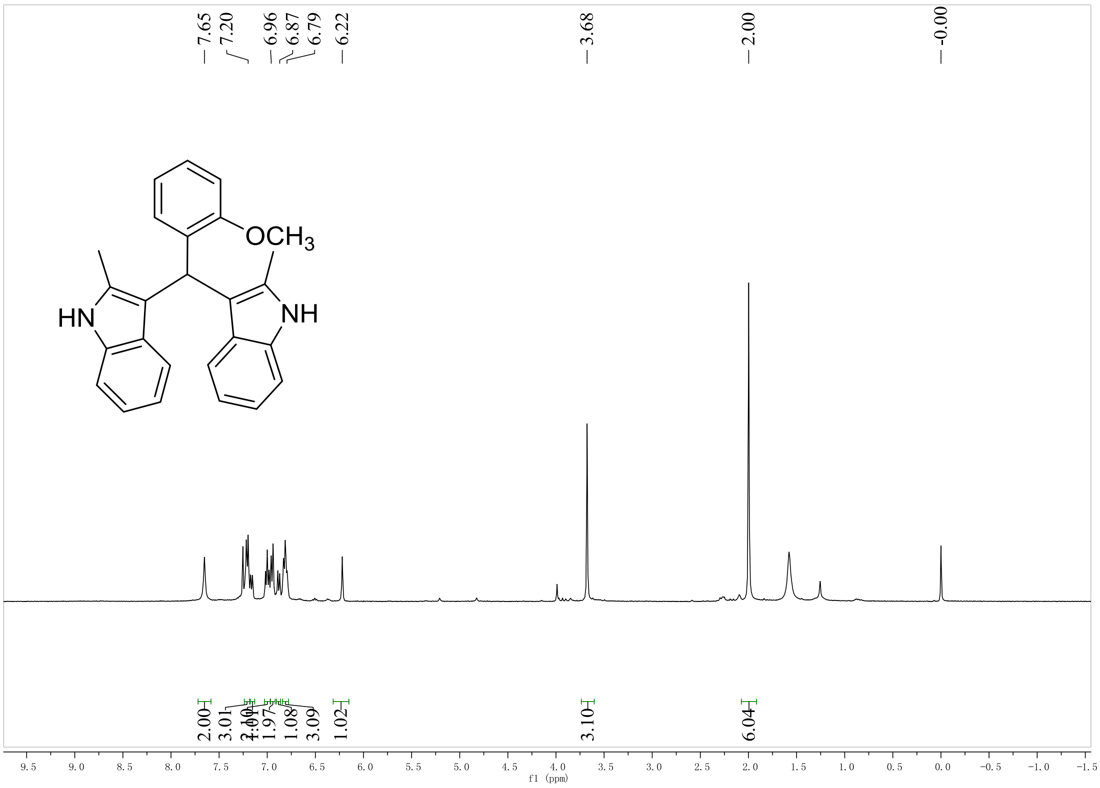

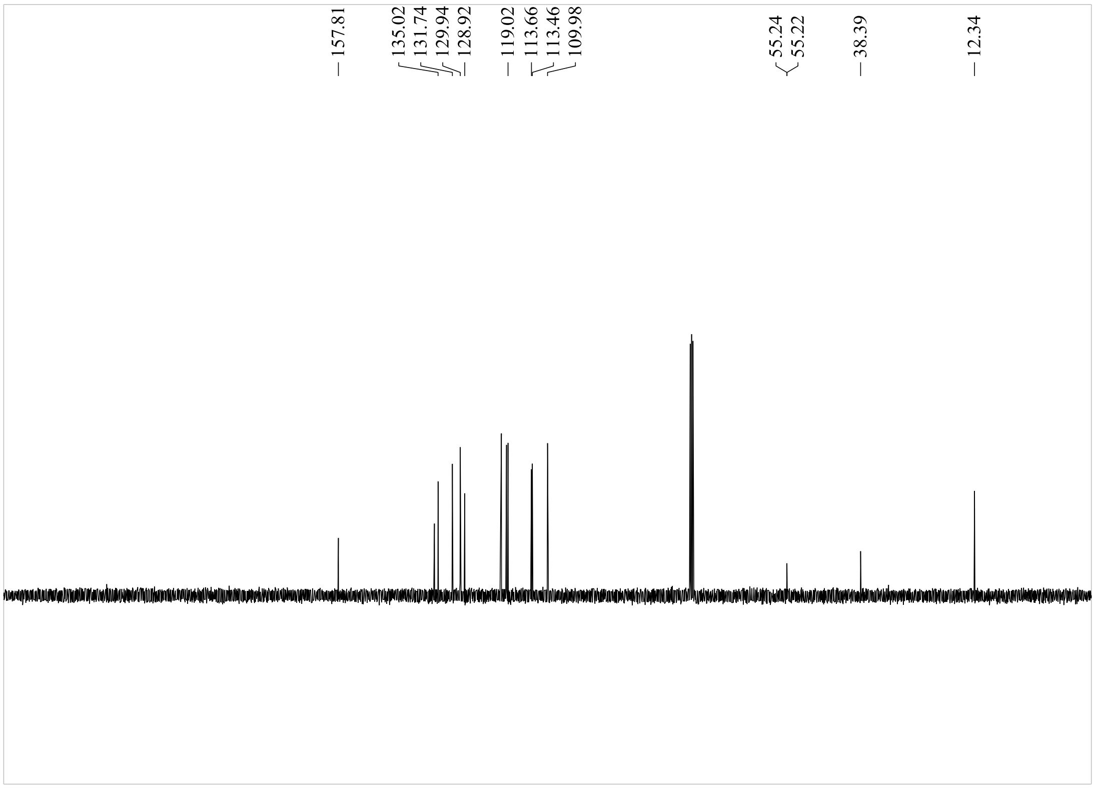

157.81
135.02
131.74
129.94
128.92
119.02
113.66
113.46
109.98
55.24
55.22
38.39
12.34

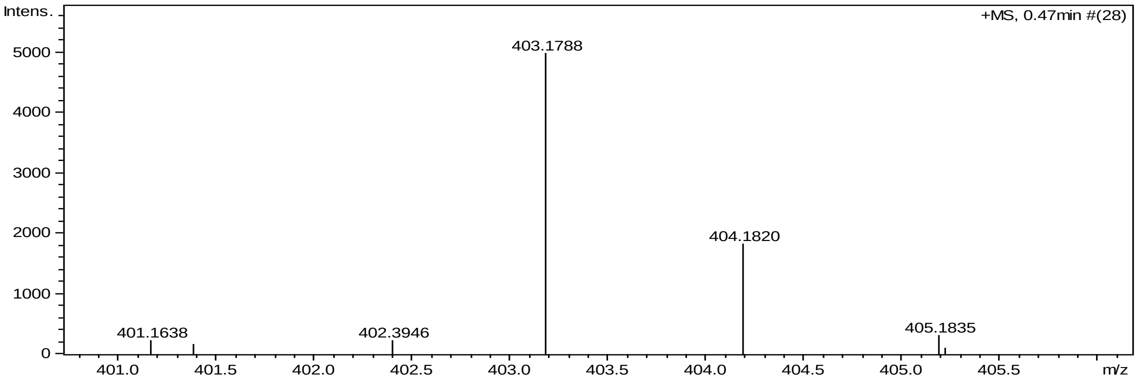

Intens.
+MS, 0.47min #(28)
403.1788
404.1820
401.1638
402.3946
405.1835
5000
4000
3000
2000
1000
0
401.0
401.5
402.0
402.5
403.0
403.5
404.0
404.5
405.0
405.5
m/z

3,3'-((3-Methoxyphenyl)methylene)bis(2-methyl-1H-indole) (6c):

purple solid, 71%, m.p. 179-181 °C; ^{1}H NMR (400 MHz, $CDCl_3$) δ: 7.72 (s, 2H, NH), 7.23 (d, J = 8.0 Hz, 2H, ArH), 7.16 (t, J = 8.0 Hz, 1H, ArH), 7.03 (t, J = 7.6 Hz, 4H, ArH), 6.86 (q, J = 7.2 Hz, 4H, ArH), 6.75 (dd, J_1 = 8.0 Hz, J_2 = 2.4 Hz, 1H, ArH), 5.98 (s, 1H, CH), 3.69 (s, 3H, OCH_3), 2.06 (s, 3H, CH_3), 2.05 (s, 3H, CH_3); ^{13}C NMR (400 MHz, $CDCl_3$) δ: 134.9, 131.7, 128.9, 128.9, 121.7, 120.5, 119.2, 119.0, 114.9, 113.2, 111.1, 109.8, 55.1, 39.2, 12.4; IR (KBr) υ: 3410, 3048, 2917, 2844, 1618, 1431, 1333, 1271, 1250, 745 cm^{-1}; MS (m/z): HRMS (ESI) Calcd. for $C_{26}H_{24}N_2O$ ($[M+Na]^+$): 403.1781, found: 403.1785.

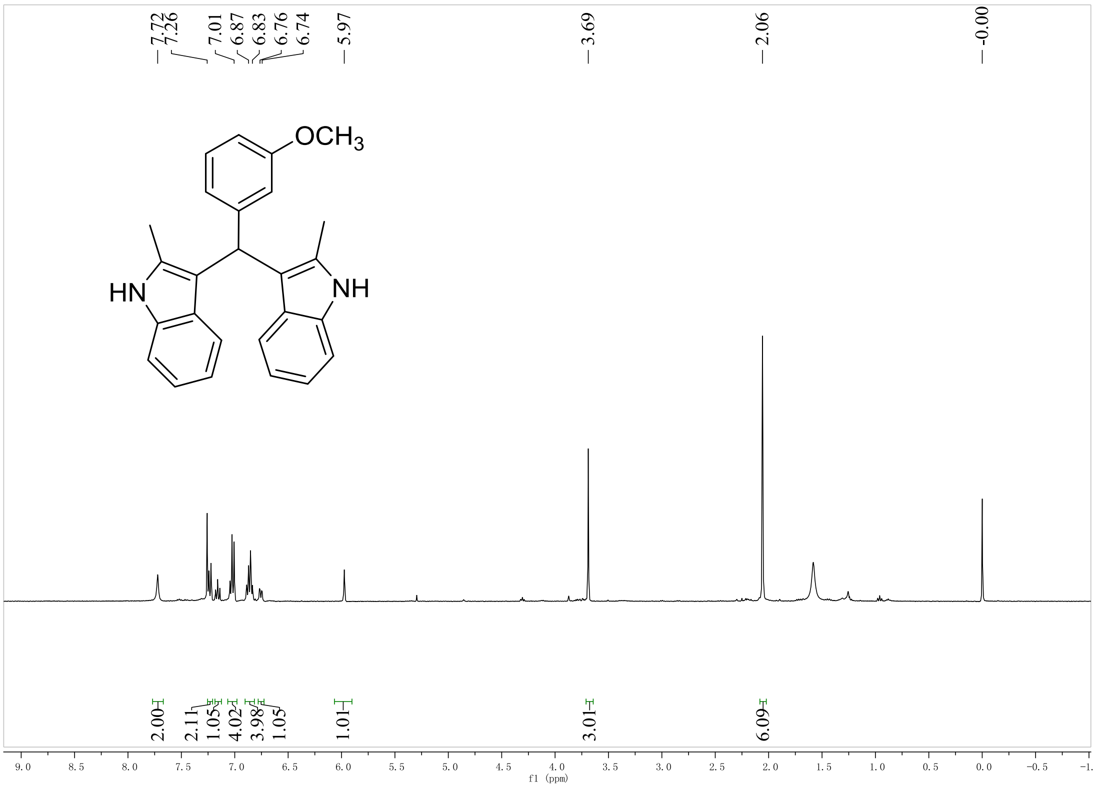

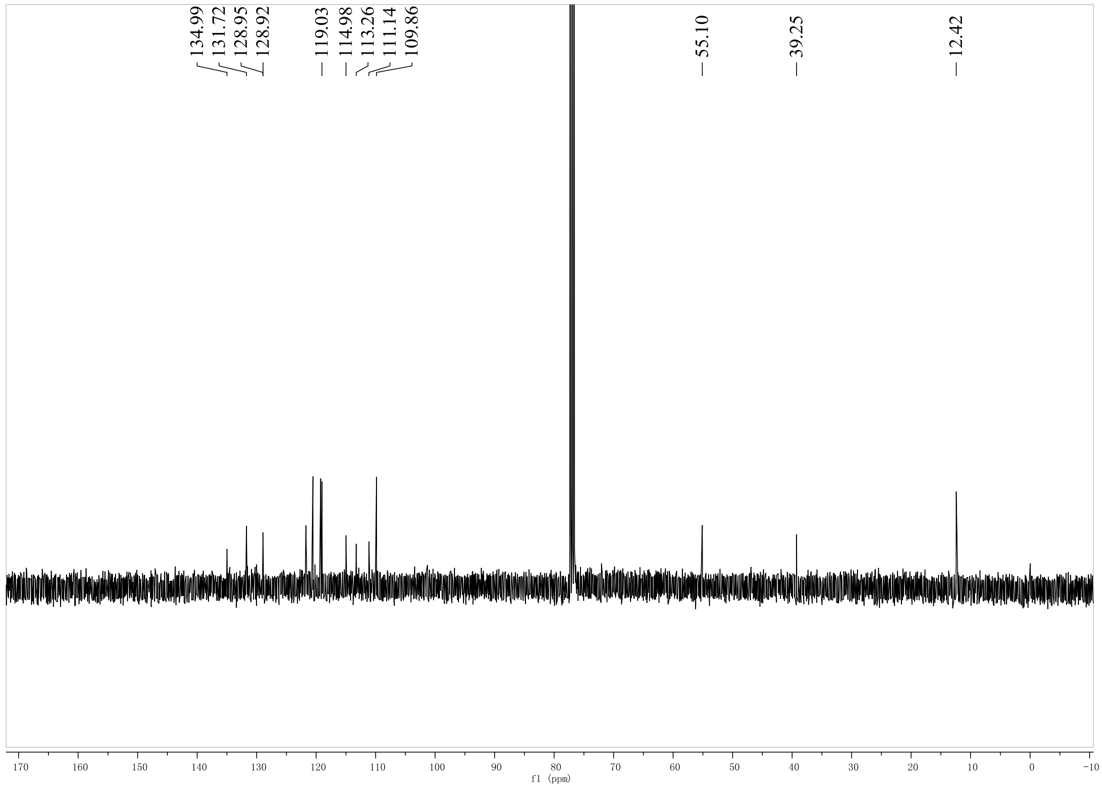
134.99
131.72
128.95
128.92
119.03
114.98
113.26
111.14
109.86
55.10
39.25
12.42
170
160
150
140
130
120
110
100
90
80
70
60
50
40
30
20
10
0
−10
f1 (ppm)

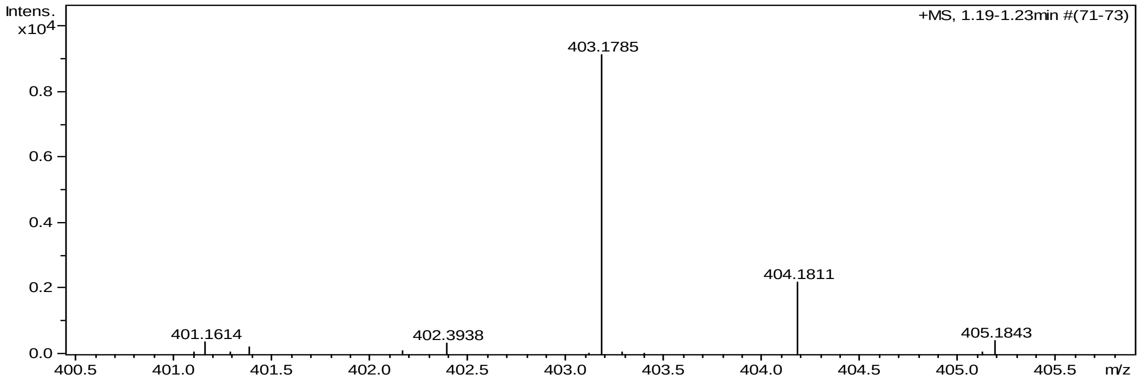
Intens.
x10^4
+MS, 1.19-1.23min #(71-73)
403.1785
404.1811
401.1614
402.3938
405.1843
0.8
0.6
0.4
0.2
0.0
400.5
401.0
401.5
402.0
402.5
403.0
403.5
404.0
404.5
405.0
405.5
m/z

4-(Bis(2-methyl-1H-indol-3-yl)methyl)-N,N-dimethylaniline (6d):

purple solid, 70%, m.p. 184-186 °C; ^{1}H NMR (400 MHz, $CDCl_3$) δ: 7.61 (s, 2H, NH), 7.18 (d, *J* = 8.0 Hz, 2H, ArH), 7.10 (d, *J* = 8.0 Hz, 2H, ArH), 7.04-6.99 (m, 4H, ArH), 6.84 (t, *J* = 7.2 Hz, 2H, ArH), 6.64 (d, *J* = 8.4 Hz, 2H, ArH), 5.90 (s, 1H, CH), 2.89 (s, 3H, CH_3), 2.88 (s, 3H, CH_3), 2.01 (s, 3H, CH_3), 2.00 (s, 3H, CH_3); ^{13}C NMR (400 MHz, $CDCl_3$) δ: 148.9, 135.0, 132.0, 131.6, 129.5, 129.0, 120.3, 119.4, 118.8, 114.0, 112.7, 109.8, 40.9, 38.2, 12.3; IR (KBr) υ: 3387, 2918, 2771, 1275, 1270, 1260, 760, 750 cm^{-1}; MS (*m/z*): HRMS (ESI) Calcd. for $C_{27}H_{27}N_3$ ($[M+Na]^+$): 416.2097, found: 416.2099.

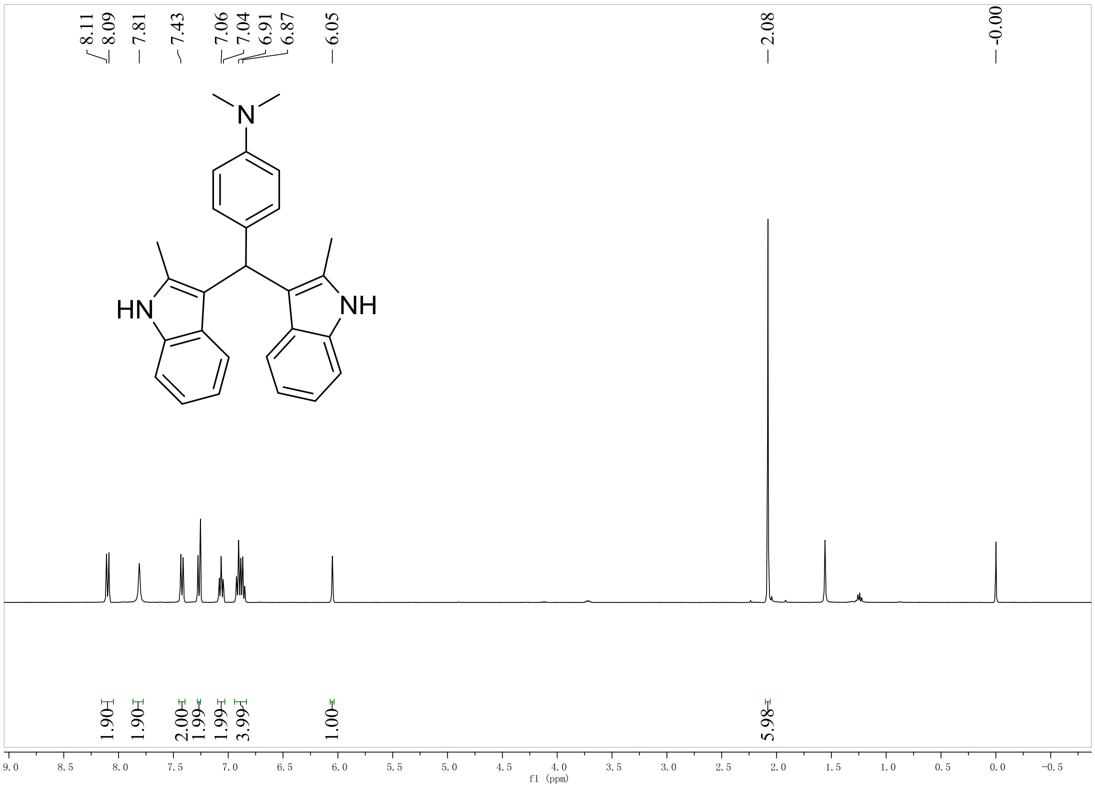

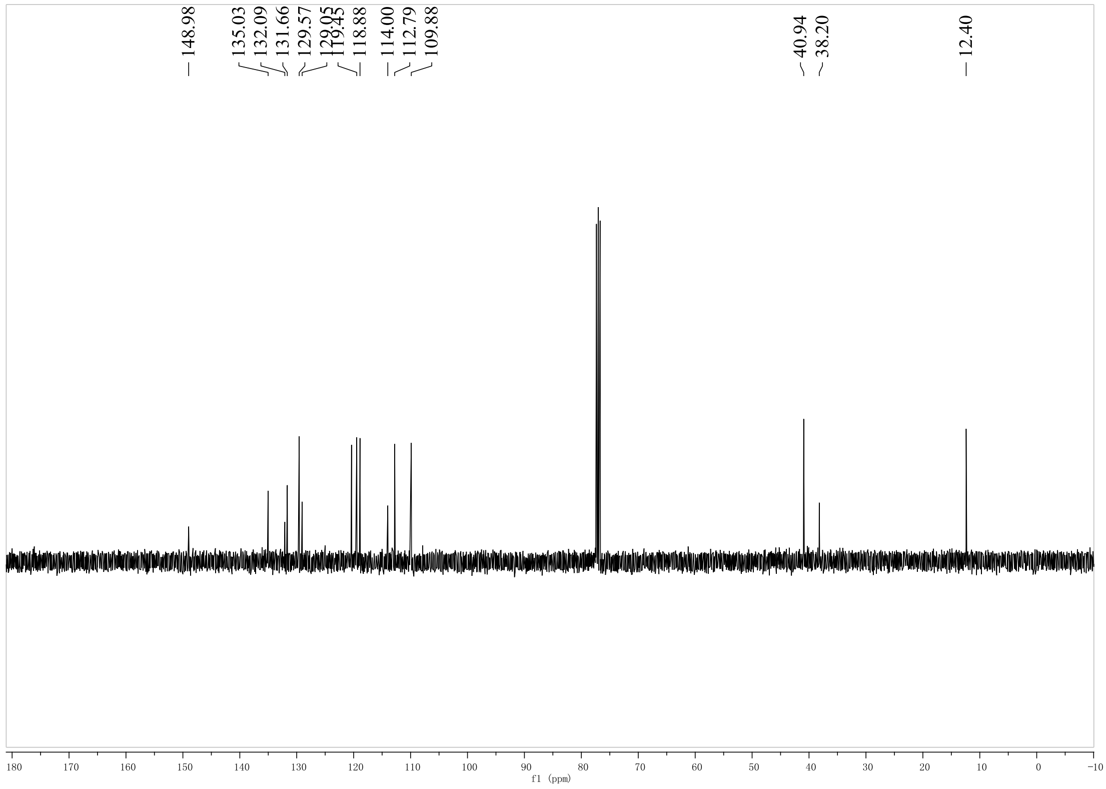
148.98
135.03
132.09
131.66
129.57
129.05
119.45
118.88
114.00
112.79
109.88
40.94
38.20
12.40
180 170 160 150 140 130 120 110 100 90 80 70 60 50 40 30 20 10 0 −10
f1 (ppm)

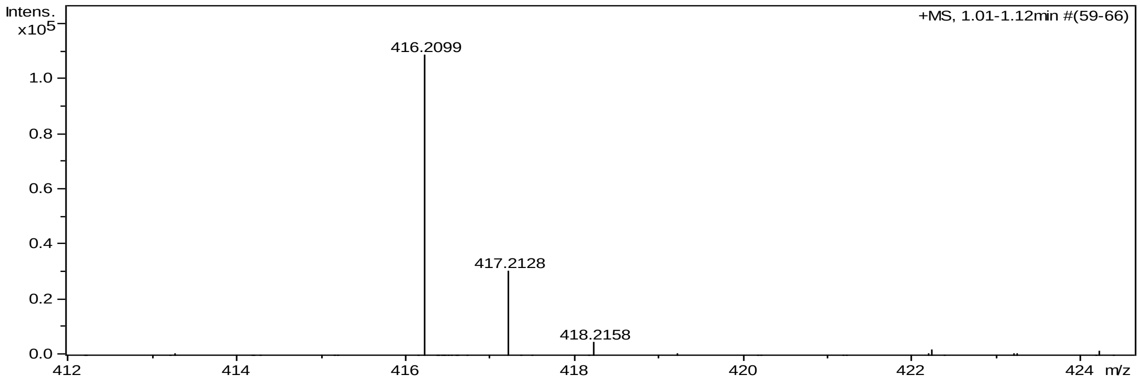
Intens.
x10^5
+MS, 1.01-1.12min #(59-66)
416.2099
417.2128
418.2158
1.0
0.8
0.6
0.4
0.2
0.0
412 414 416 418 420 422 424 m/z

3,3'-((4-Nitrophenyl)methylene)bis(2-methyl-1H-indole) (6e):

purple solid, 65%, m.p. 187-189 °C; ^{1}H NMR (400 MHz, $CDCl_3$) δ: 8.10 (d, *J* = 8.8 Hz, 2H, ArH), 7.81 (s, 2H, NH), 7.42 (d, *J* = 8.4 Hz, 2H, ArH), 7.26 (d, *J* = 8.4 Hz, 2H, ArH), 7.06 (t, *J* = 8.0 Hz, 2H, ArH), 6.93-6.87 (m, 4H, ArH), 6.05 (s, 1H, CH), 2.08 (s, 3H, CH_3), 2.07 (s, 3H, CH_3); ^{13}C NMR (400 MHz, $CDCl_3$) δ: 151.9, 135.0, 132.0, 129.8, 128.4, 123.4, 120.9, 119.4, 118.9, 111.8, 110.2, 39.3, 12.4; IR (KBr) υ: 3393, 2919, 2878, 1645, 1548, 1525, 1459, 1345, 749 cm^{-1}; MS (*m/z*): HRMS (ESI) Calcd. for $C_{25}H_{21}N_3O_2$ ($[M+Na]^+$): 418.1526, found: 418.1528.

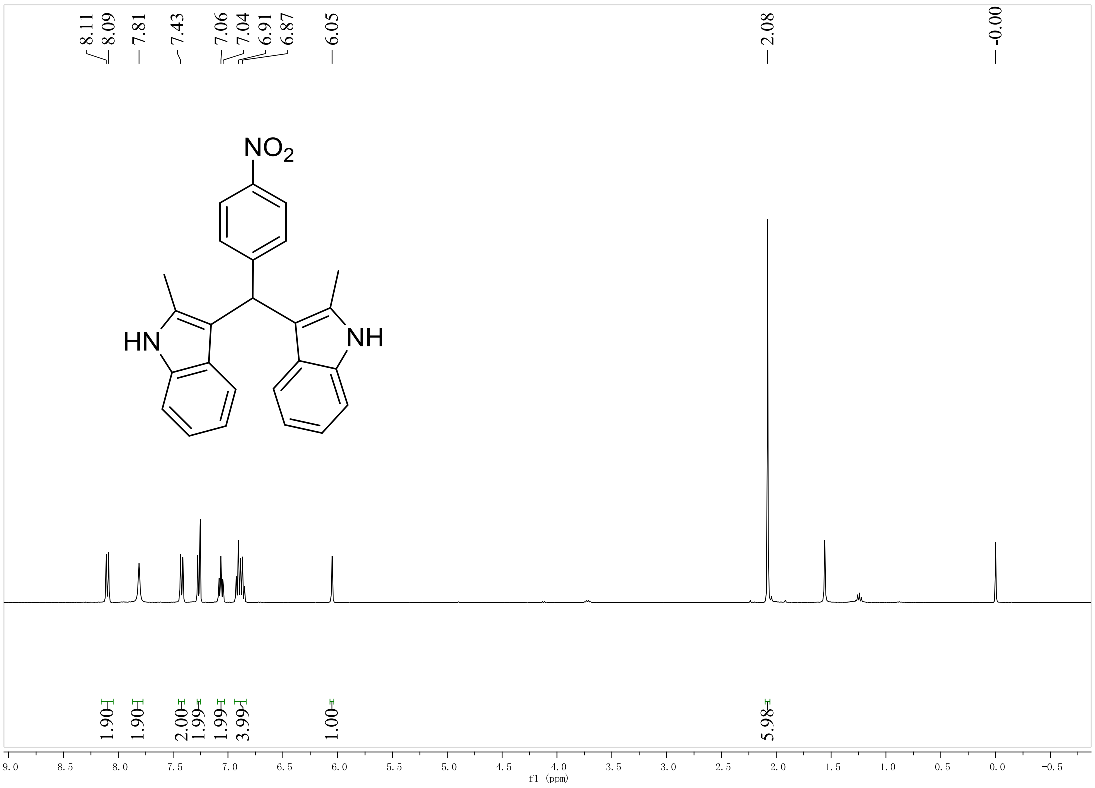

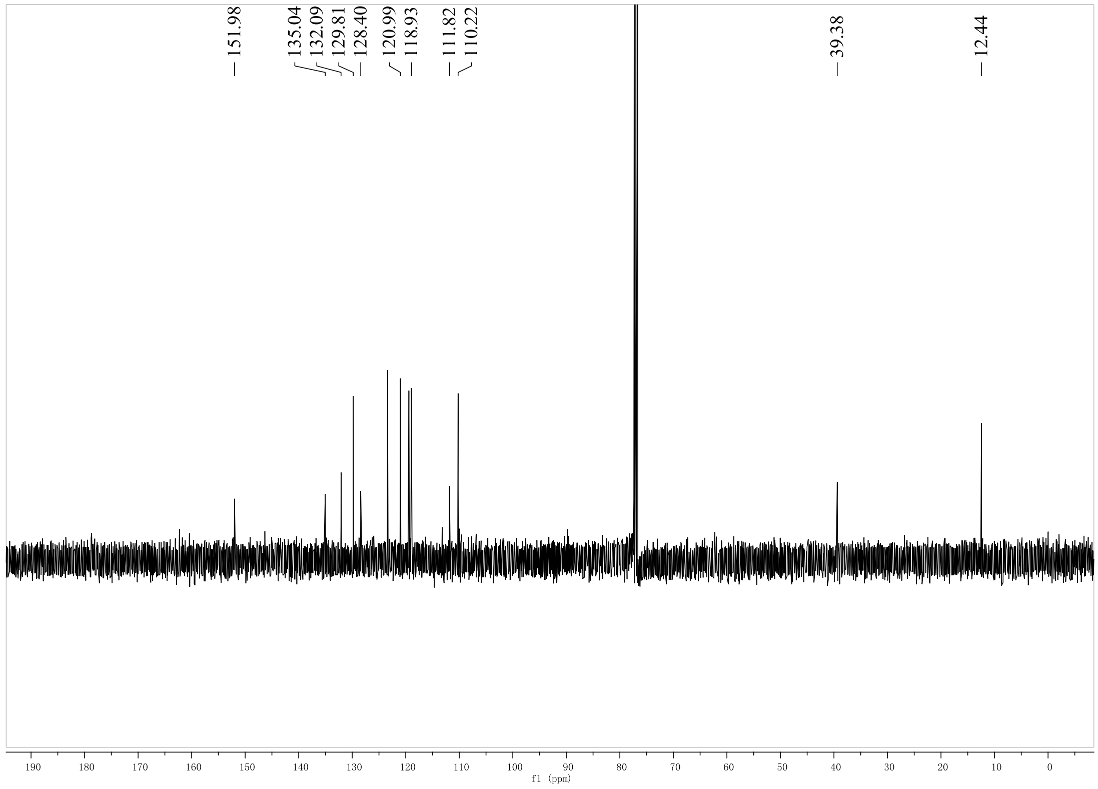
151.98
135.04
132.09
129.81
128.40
120.99
118.93
111.82
110.22
39.38
12.44
190
180
170
160
150
140
130
120
110
100
90
80
70
60
50
40
30
20
10
0
f1 (ppm)

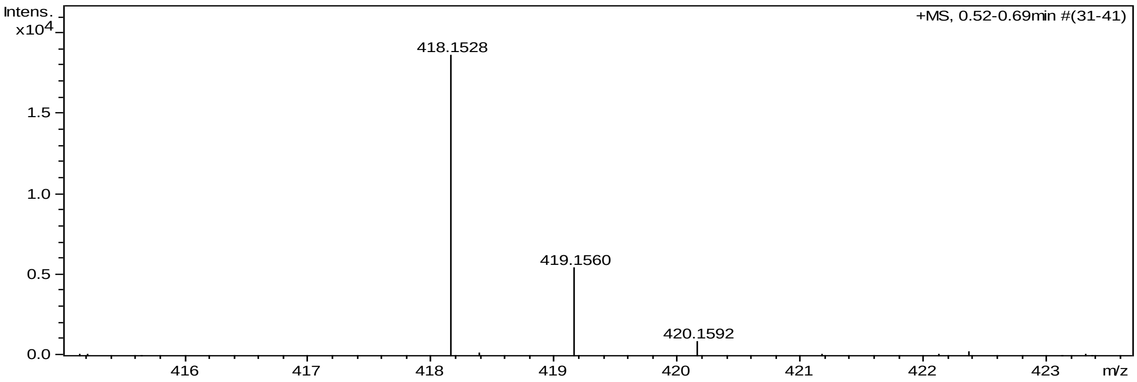
Intens.
x10^4
+MS, 0.52-0.69min #(31-41)
418.1528
419.1560
420.1592
1.5
1.0
0.5
0.0
416
417
418
419
420
421
422
423
m/z

3,3'-((3-Chlorophenyl)methylene)bis(2-methyl-1H-indole) (6f):

purple solid, 70%, m.p. 175-177 ºC; ^{1}H NMR (400 MHz, CDCl$_3$) δ: 7.76 (s, 2H, NH), 7.26-7.24 (m, 3H, ArH), 7.19-7.17 (m, 3H, ArH), 7.05 (t, *J* = 7.2 Hz, 2H, ArH), 6.97 (d, *J* = 8.0 Hz, 2H, ArH), 6.89-6.85 (m, 2H, ArH), 5.97 (s, 1H, CH), 2.06 (s, 3H, CH$_3$), 2.05 (s, 3H, CH$_3$); ^{13}C NMR (400 MHz, CDCl$_3$) δ: 142.3, 135.0, 131.8, 130.3, 128.7, 128.1, 120.7, 119.1, 119.1, 112.8, 110.0, 38.6, 12.4; IR (KBr) υ: 3412, 2925, 2853, 1617, 1474, 1337, 1281, 1251, 787, 744, 787 cm^{-1}; MS (*m/z*): HRMS (ESI) Calcd. for $C_{25}H_{21}Cl^{35}N_2$ ([M+Na]$^+$): 407.1285, found: 407.1290. $C_{25}H_{21}Cl^{37}N_2$ ([M+Na]$^+$): 409.1256, found: 409.1268.

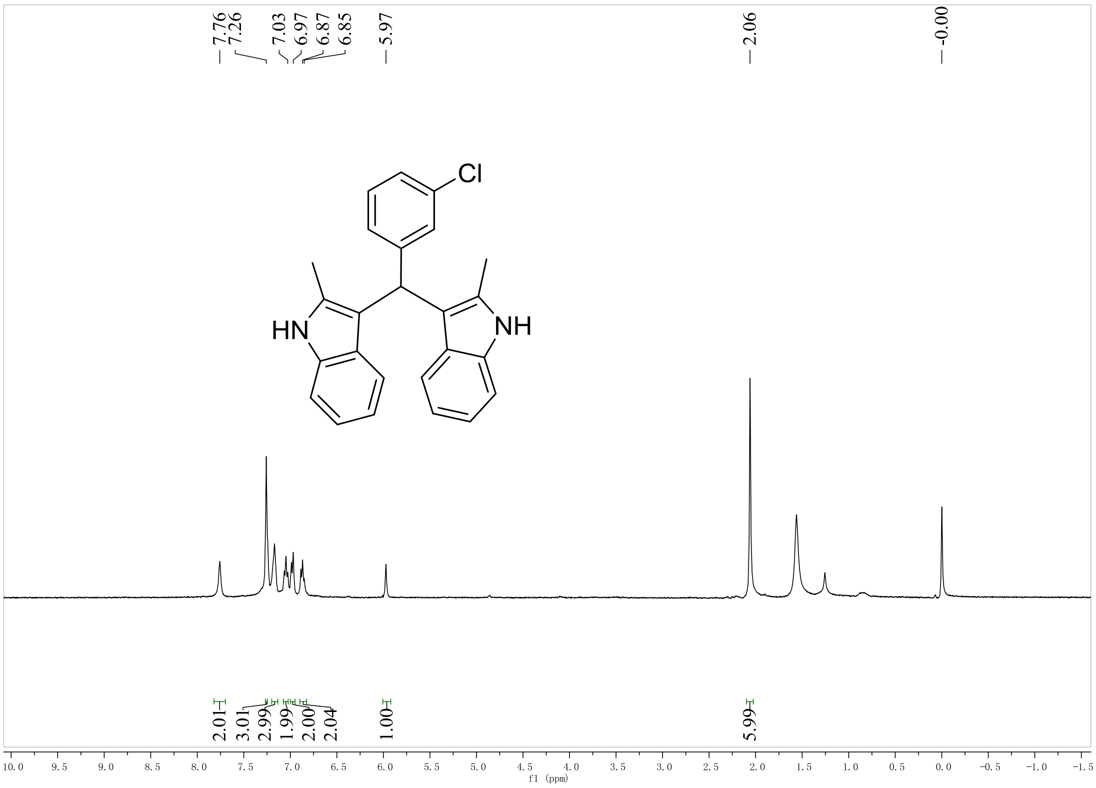

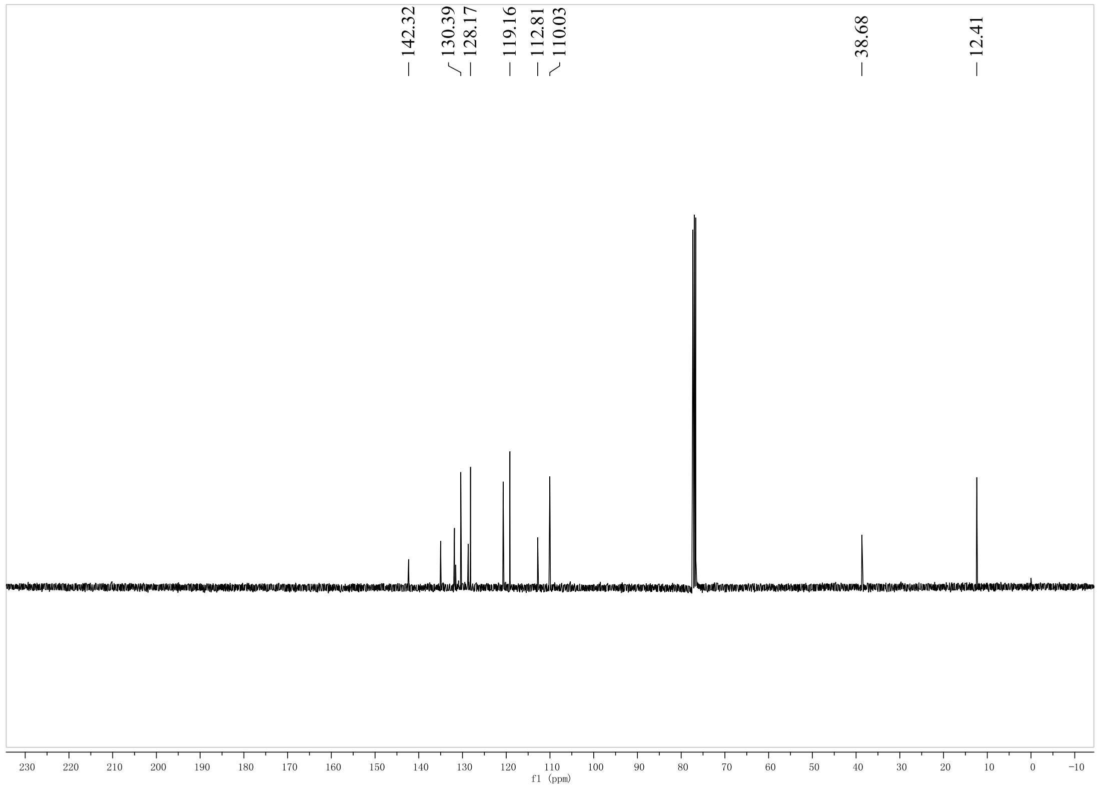

142.32
130.39
128.17
119.16
112.81
110.03
38.68
12.41
f1 (ppm)

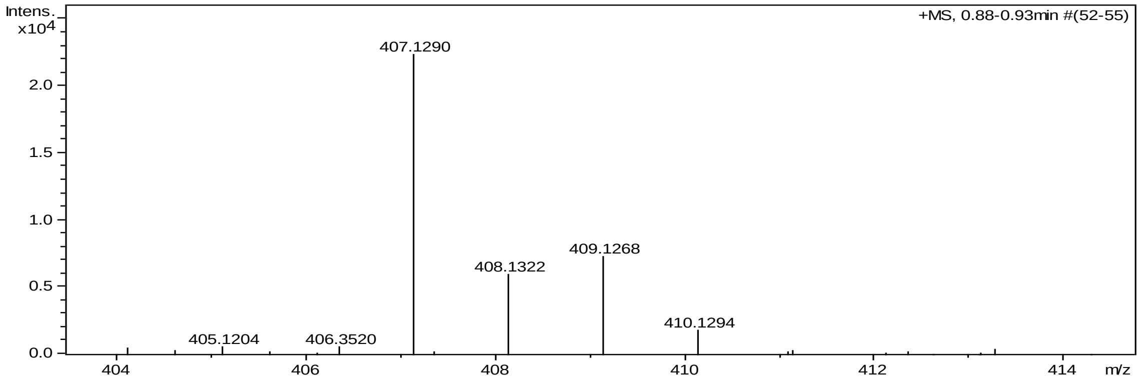

Intens.
x10^4
+MS, 0.88-0.93min #(52-55)
407.1290
408.1322
409.1268
410.1294
405.1204
406.3520
m/z

3,3'-((4-Chlorophenyl)methylene)bis(2-methyl-1H-indole) (6g):

purple solid, 76%, m.p. 170-172 °C; ^{1}H NMR (400 MHz, $CDCl_3$) δ: 7.74 (s, 2H, NH), 7.24 (d, *J* = 8.8 Hz, 3H, ArH), 7.20-7.18 (m, 3H, ArH), 7.04 (t, *J* = 7.2 Hz, 2H, ArH), 6.96 (d, *J* = 7.6 Hz, 2H, ArH), 6.86 (t, *J* = 7.6 Hz, 2H, ArH), 5.95 (s, 1H, CH), 2.05 (s, 3H, CH_3), 2.04 (s, 3H, CH_3); ^{13}C NMR (400 MHz, $CDCl_3$) δ: 142.3, 135.0, 131.8, 130.3, 128.7, 128.1, 120.7, 119.1, 112.8, 110.0, 38.6, 12.4; IR (KBr) υ: 3400, 2917, 2780, 1523, 1318, 1260, 749.8 cm^{-1}; MS (*m*/*z*): HRMS (ESI) Calcd. for $C_{25}H_{21}Cl^{35}N_2$ ($[M+Na]^+$): 407.1285, found: 407.1287. $C_{25}H_{21}Cl^{37}N_2$ ($[M+Na]^+$): 409.1256, found: 409.1267.

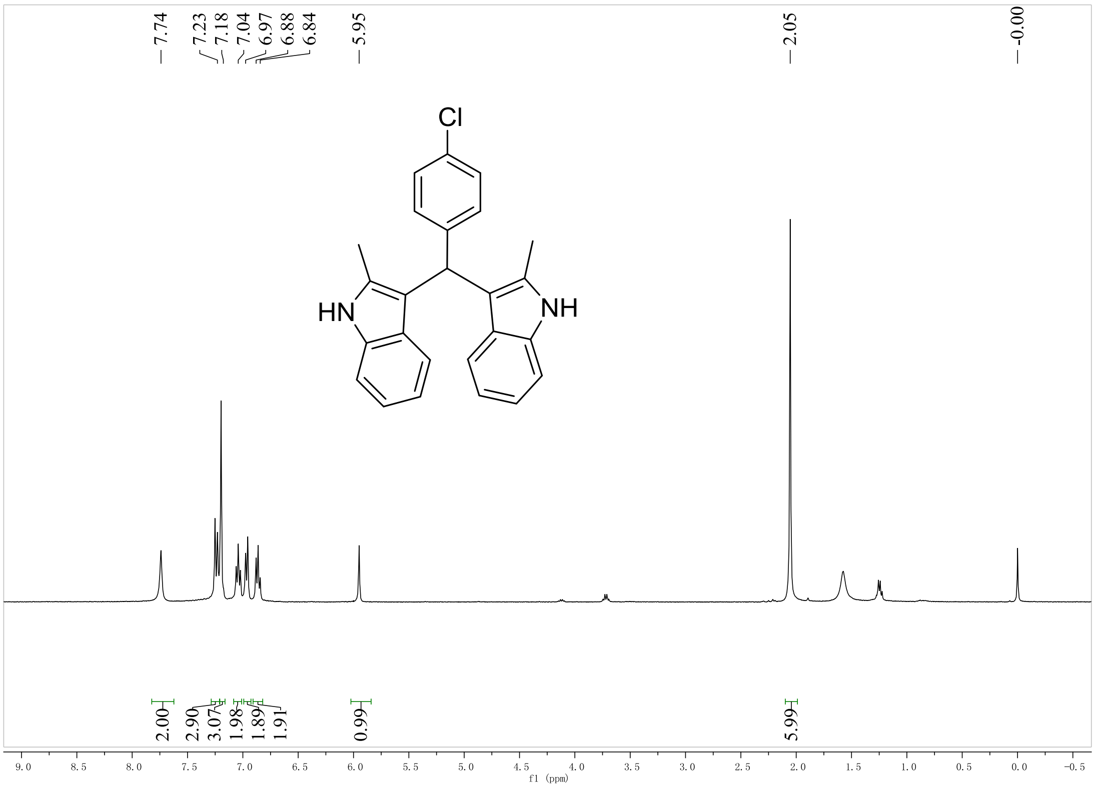

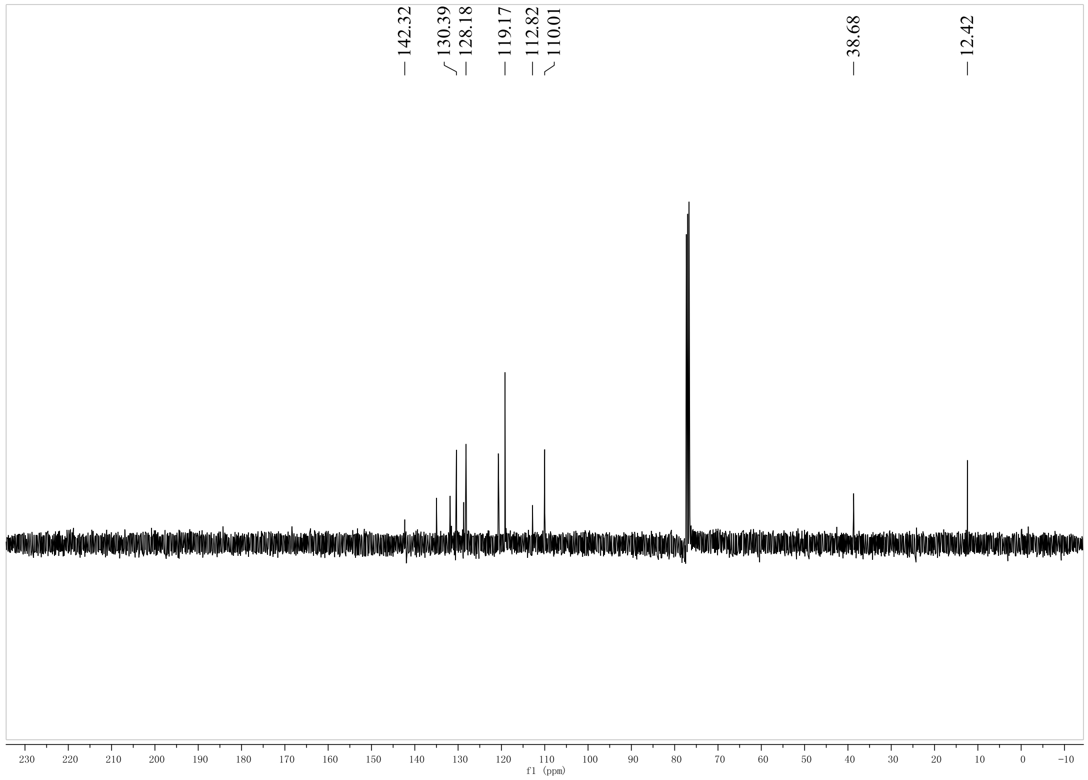
142.32
130.39
128.18
119.17
112.82
110.01
38.68
12.42
230 220 210 200 190 180 170 160 150 140 130 120 110 100 90 80 70 60 50 40 30 20 10 0 -10
f1 (ppm)

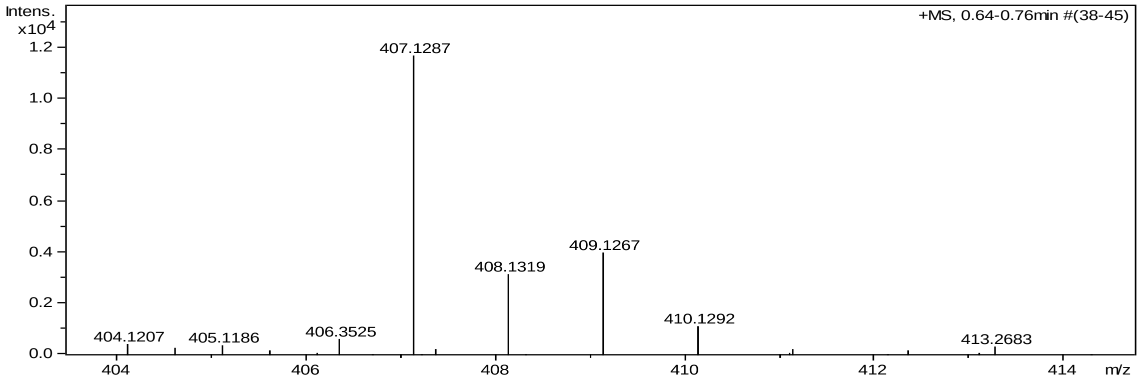
Intens.
x10^4
+MS, 0.64-0.76min #(38-45)
407.1287
408.1319
409.1267
410.1292
404.1207
405.1186
406.3525
413.2683
1.2
1.0
0.8
0.6
0.4
0.2
0.0
404 406 408 410 412 414 m/z
